# Pd/C-catalyzed regiodivergent hydrocarboxylation and esterification of alkynes†

**DOI:** 10.1039/d4sc05549g

**Published:** 2024-10-09

**Authors:** Pushkar Mehara, Poonam Sharma, Rohit Bains, Ajay Kumar Sharma, Pralay Das

**Affiliations:** a Chemical Technology Division, CSIR-Institute of Himalayan Bioresource Technology Palampur 176061 H.P. India pdas@ihbt.res.in pralaydas1976@gmail.com +91-1894-230433; b Academy of Scientific and Innovative Research (AcSIR) Ghaziabad-201002 India

## Abstract

An unprecedented and highly reactive Pd/C catalytic system has been introduced for the regiodivergent hydrocarboxylation of terminal alkynes to selectively afford various acrylic and cinnamic acids employing oxalic acid as a CO source as well as a promoter for the formation of the active Pd–H complex. Herein, the formation of cinnamic acid is proposed to follow a unique anti-Markovnikov hydroiodination mechanism and the formation of acrylic acid might follow the traditional hydrocarboxylation pathway. Additionally, internal alkynes undergo hydrocarboxylation and carbonylative esterification with aliphatic alcohols to yield different α,β-unsaturated acids and esters respectively. The designed strategies were successfully leveraged for a diverse class of α,β-unsaturated acids and esters with excellent selectivity and yields under mild reaction conditions. Furthermore, the acid functionalization of complicated naturally derived alkynes, utilizing economical and bench-stable oxalic acid and a commercially accessible reusable catalyst with gram-scale applicability are the additional benefits of the established protocol.

## Abstract 2

A Pd/C-catalyzed regiodivergent hydrocarboxylation of terminal alkynes to cinnamic and acrylic acids as well as hydroxy/alkoxy carbonylation of internal alkynes to α,β-unsaturated acids/esters have been devised using oxalic acid as a CO source.

## Graphical abstract

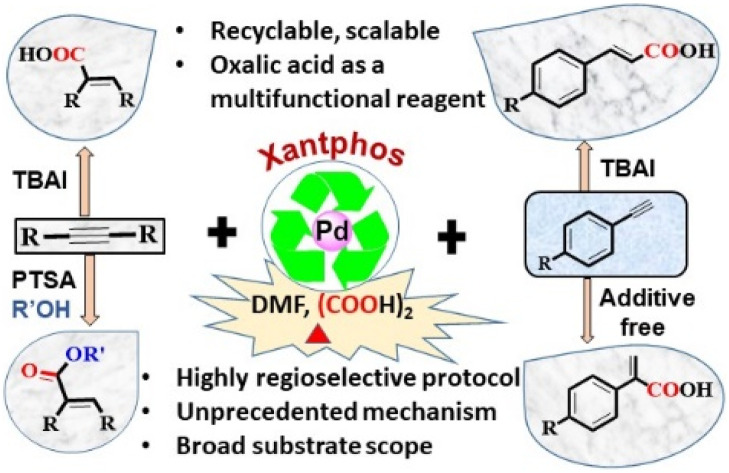

## Introduction

**Scheme 1:**
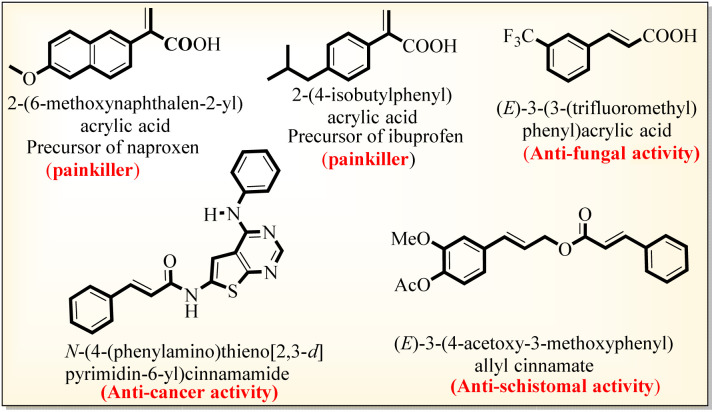
Selected biologically active acrylic and cinnamic acid derivatives.

The α,β-unsaturated acid and ester scaffolds are essential building blocks present in pharmaceutically and biologically active molecules.^1^ The acrylic acid class of α,β-unsaturated acids has extensive biological and industrial applications, including in the synthesis of adhesives, coatings, and polymer chemistry,^1,2^ while the other class of naturally occurring cinnamic acids exhibits antidiabetic, anti-inflammatory, antioxidant, and anticancer activities (Scheme 1).^3^

In addition, α,β-unsaturated carboxylates are key compounds for the production of perfumes, agrochemicals, pharmaceuticals, materials, and polymers.^4^

**Scheme 2:**
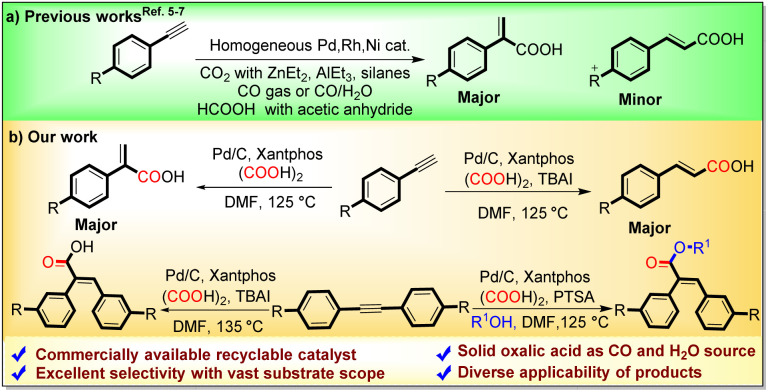
Various carbonylative protocols for the synthesis of α,β-unsaturated carboxylic acids.

The carbonylative functionalization of alkynes is an important synthetic research area, as the direct functionalization of alkenes is quite difficult due to their less chemical reactivity. However, the regio- and stereoselective carbonylative transformation of alkynes to α,β-unsaturated acids is still challenging due to multiple active sites, encouraging researchers to devise novel catalytic systems to address these selectivity limitations.^1^ The metal-catalyzed hydrocarboxylation of alkynes with naturally abundant and economical CO_2_ is a well-known straightforward technique to access α,β-unsaturated acids; however, it requires a stoichiometric amount of harsh organometallic reagents such as silanes,^5*a*,*b*^ ZnEt_2_,^5*c*,*d*^ EtMgBr,^5*e*^ or direct metals (Mn and Zn)^5*f*–*i*^ as reducing agents (Scheme 2a).

Another way to acquire α,β-unsaturated acids is through the carbonylation of alkynes using direct CO gas. However, to handle the toxic and flammable CO gas, highly sophisticated instruments are required.^6^ To avoid limitations associated with the direct use of CO gas, Zhou and Fu's research groups reported the use of formic acid for hydrocarboxylation of alkynes to α,β-unsaturated acids with the aid of homogeneous Pd(OAc)_2_ and Ni(acac)_2_ catalysts respectively.^7^ However, to utilize liquid formic acid as a CO surrogate, acetic anhydride is essentially required as an activator, which subsequently generates acetic acid as a byproduct.

Although numerous uniform Pd-catalysts have been applied in the hydrocarboxylation and carbonylative esterification of alkynes, there remains an untapped domain regarding a heterogeneous Pd catalytic setup and a regiodivergent route for generating acrylic and cinnamic acids from alkynes. Additionally, using a homogeneous catalytic system and direct CO gas, the carbonylative esterification coupling of alkynes with aliphatic alcohols is a well-known and straightforward route for the synthesis of α,β-unsaturated carboxylates.^8^ However, their synthesis using a suitable CO surrogate and heterogeneous catalysis has not been reported yet.

As part of our research aims to develop direct CO gas-free carbonylation reactions, we are continuously exploring oxalic acid as a bench stable, economical CO source in various carbonylation reactions in an *in situ* as well as *ex situ* manner.^9^ Unlike the other CO sources, the thermal decomposition of oxalic acid is activator-free and does not require any additives such as anhydrides, bases or metals, to liberate the CO gas.^9,10^ Furthermore, comparing the cost and recyclability of homogeneous Pd catalysts, the readily available Pd/C can provide an alternative, cost-efficient and greener pathway because of its high stability, safe handling and easy recycling.

In this regard, herein, we have established novel Pd/C-catalyzed robust strategies for regiodivergent hydrocarboxylation of alkynes into cinnamic and acrylic acids as well as the carbonylative esterification of internal alkynes with aliphatic alcohols to yield α,β-unsaturated esters with oxalic acid serving as a C1 building block.

At the outset, we selected phenylacetylene (1a, 0.49 mmol, 1 equiv.) as the model substrate to react with CO gas generated *in situ* from oxalic acid (5 equiv.) and Pd/C (3 mol%) as a catalyst along with additive TBAI (1 equiv.) and BINAP ligand (6 mol%) in DMF (2 mL) heated at 125 °C for 24 h. This reaction yielded a mixture of α,β-unsaturated carboxylic acids in 60% yield *i.e.*, cinnamic acid (2a) and 2-phenylacrylic acid (3a) with 74% and 26% selectivity respectively, and in the absence of the ligand, the desired product did not form (Table 1, entries 1 and 2).

**Table 1:** Optimization of the reaction conditions^a^

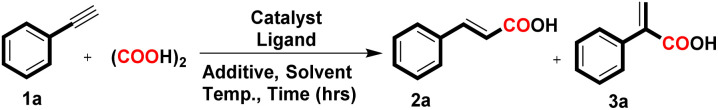							
S. no.	Catalyst	Ligand	Additive	Solvent	Selectivity		Overall yield^b^ (%)
					2a	3a	
1	Pd/C	BINAP	TBAI	DMF	74	26	60
2	Pd/C	—	TBAI	DMF	nd	nd	0
3	Pd/C	DPEphos	TBAI	DMF	80	20	57
4	Pd/C	dppe	TBAI	DMF	78	22	52
5	Pd/C	PPh_3_	TBAI	DMF	Traces	Traces	Traces
6	Pd/C	Xantphos	TBAI	DMF	82	18	75
7	Pd/C	PCy_3_	TBAI	DMF	Traces	Traces	Traces
8	Pd/C	Xantphos	TBAI(0.5)	DMF	82	18	75
**9** c	**Pd/C**	**Xantphos**	**TBAI(0.5)**	**DMF**	**84**	**16**	**85, 97** d
**10**	**Pd/C**	**Xantphos**	—	**DMF**	—	**100**	**78, 84** d
11^e^	Pd/C	Xantphos	—	DMF	Traces	100	64
12	Pd/Al_2_O_3_	Xantphos	TBAI(0.5)	DMF	82	18	79
13	Pd(OAc)_2_	Xantphos	TBAI(0.5)	DMF	84	16	75
14	Pd(PPh_3_)_2_Cl_2_	Xantphos	TBAI(0.5)	DMF	83	17	82
15	Pd(PPh_3_)_4_	Xantphos	TBAI(0.5)	DMF	78	22	59
16	Pd_2_(dba)_3_	Xantphos	TBAI(0.5)	DMF	75	25	66
17^c^	Pd/C	Xantphos	KI(0.5)	DMF	76	24	70
18^c^	Pd/C	Xantphos	NaI(0.5)	DMF	72	28	62

a Reaction conditions: phenylacetylene (1 equiv.), catalyst (3 mol%), ligand (6 mol%), additive (1.0 equiv.), oxalic acid (5 equiv.), and DMF (2 mL) at 125 °C for 24 h; TBAI = tetra-*n*-butylammonium iodide, BINAP = (2,2′-bis(diphenylphosphino)-1,1′-binaphthyl), DPEphos = bis[phenyl]ether, dppp = 1,3-bis(diphenylphosphino)propane, PPh_3_ = 1,2-bis(diphenylphosphino)ethane, Xantphos = 4,5-bis(diphenylphosphino)-9,9 dimethylxanthene.

b Isolated yields.

c Oxalic acid (3 equiv.).

d GC yield.

e Xantphos (3 mol%); nd = not detected.

Considering the importance of ligands for the desired product formation, we screened a series of bidentate phosphine ligands with the Pd/C catalyst. Bidentate phosphine ligands with different bite angles such as DPEphos, dppe, PPh_3_, Xantphos, and PCy_3_ were investigated, and Xantphos was found to be the most suitable ligand, delivering an overall 75% yield of 2a and 3a with 82% and 18% selectivity respectively (Table 1, entries 3–7). These findings revealed that the steric effect of the phosphine ligands has no profound effect on the formation of 2a. On reducing the equivalency of TBAI from 1.0 equiv. to 0.5 equiv., the yield of the desired product was not affected, which might result from TBAI recycling (Table 1, entry 8). After optimizing the other reaction parameters such as catalyst loading, time, temperature, solvent and oxalic acid equivalency, we have determined the optimized conditions as follows: Pd/C (3 mol%), Xantphos (6 mol%), oxalic acid (3.0 equiv.) and TBAI (0.5 equiv.) in DMF solvent at 125 °C for 18 h, which gives 85% isolated yield of 2a and 3a with 84% and 16% selectivity respectively *i.e.*, condition A (Table 1, entry 9 and Tables S1–S7, see ESI†). To our delight, on eliminating the additive, the regioselectivity of the product became reversed and 2-phenylacrylic acid (3a) was obtained as the major product with excellent 100% selectivity and 78% isolated yield *i.e.* condition B (Table 1, entry 10). In pursuit of high reaction yield, other palladium catalysts such as Pd/Al_2_O_3_, Pd(OAc)_2_, Pd(PPh_3_)_2_Cl_2_, Pd(PPh_3_)_4_, and Pd_2_(dba)_3_ were also examined, and Pd/C was found to be more effective and selective for the desired product formation (Table 1, entries 12–16). Other additives such as KI and NaI were also tested instead of TBAI; however, they delivered an overall yield of 70% and 62% with 76% and 72% selectivity for 2a respectively (Table 1, entries 17 and 18). The combination of Pd/C and phosphine ligands has been previously well explored in different chemical transformations,^11^ and in the present protocol, Pd/C and Xantphos in combination with oxalic acid is found to be more effective and selective for the desired acid transformation. After establishing the ideal reaction conditions for 2a and 3a, we scrutinized the robustness of the developed protocols for stereoselective hydrocarboxylation of diversely substituted terminal alkynes using conditions A and B, and the results are illustrated in Tables 2 and 3 respectively.

**Table 2:** Substrate scope for cinnamic acids^a^

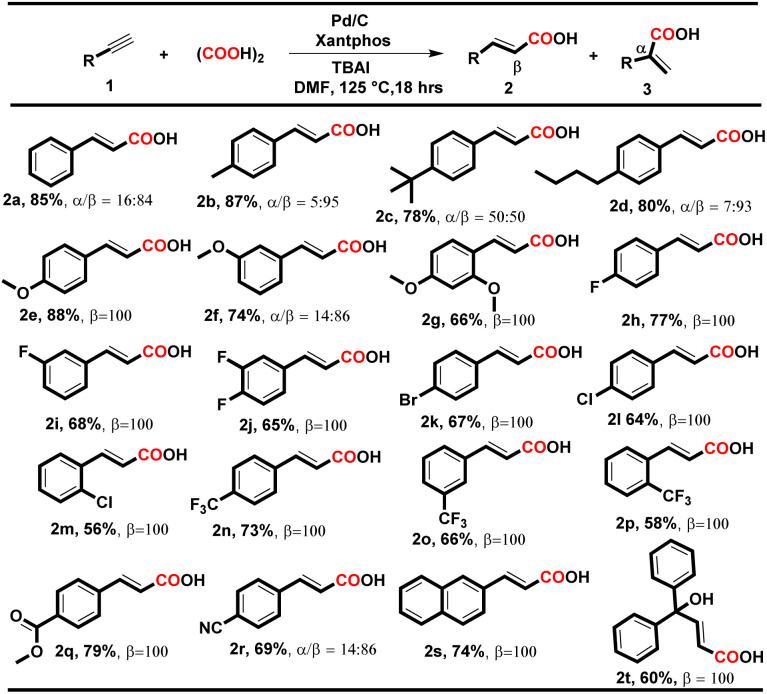

a Reaction conditions: 1 (1.0 equiv.), oxalic acid (3.0 equiv.), Pd/C (3 mol%), Xantphos (6 mol%), TBAI (0.5 equiv.), and 2.0 mL of DMF; heated for 18 h at 125 °C.

**Table 3:** Substrate scope for phenyl acrylic acids^a^

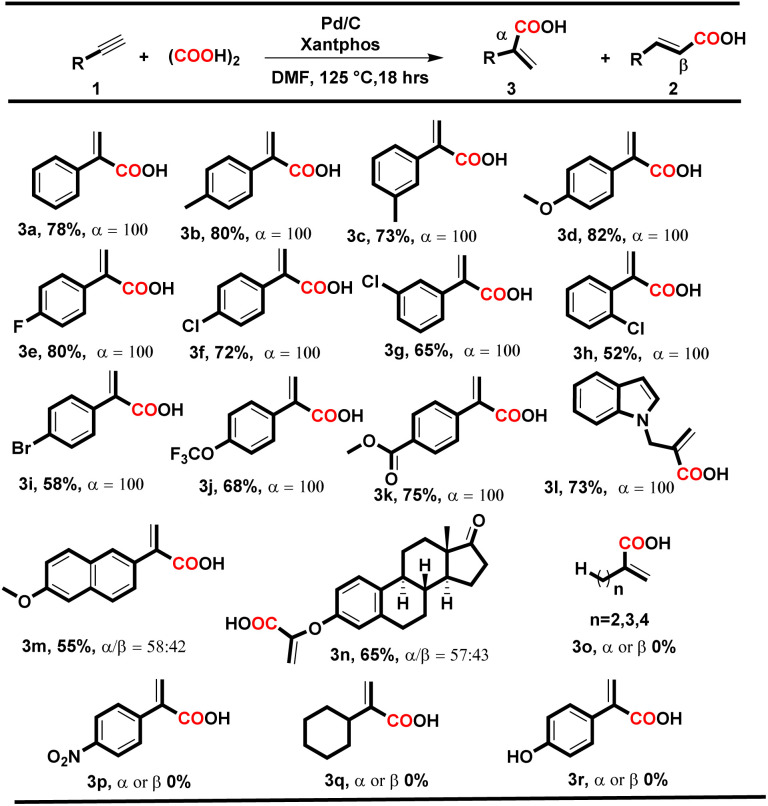

a Reaction conditions: 1 (1.0 equiv.), oxalic acid (3.0 equiv.), Pd/C (3 mol%), Xantphos (6 mol%), and 2.0 mL of DMF; heated for 18 h at 125 °C.

To our delight, under both conditions, electron-donating and electron-withdrawing substituted aryl alkynes such as methyl, methoxy, fluoro, chloro, bromo, trifluoromethyl, and polynuclear reacted successfully and delivered the corresponding acids in moderate to good yields. When the reaction was executed using condition A, the corresponding cinnamic acids were obtained in moderate to good yields (56–88%) with remarkable regioselectivity (Table 2, entries 2a–t). The selectivity for linear acids was found to be the maximum *i.e.*, 100%, for most substrates with a minimum of 50% for 2c. Additionally, when the reaction was executed using condition B, the corresponding branched acids were produced in 52–82% yields with excellent regioselectivity (Table 3, entries 3a–n). The selectivity for branched acids was found to be exceptional *i.e.* 100% in most cases (Table 3, 3a–l). However, it decreases for polynuclear compounds 3m and 3n, *i.e.* 58% and 57%, respectively (Table 3, entries 3m–n).

Notably, in both conditions, the electronic and steric effects of the substituents have a substantial impact on the yields of the products. Aryl alkynes bearing electron-withdrawing substituents delivered the corresponding acids in lower yields than electron-donating aryl alkynes (Tables 2 and 3). Remarkably, the halogen-bearing terminal alkynes exhibited 100% selectivity for the corresponding acids (2h–p and 3e–j) in good yields under both conditions. However, in the case of 2g, 2m, 2p and 3h, the yield of the desired products was lower, which might be due to steric hindrance present at the *ortho* position of the aryl alkynes. Under the optimized reaction conditions A and B, aliphatic terminal acids, 4-nitrophenyl acetylene, cyclohexyl acetylene, and 4-hydroxy phenylacetylene were not able to deliver the corresponding acids 3o–r. To check the versatility of the developed methodology, we further performed the reaction with diphenylacetylene (4a) (0.28 mmol, 1 equiv.) using conditions A and B, and the yield of the desired product 5a was found to be higher under condition A*i.e.*, 70%, than under condition B*i.e.* 58%.

During the optimization studies, it was observed that the conversion of an internal alkyne (4a) to the corresponding acid (5a) requires quite a large amount of reagents, *i.e.*, Pd/C (5 mol%), Xantphos (10 mol%) and oxalic acid (4 equiv.), and time (30 h), and delivered an optimum 88% isolated yield of 5a (see ESI†). Under these optimum reaction parameters, both symmetrical and unsymmetrical internal alkynes with electron-donating and electron-withdrawing groups were well tolerated and delivered the corresponding (*E*)-2,3-diarylacrylic acids *via syn*-addition in good to excellent yields (58–93%) (Table 4, 5a–u); no (*Z*)-isomer was found.

**Table 4:** Substrate scope of internal alkynes^a^

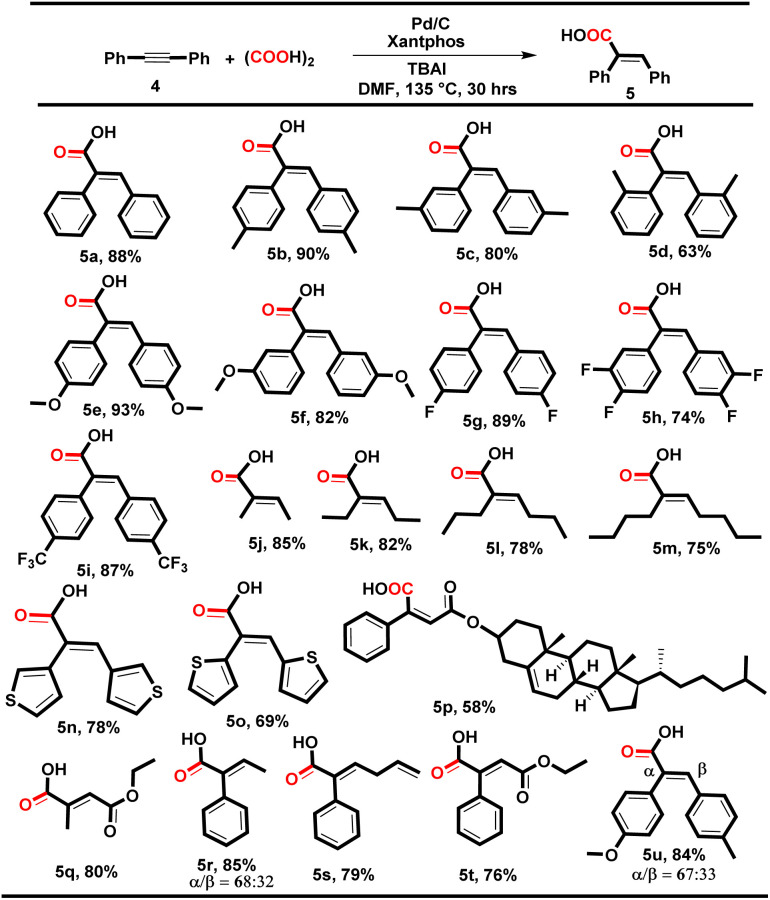

a Reaction conditions: 4 (1.0 equiv.), oxalic acid (4.0 equiv.), Pd/C (5 mol%), Xantphos (10 mol%), TBAI (0.5 equiv.), and 2.0 mL of DMF; heated for 30 h at 135 °C.

The electronic effect of the substituted diphenyl acetylene has not been found to have any prominent effect on the yield of the reaction and delivered 5a–i in 63–93% yields (Table 4, 5a–i), where the lower yield in the case of 5d might be due to the steric hindrance of the *ortho* methyl group. Subsequently, dialkyl alkynes such as 2-butyne, 3-hexyne, 4-octyne and 5-decyne also yielded the corresponding desired products 5j–m in 75–85% yields with excellent *E*-isomer selectivity, and it was found that higher alkyl substitution decreases the reaction yield. Heterocyclic aromatic internal alkynes, *i.e.* 1,2-di(thiophen-3-yl)ethyne and 1,2-di(thiophen-2-yl)ethyne, were also tolerated and yielded the desired products 5n and 5o in 78% and 69% yields, respectively. Interestingly, the alkyne-derivatized cholesterol smoothly underwent hydrocarboxylation and yielded compound 5p in 58% yield.

Despite the multifunctionality, *i.e.*, alkyne, ether, alkene and ketone in alkyne-derivatized cholesterol and estrone (3n and 5p), this methodology offers a promising late-stage functionalization strategy for alkyne-containing complex natural products and pharmaceuticals. The di-alkyl and aryl–alkyl substituted unsymmetrical internal alkynes were also well tolerated and yielded the corresponding acids 5q–t smoothly with excellent *E*-selectivity (76–85%). However, the di-aryl substituted unsymmetrical alkyne produced a mixture of the two regio-isomers (*α*/*β*) in 84% yield with 67% and 33% selectivity, respectively (5u).

Inspired by the hydrocarboxylation of alkynes, we further investigated the reaction for the carbonylative esterification of alkynes.

We performed the reaction of diphenylacetylene (0.28 mmol, 1 equiv.) with methanol using condition A, and acid 5a (65%) was formed as a major by-product along with the desired product methyl (*E*)-2,3-diphenylacrylate 7a (28%). To prevent the formation of the acid (5a), we tried the reaction using oxalic acid as an *ex situ* CO surrogate under our well-established double vial system and optimized the different reaction parameters.^9,10^ To our delight, we got an optimum yield of 80% for 7a using Pd/C (5 mol%), Xantphos (10 mol%), oxalic acid (4 equiv.), PTSA (0.5 equiv.), and methanol (0.5 mL) at 125 °C (see ESI†). With the optimum reaction conditions in hand, we successfully explored various aliphatic alcohols using diphenyl acetylene as a model substrate, yielding the desired products 7a–e in good yields (71–82%, Table 5). Additionally, various symmetrical internal alkynes containing electron-donating groups such as 4-CH_3_, 3-OCH_3_ and 4-^*t*^Bu and electron-withdrawing groups such as 4-F, 3,4-difluoro, and 4-CF_3_ were well tolerated with ethanol, yielding the corresponding carbonylative esterification products 7f–k in good to excellent yields (72–83%).

During the substrate scope investigation, it was found that the electronic effects of the diphenylacetylene derivatives had no prominent effect on the yield of the desired products. However, the 2-CH_3_ and 2-OCH_3_-substituted diphenylacetylenes failed to yield the corresponding esters, which might be due to the steric hindrance occupied at the *ortho* position. However, the heterocyclic and unsymmetrical internal alkynes were also tested with ethanol and delivered the corresponding esters 7l and 7m in 68% and 74% yields respectively with *E*-selectivity.

**Table 5:** Substrate scope for α,β-unsaturated esters^a^

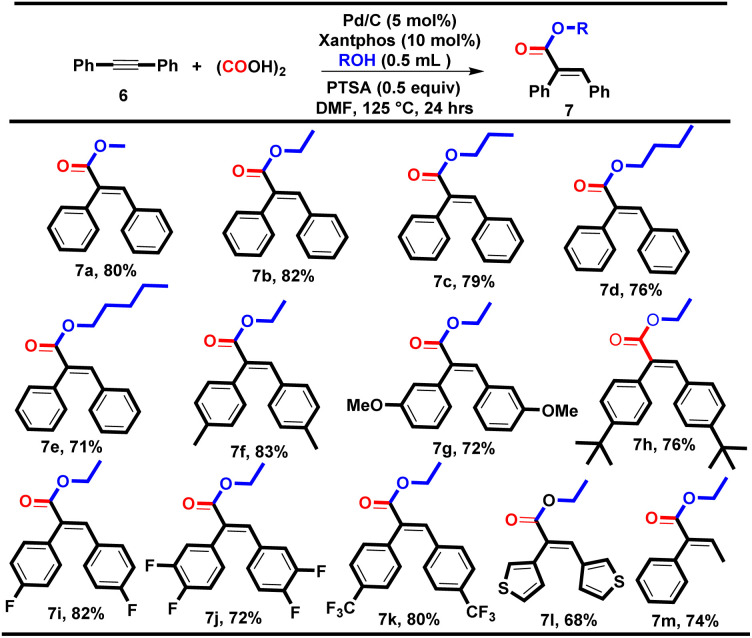

a Reaction conditions: 6 (1.0 equiv.), oxalic acid (4.0 equiv.), Pd/C (5 mol%), Xantphos (10 mol%), ROH (0.5 ml), PTSA (0.5 equiv.), and 1.5 mL of DMF; heated for 24 h at 125 °C.

**Scheme 3:**
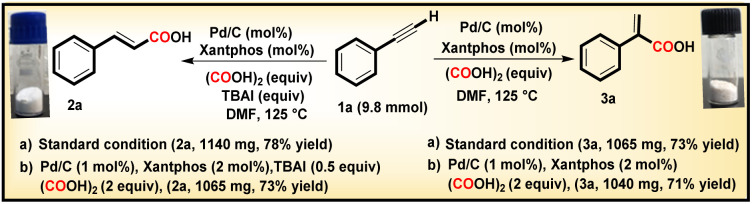
Gram-scale synthesis.

With phenylacetylene serving as the model substrate, both conditions A and B were also successfully tested for a gram scale reaction *i.e.* 9.80 mmol of 1, delivering compounds 2a and 3a in 78% and 73% yields, respectively (Scheme 3).

**Fig. 1:**
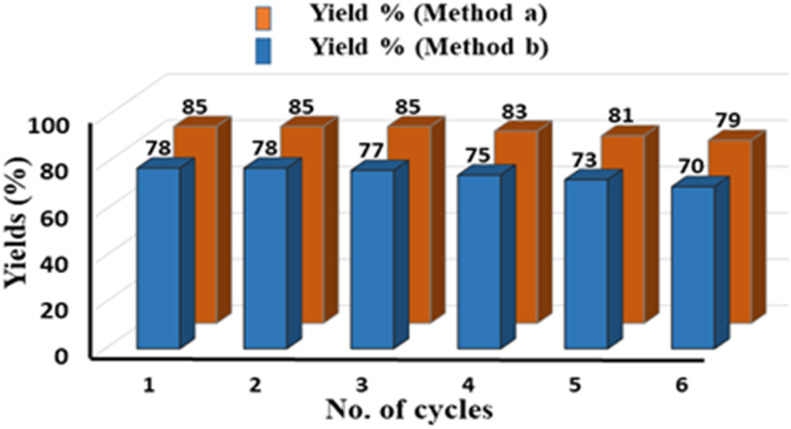
Recycling tests of Pd/C for compounds 2a and 3a.

Pleasingly, on reducing the catalyst amount to 1 mol%, with Xantphos (2 mol%), and oxalic acid (2 equiv.), both protocols A and B delivered the corresponding acids in 73% and 71% yields, respectively, without any significant decrease in yield or regioselectivity (Scheme 3). We further investigated the recyclability of Pd/C for six consecutive cycles for compounds 2a and 3a under conditions A and B respectively, and a slight decrease in their yields after the third cycle was observed (Fig. 1). The recycling experiments were performed with fresh Xantphos/TBAI, and in the absence of this Xantphos/TBAI, the recycled Pd/C did not yield the intended product. These results demonstrate the scalability, sustainability, and effectiveness of the developed procedure.

To confirm the role of Pd/C as a heterogeneous or homogeneous catalyst under the set reaction conditions, we performed a mercury poisoning test for the formation of 2a. Initially, we added 500 equiv. of mercury, *i.e.* Hg (0), into Pd/C (3 mol%) and stirred the mixture for 3 h to form an amalgam, which was further applied for the synthesis of compound 2a under the optimum reaction conditions. Only 16% of the desired product was formed. This control experiment suggests that the palladium on carbon was a heterogeneous catalyst.

However, to further identify the role of Pd/C in the catalytic cycle, we performed a hot filtration test for the formation of 2a. At the outset, the reaction mixture at 125 °C was filtered through a 0.20 μm-filter after 8 h, and the filtrate was further continued for an additional 10 h, by adding oxalic acid (6 equiv.) into the reaction mixture to maintain the internal CO pressure. However, a 15% increase in the yield of 2a was observed *i.e.* 40 to 55%. This increase in the yield indicates that there might be a possibility that Pd/C in the catalytic cycle undergoes a Pd(0) leaching process and is redeposited on the surface of charcoal at the end of the catalytic cycle. Nevertheless, these Pd/C and ligand-mediated reactions lead to a complex interplay between homogeneous and heterogeneous phases.^12^ Therefore, whether Pd/C functions as a true heterogeneous catalyst or undergoes Pd(0) leaching in the catalytic cycle is highly contentious.^11*a*^

**Scheme 4:**
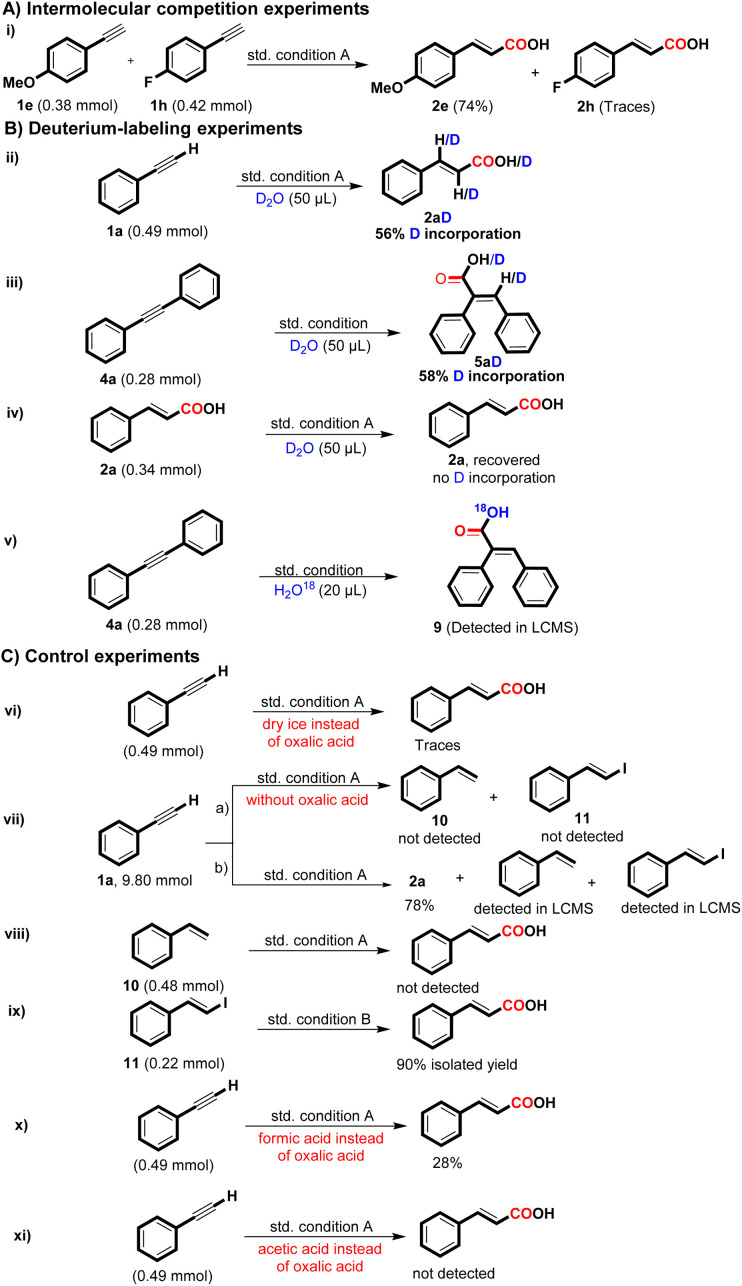
Set of control experiments.

To probe the mechanism involved in the regioselective hydrocarboxylation of terminal alkynes, a set of control experiments were conducted and the outcomes are detailed in Scheme 4. The intermolecular competition experiment of 4-ethynylanisole and 1-ethynyl-4-fluorobenzene under condition A produced 2e in 74% yield and 2h in trace amounts, indicating that the reaction favors electron-rich alkynes over electron-deficient alkynes for the desired product formation (i). Deuterium labelling experiments were also carried out for the formation of 2a and 5a with D_2_O (>99% D) under the corresponding optimized reaction conditions and yielded the targeted deuterated product 2aD with 56% D incorporation and 5aD with 58% D incorporation (ii & iii) (see ESI†). We further carried out the reaction of 2a with D_2_O under condition A and recovered 2a without any incorporation of deuterium; this experiment shows that there is no H/D exchange possibility in the formation of 2a under the set reaction conditions (iv). To further identify whether the oxalic acid acts as a CO_2_ source or not, we conducted the reaction of 4a with H_2_O^18^ under standard condition and detected the targeted compound 9 through LC-MS (v) (see ESI†). Additionally, when the oxalic acid was substituted with dry ice (solid CO_2_), the desired product was obtained in trace amounts (vi); these results indicated that the CO_2_ generated from oxalic acid might act as a CO source.^9*a*^

During the gram scale synthesis of 2a, we were able to identify some reaction intermediates. The reaction crude was analyzed through LC-MS and GC-MS techniques, and it was found that styrene (10) and *E*-(β-iodo styrene) (11) were the other by-products or intermediates formed during the reaction (vii b) (see ESI†). When eliminating oxalic acid, styrene and *E*(β-iodo styrene) were not obtained (vii a). These experiments demonstrate that oxalic acid plays a role in activating 1a for the desired product formation.

We further conducted the reaction with styrene and *E*(β-iodo styrene) for the desired product formation (viii & ix), and delightfully, the desired product 2a was formed in 90% yield under condition ix, while condition viii did not yield 2a. These experiments suggest that *E*(β-iodostyrene) may be the intermediate associated with the synthesis of 2a. There is a possibility that oxalic acid could *in situ* form formic acid, which might participate in the reaction. ^13^ Although the formation of formic acid from oxalic acid requires a high temperature, to eliminate this possibility, we carried out the reaction with formic acid (3 equiv.), and only a 28% yield of 2a was obtained, indicating that the reaction did not follow the formic acid pathway (Scheme 4, x). Furthermore, we replaced oxalic acid with acetic acid (3 equiv.) under the standard reaction conditions; however, no desired product formation was obtained, indicating that under acidic conditions, DMF doesn't act as a CO source (Scheme 4, xi).

**Scheme 5:**
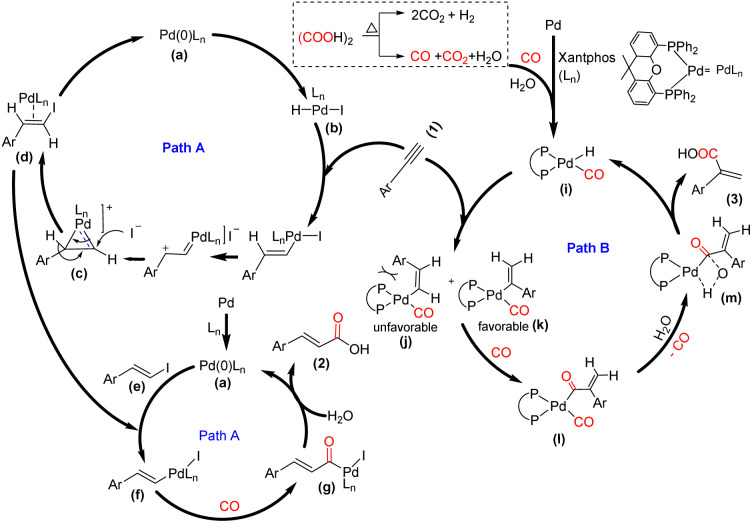
Plausible reaction mechanism.

Although the exact mechanism of the reaction is still unclear, based on the results obtained from control experiments and previous reports,^6,14^ a plausible reaction mechanism has been proposed in Scheme 5. In the cinnamic acid pathway, initially, the HI generated from oxalic acid and TBAI undergoes oxidative addition with the active palladium catalyst a, which is generated from Pd/C and Xantphos and delivers intermediate b. This intermediate b might undergo an anti-Markovnikov hydroiodination with the terminal alkyne^15^ and form intermediate d through substrate controlled face attack for (*E*)-selectivity of hydroiodination on cyclopalladium intermediate c. The intermediate d undergoes elimination to give intermediate e, *i.e. E*(β-iodo styrene), and regenerate a. The intermediate e undergoes oxidative addition with the active palladium catalyst a and delivers intermediate f, followed by CO coordination and insertion to afford intermediate g. Subsequently, the hydroxide anion acts as a nucleophile and participates in reductive elimination with intermediate g and delivers 2 (Path A). In contrast, the acrylic acid pathway follows the traditional hydrocarboxylation mechanism. Initially, the active palladium hydride complex i is formed with the aid of CO and H_2_O, generated from oxalic acid.^6,14*a*^ Subsequently, there is rapid insertion of the alkyne into the Pd–hydride complex, which forms intermediates j and k. The steric hindrance between the –Ar group and bulky Xantphos skeleton in j restricts the formation of the Pd-complexes, and consequently, there is an unfavorable generation of the corresponding linear acid 2.

However, the thermodynamically stable intermediate k, which might be responsible for the formation of 3, undergoes CO insertion and delivers the intermediate l, followed by hydrolysis, which delivers the desired product 3 through the formation of intermediate m and regenerates the complex i (Path B).

In conclusion, we have established a novel Pd/C and Xantphos combined one-pot efficient catalytic system for stereo- and regio-selective hydrocarboxylation of terminal alkynes, which selectively provides two distinct classes of acids: acrylic and cinnamic acids. Additionally, the hydrocarboxylation of internal alkynes and their carbonylative esterification with aliphatic alcohols delivered the α,β-unsaturated acids and esters, respectively. In the present protocol, under fine-tuned reaction conditions, various acrylic acids, cinnamic acids, α,β-unsaturated acids and esters were selectively obtained with good to excellent yields. The CO and H_2_O produced from oxalic acid (Brønsted acid) might act as a promoter for the formation of a Pd–H complex^6,14^ or may be formed from H_2_. The reaction process is easy to operate and requires no toxic gas. Moreover, Pd/C as an economical and easily recyclable catalyst, along with oxalic acid as a multifunctional reagent, makes this protocol more sustainable and greener, and this methodology could be suitable for academic and industrial applications.

## Data availability

The data supporting this article have been included as part of the ESI.†

## Author contributions

Pushkar Mehara: conceptualization, methodology, experiment, investigation, data curation, and writing: original draft. Poonam Sharma: experiment, data curation, writing: review and editing. Rohit Bains: experiment, data curation, writing: review and editing. Ajay K. Sharma: data curation, writing: review and editing. Pralay Das: supervision, conceptualization, roles/writing: original draft. All authors have read and agreed to the published version of the manuscript.

## Conflicts of interest

There are no conflicts to declare.

## Supplementary Material

SC-OLF-D4SC05549G-s001
