# Supplementary material for: Pd/C-catalyzed regiodivergent hydrocarboxylation and esterification of alkynes
Source: Chem Sci. 2024 Oct 9;15(44):18379–86. doi: 10.1039/d4sc05549g (PMC11499951; doi:10.1039/d4sc05549g)
Supplement: SC-015-D4SC05549G-s001 [file SC-015-D4SC05549G-s001.pdf]

This journal is © The Royal Society of Chemistry 2024

Pd/C-Catalyzed Regiodivergent Hydrocarboxylation and Esterification of Alkynes

Pushkar Mehara,[a,b] Poonam Sharma,[a,b] Rohit Bains, [a,b] Ajay Kumar Sharma,[a,b] Pralay Das*[a,b]

[a] Chemical Technology Division, CSIR-Institute of Himalayan Bioresource Technology, Palampur-176061, H.P, India.

[b] Academy of Scientific and Innovative Research (AcSIR), Ghaziabad-201002, India.

*Corresponding Author

E-mail: pdas@ihbt.res.in, pralaydas1976@gmail.com.

Table of Contents

S. No.	Content	Page No.
**A**	General Information	S2
**B**	Optimization studies of terminal alkynes to cinnamic acid (2a)	S2-S5
**C**	Optimization studies of internal alkynes to *α,β*-unsaturated acids	S6
**D**	Optimization studies of internal alkynes to *α,β*-unsaturated ester	S6-7
**E**	Control experiments data (^{1}H NMR, LC-MS, GC-MS)	S7-S10
**F**	General procedure for the synthesis and characterization of compounds **2a-t, 3a-n** and **5a-u**	S10-S32
**G**	General procedure for the synthesis and characterization of compounds **7a-m**	S33-S39
**H**	^{1}H, ^{13}C, ^{19}F NMR and ESI-MS Spectra of compounds **2a-t, 3a-n, 5a-u** and **7a-m**	S39-S143

A. General Information

High quality reagents and analytical grade solvents were purchased from Sigma Aldrich, TCI Chemicals, Merck and Sd Fine Chem. Ltd. Pd/C, Pd/Al_2O_3, $Pd(OAc)_2$ $PdCl_2$, $Pd(PPh_3)_2Cl_2$, $Pd(PPh_3)_4$, and $Pd_2(dba)_3$ were purchased from TCI chemicals and Sigma Aldrich respectively. Reactions were monitored using TLC which was performed on pre-coated silica gel plates 60 F254 (purchased from Merck) in UV light detector. Silica gel (60-120 mesh size) for column chromatography purchased from Merck was used for the purification of compounds. ^1H and ^{13}C NMR spectra were recorded using a Bruker Advance 600 spectrometer operating at Bruker Advance 600 spectrometer (600 MHz, ^1H at 600 MHz, ^{13}C at 151 MHz) and JEOL ECX500 MHz (^1H at 500 MHz, ^{13}C at 126 MHz) Spectra were recorded at 25 °C in DMSO-d_6 [residual DMSO (δ_H 2.50 and 3.33 ppm), DMSO (δ_C 39.52 ppm), $CDCl_3$ (δ_H 7.26 ppm and δ_C 77.00 ppm)] with TMS as an internal standard. Chemical shifts were recorded in δ (ppm) relative to the TMS and NMR solvent signal, coupling constants (J) are given in Hz and multiplicities of signals are reported as follows: s, singlet; d, doublet; t, triplet; m, multiplet; brs, broad singlet. Mass spectra were recorded on electrospray ionization (ESI) quadrupole time of flight (Q-TOF) mass spectrometer.

B. Optimization studies of terminal alkynes to cinnamic acid (2a)

Table S1: Effect of Pd/C loading on 2a:

Sr. No.	Pd/C (mol%)	Isolated Yield (%)
**1**	2	65
**2**	3	75
**3**	4	76
**4**	5	76

Reaction conditions: Phenyl acetylene (1 equiv), xantphos (0.06 equiv), TBAI (0.5 equiv), Oxalic acid (5 equiv), DMF (2 mL) at 125 ^oC for 24 hrs.

Table S2: Effect of Temperature in Reaction Progress

Sr. No.	Temperature (°C)	Isolated Yield (%)
**1**	115	n.r.
**2**	125	75
**3**	135	72

Reaction conditions: Phenyl acetylene (1 equiv), Pd/C (0.03 equiv), xantphos (0.06 equiv), TBAI (0.5 equiv), Oxalic acid (5 equiv), DMF (2 mL) for 24 hrs.

Table S3: Effect of Time in Reaction Progress

Sr. No.	Time (hours)	Isolated Yield (%)
**1**	9	48
**2**	12	60
**3**	15	68
**4**	18	75
**5**	24	75

Reaction conditions: Phenyl acetylene (1 equiv), Pd/C (0.03 equiv) ligand (0.06 equiv), TBAI (0.5 equiv), Oxalic acid (5 equiv), DMF (2 mL) at 125 °C.

Table S4: Effect of Oxalic acid:

Sr. No.	Oxalic acid (equiv)	Isolated yield (%)
**1**	2	72
**2**	3	85

**3**	4	80
**4**	5	75

Reaction conditions: Phenyl acetylene (1 equiv), Pd/C (0.03 equiv), xantphos (0.06 equiv), TBAI (0.5 equiv), DMF (2 mL) for 18 hrs.

Table S5: Screening of other CO sources:

Sr. No.	CO Source (3 equiv)	Isolated Yield (%)[a]
**1**	Oxalyl Chloride	Traces
**2**[b]	Formic acid	46
**3**	Formic acid	28
**4**	*N*-Formylsaccharin	34
**5**	$(HCHO)_n$	Traces
**6**	DMF	nr
**7**[c]	DMF	nr

[a] **Reaction conditions**: Phenyl acetylene (1 equiv), Pd/C (3 mol%), xantphos (0.06 equiv), TBAI (0.5 equiv), DMF (2 mL) at 125 ^{o}C for 18 hrs. [b] Acetic anhydride (3 equiv). [c] Acetic acid (3 equiv).

Table S6: Screening of ligands:

Sr. No.	Ligand	Isolated Yield (%)

**1**	Dppb	46
**2**	Dppp	38
**3**	Dppf	n.r.
**4**	1,10-Phenanthroline	Traces
**5**	Tri(*p*-tolyl)phosphine	48

Reaction conditions: Phenyl acetylene (1 equiv), Pd/C (0.03 equiv) ligand (0.06 equiv), TBAI (0.5 equiv), Oxalic acid (3 equiv), DMF (2 mL) at 125 ^{o}C for 18 hrs.

Table S7: Screening of solvent:

**Sr. No.**	**Solvent**	**Isolated Yield (%)**
**1**	DMA	80
**2**	Toluene	n.r
**3**	Propylene carbonate	n.r
**4**	PEG-400	35
**5**	Xylene	n.r
**6**	1,4-dioxane	n.r
**7**	DMSO	44

Reaction conditions: Phenyl acetylene (1 equiv), Pd/C (0.03 equiv) Xantphos (0.06 equiv), TBAI (0.5 equiv), Oxalic acid (3 equiv), Solvent (2 mL) at 125 ^{o}C for 18 hrs.

C. Optimization study of internal alkynes to *α,β*-unsaturated acid

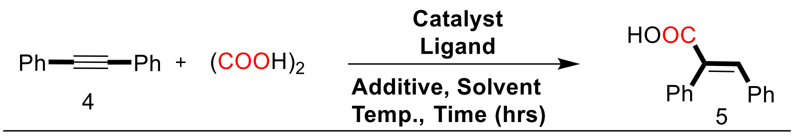

S.No.	Catalyst (mol%)	Ligand (mol%)	Additive (equiv.)	Solvent	Yield(%)[b]
1	Pd/C	Xantphos	TBAI	DMF	42
2[c]	Pd/C	Xantphos	TBAI	DMF	52
3[d]	Pd/C	Xantphos	TBAI	DMF	58
4[d,e]	Pd/C	Xantphos	TBAI	DMF	65
5[d,f]	Pd/C	Xantphos	TBAI	DMF	66
6[d,g]	Pd/C	Xantphos	TBAI	DMF	71
7[d,h]	Pd/C	Xantphos	TBAI	DMF	79
8[d,i]	Pd/C	Xantphos	TBAI	DMF	88

[a]Reaction conditions: **4** (1.0 equiv), Pd/C (3 mol%), xantphos (6 mol%), TBAI (0.5 equiv), oxalic acid (3.0 equiv), 2.0 mL of DMF; heated for 18 hrs at 125 °C; [b] Isolated yields; [c] 24 h; [d] 30 h; [e] 4 equiv oxalic acid; [f] 5 equiv oxalic acid; [g] temperature 135 °C; [h] Pd/C:xantphos (mol%) 4:8; [i] Pd/C:xantphos (mol%) 5:10

D. Optimization study of internal alkynes to *α,β*-unsaturated esters

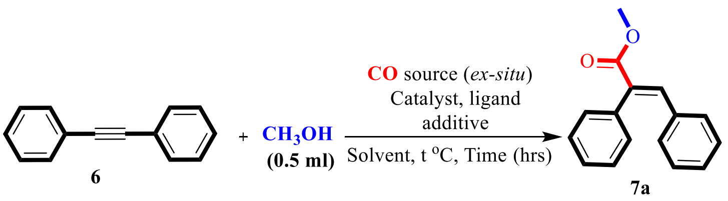

S.No.	Catalyst (mol%)	Ligand (mol%)	Additive (equiv)	Solvent	Yield(%)[b]
1	Pd/C (5)	Xantphos (10)	TBAI (0.5)	DMF	48
2[c]	Pd/C (5)	Xantphos (10)	TBAI (0.5)	DMF	28
3	Pd/C (5)	Xantphos (10)	CF_3CO_2H (0.5)	DMF	58
4	Pd/C (5)	Xantphos (10)	$(CH_3)_3CCO_2H$ (0.5)	DMF	25
5	Pd/C (5)	Xantphos (10)	CH_3COOH (0.5)	DMF	42
**6**	**Pd/C (5)**	**Xantphos (10)**	**PTSA (0.5)**	**DMF**	**80**
7	Pd/C (5)	Xantphos (10)	PTSA (1.0)	DMF	80
8	Pd/C (5)	Xantphos (10)	PTSA (0.25)	DMF	50
9	Pd/C (5)	Xantphos (10)	PTSA (0.5)	DMSO	53
10	Pd/C (5)	Xantphos (10)	PTSA (0.5)	PEG	45
11	Pd/C (5)	Xantphos (10)	PTSA (0.5)	Xylene	trace
12	Pd/C (5)	Xantphos (10)	PTSA (0.5)	DMA	76
13[d]	Pd/C (5)	Xantphos (10)	PTSA (0.5)	DMF	69
14[e]	Pd/C (5)	Xantphos (10)	PTSA (0.5)	DMF	65
15[f]	Pd/C (5)	Xantphos (10)	PTSA (0.5)	DMF	80
16[g]	Pd/C (5)	Xantphos (10)	PTSA (0.5)	DMF	76
17[h]	Pd/C (5)	Xantphos (10)	PTSA (0.5)	DMF	74
18[i]	Pd/Al_2O_3 (5)	Xantphos (10)	PTSA (0.5)	DMF	72

[a]Reaction conditions: Inner vial: **6** (0.28 mmol, 1 equiv.), 0.5 mL of methanol, Pd/C (5 mol%), Xantphos (10 mol%), Additive (equiv), 1.5 mL of DMF; Outer vial: oxalic acid (4 equiv) in 0.5 mL of DMF heated for 24 h at 125 °C; [b] Isolated yields; [c] one-pot reaction; [d] 1 mL of methanol; [e] 3 equiv oxalic acid; [f] 5 equiv oxalic acid; [g] temperature 135 °C; [h] 18 h [i] 5 wt%.

E. Control experiments data

i) ^{1}H NMR spectra of 5aD (600 MHz, CHLOROFORM-*D*) δ (*ppm*) 7.37 (d, *J* = 6.9 Hz, 3H), 7.25 – 7.22 (m, 3H), 7.16 (t, *J* = 7.3 Hz, 2H), 7.07 (d, *J* = 7.4 Hz, 2H).

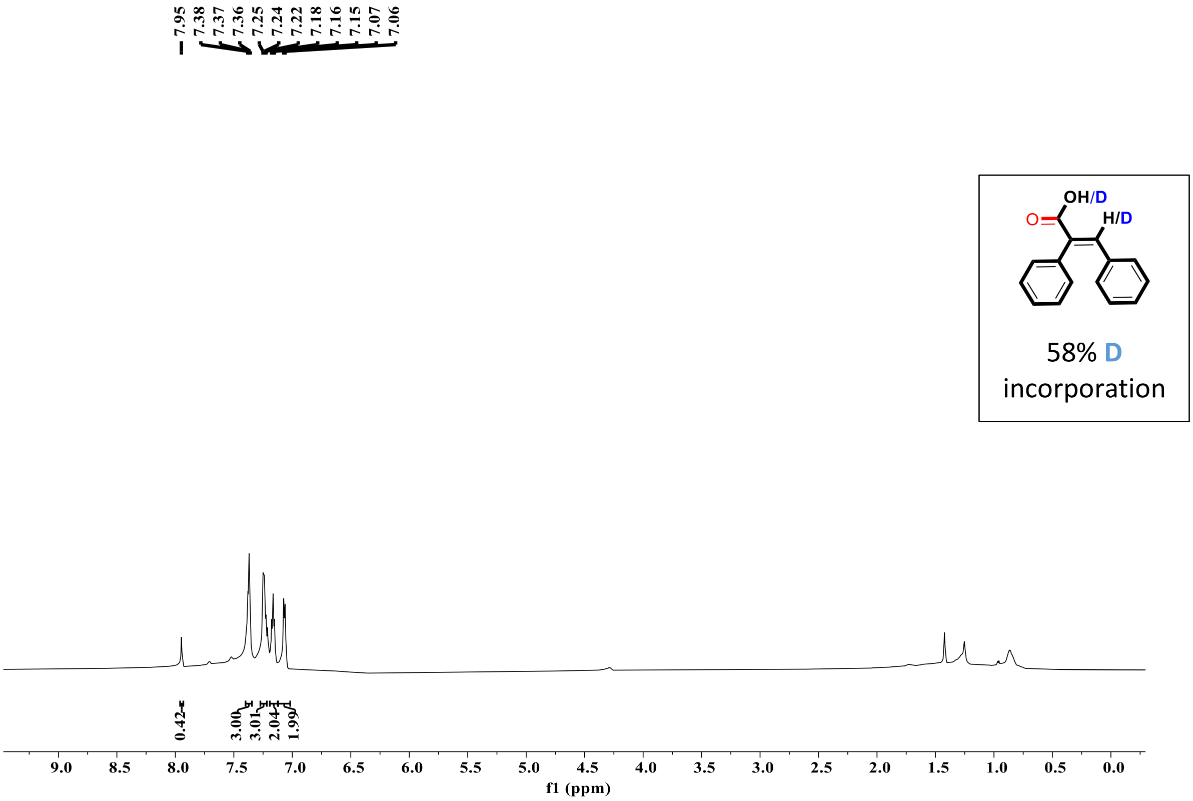

ii) LC-MS spectra of compound 5aD

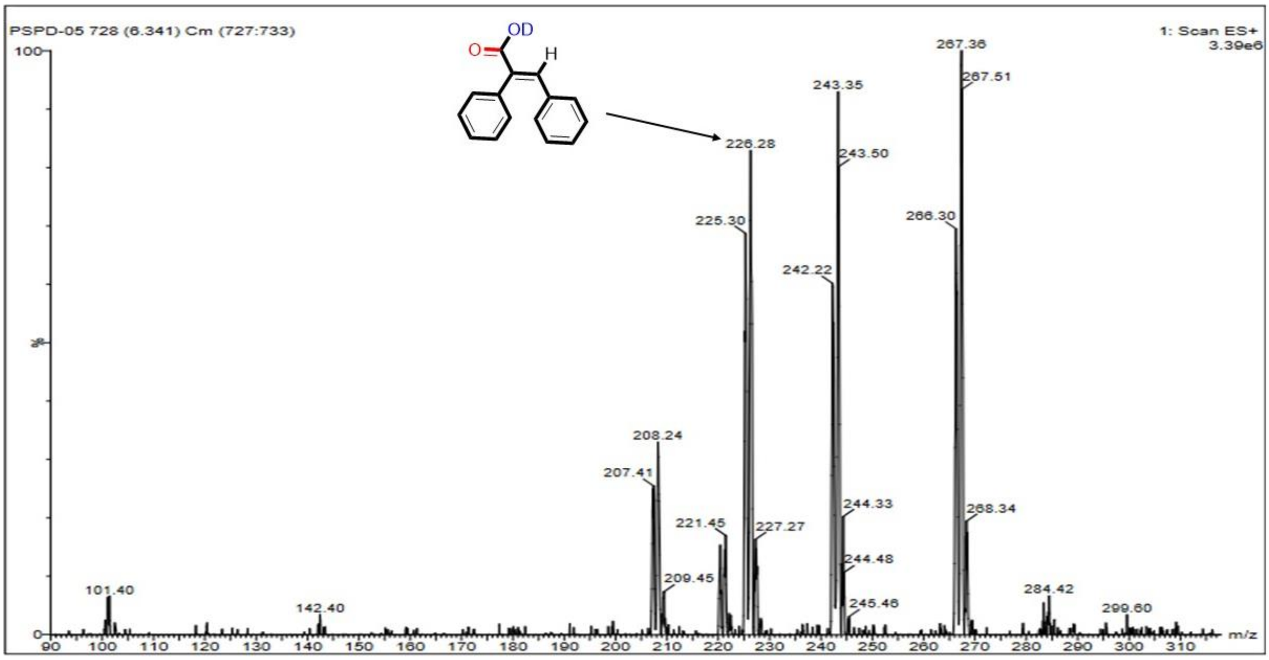

iii) ^{1}H NMR of 2aD (600 MHz, CHLOROFORM-*D*) δ 7.57 – 7.55 (m, 1H), 7.47 – 7.34 (m, 4H)

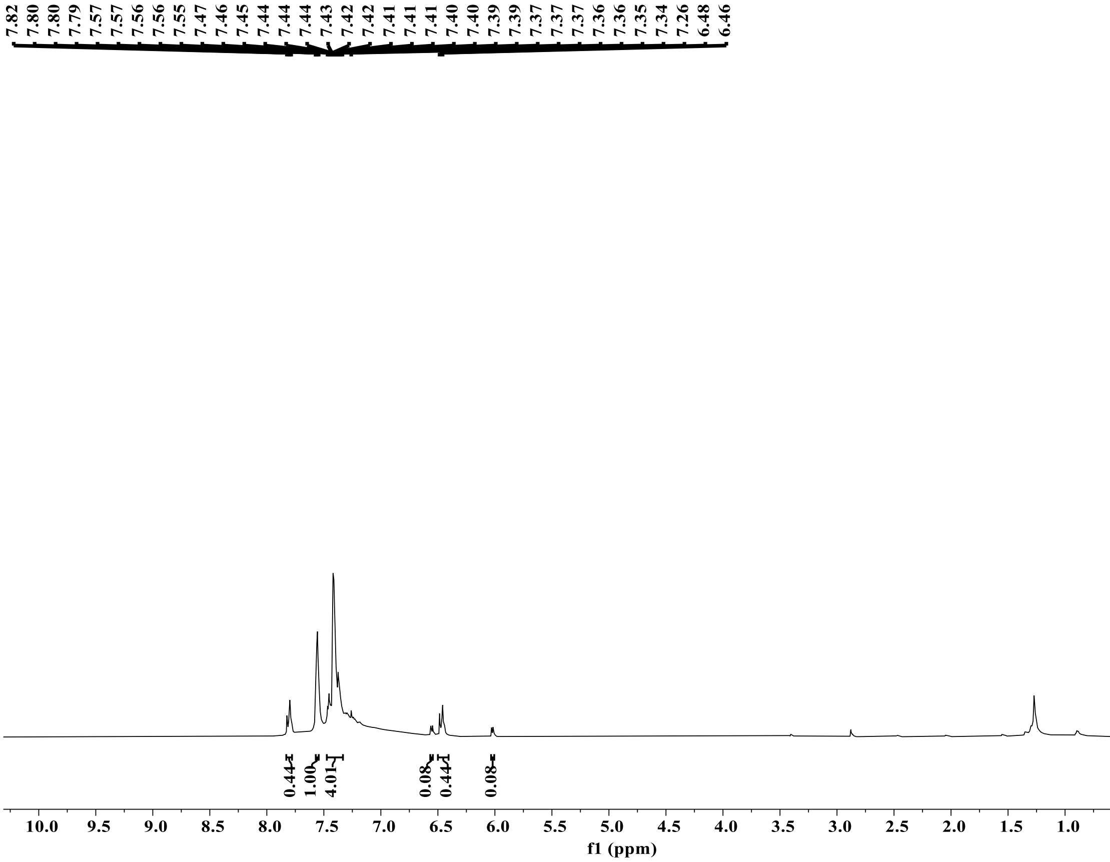

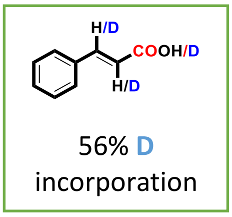

iv) LC-MS spectra of compounds 2aD and 9

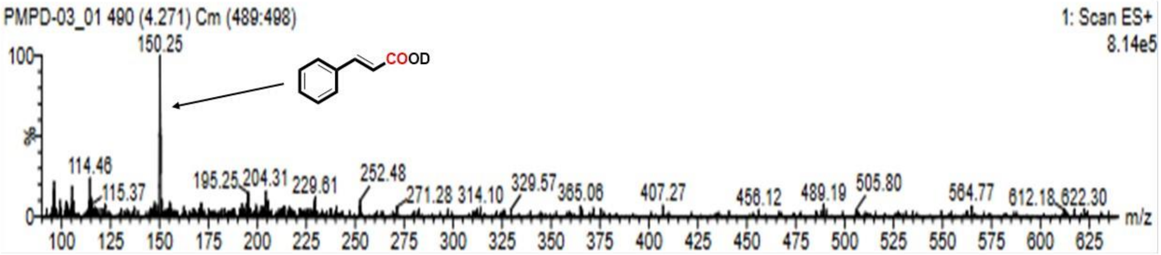

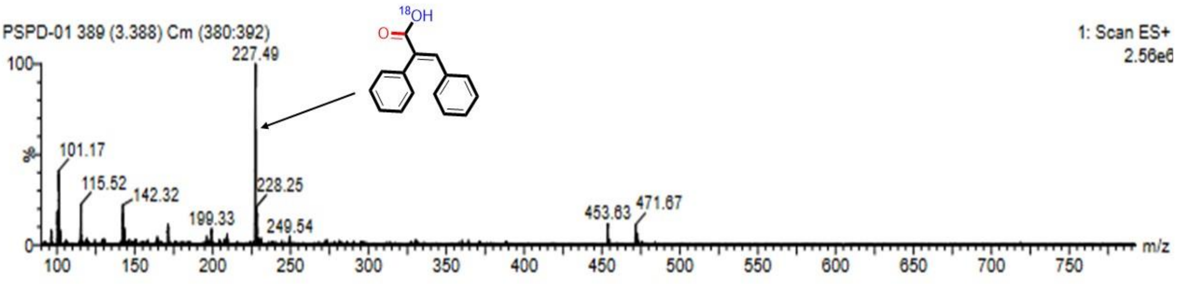

v) GC-MS and LC-MS spectra of styrene (10) and E-(*β*-iodo styrene) (11)

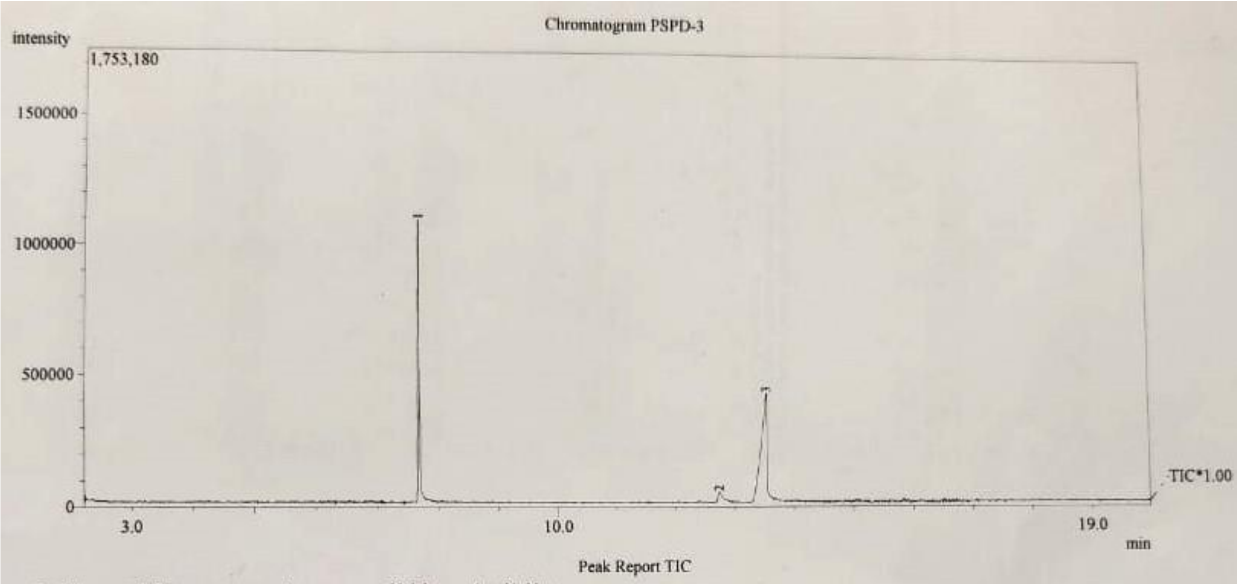

Peak Report TIC

Peak#	R.Time	Area	Height	Area%	Name
1	7.680	1937638	1081069	39.08	Styrene
2	12.737	171047	38246	3.45	Benzene, (2-iodoethenyl)-
3	13.538	2849995	415421	57.47	trans-Cinnamic acid
		4958680	1534736	100.00	

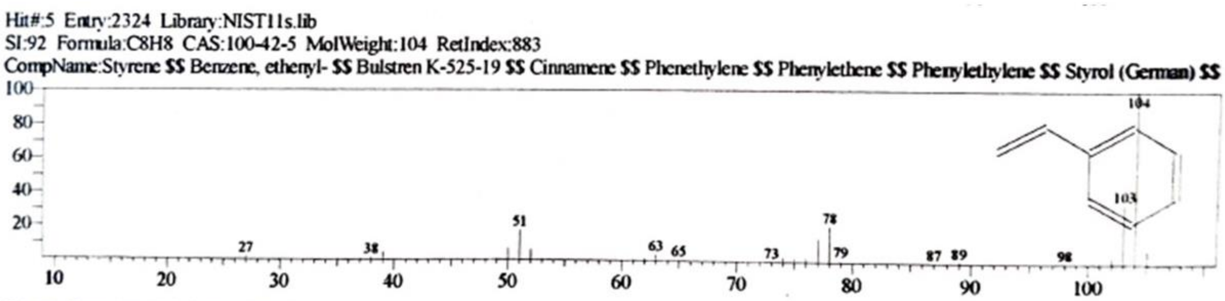

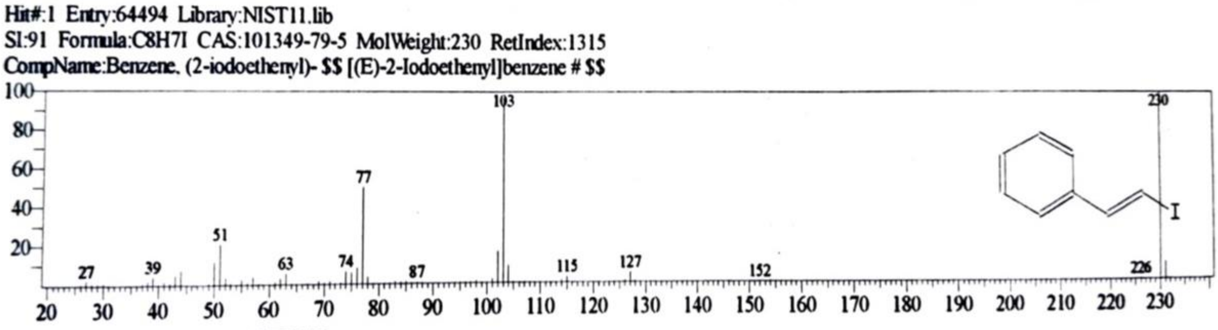

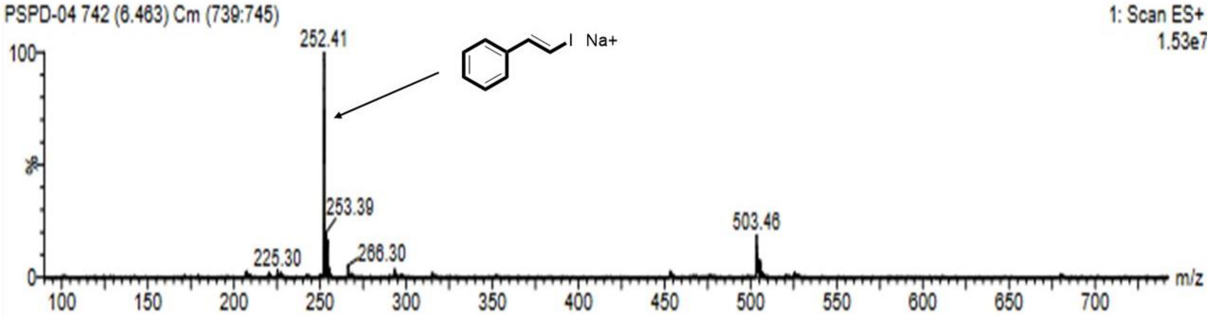

F. General procedure for the synthesis and characterization of compounds 2a-t, 3a-n and 5a-u

Cinnamic acid (2a)

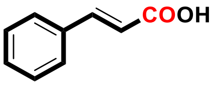

We executed the reaction using WHEATON® NextGen™ V Vial® (5 mL) and the entire system was fitted with PTFE faced black solid cap. Initially, the system was charged with phenylacetylene 1 (0.49 mmol, 53.7 µL), 5 wt% Pd/C (0.0147 mmol, 31 mg), Xantphos (0.0294 mmol, 17 mg), TBAI (0.245 mmol, 90 mg), oxalic acid (1.47 mmol, 132 mg) and DMF (2 mL). Further, the entire system was capped with the solid PTFE-faced cap and further tightened with Teflon tape to prevent the leakage of gas. The whole system was allowed to heat in preheated heating blocks at 125 ^{o}C for 18 hrs. The reaction progress was observed with the help of TLC. After the accomplishment of the reaction, the reaction mixture was placed in a separatory funnel. Further, 3 mL of water was added to the reaction mixture

and organic contents were extracted with ethyl acetate. The combined organic layer was passed over anhydrous Na_2SO_4 and concentrated under reduced pressure. The concentrated crude mixture was further purified by silica gel (mesh 100:200) column chromatography (15% EtOAc in *n*-hexane) afforded **2a** as white solid (61 mg, 85% yield, *α:β* = 16:84).

^{1}H NMR (500 MHz, DMSO-D_6) *δ* (*ppm*) 12.41 (s, 1H), 7.65-7.63 (m, 2H), 7.57 (d, *J* = 16.1 Hz, 1H), 7.37 – 7.36 (m, 3H), 6.51 (d, *J* = 15.6 Hz, 1H).^{13}C NMR (126 MHz, DMSO-D_6) *δ* (*ppm*) 167.73, 144.06, 134.33, 130.31, 129.00, 128.30, 119.32. ESI-MS [M-H]$^-$ *(m/z)* calcd. for $C_9H_7O_2^-$ is 147.0446, obsd. 147.0453.

4-Methylcinnamic acid (2b)

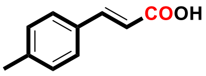

Prepared as described for **2a**; the reaction of 1-ethynyl-4-methylbenzene (0.43 mmol, 50 mg), 5 wt% Pd/C (0.0129 mmol, 27 mg), Xantphos (0.025 mmol, 17 mg), TBAI (0.215 mmol, 79 mg), oxalic acid (1.29 mmol, 116 mg) and DMF (2 mL) gave **2b** after purification silica gel (mesh 60:120) column chromatography (15% EtOAc in *n*-hexane) as white solid (60 mg, 87% yield, *α:β* = 5:95).

^{1}H NMR (600 MHz, $CDCl_3$) *δ* (*ppm*) 7.77 (d, *J* = 15.9 Hz, 1H), 7.45 (d, *J* = 8.2 Hz, 2H), 7.21 (d, *J* = 7.9 Hz, 2H), 6.41 (d, *J* = 15.9 Hz, 1H), 2.39 (s, 3H); ^{13}C NMR (151 MHz, $CDCl_3$) *δ* (*ppm*) 172.50, 147.09, 141.27, 131.33, 129.68, 128.37, 116.14, 21.49. ESI-MS [M-H]$^+$ *(m/z)* calcd. for $C_{10}H_{11}O_2^+$ is 163.0754, obsd. 163.0766

4-(*tert*-butyl)cinnamic acid (2c)

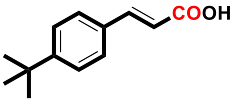

Prepared as described for **2a**; the reaction of 1-(*tert*-butyl)-4-ethynylbenzene (0.316 mmol, 50 mg), 5 wt% Pd/C (0.0094 mmol, 20 mg), Xantphos (0.0189 mmol, 11 mg), TBAI (0.158 mmol, 58 mg), oxalic acid (0.949 mmol, 86 mg) and DMF (2 mL) gave **2c** after purification silica gel (mesh 60:120) column chromatography (12% EtOAc in *n*-hexane) as white solid (50 mg, 78% yield, *α:β* = 50:50).

^{1}H NMR (600 MHz, $CDCl_3$) *δ* (*ppm*) 7.91 (s, 1H), 7.41 (d, *J* = 8.3 Hz, 2H), 7.20 – 7.18 (m, 4H), 7.03 (d, *J* = 8.5 Hz, 2H), 1.37 (s, 9H), 1.26 (s, 9H); ^{13}C NMR (151 MHz, $CDCl_3$) *δ* (*ppm*) 173.10, 153.01, 150.90, 142.11, 132.53, 131.60, 130.87, 129.28, 125.68, 125.28, 34.78, 34.67, 31.40, 31.09. ESI-MS [M-H]$^-$ *(m/z)* calcd. for $C_{13}H_{15}O_2^-$ is 203.1072, obsd. 203.1082

4-(*n*-butyl)cinnamic acid (2d)

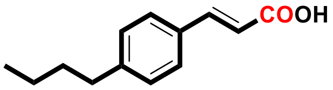

Prepared as described for **2a**; the reaction of 1-ethynyl-4-butylbenzene (0.316 mmol, 50 mg), 5 wt% Pd/C (0.0094 mmol, 20 mg), Xantphos (0.0189 mmol, 11 mg), TBAI (0.158 mmol, 58 mg), oxalic acid (0.949 mmol, 86 mg) and DMF (2 mL) gave **2d** after purification silica gel (mesh 60:120) column chromatography (12% EtOAc in *n*-hexane) as white solid (52 mg, 80% yield, $\alpha:\beta$ = 7:93).

^{1}H NMR (500 MHz, $CDCl_3$) δ (*ppm*) 7.79 (d, *J* = 15.9 Hz, 1H), 7.48 (d, *J* = 8.1 Hz, 2H), 7.22 (d, *J* = 8.2 Hz, 2H), 6.43 (d, *J* = 16.1 Hz, 1H), 2.66 – 2.63 (m, 2H), 1.65 – 1.58 (m, 2H), 1.41-1.33 (m, 2H), 0.94 (t, *J* = 7.2 Hz, 3H). ^{13}C NMR (126 MHz, $CDCl_3$) δ (*ppm*) 172.87, 147.14, 146.28, 131.47, 129.03, 128.40, 116.16, 35.56, 33.32, 22.29, 13.89. ESI-MS $[M-H]^-$ *(m/z)* calcd. for $C_{13}H_{15}O_2^-$ is 203.1072, obsd. 203.1082.

4-Methoxycinnamic acid (2e)

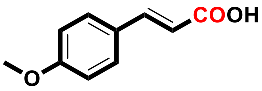

Prepared as described for **2a**, the reaction of 1-ethynyl-4-methoxybenzene (0.378 mmol, 50 mg), 5 wt% Pd/C (0.0113 mmol, 24 mg), Xantphos (0.0226 mmol, 13 mg), TBAI (0.189 mmol, 70 mg), oxalic acid (1.13 mmol, 102 mg) and DMF (2 mL) gave **2e** after purification silica gel (mesh 60:120) column chromatography (15% EtOAc in *n*-hexane) as white solid (59 mg, 88% yield).

^{1}H NMR (600 MHz, $CDCl_3$) *δ* (*ppm*) 7.74 (d, *J* = 15.9 Hz, 1H), 7.51 (d, *J* = 8.8 Hz, 2H), 6.92 (d, *J* = 8.8 Hz, 2H), 6.32 (d, *J* = 15.9 Hz, 1H), 3.85 (s, 3H); ^{13}C NMR (151 MHz, $CDCl_3$) *δ* (*ppm*) 172.25, 161.76, 146.72, 130.10, 126.82, 114.61, 114.41, 55.40. ESI-MS $[M-H]^-$ *(m/z)* calcd. for $C_{10}H_9O_3^-$ is 177.0552, obsd. 177.0556.

3-Methoxycinnamic acid (2f)

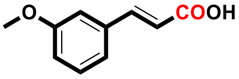

Prepared as described for **2a**; the reaction of 1-ethynyl-3-methoxybenzene (0.378 mmol, 50 mg), 5 wt% Pd/C (0.0113 mmol, 24 mg), Xantphos (0.0226 mmol, 13 mg), TBAI (0.189 mmol, 70 mg), oxalic acid (1.13 mmol, 102 mg) and DMF (2 mL) gave **2f** after purification silica gel (mesh 60:120) column chromatography (15% EtOAc in n-hexane) as white solid (49 mg, 74% yield, $\alpha:\beta$ = 14:86).

^{1}H NMR (600 MHz, DMSO-d_6) δ (*ppm*) 7.58 (d, J = 16.0 Hz, 1H), 7.32 (t, J = 7.9 Hz, 1H), 7.33 – 7.23 (m, 2H), 6.96.97 (m, 1H), 6.56 (d, J = 16.0 Hz, 1H), 3.79 (s, 3H); ^{13}C NMR (151 MHz, DMSO-d_6) δ 167.74, 159.66, 143.93, 135.72, 129.96, 120.79, 119.62, 116.27, 112.98, 55.24. ESI-MS $[M-H]^-$ *(m/z)* calcd. for $C_{10}H_9O_3^-$ is 177.0552, obsd. 177.0556.

2,4-Dimethoxycinnamic acid (2g)

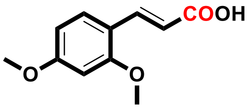

Prepared as described for 2a; the reaction of 1-ethynyl-2,4-dimethoxybenzene (0.308 mmol, 50mg), 5 wt% Pd/C (0.009 mmol, 20 mg), Xantphos (0.0226 mmol, 11 mg), TBAI (0.154 mmol, 57 mg), oxalic acid (0.925 mmol, 84 mg) and DMF (2 mL) gave **2g** after purification silica gel (mesh 60:120) column chromatography (20% EtOAc in *n*-hexane) as white solid (42 mg, 66% yield).

^{1}H NMR (500 MHz, DMSO-d_6) δ (*ppm*) 12.07 (s, 1H), 7.72 (d, J = 16.1 Hz, 1H), 7.57 (d, J = 8.6 Hz, 1H), 6.57 (d, J = 2.4 Hz, 1H), 6.53 (dd, J = 8.6, 2.4 Hz, 1H), 6.34 (d, J = 16.1 Hz, 1H), 3.82 (s, 3H), 3.77 (s, 3H); ^{13}C NMR (126 MHz, DMSO-d_6) δ (*ppm*) 168.24, 162.56, 159.33, 138.81, 129.94, 116.33, 115.44, 106.13, 98.36, 55.72, 55.46. ESI-MS $[M-H]^-$ *(m/z)* calcd. for $C_{11}H_{11}O_4^-$ is 207.0658, obsd. 207.0673

4-Fluorocinnamic acid (2h)

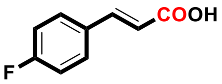

Prepared as described for **2a**; the reaction of 1-ethynyl-4-fluorobenzene (0.4166 mmol, 50 mg), 5 wt% Pd/C (0.0125 mmol, 26 mg), Xantphos (0.025 mmol, 14 mg), TBAI (0.208 mmol, 77 mg), oxalic acid (1.25 mmol, 113 mg) and DMF (2 mL) gave **2h** after purification silica gel (mesh 60:120) column chromatography (12% EtOAc in *n*-hexane) as white solid (53 mg, 77% yield).

^{1}H NMR (600 MHz, $CDCl_3$) δ (*ppm*) 7.44 – 7.42 (m, 2H), 7.06 (t, J = 8.7 Hz, 2H), 6.56 (s, 1H), 6.01 (d, J = 1.0 Hz, 1H); ^{13}C NMR (151 MHz, $CDCl_3$) δ (*ppm*) 172.07, 162.81 (d, J = 247.8 Hz), 139.54, 132.06 (d, J = 3.4 Hz), 130.26 (d, J = 8.2 Hz), 129.58, 115.09 (d, J = 21.6 Hz). ESI-MS $[M-H]^-$ *(m/z)* calcd. for $C_9H_6FO_2^-$ is 165.0352, obsd. 165.0364

3-Fluorocinnamic acid (2i)

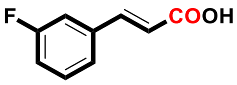

Prepared as described for **2a**; the reaction of 1-ethynyl-3-fluorobenzene (0.4166 mmol, 50 mg), 5 wt% Pd/C (0.0125 mmol, 26 mg), Xantphos (0.025 mmol, 14 mg), TBAI (0.208 mmol, 77 mg), oxalic acid (1.25 mmol, 113 mg) and DMF (2 mL) gave **2i** after purification silica gel (mesh 60:120) column chromatography (12 % EtOAc in n-hexane) as white solid (47 mg, 68% yield).

^{1}H NMR (600 MHz, DMSO-d_6) δ (*ppm*) 12.42 (s, 1H), 7.77 – 7.74 (m, 2H), 7.59 (d, J = 16.0 Hz, 1H), 7.23 (t, J = 8.8 Hz, 2H), 6.49 (d, J = 16.0 Hz, 1H); ^{13}C NMR (151 MHz, DMSO-d_6) δ (*ppm*) 167.57, 163.19 (d, J = 248.4 Hz), 142.74, 130.94 (d, J = 3.0 Hz), 130.54 (d, J = 8.4 Hz), 119.16, 119.14, 115.98, 115.84. ESI-MS [M-H]$^-$ *(m/z)* calcd. for $C_9H_6FO_2^-$ is 165.0352, obsd. 165.0360.

3,4-Difluorocinnamic acid (2j)

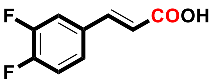

Prepared as described for **2a**; the reaction of 4-ethynyl-1,2-difluorobenzene (0.362 mmol, 50 mg), 5 wt% Pd/C (0.010 mmol, 23 mg), Xantphos (0.020 mmol, 13 mg), TBAI (0.181 mmol, 67 mg), oxalic acid (1.08 mmol, 98 mg) and DMF (2 mL) gave **2j** after purification silica gel (mesh 60:120) column chromatography (15% EtOAc in *n*-hexane) as white solid (43 mg, 65% yield).

^{1}H NMR (500 MHz, DMSO-d_6) δ (*ppm*) 12.60 (s, 1H), 7.55 (d, J = 16.0 Hz, 1H), 7.48-7.45 (m, 2H), 7.25-7.20 (m, 1H), 6.66 (d, J = 16.0 Hz, 1H); ^{13}C NMR (126 MHz, DMSO-d_6) δ (*ppm*) 167.27, 163.65 (d, J = 13.6 Hz), 161.70 (d, J = 13.2 Hz), 141.45, 138.16 (t, J = 9.9 Hz), 122.38, 111.32 (d, J = 6.0 Hz), 111.17 (d, J = 5.8 Hz), 105.21 (t, J = 26.1 Hz). ^{19}F NMR (471 MHz, DMSO-d_6) δ (*ppm*) -136.95 (d, J = 20.6 Hz), -139.00 (d, J = 22.9 Hz). ESI-MS [M-H]$^-$ *(m/z)* calcd. for $C_9H_5F_2O_2^-$ is 183.0258, obsd. 183.0269.

4-Bromocinnamic acid (2k)

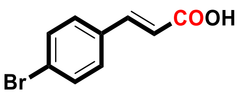

Prepared as described for **2a**; the reaction of 1-bromo-4-ethynylbenzene (0.274 mmol, 50 mg), 5 wt% Pd/C (0.008 mmol, 18 mg), Xantphos (0.0165 mmol, 10 mg), TBAI (0.137 mmol, 51 mg), oxalic acid (0.824 mmol, 74 mg) and DMF (2 mL) gave **2k** after purification silica gel (mesh 60:120) column chromatography (15% EtOAc in *n*-hexane) as pale yellow solid (42 mg, 67% yield).

^{1}H NMR (600 MHz, DMSO-d_6) δ (*ppm*) 7.61 (d, J = 8.5 Hz, 2H), 7.57 (d, J = 8.6 Hz, 2H), 7.52 (d, J = 16.0 Hz, 1H), 6.53 (d, J = 16.0 Hz, 1H); ^{13}C NMR (151 MHz, DMSO-d_6) δ (*ppm*) 167.48, 142.53, 133.60, 131.88, 130.17, 123.52, 120.29. ESI-MS $[M-H]^-$ & $[M+2-H]^-$ *(m/z)* calcd. for $C_9H_6BrO_2^-$ is 224.9551, obsd. 224.9562.

4-Chlorocinnamic acid (2l)

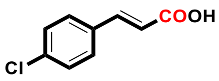

Prepared as described for **2a**; the reaction of 1-chloro-4-ethynylbenzene (0.366 mmol, 50 mg), 5 wt% Pd/C (0.011 mmol, 23 mg), Xantphos (0.0219 mmol, 13 mg), TBAI (0.183 mmol, 68 mg), oxalic acid (1.09 mmol, 98 mg) and DMF (2 mL) gave **2l** after purification silica gel (mesh 60:120) column chromatography (15% EtOAc in *n*-hexane) as pale yellow solid (43 mg, 64% yield).

^{1}H NMR (600 MHz, DMSO-d_6) δ (*ppm*) 12.49 (s, 1H), 7.73 (d, J = 8.5 Hz, 2H), 7.59 (d, J = 16.0 Hz, 1H), 7.47 (d, J = 8.5 Hz, 2H), 6.56 (d, J = 16.0 Hz, 1H); ^{13}C NMR (151 MHz, DMSO-d_6) δ (*ppm*) 167.42, 142.51, 134.71, 133.22, 129.93, 128.93, 120.09. ESI-MS $[M-H]^-$ *(m/z)* calcd. for $C_9H_6ClO_2^-$ is 181.0057, obsd. 181.0061.

2-Chlorocinnamic acid (2m)

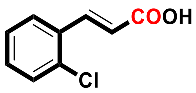

Prepared as described for **2a**; the reaction of 1-chloro-2-ethynylbenzene (0.366 mmol, 50mg), 5 wt% Pd/C (0.011 mmol, 23 mg), Xantphos (0.0219 mmol, 13 mg), TBAI (0.183 mmol, 68 mg), oxalic acid (1.09 mmol, 98 mg) and DMF (2 mL) gave **2m** after purification silica gel (mesh 60:120) column chromatography (15% EtOAc in *n*-hexane) as pale yellow solid (37 mg, 56% yield).

^{1}H NMR (500 MHz, DMSO-d_6) δ (*ppm*) 12.64 (s, 1H), 7.92- 7.90 (m, 1H), 7.87 (d, J = 16.0 Hz, 1H), 7.54 – 7.52 (m, 1H), 7.45 – 7.41 (m, 1H), 7.40-7.37 (m, 1H), 6.60 (d, J = 15.9 Hz, 1H); ^{13}C NMR (126 MHz, DMSO-d_6) δ (*ppm*) 167.18, 138.72, 133.59, 131.88, 131.68, 129.96, 128.25, 127.78, 122.32. ESI-MS $[M-H]^-$ *(m/z)* calcd. for $C_9H_6ClO_2^-$ is 181.0057, obsd. 181.0061.

4-(Trifluoromethyl)cinnamic acid (2n)

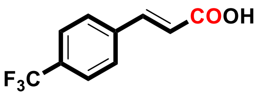

Prepared as described for **2a**; the reaction of 1-ethynyl-4-(trifluoromethyl)benzene (0.378 mmol, 50 mg), 5 wt% Pd/C (0.0113 mmol, 24 mg), Xantphos (0.0226 mmol, 13 mg), TBAI (0.189 mmol, 70 mg), oxalic acid (1.13 mmol, 102 mg) and DMF (2 mL) gave **2n** after purification silica gel (mesh 60:120) column chromatography (15% EtOAc in *n*-hexane) as white solid (47 mg, 73% yield).

^{1}H NMR (600 MHz, DMSO-*d*$_6$) *δ* (*ppm*) 12.61 (s, 1H), 7.92 (d, *J* = 8.1 Hz, 2H), 7.76 (d, *J* = 8.2 Hz, 2H), 7.66 (d, *J* = 16.1 Hz, 1H), 6.68 (d, *J* = 16.1 Hz, 1H); ^{13}C NMR (151 MHz, DMSO-*d*$_6$) *δ* (*ppm*) 167.67, 142.52, 138.78, 130.39 (d, *J* = 32.1 Hz), 129.31, δ 126.16 (d, *J* = 3.7 Hz),125.41 122.68. ^{19}F NMR (565 MHz, DMSO-*d*$_6$) *δ* (*ppm*) -61.26. ESI-MS [M-H]$^-$ *(m/z)* calcd. for $C_{10}H_6F_3O_2^-$ is 215.032, obsd. 215.0334.

3-(Trifluoromethyl)cinnamic acid (2o)

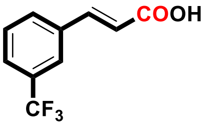

Prepared as described for **2a**; the reaction of 1-ethynyl-4-(trifluoromethyl)benzene (0.378 mmol, 50 mg), 5 wt% Pd/C (0.0113 mmol, 24 mg), Xantphos (0.0226 mmol, 13 mg), TBAI (0.189 mmol, 70 mg), oxalic acid (1.13 mmol, 102 mg) and DMF (2 mL) gave **2o** after purification silica gel (mesh 60:120) column chromatography (15% EtOAc in *n*-hexane) as white solid (42 mg, 66% yield).

^{1}H NMR (500 MHz, DMSO-*d*$_6$) *δ* (*ppm*) 12.58 (s, 1H), 8.06 (s, 1H), 8.01 (d, *J* = 8.0 Hz, 1H), 7.75 – 7.73 (m, 1H), 7.68 (d, *J* = 16.1 Hz, 1H), 7.63 (t, *J* = 7.8 Hz, 1H), 6.71 (d, *J* = 16.1 Hz, 1H); ^{13}C NMR (126 MHz, DMSO-*d*$_6$) *δ* (*ppm*) 167.43, 142.25, 135.52, 131.85, 129.98, 129.60 (d, *J* = 32.4 Hz), 126.46, 125.01 (d, *J* = 4.2 Hz), 122.98, 121.52. ^{19}F NMR (471 MHz, DMSO-*d*$_6$) *δ* (*ppm*) -62.21. ESI-MS [M-H]$^-$ *(m/z)* calcd. for $C_{10}H_6F_3O_2^-$ is 215.032, obsd. 215.0334.

2-(Trifluoromethyl)cinnamic acid (2p)

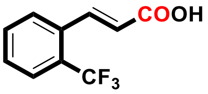

Prepared as described for **2a**, reaction of 1-ethynyl-2-(trifluoromethyl)benzene (0.378 mmol, 50 mg), 5 wt% Pd/C (0.0113 mmol, 24 mg), Xantphos (0.0226 mmol, 13 mg), TBAI (0.189 mmol, 70 mg), oxalic acid (1.13 mmol, 102 mg) and DMF (2 mL) gave **2p** after purification silica gel (mesh 60:120) column chromatography (15% EtOAc in *n*-hexane) as white solid (37 mg, 58% yield).

^{1}H NMR (500 MHz, DMSO-d_6) δ (*ppm*) 12.78 (s, 1H), 8.01 (d, J = 7.8 Hz, 1H), 7.84 – 7.83 (m, 2H), 7.70 (t, J = 7.7 Hz, 1H), 7.60 (t, J = 7.7 Hz, 1H), 6.62 (dd, J = 15.7, 1.3 Hz, 1H); ^{13}C NMR (126 MHz, DMSO-d_6) δ (*ppm*) 138.24 (d, J = 2.3 Hz), 133.09, 132.51 (d, J = 1.6 Hz), 130.27, 128.50, 127.71 – 126.54 (m), 126.14 (q, J = 5.6 Hz), 125.28, 123.93, 123.10. ESI-MS [M-H]$^-$ *(m/z)* calcd. for $C_{10}H_6F_3O_2^-$ is 215.032, obsd. 215.0334.

4-(2-Carboxy-vinyl)-benzoic acid methyl ester (2q)

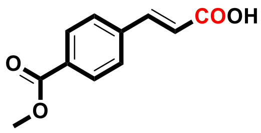

Prepared as described for **2a**; the reaction of methyl 4-ethynylbenzoate (0.287 mmol, 50 mg), 5 wt% Pd/C (0.0087 mmol, 18 mg), Xantphos (0.017 mmol, 10 mg), TBAI (0.143 mmol, 53 mg), oxalic acid (0.862 mmol, 78 mg) and DMF (2 mL) gave **2q** after purification silica gel (mesh 60:120) column chromatography (15% EtOAc in *n*-hexane) as white solid (47 mg, 79% yield).

^{1}H NMR (600 MHz, DMSO-d_6) δ (*ppm*) 7.97 – 7.95 (m, 2H), 7.83 – 7.82 (m, 2H), 7.63 (d, J = 16.0 Hz, 1H), 6.65 (d, J = 16.0 Hz, 1H), 3.86 (s, 3H); ^{13}C NMR (151 MHz, DMSO-d_6) δ (*ppm*) 167.28, 165.80, 142.47, 138.79, 130.60, 129.62, 128.45, 121.95, 52.31. ESI-MS [M-H]$^-$ *(m/z)* calcd. for $C_{11}H_9O_4^-$ is 205.0501, obsd. 205.0503

4-Cyanocinnamic acid (2r)

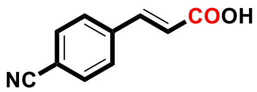

Prepared as described for **2a**; the reaction of 4-ethynyl benzonitrile (0.397 mmol, 50 mg), 5 wt% Pd/C (0.0118 mmol, 25 mg), Xantphos (0.0236 mmol, 14 mg), TBAI (0.196 mmol, 73 mg), oxalic acid (1.18 mmol, 106 mg) and DMF (2 mL) gave **2r** after purification silica gel (mesh 60:120) column chromatography (15% EtOAc in *n*-hexane) as white solid (47 mg, 69% yield, $\alpha:\beta$ = 14:86).

^{1}H NMR (600 MHz, DMSO-d_6) δ (*ppm*) 12.62 (s, 1H), 7.90 – 7.86 (m, 4H), 7.64 (d, J = 16.1 Hz, 1H), 6.71 (d, J = 16.1 Hz, 1H); ^{13}C NMR (151 MHz, DMSO-d_6) δ (*ppm*) 167.14, 141.90, 138.84, 132.72, 128.88, 122.85, 118.60, 112.11. ESI-MS [M-H]$^-$ *(m/z)* calcd. for $C_{10}H_6NO_2^-$ is 172.0399, obsd. 172.0413

(*E*)-3-(naphthalen-2-yl)acrylic acid (2s)

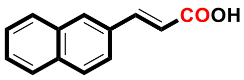

Prepared as described for **2a**; the reaction of 2-ethynyl naphthalene (0.328 mmol, 50 mg), 5 wt% Pd/C (0.009 mmol, 21 mg), Xantphos (0.019 mmol, 12 mg), TBAI (0.164 mmol, 61 mg), oxalic acid (.98 mmol, 89 mg) and DMF (2 mL) gave **2s** after purification silica gel (mesh 60:120) column chromatography (15% EtOAc in *n*-hexane) as white solid (48 mg, 74% yield).

^{1}H NMR (600 MHz, DMSO-d_6) δ (*ppm*) 8.39 (d, *J* = 15.7 Hz, 1H), 8.19 (t, *J* = 7.6 Hz, 2H), 8.02 – 7.92 (m, 2H), 7.63 – 7.55 (m, 3H), 6.60 (d, *J* = 15.7 Hz, 1H); ^{13}C NMR (151 MHz, DMSO-d_6) δ (*ppm*) 167.45, 140.08, 133.30, 131.04, 130.76, 130.33, 128.72, 127.13, 126.29, 125.72, 125.21, 122.98, 122.07. ESI-MS [M-H]$^-$ *(m/z)* calcd. for $C_{13}H_9O_2^-$ is 197.0603, obsd. 197.0611.

(*E*)-4-hydroxy-4,4-diphenylbut-2-enoic acid (2t)

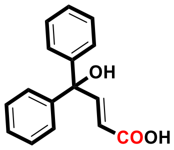

Prepared as described for **2a**; the reaction of 1,1-diphenylprop-2-yn-1-ol (0.240 mmol, 50 mg), 5 wt% Pd/C (0.007 mmol, 15 mg), Xantphos (0.014 mmol, 8 mg), TBAI (0.120 mmol, 44 mg), oxalic acid (0.72 mmol, 89 mg) and DMF (2 mL) gave **2t** after purification silica gel (mesh 60:120) column chromatography (15% EtOAc in *n*-hexane) as white solid (37 mg, 60% yield).

^{1}H NMR (500 MHz, $CDCl_3$) δ (*ppm*) 7.43 (t, *J* = 7.5 Hz, 2H), 7.38 (t, *J* = 7.2 Hz, 1H), 7.30 (d, *J* = 3.7 Hz, 6H), 7.23 (d, *J* = 7.6 Hz, 2H), 6.29 (t, *J* = 7.4 Hz, 1H), 3.26 (d, *J* = 7.5 Hz, 1H). ^{13}C NMR (126 MHz, $CDCl_3$) δ (*ppm*) 178.55, 145.22, 141.71, 138.99, 129.67, 128.40, 128.13, 127.77, 127.48, 127.40, 119.40, 35.16. ESI-MS [M-H]$^-$ *(m/z)* calcd. for $C_{16}H_{14}O_3^-$ is 253.0865, obsd. 253.0877

2-phenylacrylic acid (3a)

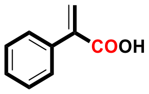

Prepared as described for **2a**; the reaction of phenylacetylene (0.49 mmol, 53.7 µL), 5 wt% Pd/C (0.0147 mmol, 31 mg), Xantphos (0.0294 mmol, 17 mg), oxalic acid (1.47 mmol, 132 mg) and DMF (2 mL) gave **3a** after purification silica gel (mesh 60:120) column chromatography (15% EtOAc in *n*-hexane) as white solid (56 mg, 78% yield).

^{1}H NMR (600 MHz, $CDCl_3$) δ (*ppm*) 7.49-7.47 (m, 2H), 7.41 – 7.39 (m, 3H), 6.59-6.58 (m, 1H), 6.06-6.05 (m, 1H); ^{13}C NMR (151 MHz, $CDCl_3$) δ (*ppm*) 172.34, 140.63, 136.06, 129.43, 128.43, 128.32, 128.11. ESI-MS [M-H]$^-$ *(m/z)* calcd. for $C_9H_7O_2^-$ is 147.0446, obsd. 147.0453.

2-(*p*-tolyl)acrylic acid (3b)

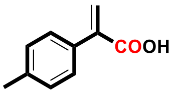

Prepared as described for **2a**; the reaction of 1-ethynyl-4-methylbenzene (0.43 mmol, 50 mg), 5 wt% Pd/C (0.0129 mmol, 27 mg), Xantphos (0.025mmol, 17 mg), oxalic acid (1.29 mmol, 116 mg) and DMF (2 mL) gave **3b** after purification silica gel (mesh 60:120) column chromatography (15% EtOAc in *n*-hexane) as white solid (56 mg, 80% yield).
^{1}H NMR (500 MHz, $CDCl_3$) δ (*ppm*) 7.35 – 7.34 (m, 2H), 7.19 (d, *J* = 7.8 Hz, 2H), 6.50 (d, *J* = 1.2 Hz, 1H), 6.00 (d, *J* = 1.2 Hz, 1H), 2.37 (s, 3H); ^{13}C NMR (126 MHz, $CDCl_3$) δ (*ppm*) 172.19, 140.43, 138.27, 133.20, 128.84, 128.69, 128.31, 21.19. ESI-MS [M-H]$^-$ *(m/z)* calcd. for $C_{10}H_9O_2^-$ is 161.0603, obsd. 161.0616

2-(*m*-tolyl)acrylic acid (3c)

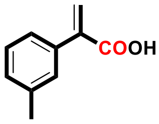

Prepared as described for **2a**; the reaction of 1-ethynyl-3-methylbenzene (0.43 mmol, 50 mg), 5 wt% Pd/C (0.0129 mmol, 27 mg), Xantphos (0.025mmol, 16.9 mg), oxalic acid (1.29 mmol, 116 mg) and DMF (2 mL) gave **3c** after purification silica gel (mesh 60:120) column chromatography (15% EtOAc in *n*-hexane) as white solid (51 mg, 73% yield).
^{1}H NMR (500 MHz, $CDCl_3$) δ (*ppm*) 7.28 – 7.18 (m, 3H), 7.17 – 7.16 (m, 1H), 6.52 (d, *J* = 1.2 Hz, 1H), 6.00 (d, *J* = 1.2 Hz, 1H), 2.37 (s, 3H); ^{13}C NMR (126 MHz, $CDCl_3$) δ (*ppm*) 172.13, 140.70, 137.75, 136.03, 129.21, 129.12, 129.11, 128.04, 125.52, 21.41. ESI-MS [M+H]$^+$ *(m/z)* calcd. for $C_{10}H_{11}O_2^+$ is 163.0754, obsd. 163.0766.

2-(4-methoxyphenyl)acrylic acid (3d)

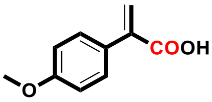

Prepared as described for **2a**; the reaction of 1-ethynyl-4-methoxybenzene (0.378 mmol, 50 mg), 5 wt% Pd/C (0.0113 mmol, 24 mg), Xantphos (0.0226 mmol, 13 mg), oxalic acid (1.13 mmol, 102 mg) and DMF (2 mL) gave **3d** after purification silica gel (mesh 60:120) column chromatography (15% EtOAc in *n*-hexane) as white solid (53 mg, 82% yield).

^{1}H NMR (500 MHz, $CDCl_3$) δ (*ppm*) 7.41 – 7.26 (m, 2H), 6.91-6.89 (m, 2H), 6.46 (d, *J* = 1.2 Hz, 1H), 5.97 (d, *J* = 1.2 Hz, 1H), 3.82 (s, 3H); ^{13}C NMR (126 MHz, $CDCl_3$) δ (*ppm*) 172.18, 159.72, 139.88, 129.67, 128.52, 127.99, 113.57, 55.30. ESI-MS [M-H]$^-$ *(m/z)* calcd. for $C_{10}H_9O_3^-$ is 177.0552, obsd. 177.0556.

2-(4-fluorophenyl)acrylic acid (3e)

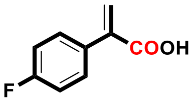

Prepared as described for **2a**; the reaction of 1-ethynyl-3-fluorobenzene (0.4166 mmol, 50 mg), 5 wt% Pd/C (0.0125 mmol, 26 mg), Xantphos (0.025 mmol, 14 mg), oxalic acid (1.25 mmol, 113 mg) and DMF (2 mL) gave **3e** after purification silica gel (mesh 60:120) column chromatography (12% EtOAc in *n*-hexane) as white solid (54 mg, 80% yield). ^{1}H NMR (600 MHz, $CDCl_3$) δ (*ppm*) 7.44 – 7.42 (m, 2H), 7.08 – 7.05 (m, 2H), 6.56 (s, 1H), 6.01 (d, *J* = 1.0 Hz, 1H); ^{13}C NMR (151 MHz, $CDCl_3$) δ (*ppm*) 172.07, 162.81 (d, *J* = 247.8 Hz), 139.54, 132.06 (d, *J* = 3.3 Hz), 130.26 (d, *J* = 8.2 Hz), 129.57, 115.09 (d, *J* = 21.6 Hz). ESI-MS [M-H]$^-$ *(m/z)* calcd. for $C_9H_6FO_2^-$ is 165.0352, obsd. 165.0364.

2-(4-chlorophenyl)acrylic acid (3f)

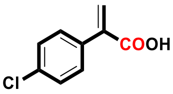

Prepared as described for **2a**; the reaction of 1-chloro-4-ethynylbenzene (0.366 mmol, 50 mg), 5 wt% Pd/C (0.0109 mmol, 23 mg), Xantphos (0.0219 mmol, 13 mg), oxalic acid (1.09 mmol, 98 mg) and DMF (2 mL) gave **3f** after purification silica gel (mesh 60:120) column chromatography (15% EtOAc in n-hexane) as white solid (48 mg, 72% yield).
^{1}H NMR (500 MHz, $CDCl_3$) δ (*ppm*) 7.40 – 7.37 (m, 2H), 7.36 – 7.33 (m, 2H), 6.58 (d, *J* = 0.9 Hz, 1H), 6.04 (d, *J* = 0.9 Hz, 1H). ^{13}C NMR (126 MHz, $CDCl_3$) δ (*ppm*) 171.70, 139.43, 134.48, 134.40, 130.02, 129.80, 128.35. ESI-MS [M-H]$^-$ *(m/z)* calcd. for $C_9H_6ClO_2^-$ is 181.0057, obsd. 181.0061.

2-(3-chlorophenyl)acrylic acid (3g)

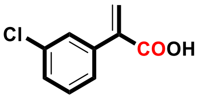

Prepared as described for **2a**; the reaction of 1-chloro-3-ethynylbenzene (0.366 mmol, 50 mg), 5 wt% Pd/C (0.0109 mmol, 23 mg), Xantphos (0.0219 mmol, 13 mg), oxalic

acid (1.09 mmol, 98 mg) and DMF (2 mL) gave **3g** after purification silica gel (mesh 60:120) column chromatography (15% EtOAc in *n*-hexane) as white solid (43 mg, 65% yield).

^{1}H NMR (500 MHz, $CDCl_3$) δ (*ppm*) 7.45 (t, *J* = 1.8 Hz, 1H), 7.37 – 7.28 (m, 3H), 6.60 (s, 1H), 6.06 (s, 1H); ^{13}C NMR (126 MHz, $CDCl_3$) δ (*ppm*) 171.36, 139.34, 137.70, 134.03, 130.56, 129.38, 128.60, 128.47, 126.68. ESI-MS $[M-H]^-$ *(m/z)* calcd. for $C_9H_6ClO_2^-$ is 181.0057, obsd. 181.0061

2-(2-chlorophenyl)acrylic acid (3h)

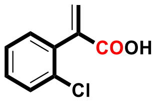

Prepared as described for **2a**; the reaction of 1-chloro-2-ethynylbenzene (0.366 mmol, 50 mg), 5 wt% Pd/C (0.0109 mmol, 23 mg), Xantphos (0.0219 mmol, 13 mg), oxalic acid (1.09 mmol, 98 mg) and DMF (2 mL) gave **3h** after purification silica gel (mesh 60:120) column chromatography (15% EtOAc in *n*-hexane) as white solid (35 mg, 52% yield).

^{1}H NMR (500 MHz, $CDCl_3$) δ (*ppm*) 7.55 – 7.36 (m, 1H), 7.33 – 7.19 (m, 3H), 6.67 (d, *J* = 1.2 Hz, 1H), 5.93 (d, *J* = 1.2 Hz, 1H); ^{13}C NMR (126 MHz, $CDCl_3$) δ (*ppm*) 170.98, 139.46, 135.81, 133.40, 131.47, 130.95, 129.69, 129.36, 126.71. ESI-MS $[M-H]^-$ *(m/z)* calcd. for $C_9H_6ClO_2^-$ is 181.0057, obsd. 181.0061.

2-(4-bromophenyl)acrylic acid (3i)

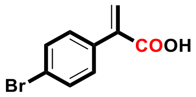

Prepared as described for **2a**; the reaction of 1-bromo-4-ethynylbenzene (0.274 mmol, 50 mg), 5 wt% Pd/C (0.008 mmol, 18 mg), Xantphos (0.0165 mmol, 10 mg), oxalic acid (0.824 mmol, 74 mg) and DMF (2 mL) gave **3i** after purification silica gel (mesh 60:120) column chromatography (15% EtOAc in *n*-hexane) as white solid (36 mg, 58% yield).

1H NMR (500 MHz, $CDCl_3$) δ (*ppm*) 7.50 (d, *J* = 8.5 Hz, 2H), 7.32 (d, *J* = 8.5 Hz, 2H), 6.57 (d, *J* = 1.0 Hz, 1H), 6.04 (d, *J* = 1.0 Hz, 1H). ^{13}C NMR (126 MHz, $CDCl_3$) δ (*ppm*) 171.33, 139.46, 134.89, 131.32, 130.10, 130.02, 122.71. ESI-MS $[M-H]^-$ & $[M+2,-H]^-$ *(m/z)* calcd. for $C_9H_6BrO_2^-$ is 224.9551, 226.9531, obsd. 224.9562.

2-(4-(trifluoromethoxy)phenyl)acrylic acid (3j)

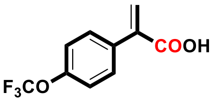

Prepared as described for **2a**; the reaction of 1-ethynyl-4-(trifluoromethoxy)benzene (0.268 mmol, 50 mg), 5 wt% Pd/C (0.008 mmol, 18 mg), Xantphos (0.0161 mmol, 9 mg), oxalic acid (0.806 mmol, 73 mg) and DMF (2 mL) gave **3j** after purification silica gel (mesh 60:120) column chromatography (15% EtOAc in *n*-hexane) as white solid (43 mg, 68% yield).

^{1}H NMR (500 MHz, $CDCl_3$) *δ* (*ppm*) 7.32 – 7.28 (m, 1H), 7.18 – 7.13 (m, 3H), 6.60 (s, 1H), 6.05 (s, 1H); ^{13}C NMR (126 MHz, $CDCl_3$) *δ* (*ppm*) 171.63, 151.47 – 148.74 (m), 138.53, 132.75 (t, *J* = 5.4 Hz), 130.65, 124.72 (t, *J* = 4.8 Hz), 117.77 (d, *J* = 17.5 Hz), 116.98 (d, *J* = 16.8 Hz). ESI-MS $[M-H]^+$ *(m/z)* calcd. for $C_{10}H_8F_3O_3^+$ is 233.0420, obsd. 233.0420.

2-(4-(methoxycarbonyl)phenyl)acrylic acid (3k)

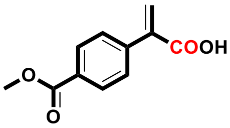

Prepared as described for **2a**; the reaction of 1 methyl 4-ethynylbenzoate (0.287 mmol, 50 mg), 5 wt% Pd/C (0.0087 mmol, 18 mg), Xantphos (0.017 mmol, 10 mg), oxalic acid (0.862 mmol, 78 mg) and DMF (2 mL) gave **3k** after purification silica gel (mesh 60:120) column chromatography (15% EtOAc in *n*-hexane) as white solid (44 mg, 75% yield).

^{1}H NMR (500 MHz, $CDCl_3$) *δ* (*ppm*) 8.05 – 8.03 (m, 2H), 7.53 – 7.51 (m, 2H), 6.64 (d, *J* = 0.9 Hz, 1H), 6.11 (d, *J* = 0.9 Hz, 1H), 3.93 (s, 3H); ^{13}C NMR (126 MHz, $CDCl_3$) *δ* (*ppm*) 171.09, 166.74, 140.48, 139.77, 130.83, 129.93, 129.42, 128.50,52.21. ESI-MS $[M-H]^-$ *(m/z)* calcd. for $C_{11}H_9O_4^-$ is 205.0501, obsd. 205.0503.

2-((1*H*-indol-1-yl)methyl)acrylic acid (3l)

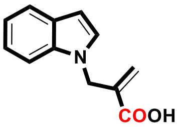

Prepared as described for **2a**; the reaction of 1-(prop-2-yn-1-yl)-1*H*-indole (0.3225 mmol, 50 mg), 5 wt% Pd/C (0.0096 mmol, 20 mg), Xantphos (0.0193 mmol, 11 mg), oxalic acid (0.967 mmol, 87 mg) and DMF (2 mL) gave **3l** after purification silica gel (mesh 60:120) column chromatography (10% EtOAc in *n*-hexane) as pale yellow solid (47 mg, 73% yield).

^{1}H NMR (500 MHz, $CDCl_3$) *δ* (*ppm*) 9.66 (s, 1H), 7.65 (d, *J* = 7.8 Hz, 1H), 7.28 – 7.20 (m, 2H), 7.14 – 7.11 (m, 2H), 6.55 (d, *J* = 3.3 Hz, 1H), 6.39 (s, 1H), 5.28 (s, 1H), 4.99 (s, 2H); ^{13}C

NMR (126 MHz, $CDCl_3$) δ (*ppm*) 170.99, 135.90, 135.76, 128.72, 128.61, 128.33, 121.84, 121.05, 119.67, 109.44, 102.02, 46.30. ESI-MS $[M-H]^-$ (m/z) calcd. for $C_{12}H_{10}NO_2^-$ is 200.0712, obsd. 200.0717.

2-(6-methoxynaphthalen-2-yl)acrylic acid (3m)

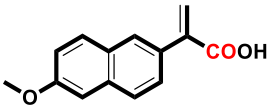

Prepared as described for **2a**, reaction of 2-ethynyl-6-methoxynaphthalene (0.274 mmol, 50 mg), 5 wt% Pd/C (0.008 mmol, 17 mg), Xantphos (0.0164 mmol, 10 mg), oxalic acid (0.824 mmol, 74 mg) and DMF (2 mL) gave **3m** after purification silica gel (mesh 60:120) column chromatography (20% EtOAc in *n*-hexane) as white solid (34 mg, 55% yield, $\alpha:\beta$ = 58:42).

^{1}H NMR (500 MHz, DMSO-d_6) δ (*ppm*) 8.09 (s, 1H), 7.91-7.90 (m, 1H), 7.86-7.78 (m, 4H), 7.76-7.69 (m, 2H), 7.55- 7.52 (m, 1H), 7.35 (m, 1H), 7.32-7.31 (m, 1H), 7.21 – 7.16 (m, 2H), 6.60 (d, *J* = 15.9 Hz, 1H), 6.26 (s, 1H), 6.05 (s, 1H), 3.89 (s, 3H), 3.88 (s, 3H). ^{13}C NMR (126 MHz, DMSO-d_6) δ (*ppm*) 168.04, 167.76, 158.38, 157.62, 144.19, 141.52, 135.32, 133.84, 131.83, 130.08, 129.73, 129.59, 129.53, 128.24, 128.00, 127.41, 126.85, 126.56, 126.27, 125.40, 124.54, 119.21, 118.84, 118.26, 106.23, 105.71, 55.32, 55.21. ESI-MS $[M-H]^-$ *(m/z)* calcd. for $C_{14}H_{11}O_3^-$ is 227.0708, obsd. 227.0721

2-(((9S,14S)-13-methyl-17-oxo-7,8,9,11,12,13,14,15,16,17-decahydro-6*H*-cyclopenta[a]phenanthren-3-yl)oxy)acrylic acid (3n)

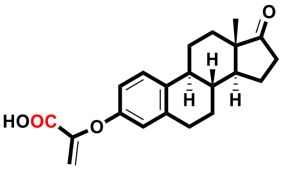

Prepared as described for **2a**; the reaction of (9S,14S)-3-(ethynyloxy)-13-methyl-6,7,8,9,11,12,13,14,15,16-decahydro-17*H*-cyclopenta[a]phenanthren-17-one (0.179 mmol, 50 mg), 5 wt% Pd/C (0.0053 mmol, 11 mg), Xantphos (0.0107 mmol, 6 mg), oxalic acid (0.537 mmol, 49 mg) and DMF (2 mL) gave **3n** after purification silica gel (mesh 60:120) column chromatography (20% EtOAc in *n*-hexane) as white solid (37 mg, 65% yield, $\alpha:\beta$ = 57:43).

^{1}H NMR (500 MHz, $CDCl_3$) δ (*ppm*) 7.34 (s, 1H), 7.29 (d, *J* = 5.3 Hz, 1H), 7.23 (d, *J* = 8.0 Hz, 1H), 7.18 (s, 1H), 6.49 (s, 1H), 5.99 (s, 1H), 2.97-2.89 (m, 6H), 2.69 (t, *J* = 7.9 Hz, 5H), 2.42 – 2.55 (m, 5H), 2.35 – 2.30 (m, 2H), 2.19 – 2.13 (m, 2H), 2.10 – 1.96 (m, 8H), 1.66 – 1.59 (m,

5H), 1.58-1.46 (m, 11H), 0.92-0.90 (m, 6H); ^{13}C NMR (126 MHz, $CDCl_3$) δ (*ppm*) 221.21, 221.00, 172.53, 172.11, 147.19, 143.20, 140.58, 140.22, 137.34, 136.46, 133.82, 131.69, 129.24, 129.12, 129.03, 128.83, 126.15, 125.98, 125.87, 125.79, 125.69, 125.36, 116.48, 50.57, 48.11, 48.05, 44.72, 44.49, 38.26, 38.13, 38.02, 35.98, 35.66, 31.65, 30.15, 29.50, 29.37, 26.56, 26.41, 25.73, 21.69, 18.22, 13.93.

(E)-2,3-diphenylacrylic acid (5a)

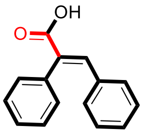

Prepared as described for **2a**; the reaction of diphenylacetylene (0.28 mmol), 5 wt% Pd/C (0.014 mmol, 29 mg), Xantphos (0.028 mmol, 16 mg), TBAI (0.14 mmol, 52 mg), oxalic acid (1.13 mmol, 101 mg) and DMF (2 mL) gave **5a** after purification silica gel (mesh 60:120) column chromatography (20% EtOAc in *n*-hexane) as white solid (55 mg, 88% yield).

^{1}H NMR (600 MHz, $CDCl_3$) δ (*ppm*) 8.01 (s, 1H), 7.43 – 7.42 (m, 3H), 7.31 – 7.26 (m, 3H), 7.23- 7.20 (m, 2H), 7.12 (d, J = 7.7 Hz, 2H); ^{13}C NMR (151 MHz, $CDCl_3$) δ (*ppm*) 173.18, 142.35, 135.30, 134.29, 131.71, 130.81, 129.75, 129.43, 128.66, 128.22, 127.99. ESI-MS [M-H]$^-$ *(m/z)* calcd. for $C_{15}H_{11}O_2^-$ is 223.0759, obsd. 223.0765

(E)-2,3-di-*p*-tolylacrylic acid (5b)

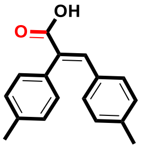

Prepared as described for **2a**; the reaction of 1,2-di-*p*-tolylethyne (0.24 mmol, 50 mg), 5 wt% Pd/C (0.012 mmol, 26 mg), Xantphos (0.024 mmol, 14 mg), TBAI (0.12 mmol, 45 mg), oxalic acid (0.968 mmol, 87 mg) and DMF (2 mL) gave **5b** after purification silica gel (mesh 60:120) column chromatography (20% EtOAc in *n*-hexane) as white solid (55 mg, 90% yield).

^{1}H NMR (600 MHz, $CDCl_3$) δ (*ppm*) 7.92 (d, J = 1.4 Hz, 1H), 7.21 (d, J = 7.7 Hz, 2H), 7.15 (d, J = 7.7 Hz, 2H), 7.01 (s, 4H), 2.41 (s, 3H), 2.30 (s, 3H); ^{13}C NMR (151 MHz, $CDCl_3$) δ (*ppm*) 173.49, 142.20, 139.78, 137.64, 132.50, 131.65, 130.86, 130.63, 129.60, 129.44, 128.99, 21.34, 21.33. ESI-MS [M-H]$^-$ *(m/z)* calcd. for $C_{17}H_{15}O_2^-$ is 251.1072, obsd. 251.1087

(E)-2,3-di-*m*-tolylacrylic acid (5c)

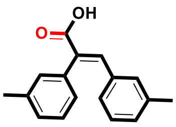 Prepared as described for **2a**; the reaction of 1,2-di-*m*-tolylethyne (0.24 mmol, 50 mg), 5 wt% Pd/C (0.012 mmol, 26 mg), Xantphos (0.024 mmol, 14 mg), TBAI (0.12 mmol, 45 mg), oxalic acid (0.968 mmol, 87 mg) and DMF (2 mL) gave **5c** after purification silica gel (mesh 60:120) column chromatography (20% EtOAc in *n*-hexane) as white solid (49 mg, 80% yield).

^{1}H NMR (600 MHz, $CDCl_3$) δ (*ppm*) 7.91 (s, 1H), 7.29 – 7.26 (m, 1H), 7.19 – 7.18 (m, 1H), 7.08 – 67.03 (m, 4H), 6.93 (d, J = 2.1 Hz, 1H), 6.86 – 6.84 (m, 1H), 2.35 (s, 3H), 2.20 (s, 3H); ^{13}C NMR (151 MHz, $CDCl_3$) δ (*ppm*) 173.14, 142.39, 138.29, 137.78, 135.35, 134.26, 131.93, 131.52, 130.27, 130.20, 128.75, 128.57, 128.07, 127.79, 126.70, 21.41, 21.22. ESI-MS $[M-H]^-$ *(m/z)* calcd. for $C_{17}H_{15}O_2^-$ is 251.1072, obsd. 251.1087

(E)-2,3-di-*o*-tolylacrylic acid (5d)

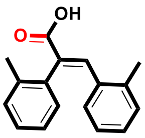Prepared as described for 2a; the reaction of 1,2-di-*o*-tolylethyne (0.24 mmol, 50 mg), 5 wt% Pd/C (0.012 mmol, 26 mg), Xantphos (0.024 mmol, 14 mg), TBAI (0.12 mmol, 45 mg), oxalic acid (0.968 mmol, 87 mg) and DMF (2 mL) gave 5d after purification silica gel (mesh 60:120) column chromatography (20% EtOAc in *n*-hexane) as white solid (38 mg, 63% yield).

^{1}H NMR (500 MHz, $CDCl_3$) δ (*ppm*) 8.25 (s, 1H), 7.25 – 7.21 (m, 2H), 7.18 – 7.10 (m, 3H), 7.07 (d, J = 7.2 Hz, 1H), 6.84 (t, J = 7.5 Hz, 1H), 6.69 (d, J = 7.9 Hz, 1H), 2.46 (s, 3H), 2.17 (s, 3H). ^{13}C NMR (126 MHz, $CDCl_3$) δ (*ppm*) 173.40, 140.80, 138.08, 136.68, 134.71, 133.38, 131.60, 130.19, 130.13, 129.99, 129.16, 128.93, 128.09, 125.95, 125.52, 20.03, 19.59.
ESI-MS $[M-H]^-$ *(m/z)* calcd. for $C_{17}H_{15}O_2^-$ is 251.1072, obsd. 251.1087

(E)-2,3-bis(4-methoxyphenyl)acrylic acid (5e)

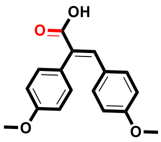 Prepared as described for **2a**; the reaction of 1,2-bis(4-methoxyphenyl)ethyne**,** 1 (0.21 mmol, 50 mg), 5 wt% Pd/C (0.011 mmol, 22 mg), Xantphos (0.021 mmol, 12 mg), TBAI (0.10 mmol, 39 mg), oxalic acid (1.05 mmol, 95 mg) and DMF (2 mL) gave **5e** after purification

silica gel (mesh 60:120) column chromatography (25% EtOAc in *n*-hexane) as white solid (56 mg, 93% yield).

^{1}H NMR (600 MHz, $CDCl_3$) δ (*ppm*) 7.88 (s, 1H), 7.18 – 7.16 (m, 2H), 7.07 – 7.05 (m, 2H), 6.94 – 6.92 (m, 2H), 6.72 – 6.70 (m, 2H), 3.85 (s, 3H), 3.77 (s, 3H); ^{13}C NMR (151 MHz, $CDCl_3$) δ (*ppm*) 173.30, 160.53, 159.22, 141.96, 132.64, 131.04, 128.70, 127.83, 127.14, 114.29, 113.76, 55.21, 55.21. ESI-MS [M-H]$^-$ *(m/z)* calcd. for $C_{17}H_{15}O_4^-$ is 283.0971, obsd. 283.0964.

(E)-2,3-bis(4-methoxyphenyl)acrylic acid (5f)

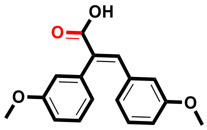

Prepared as described for **2a**; the reaction of 1,2-bis(3-methoxyphenyl)ethyne**,** (0.21 mmol, 50 mg), 5 wt% Pd/C (0.011 mmol, 22 mg), Xantphos (0.021 mmol, 12 mg), TBAI (0.10 mmol, 39 mg), oxalic acid (1.05 mmol, 95 mg) and DMF (2 mL) gave **5f** after purification silica gel (mesh 60:120) column chromatography (25% EtOAc in *n*-hexane) as white solid (49 mg, 82% yield).

^{1}H NMR (600 MHz, $CDCl_3$) δ (*ppm*) 7.91 (s, 1H), 7.32 (dd, J = 8.3, 7.5 Hz, 1H), 7.13 (t, J = 8.0 Hz, 1H), 6.93-6.91 (m, 1H), 6.87-6.85 (m, 1H), 6.81-6.77 (m, 3H), 6.59 (t, J = 2.1 Hz, 1H), 3.77 (s, 3H), 3.51 (s, 3H); ^{13}C NMR (151 MHz, $CDCl_3$) δ (*ppm*) 172.80, 159.90, 159.11, 142.34, 136.73, 135.36, 131.51, 129.85, 129.24, 124.06, 122.06, 116.60, 114.99, 114.53, 113.93, 55.25, 54.82. ESI-MS [M-H]$^-$ *(m/z)* calcd. for $C_{17}H_{15}O_4^-$ is 283.0971, obsd. 283.0964.

(E)-2,3-bis(4-fluorophenyl)acrylic acid (5g)

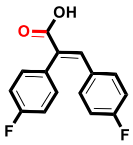

Prepared as described for **2a**; the reaction of 1,2-bis(4-fluorophenyl)ethyne**,** (0.23 mmol, 50 mg), 5 wt% Pd/C (0.011 mmol, 25 mg), Xantphos (0.023 mmol, 13 mg), TBAI (0.11 mmol, 44 mg), oxalic acid (0.93 mmol, 84 mg) and DMF (2 mL) gave **5g** after purification silica gel (mesh 60:120) column chromatography (20% EtOAc in *n*-hexane) as white solid (54 mg, 89% yield).

^{1}H NMR (600 MHz, $CDCl_3$) δ (*ppm*) 7.93 (s, 1H), 7.22 – 7.20 (m, 2H), 7.11 – 7.05 (m, 4H), 6.89 (t, J = 8.7 Hz, 2H); ^{13}C NMR (151 MHz, $CDCl_3$) δ (*ppm*) 172.98, 163.71 (d, J = 93.0 Hz), 162.05 (d, J = 88.6 Hz), 141.66, 132.75 (d, J = 8.4 Hz), 131.62 (d, J = 8.1 Hz), 130.81 (d, J =

3.5 Hz), 130.25,130.27, 115.95 (d, J = 21.5 Hz), 115.57 (d, J = 21.8 Hz). ^{19}F NMR (471 MHz, DMSO-d_6) δ (*ppm*) -112.24, -115.36. ESI-MS [M-H]$^+$ *(m/z)* calcd. for $C_{15}H_{12}F_2O_2^+$ is 261.0722, obsd. 261.0724.

(E)-2,3-bis(3,4-difluorophenyl)acrylic acid (5h)

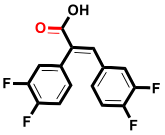

Prepared as described for **2a**; the reaction of 1,2-bis(3,4-difluorophenyl)ethyne (0.20 mmol, 50 mg), 5 wt% Pd/C (0.01 mmol, 21 mg), Xantphos (0.020 mmol, 12 mg), TBAI (0.10 mmol, 37 mg), oxalic acid (0.80 mmol, 72 mg) and DMF (2 mL) gave **5h** after purification silica gel (mesh 60:120) column chromatography (20 % EtOAc in n-hexane) as white solid (43 mg, 74% yield).

^{1}H NMR (600 MHz, $CDCl_3$) δ (*ppm*) 7.87 (s, 1H), 7.20 (dt, J = 10.1, 8.2 Hz, 1H), 7.09 – 7.02 (m, 2H), 6.96 – 6.94 (m, 1H), 6.88 – 6.83 (m, 2H); ^{13}C NMR (151 MHz, $CDCl_3$) δ (*ppm*) 172.22, 151.93 (d, J = 12.9 Hz), 151.35 (dd, J = 21.7, 12.5 Hz), 150.90 (d, J = 13.1 Hz), 150.25 (d, J = 12.9 Hz), 149.69 (dd, J = 22.7, 12.5 Hz), 149.24 (d, J = 12.8 Hz), 141.15, 130.89 (dt, J = 52.5, 5.3 Hz), 130.43, 127.57 (dd, J = 6.6, 3.4 Hz), 126.18 (dd, J = 6.2, 3.6 Hz), 119.12 (t, J = 17.5 Hz), 118.03 (d, J = 17.5 Hz), 117.58 (d, J = 17.5 Hz). ESI-MS [M-H]$^-$ *(m/z)* calcd. for $C_{15}H_7F_4O_2^-$ is 295.0382, obsd. 295.0394.

(E)-2,3-bis(4-(trifluoromethyl)phenyl)acrylic acid (5i)

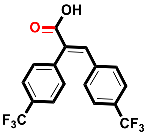

Prepared as described for **2a**; the reaction of 1,2-bis(4-(trifluoromethyl)phenyl)ethyne (0.16 mmol, 50mg), 5 wt% Pd/C (0.008 mmol, 17 mg), Xantphos (0.016 mmol, 9 mg), TBAI (0.079 mmol, 29 mg), oxalic acid (0.64 mmol, 57 mg) and DMF (2 mL) gave **5i** after purification silica gel (mesh 60:120) column chromatography (20 % EtOAc in n-hexane) as white solid (50 mg, 87% yield).

^{1}H NMR (600 MHz, $CDCl_3$) δ (*ppm*) 8.03 (s, 1H), 7.65 (d, J = 8.1 Hz, 2H), 7.47 (d, J = 8.2 Hz, 2H), 7.36 (d, J = 8.0 Hz, 2H), 7.16 (d, J = 8.2 Hz, 2H); ^{13}C NMR (151 MHz, $CDCl_3$) δ (*ppm*) 171.59, 141.66, 138.24, 137.12, 132.57, (130.71-130.29), (125.80- 125.47), 125.42, 125.39, 124.82, 124.51, 123.01, 122.71. ESI-MS [M-H]$^-$ *(m/z)* calcd. for $C_{17}H_9F_6O_2^-$ is 359.0520, obsd. 359.0528

(E)-2-methylbut-2-enoic acid (5j)

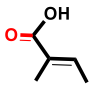

Prepared as described for **2a**; the reaction of but-2-yne (0.92 mmol, 50 mg), 10 wt% Pd/C (0.046 mmol, 24 mg), Xantphos (0.046 mmol, 27 mg), TBAI (0.46 mmol, 169 mg), oxalic acid (1.84 mmol, 166 mg) and DMF (2 mL) gave **5j** after purification silica gel (mesh 60:120) column chromatography (10% EtOAc in *n*-hexane) as semi-solid (78 mg, 85% yield). ^{1}H NMR (500 MHz, $CDCl_3$) δ (*ppm*) 7.00 (q, *J* = 6.6 Hz, 1H), 1.89 – 1.71 (m, 6H); ^{13}C NMR (126 MHz, $CDCl_3$) δ (*ppm*) 173.95, 139.96, 128.02, 14.50, 11.51.

(E)-2-ethylpent-2-enoic acid (5k)

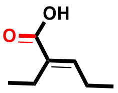

Prepared as described for **2a**; the reaction of hex-3-yne (0.609 mmol, 50 mg), 5 wt% Pd/C (0.030 mmol, 32 mg), Xantphos (0.0609 mmol, 35 mg), TBAI (0.30 mmol, 110 mg), oxalic acid (1.83 mmol, 164 mg) and DMF (2 mL) gave **5k** after purification silica gel (mesh 60:120) column chromatography (10% EtOAc in *n*-hexane) as semi-solid (64 mg, 82% yield). ^{1}H NMR (600 MHz, $CDCl_3$) δ (*ppm*) 6.87 (s, 1H), 2.30 (q, *J* = 7.5 Hz, 2H), 2.22 (p, *J* = 7.5 Hz, 2H), 1.06 (t, *J* = 7.6 Hz, 3H), 1.02 (t, *J* = 7.5 Hz, 3H); ^{13}C NMR (151 MHz, $CDCl_3$) δ (*ppm*) 173.81, 146.32, 132.84, 21.82, 19.60, 13.85, 13.19. ESI-MS $[M-H]^-$ *(m/z)* calcd. for $C_7H_{11}O_2^-$ is 127.0759, obsd. 127.0766.

(E)-2-propylhex-2-enoic acid (5l)

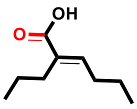

Prepared as described for **2a**; the reaction of oct-4-yne (0.453 mmol, 50 mg), 5 wt% Pd/C (0.0226 mmol, 24 mg), Xantphos (0.045 mmol, 26 mg), TBAI (0.226 mmol, 83 mg), oxalic acid (1.81 mmol, 163 mg) and DMF (2 mL) gave **5l** after purification silica gel (mesh 60:120) column chromatography (10% EtOAc in *n*-hexane) as semi-solid (55 mg, 78% yield). ^{1}H NMR (600 MHz, $CDCl_3$) δ (*ppm*) 6.91 (t, *J* = 7.5 Hz, 1H), 2.28 – 2.17 (m, 4H), 1.51 – 1.41 (m, 4H), 0.96-0.90 (m, 6H); ^{13}C NMR (151 MHz, $CDCl_3$) δ (*ppm*) 173.72, 145.49, 131.74, 30.76, 28.37, 22.42, 21.98, 13.95, 13.90.

(E)-2-butylhept-2-enoic acid (5m)

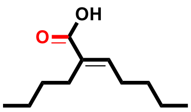 Prepared as described for **2a**, reaction of dec-5-yne (0.362 mmol, 50 mg), 5 wt% Pd/C (0.018 mmol, 19 mg), Xantphos (0.036 mmol, 21 mg), TBAI (0.181 mmol, 67 mg), oxalic acid (1.44 mmol, 130 mg) and DMF (2 mL) gave **5m** after purification silica gel (mesh 60:120) column chromatography (10% EtOAc in *n*-hexane) as semi-solid (50 mg, 75% yield). ^{1}H NMR (600 MHz, $CDCl_3$) δ (*ppm*) 6.90 (t, J = 7.5 Hz, 1H), 2.30 – 2.27 (m, 2H), 2.20 (q, J = 7.4 Hz, 2H), 1.46 – 1.31 (m, 8H), 0.93-0.90 (m, 6H); ^{13}C NMR (151 MHz, $CDCl_3$) δ (*ppm*) 173.85, 145.44, 131.87, 31.47, 30.85, 28.43, 26.18, 22.67, 22.46, 13.91, 13.85. ESI-MS [M-H]$^-$ *(m/z)* calcd. for $C_{11}H_{19}O_2^-$ is 183.0258, obsd. 183.0265.

(E)-2,3-di(thiophen-3-yl)acrylic acid (5n)

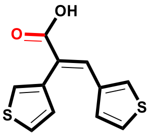 Prepared as described for **2a**; the reaction of 1,2-di(thiophen-3-yl)ethyne (0.263 mmol, 50 mg), 5wt% Pd/C (0.0131 mmol, 28 mg), Xantphos (0.026 mmol, 15 mg), TBAI (0.131 mmol, 48 mg), oxalic acid (1.31 mmol, 118 mg) and DMF (2 mL) gave **5n** after purification silica gel (mesh 60:120) column chromatography (25% EtOAc in *n*-hexane) as pale yellow solid (48 mg, 78% yield).

^{1}H NMR (500 MHz, DMSO-d_6) δ (*ppm*) 7.81 (d, J = 0.7 Hz, 1H), 7.59-7.58 (m, 1H), 7.57-7.56 (m, 1H), 7.38-7.37 (m, 1H), 7.35-7.34 (m, 1H), 6.96-6.95 (m, 1H), 6.44-6.43 (m, 1H); ^{13}C NMR (126 MHz, DMSO-d_6) δ (*ppm*) 168.35, 136.79, 136.22, 134.25, 130.43, 129.03, 127.16, 126.66, 126.20, 126.18, 124.58. ESI-MS [M-H]$^+$ *(m/z)* calcd. for $C_{11}H_7S_2O_2^-$ is 234.9888, obsd. 234.9888.

(E)-2,3-di(thiophen-2-yl)acrylic acid (5o)

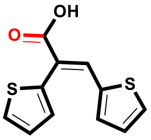 Prepared as described for **2a**; the reaction of 1,2-di(thiophen-3-yl)ethyne (0.263 mmol, 50 mg), 5 wt% Pd/C (0.0131 mmol, 28 mg), Xantphos (0.026 mmol, 15 mg), TBAI (0.131 mmol, 48 mg), oxalic acid (1.31 mmol, 118 mg) and DMF (2 mL) gave **5o** after purification silica gel (mesh 60:120) column chromatography (25% EtOAc in *n*-hexane) as pale yellow solid (43 mg, 69% yield).

^{1}H NMR (500 MHz, DMSO-d_6) δ (*ppm*) 8.12 (s, 1H), 7.72 (d, *J* = 5.2 Hz, 1H), 7.62 (d, *J* = 4.9 Hz, 1H), 7.48 (d, *J* = 3.5 Hz, 1H), 7.15 (m, 1H), 7.06 – 7.04 (m, 1H), 7.00 (d, *J* = 3.4 Hz, 1H); ^{13}C NMR (126 MHz, DMSO-d_6) δ (*ppm*) 167.57, 137.70, 136.33, 135.33, 135.21, 132.53, 128.79, 128.43, 127.67, 126.96, 122.04. ESI-MS $[M-H]^+$ *(m/z)* calcd. for $C_{11}H_7S_2O_2^-$ is 234.9888, obsd. 234.9882.

(Z)-4-(((3S,9S,14S,17R)-10,13-dimethyl-17-(6-methylheptan-2-yl)-2,3,4,7,8,9,10,11,12,13,14,15,16,17-tetradecahydro-1*H*-cyclopenta[a]phenanthren-3-yl)oxy)-4-oxo-2-phenylbut-2-enoic acid (5p)

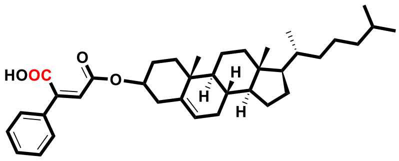

Prepared as described for **2a**; reaction of (3S,9S,14S,17R)-10,13-dimethyl-17-(6-methylheptan-2-yl)-2,3,4,7,8,9,10,11,12,13,14,15,16,17-tetradecahydro-1*H*-cyclopenta[a]phenanthren-3-yl-3-phenylpropiolate (0.0967 mmol, 50 mg), 5 wt% Pd/C (0.0029 mmol, 7 mg), Xantphos (0.0058 mmol, 4 mg), TBAI (0.049 mmol, 18 mg), oxalic acid (0.77 mmol, 70 mg) and DMF (2 mL) gave **5p** after purification silica gel (mesh 60:120) column chromatography (25% EtOAc in n-hexane) as pale yellow solid (32 mg, 58% yield).

^{1}H NMR (500 MHz, $CDCl_3$) δ (*ppm*) 7.37 (d, *J* = 4.1 Hz, 3H), 7.25 – 7.24 (m, 2H), 7.12 (s, 1H), 5.30 – 5.29 (m, 1H), 4.55-4.48 (m, 1H), 2.12 – 1.90 (m, 3H), 1.86 – 1.75 (m, 2H), 1.23-1.56 (m, 13H), 1.17 – 0.96 (m, 10H), 0.92-0.89 (m, 6H), 0.87 – 0.85 (m, 6H), 0.66 (s, 3H); ^{13}C NMR (126 MHz, $CDCl_3$) δ (*ppm*) 171.58, 164.56, 142.07, 139.20, 133.44, 131.86, 128.91, 128.64, 127.82, 122.81, 74.97, 56.58, 56.06, 49.87, 42.23, 39.63, 39.47, 37.46, 36.74, 36.45, 36.13, 35.75, 31.80, 31.74, 28.18, 27.97, 27.18, 24.23, 23.79, 22.80, 22.54, 20.94, 19.15, 18.67, 11.80. ESI-MS $[M-H]^-$ *(m/z)* calcd. for $C_{37}H_{51}O_4^-$ is 559.3788, obsd. 559.3805.

(E)-4-ethoxy-2-methyl-4-oxobut-2-enoic acid (5q)

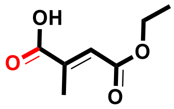

Prepared as described for **2a**; the reaction of ethyl but-2-ynoate (0.446 mmol, 50 mg), 5 wt% Pd/C (0.0223 mmol, 47 mg), Xantphos (0.045 mmol, 26 mg), TBAI (0.223 mmol, 82 mg), oxalic acid (1.78 mmol, 160 mg) and DMF (2 mL) gave **5q** after purification silica gel

(mesh 60:120) column chromatography (5% EtOAc in *n*-hexane) as white solid (56 mg, 80% yield).

^{1}H NMR (500 MHz, $CDCl_3$) δ (*ppm*) 11.91 (s, 1H), 6.87 (d, *J* = 1.6 Hz, 1H), 4.22 (q, *J* = 7.1 Hz, 2H), 2.26 (d, *J* = 1.6 Hz, 3H), 1.29 (t, *J* = 7.1 Hz, 3H); ^{13}C NMR (126 MHz, $CDCl_3$) δ (*ppm*) 172.65, 165.65, 142.40, 128.73, 60.84, 14.05, 13.79. ESI-MS $[M-H]^-$ *(m/z)* calcd. for $C_7H_9O_4^-$ is 157.0501, obsd. 157.0511.

(E)-2-phenylbut-2-enoic acid (5r)

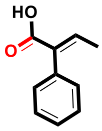

Prepared as described for **2a**; the reaction of ethyl prop-1-yn-1-ylbenzene (0.431 mmol, 50 mg), 5 wt% Pd/C (0.0215 mmol, 44 mg), Xantphos (0.0431 mmol, 25 mg), TBAI (0.215 mmol, 79 mg), oxalic acid (1.72 mmol, 155 mg) and DMF (2 mL) gave **5r** after purification silica gel (mesh 60:120) column chromatography (10% EtOAc in *n*-hexane) as white solid (59 mg, 85% yield, $\alpha:\beta$ = 68:32).

^{1}H NMR (600 MHz, $CDCl_3$) δ (*ppm*) 7.87 (t, *J* = 1.7 Hz, 1H), 7.47 – 7.41 (m, 5H), 7.38 – 7.36 (m, 2H), 7.26 – 7.23 (m, 1H), 2.17 (d, *J* = 1.5 Hz, 3H), 1.81 (d, *J* = 7.2 Hz, 1H); ^{13}C NMR (151 MHz, $CDCl_3$) δ (*ppm*) 174.50, 172.74, 142.81, 141.10, 135.54, 134.39, 134.20, 129.81, 129.79, 128.67, 128.40, 128.04, 127.60, 127.54, 15.73, 13.65.

(E)-2-phenylhexa-2,5-dienoic acid (5s)

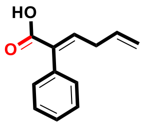

Prepared as described for **2a**; the reaction of pent-4-en-1-yn-1-ylbenzene (0.352 mmol, 50 mg), 5 wt% Pd/C (0.0176 mmol, 37 mg), Xantphos (0.0352 mmol, 20 mg), TBAI (0.176 mmol, 65 mg), oxalic acid (1.4 mmol, 126 mg) and DMF (2 mL) gave **5s** after purification silica gel (mesh 60:120) column chromatography (15% EtOAc in *n*-hexane) as white solid (52 mg, 79% yield).

^{1}H NMR (500 MHz, $CDCl_3$) δ (*ppm*) 7.62 (s, 1H), 7.48 (d, *J* = 7.5 Hz, 2H), 7.42 – 7.19 (m, 5H), 6.40 – 6.35 (m, 1H), 1.87 (d, *J* = 6.2 Hz, 2H).^{13}C NMR (126 MHz, $CDCl_3$) δ (*ppm*) 173.66, 139.26, 135.31, 133.54, 130.17, 128.98, 128.78, 128.33, 123.40, 19.26. ESI-MS $[M-H]^-$ *(m/z)* calcd. for $C_{10}H_9O_2^-$ is 161.0603, obsd. 161.0616.

(E)-4-ethoxy-4-oxo-2-phenylbut-2-enoic acid (5t)

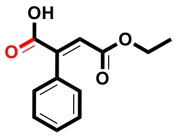

Prepared as described for **2a**; the reaction of pent-4-en-1-yn-1-ylbenzene (0.287 mmol, 50 mg), 5 wt% Pd/C (0.0143 mmol, 30 mg), Xantphos (0.0287 mmol, 16 mg), TBAI (0.143 mmol, 52 mg), oxalic acid (1.149 mmol, 103 mg) and DMF (2 mL) gave **5t** after purification silica gel (mesh 60:120) column chromatography (15% EtOAc in *n*-hexane) as white solid (51 mg, 76% yield).

^{1}H NMR (600 MHz, $CDCl_3$) δ (*ppm*) 7.38-7.37 (m, 3H), 7.26 – 7.24 (m, 2H), 7.12 (s, 1H), 4.05 (q, J = 7.1 Hz, 2H), 1.06 (t, J = 7.1 Hz, 3H); ^{13}C NMR (151 MHz, $CDCl_3$) δ (*ppm*) 171.04, 165.12, 142.68, 133.37, 131.08, 128.86, 128.69, 127.82, 61.03, 14.09. ESI-MS $[M-H]^-$ *(m/z)* calcd. for $C_{12}H_{11}O_4^-$ is 219.0658, obsd. 219.0673

(E)-2-(4-methoxyphenyl)-3-(*p*-tolyl)acrylic acid (5u)

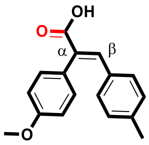

Prepared as described for **2a**; the reaction of 1-methoxy-4-(*p*-tolylethynyl)benzene (0.223 mmol, 50mg), 5wt% Pd/C (0.011 mmol, 23 mg), Xantphos (0.0223 mmol, 13 mg), TBAI (0.115 mmol, 42 mg), oxalic acid (0.892 mmol, 80 mg) and DMF (2 mL) gave **5u** after purification silica gel (mesh 60:120) column chromatography (15 % EtOAc in n-hexane) as white solid (50 mg, 84% yield, α:β = 67:33).

^{1}H NMR (600 MHz, $CDCl_3$) δ (*ppm*) 7.21 (d, J = 7.7 Hz, 1H), 7.17 – 7.14 (m, 3H), 7.06– 7.05 (m, 1H), 7.00 (s, 4H), 6.71 – 6.70 (m, 1H), 3.84 (s, 3H), 2.29 (s, 3H); ^{13}C NMR (151 MHz, $CDCl_3$) δ (*ppm*) 173.64, 160.49, 159.19, 142.02, 141.77, 139.64, 137.53, 132.74, 132.65, 131.78, 131.03, 130.80, 130.47, 129.63, 129.51, 129.26, 128.98, 127.75, 127.13, 114.12, 113.70, 55.18, 55.16, 21.35, 21.33. ESI-MS $[M-H]^-$ *(m/z)* calcd. for $C_{17}H_{15}O_3^-$ is 269.1172, obsd. 269.1177.

G. General procedure for the synthesis and characterization of compounds 7a-m methyl (E)-2,3-diphenylacrylate (7a)

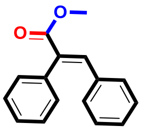 We executed the reaction using Double-Vial (DV) system comprised of an inner vial of 2 mL and an outer vial of 5 mL and the entire system was fitted with PTFE faced black solid cap. Initially, the inner vial of the DV system was charged with diphenylacetylene (0.223 mmol, 50 mg), 5 wt% Pd/C (0.011 mmol, 23 mg), Xantphos (0.0223 mmol, 13 mg), PTSA (0.14 mmol, 27 mg), methanol (0.5 mL) and DMF (1.5 mL), while the outer vial contained oxalic acid (0.892 mmol, 80 mg) in 0.5 mL of DMF. Thereafter, the inner vial having contents was positioned carefully inside the outer vial (5 mL) containing oxalic acid/DMF (0.5 mL). Further, the entire system was capped with the solid PTFE faced cap and further tightened with Teflon tape to prevent the leakage of gas. The whole system was allowed to subjected in pre-heated blocks at 125 ^{o}C for 24 hrs. The reaction progress was observed with the help of TLC. After the accomplishment of the reaction, the inner vial containing contents was removed and the contents of the inner vial were transferred to a separatory funnel. Further, 3 mL of water was added in the reaction mixture and organic contents were extracted with ethyl acetate. The combined organic layer was passed over anhydrous Na_2SO_4 and concentrated under reduced pressure. The concentrated crude mixture was further purified by silica gel (mesh 60:120) column chromatography (5% EtOAc in *n*-hexane) afforded **7a** as white solid (54 mg, 80% yield).

^{1}H NMR (400 MHz, $CDCl_3$) δ (*ppm*) 7.98 – 7.90 (m, 1H), 7.42-7.40 (m, 3H), 7.33 – 7.21 (m, 5H), 7.19 – 7.07 (m, 2H), 3.83 (s, 3H); ^{13}C NMR (101 MHz, $CDCl_3$) δ (*ppm*) 168.26, 140.47, 135.83, 134.63, 132.43, 130.58 (d, *J* = 5.5 Hz), 129.74 (d, *J* = 6.6 Hz), 129.03 (d, *J* = 6.2 Hz), 128.62 (d, *J* = 5.7 Hz), 128.25 – 128.04 (m), 127.82 (d, *J* = 4.8 Hz), 52.31. ESI-MS $[M-H]^+$ *(m/z)* calcd. for $C_{16}H_{15}O_2^+$ is 239.1067, obsd. 239.1074

ethyl (E)-2,3-diphenylacrylate (7b)

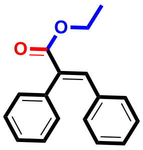 Prepared as described for **7a**; the reaction of diphenylacetylene (0.223 mmol, 50 mg), 5 wt% Pd/C (0.011 mmol, 23 mg), Xantphos (0.0223 mmol, 13 mg), PTSA (0.14 mmol, 27 mg), ethanol (0.5 ml), DMF (1.5 mL) and oxalic acid (0.892 mmol, 80 mg), in 0.5 mL of

DMF gave **7b** after purification silica gel (mesh 60:120) column chromatography (5% EtOAc in *n*-hexane) as white solid (58 mg, 82% yield).

^{1}H NMR (500 MHz, $CDCl_3$) δ (*ppm*) 7.84 (s, 1H), 7.38 – 7.34 (m, 3H), 7.24 – 7.14 (m, 5H), 7.06 – 7.03 (m, 2H), 4.27 (q, *J* = 7.1 Hz, 2H), 1.30 (t, *J* = 7.1 Hz, 3H); ^{13}C NMR (126 MHz, $CDCl_3$) δ (*ppm*) 167.81, 140.09, 135.87, 134.65, 132.77, 130.54, 129.76, 128.92, 128.53, 128.14, 127.72, 61.17, 14.25. ESI-MS $[M-H]^-$ *(m/z)* calcd. for $C_9H_7O_2^-$ is 147.0446, obsd. 147.0453. ESI-MS $[M-H]^+$ (m/z) calcd. for C17H17O2+ is 253.1223, obsd. 253.1226.

propyl (E)-2,3-diphenylacrylate (7c)

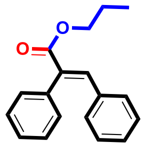

Prepared as described for **7a**; the reaction of diphenylacetylene (0.223 mmol, 50 mg), 5 wt% Pd/C (0.011 mmol, 23 mg), Xantphos (0.0223 mmol, 13 mg), PTSA (0.14 mmol, 27 mg), propanol (0.5 ml), DMF (1.5 mL) and oxalic acid (0.892 mmol, 80 mg), in 0.5 mL of DMF gave **7c** after purification silica gel (mesh 60:120) column chromatography (3-5% EtOAc in *n*-hexane) as white solid (59 mg, 79% yield).

^{1}H NMR (400 MHz, $CDCl_3$) δ (*ppm*) 7.84 (s, 1H), 7.39 – 7.33 (m, 3H), 7.23 – 7.13 (m, 5H), 7.06 – 7.04 (m, 2H), 4.17 (t, *J* = 6.6 Hz, 2H), 1.72-1.63 (m, 2H), 0.92 (t, *J* = 7.4 Hz, 3H); ^{13}C NMR (101 MHz, $CDCl_3$) δ (*ppm*) 167.87, 140.03, 135.97, 134.70, 132.88, 130.56, 129.74, 128.92, 128.50, 128.15, 127.68, 66.72, 22.01, 10.40. ESI-MS $[M-H]^+$ (m/z) calcd. for C18H19O2+ is 267.1380, obsd. 267.1387.

butyl (E)-2,3-diphenylacrylate (7d)

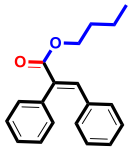

Prepared as described for **7a**; the reaction of diphenylacetylene (0.223 mmol, 50 mg), 5 wt% Pd/C (0.011 mmol, 23 mg), Xantphos (0.0223 mmol, 13 mg), PTSA (0.14 mmol, 27 mg), butanol (0.5 ml), DMF (1.5 mL) and oxalic acid (0.892 mmol, 80 mg), in 0.5 mL of DMF gave **7d** after purification silica gel (mesh 60:120) column chromatography (3-5% EtOAc in *n*-hexane) as semi-solid (60 mg, 76% yield).

^{1}H NMR (500 MHz, $CDCl_3$) δ (*ppm*) 7.86 (s, 1H), 7.40 – 7.35 (m, 3H), 7.25 – 7.15 (m, 5H), 7.08 – 7.06 (m, 2H), 4.24-4.22 (m, 2H), 1.69-1-63 (m, 2H), 1.41 – 1.34 (m, 2H), 0.94 (t, *J* =

7.4 Hz, 3H); ^{13}C NMR (126 MHz, $CDCl_3$) δ (*ppm*) 167.80, 139.98, 135.87, 134.60, 132.76, 130.50, 129.66, 128.87, 128.45, 128.09, 127.63, 64.96, 30.57, 19.08, 13.64. ESI-MS $[M-H]^+$ *(m/z)* calcd. for $C_{19}H_{21}O_2^+$ is 281.1536, obsd. 281.1541.

hexyl(E)-2,3-diphenylacrylate (7e)

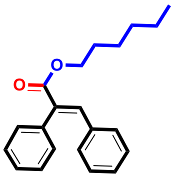

Prepared as described for **7a**; the reaction of diphenylacetylene (0.223 mmol, 50 mg), 5 wt% Pd/C (0.011 mmol, 23 mg), Xantphos (0.0223 mmol, 13 mg), PTSA (0.14 mmol, 27 mg), butanol (0.5 ml), DMF (1.5 mL) and oxalic acid (0.892 mmol, 80 mg) in 0.5 mL of DMF gave **7e** after purification silica gel (mesh 60:120) column chromatography (3-5% EtOAc in *n*-hexane) as semi-solid (58 mg, 71% yield).

^{1}H NMR (400 MHz, $CDCl_3$) δ (*ppm*) 7.85 (s, 1H), 7.39 – 7.34 (m, 3H), 7.24 – 7.14 (m, 5H), 7.07-7.05 (m, 2H), 4.21 (t, J = 6.7 Hz, 2H), 1.67 – 1.62 (m, 2H), 1.35 – 1.27 (m, 6H), 0.91 – 0.88 (m, 3H); ^{13}C NMR (101 MHz, $CDCl_3$) δ (*ppm*) 167.84, 139.98, 135.96, 134.68, 132.88, 130.53, 129.72, 128.89, 128.47, 128.12, 127.65, 65.29, 31.30, 28.50, 25.51, 22.41, 13.92. ESI-MS $[M-H]^+$ *(m/z)* calcd. for $C_{21}H_{25}O_2^+$ is 309.1849, obsd. 309.1861.

ethyl (E)-2,3-di-*p*-tolylacrylate (7f)

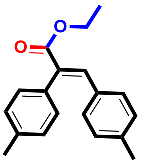

Prepared as described for **7a**; the reaction of 1,2-di-*p*-tolylethyne (0.242 mmol, 50 mg), 5 wt% Pd/C (0.0121 mmol, 13 mg), Xantphos (0.0242 mmol, 14 mg), PTSA (0.121 mmol, 23 mg), ethanol (0.5 ml), DMF (1.5 mL) and oxalic acid (0.968 mmol, 87 mg), in 0.5 mL of DMF gave **7f** after purification silica gel (mesh 60:120) column chromatography (5% EtOAc in *n*-hexane) as white solid (56 mg, 83% yield).

^{1}H NMR (500 MHz, $CDCl_3$) δ (*ppm*) 7.82 (s, 1H), 7.20 (d, J = 7.9 Hz, 2H), 7.14 (d, J = 7.6 Hz, 2H), 6.99 (s, 4H), 4.29 (q, J = 7.1 Hz, 2H), 2.41 (s, 3H), 2.29 (s, 3H), 1.32 (t, J = 7.0 Hz, 3H); ^{13}C NMR (126 MHz, $CDCl_3$) δ (*ppm*) 168.03, 139.83, 139.05, 137.24, 133.00, 131.93, 131.69, 130.51, 129.55, 129.25, 128.84, 60.97, 21.29, 21.24, 14.24. ESI-MS $[M-H]^+$ *(m/z)* calcd. for $C_{19}H_{20}O_2^+$ is 281.1536, obsd. 281.1542.

ethyl (E)-2,3-bis(3-methoxyphenyl)acrylate (7g)

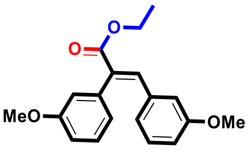

Prepared as described for **7a**; the reaction of 1,2-bis(3-methoxyphenyl)ethyne (0.21 mmol, 50 mg), 5 wt% Pd/C (0.0105 mmol, 11 mg), Xantphos (0.021 mmol, 12 mg), PTSA (0.105 mmol, 20 mg), ethanol (0.5 ml), DMF (1.5 mL) and oxalic acid (0.84 mmol, 76 mg), in 0.5 mL of DMF gave **7g** after purification silica gel (mesh 60:120) column chromatography (8% EtOAc in *n*-hexane) as white solid (47 mg, 72% yield).

^{1}H NMR (400 MHz, $CDCl_3$) δ (*ppm*) 7.79 (s, 1H), 7.32 – 7.26 (m, 1H), 7.11 (t, J = 8.0 Hz, 1H), 6.91-6.88 (m, 1H), 6.85-6.82 (m, 1H), 6.79 – 6.74 (m, 3H), 6.56 (t, J = 2.1 Hz, 1H), 4.28 (q, J = 7.1 Hz, 2H), 3.76 (s, 3H), 3.50 (s, 3H), 1.30 (t, J = 7.1 Hz, 3H); ^{13}C NMR (101 MHz, $CDCl_3$) δ (*ppm*) 167.58, 159.77, 159.05, 139.93, 137.33, 135.76, 132.72, 129.61, 129.09, 123.66, 122.11, 115.94, 115.02, 114.34, 113.57, 61.14, 55.23, 54.76, 14.23. ESI-MS $[M-H]^+$ *(m/z)* calcd. for $C_{19}H_{21}O_4^+$ is 313.1434, obsd. 313.1443

ethyl (E)-2,3-bis(4-(*tert*-butyl)phenyl)acrylate (7h)

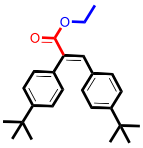

Prepared as described for **7a**; the reaction of 1,2-bis(4-(*tert*-butyl)phenyl)ethyne (0.172 mmol, 50 mg), 5 wt% Pd/C (0.0086 mmol, 18 mg), Xantphos (0.0172 mmol, 10 mg), PTSA (0.086 mmol, 16 mg), ethanol (0.5 ml), DMF (1.5 mL) and oxalic acid (0.69 mmol, 63 mg), in 0.5 mL of DMF gave **7h** after purification silica gel (mesh 60:120) column chromatography (5% EtOAc in *n*-hexane) as white solid (47 mg, 76% yield).

^{1}H NMR (400 MHz, $CDCl_3$) δ (*ppm*) 7.80 (s, 1H), 7.41 (d, J = 8.3 Hz, 2H), 7.20 – 7.17 (m, 4H), 7.08 – 6.96 (m, 2H), 4.28 (q, J = 7.1 Hz, 2H), 1.38 (s, 9H), 1.32 (t, J = 7.1 Hz, 3H), 1.27 (s, 9H); ^{13}C NMR (101 MHz, $CDCl_3$) δ (*ppm*) 168.15, 152.25, 150.51, 139.64, 133.07, 131.92, 131.74, 130.48, 129.26, 125.44, 125.09, 60.97, 34.64, 34.58, 31.37, 31.07, 14.28. ESI-MS $[M-H]^+$ *(m/z)* calcd. for $C_{25}H_{33}O_2^+$ is 365.2475, obsd. 365.2487.

ethyl (E)-2,3-bis(4-fluorophenyl)acrylate (7i)

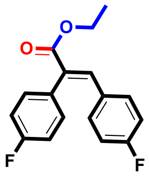

Prepared as described for **7a**; the reaction of 1,2-bis(4-fluorophenyl)ethyne (0.21 mmol, 50 mg), 5 wt% Pd/C (0.0117 mmol, 25 mg), Xantphos (0.0234 mmol, 14 mg), PTSA (0.117 mmol, 22 mg), ethanol (0.5 ml), DMF (1.5 mL) and oxalic acid (0.934 mmol, 84 mg), in 0.5 mL of DMF gave **7i** after purification silica gel (mesh 60:120) column chromatography (5% EtOAc in *n*-hexane) as white solid (55 mg, 82% yield).

^{1}H NMR (500 MHz, $CDCl_3$) δ (*ppm*) 7.80 (s, 1H), 7.19 – 7.17 (m, 2H), 7.08 – 7.01 (m, 4H), 6.88 – 6.85 (m, 2H), 4.29 – 4.26 (m, 2H), 1.30 (t, J = 7.2 Hz, 3H); ^{13}C NMR (126 MHz, $CDCl_3$) δ (*ppm*) 167.46, 163.56 (d, J = 52.8 Hz), 161.58 (d, J = 48.5 Hz), 139.22, 132.36 (d, J = 8.1 Hz), 131.55 (d, J = 7.6 Hz), 131.43 (d, J = 5.9 Hz), 130.62, 115.74 (d, J = 21.3 Hz), 115.37 (d, J = 21.6 Hz), 61.29, 14.20. ESI-MS [M-H]$^+$ *(m/z)* calcd. for $C_{17}H_{15}F_2O_2^+$ is 289.1035, obsd. 289.1035.

ethyl (E)-2,3-bis(3,4-difluorophenyl)acrylate (7j)

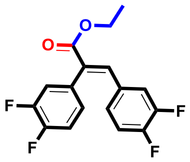

Prepared as described for **7a**; the reaction of 1,2-bis(3,4-difluorophenyl)ethyne (0.20 mmol, 50 mg), 5 wt% Pd/C (0.01 mmol, 21 mg), Xantphos (0.020 mmol, 12 mg), PTSA (0.10 mmol, 19 mg), ethanol (0.5 mL), DMF (1.5 mL) and oxalic acid (0.80 mmol, 72 mg), in 0.5 mL of DMF gave **7j** after purification silica gel (mesh 60:120) column chromatography (5% EtOAc in *n*-hexane) as white solid (47 mg, 72% yield).

^{1}H NMR (500 MHz, $CDCl_3$) δ (*ppm*) 7.74 (s, 1H), 7.20-7.14 (m, 1H), 7.06 – 6.98 (m, 2H), 6.93-6.90 (m, 1H), 6.84 – 6.79 (m, 2H), 4.27 (q, J = 7.1 Hz, 2H), 1.30 (t, J = 7.1 Hz, 3H); ^{13}C NMR (126 MHz, $CDCl_3$) δ (*ppm*) 166.63, 152.27 – 150.54 (m), 149.96 – 148.60 (m), 138.63, 131.80 (dd, J = 6.0, 4.2 Hz), 131.61, 131.15, 127.11 (dd, J = 6.6, 3.6 Hz), 126.10 (dd, J = 6.0, 3.6 Hz), 118.95 (d, J = 11.6 Hz), 118.80 (d, J = 12.1 Hz), 117.83, 117.70, 117.41, 117.27, 61.61, 14.14. ESI-MS [M-H]$^+$ *(m/z)* calcd. for $C_{17}H_{13}F_4O_2^+$ is 325.0846, obsd. 325.0850.

ethyl (E)-2,3-bis(4-(trifluoromethyl)phenyl)acrylate (7k)

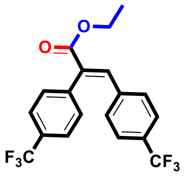 Prepared as described for **7a**; the reaction of 1,2-bis(4-(trifluoromethyl)phenyl)ethyne (0.159 mmol, 50 mg), 5wt% Pd/C (0.0079 mmol, 17 mg), Xantphos (0.0159 mmol, 9 mg), PTSA (0.079 mmol, 15 mg), ethanol (0.5 ml), DMF (1.5 mL) and oxalic acid (0.636 mmol, 57 mg), in 0.5 mL of DMF gave **7k** after purification silica gel (mesh 60:120) column chromatography (5% EtOAc in *n*-hexane) as pale yellow solid (50 mg, 80% yield).

^{1}H NMR (500 MHz, $CDCl_3$) δ (*ppm*) 7.91 (s, 1H), 7.64-7.62 (m, 2H), 7.44 (d, J = 8.3 Hz, 2H), 7.35 – 7.33 (m, 2H), 7.14-7.12 (m, 2H), 4.32 – 4.28 (m, 2H), 1.33 – 1.30 (m, 3H); ^{13}C NMR (126 MHz, $CDCl_3$) δ (*ppm*) 166.61, 139.29, 138.95, 137.63, 133.76, 130.86, 130.60, 130.45, 130.36, 130.26, 130.10, 125.61 (q, J = 3.7 Hz), 125.28 (q, J = 3.7 Hz), 61.71, 14.15. ESI-MS $[M-H]^+$ *(m/z)* calcd. for $C_{19}H_{15}F_6O_2^+$ is 389.0971, obsd. 389.0981.

ethyl (E)-2,3-di(thiophen-3-yl)acrylate (7l)

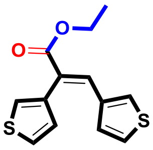 Prepared as described for **7a**; the reaction of 1,2-di(thiophen-3-yl)ethyne (0.263 mmol, 50 mg), 5 wt% Pd/C (0.0131 mmol, 27 mg), Xantphos (0.0263 mmol, 15 mg), PTSA (0.131 mmol, 25 mg), ethanol (0.5 mL), DMF (1.5 mL) and oxalic acid (1.05 mmol, 118 mg), in 0.5 mL of DMF gave **7l** after purification silica gel (mesh 60:120) column chromatography (8% EtOAc in *n*-hexane) as pale yellow solid (47 mg, 68% yield).

^{1}H NMR (500 MHz, $CDCl_3$) δ (*ppm*) 7.85 (d, J = 0.7 Hz, 1H), 7.38-7.36 (m, 1H), 7.20 – 7.19 (m, 2H), 7.11-7.09 (m, 1H), 6.99-6.98 (m, 1H), 6.58-6.57 (m, 1H), 4.27 (q, J = 7.1 Hz, 2H), 1.30 (t, J = 7.1 Hz, 3H); ^{13}C NMR (126 MHz, $CDCl_3$) δ (*ppm*) 167.36, 136.64, 135.56, 134.60, 129.17, 128.80, 127.87, 125.74, 125.41, 125.35, 124.45, 60.96, 14.18. ESI-MS $[M-H]^+$ *(m/z)* calcd. for $C_{13}H_{13}S_2O_2^+$ is 265.0351, obsd. 265.0353.

ethyl (E)-2-phenylbut-2-enoate (7m)

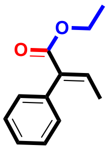 Prepared as described for **7a**; the reaction of ethyl prop-1-yn-1-ylbenzene (0.431 mmol, 50 mg), 5 wt% Pd/C (0.0215 mmol, 44 mg), Xantphos (0.0431 mmol, 25 mg), PTSA (0.215 mmol, 41 mg), ethanol (0.5 ml), DMF (1.5 mL) and oxalic acid (1.72 mmol, 118 mg),

in 0.5 mL of DMF gave **7m** after purification silica gel (mesh 60:120) column chromatography (3-5% EtOAc in *n*-hexane) as colourless liquid (60 mg, 74% yield).

^{1}H NMR (500 MHz, $CDCl_3$) δ (*ppm*) 7.70 (m, 1H), 7.41 – 7.39 (m, 4H), 7.33 – 7.31 (m, 1H), 4.28 (q, J = 6.9 Hz, 2H), 2.13 (d, J = 1.9 Hz, 3H), 1.36 (t, J = 7.0 Hz, 3H); ^{13}C NMR (126 MHz, $CDCl_3$) δ (*ppm*) 168.64, 138.59, 135.88, 129.57, 128.54, 128.28, 128.18, 60.82, 14.26, 13.99. ESI-MS $[M-H]^+$ *(m/z)* calcd. for $C_{12}H_{15}O_2^+$ is 191.1067, obsd. 191.1067.

H. ^{1}H, ^{13}C NMR and ESI-MS Spectra of compounds 2a-t, 3a-n, 5a-u and 7a-m

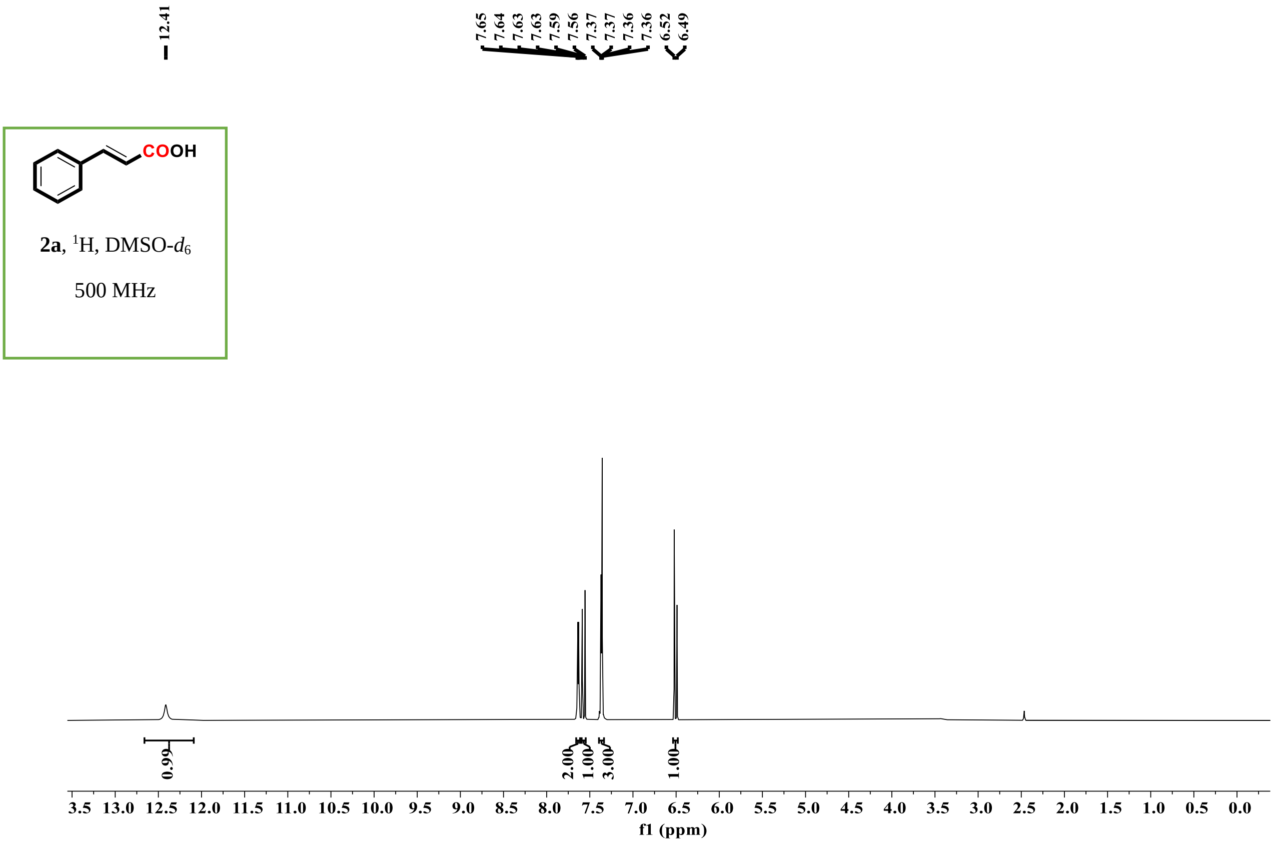

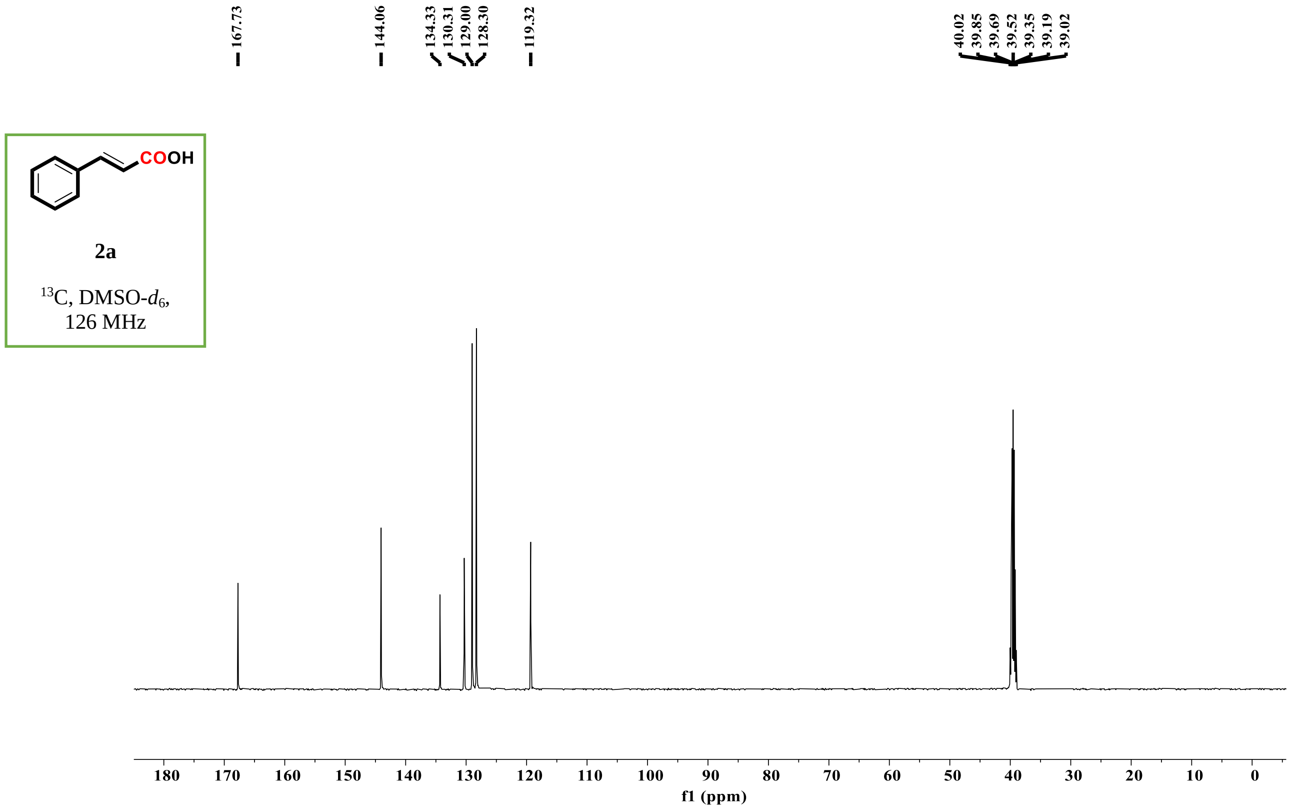

167.73
144.06
134.33
130.31
129.00
128.30
119.32
40.02
39.85
39.69
39.52
39.35
39.19
39.02
COOH
2a
13C, DMSO-d6,
126 MHz
180
170
160
150
140
130
120
110
100
90
80
70
60
50
40
30
20
10
0
f1 (ppm)

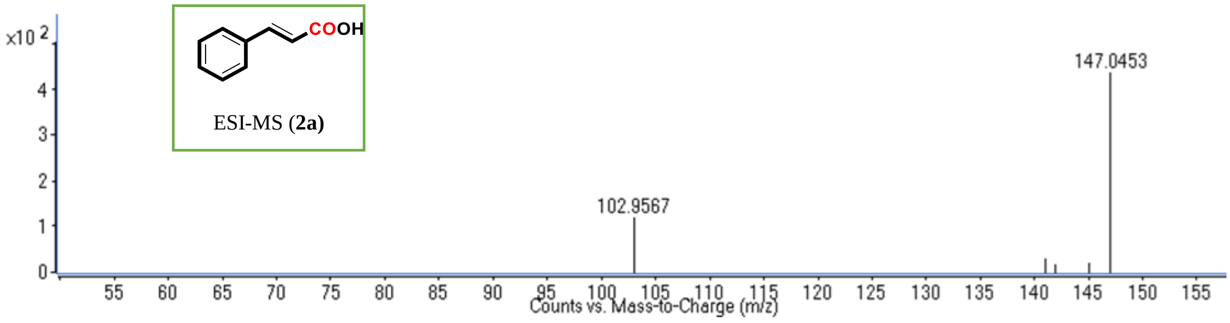

x10 2
4
3
2
1
0
COOH
ESI-MS (2a)
147.0453
102.9567
55
60
65
70
75
80
85
90
95
100
105
110
115
120
125
130
135
140
145
150
155
Counts vs. Mass-to-Charge (m/z)

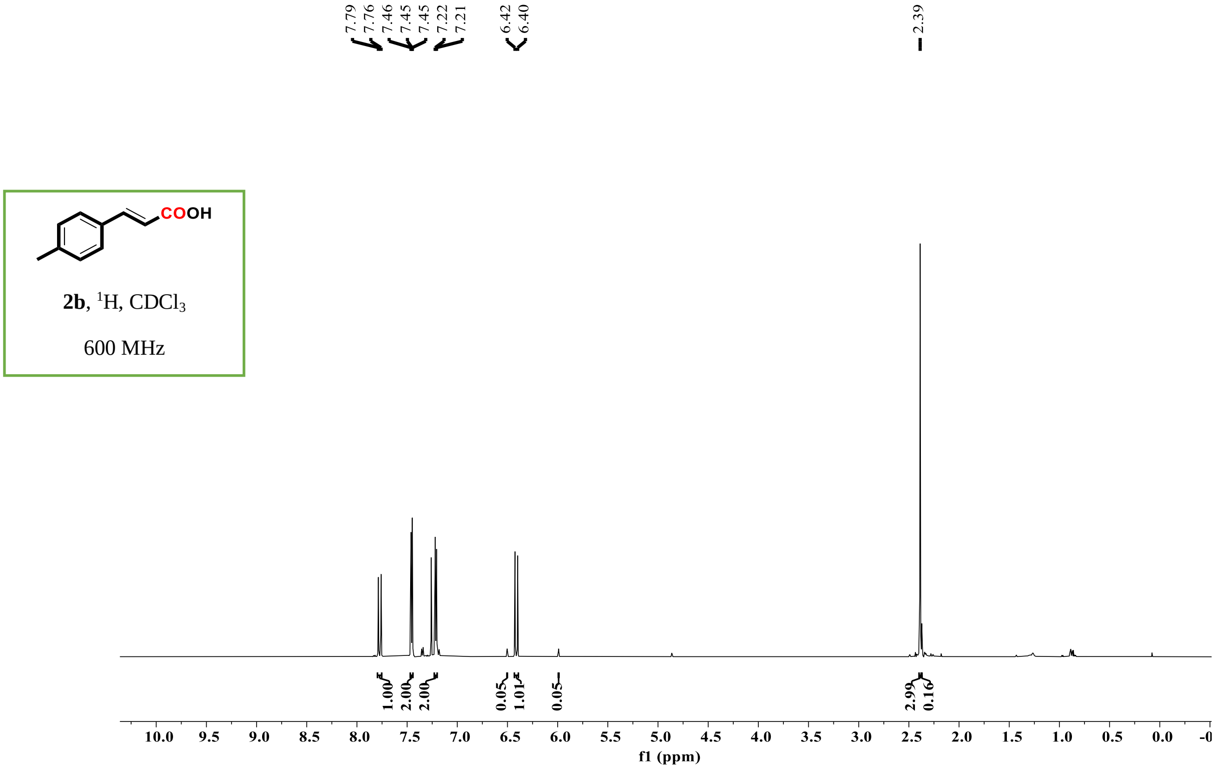
7.79
7.76
7.46
7.45
7.45
7.22
7.21
6.42
6.40
2.39
COOH
2b, 1H, CDCl3
600 MHz
1.00
2.00
2.00
0.05
1.01
0.05
2.99
0.16
10.0 9.5 9.0 8.5 8.0 7.5 7.0 6.5 6.0 5.5 5.0 4.5 4.0 3.5 3.0 2.5 2.0 1.5 1.0 0.5 0.0
f1 (ppm)

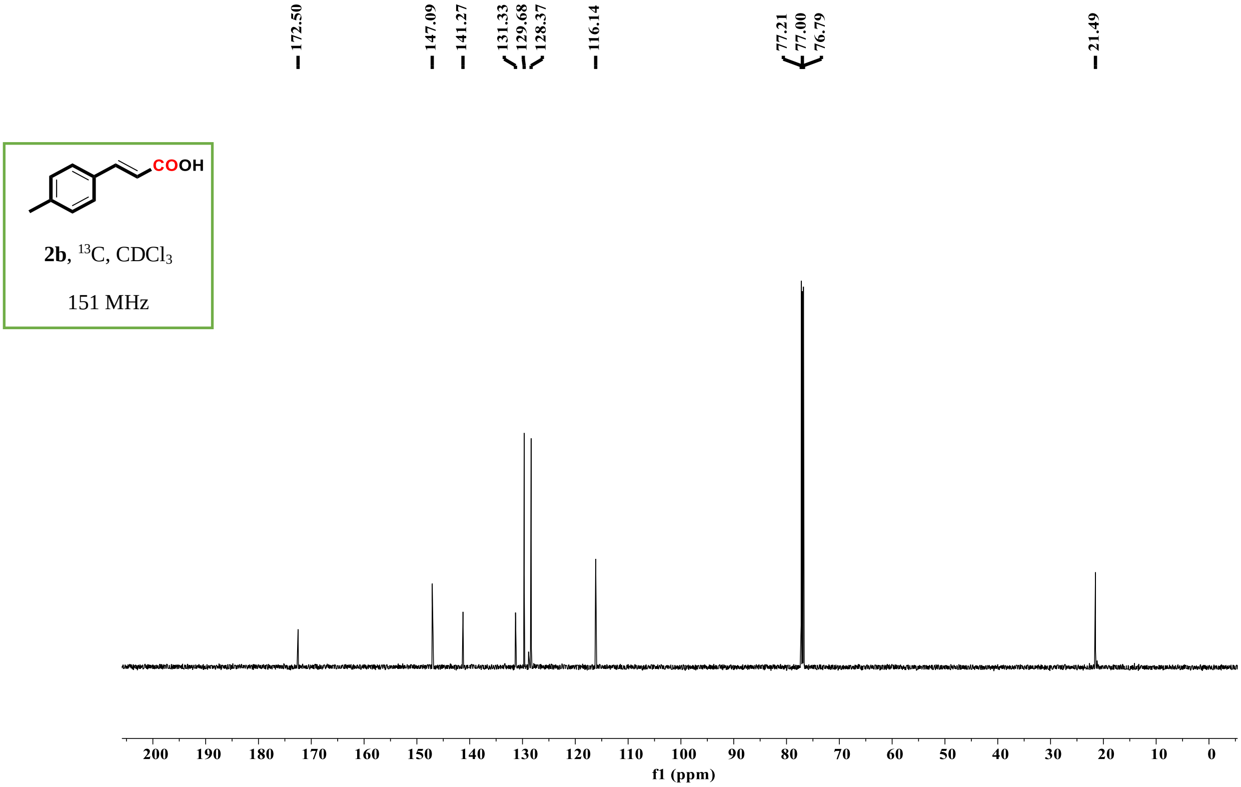
172.50
147.09
141.27
131.33
129.68
128.37
116.14
77.21
77.00
76.79
21.49
COOH
2b, 13C, CDCl3
151 MHz
200 190 180 170 160 150 140 130 120 110 100 90 80 70 60 50 40 30 20 10 0
f1 (ppm)

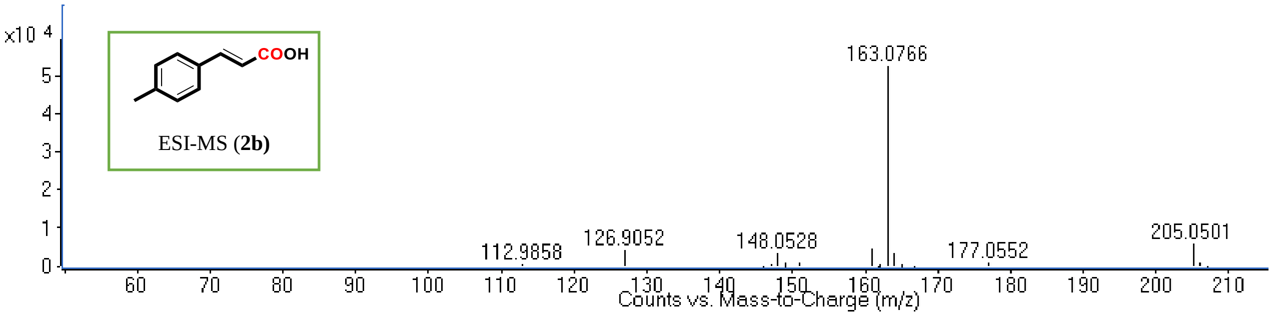
x10 4
5
4
3
2
1
0
COOH
ESI-MS (2b)
163.0766
112.9858
126.9052
148.0528
177.0552
205.0501
60 70 80 90 100 110 120 130 140 150 160 170 180 190 200 210
Counts vs. Mass-to-Charge (m/z)

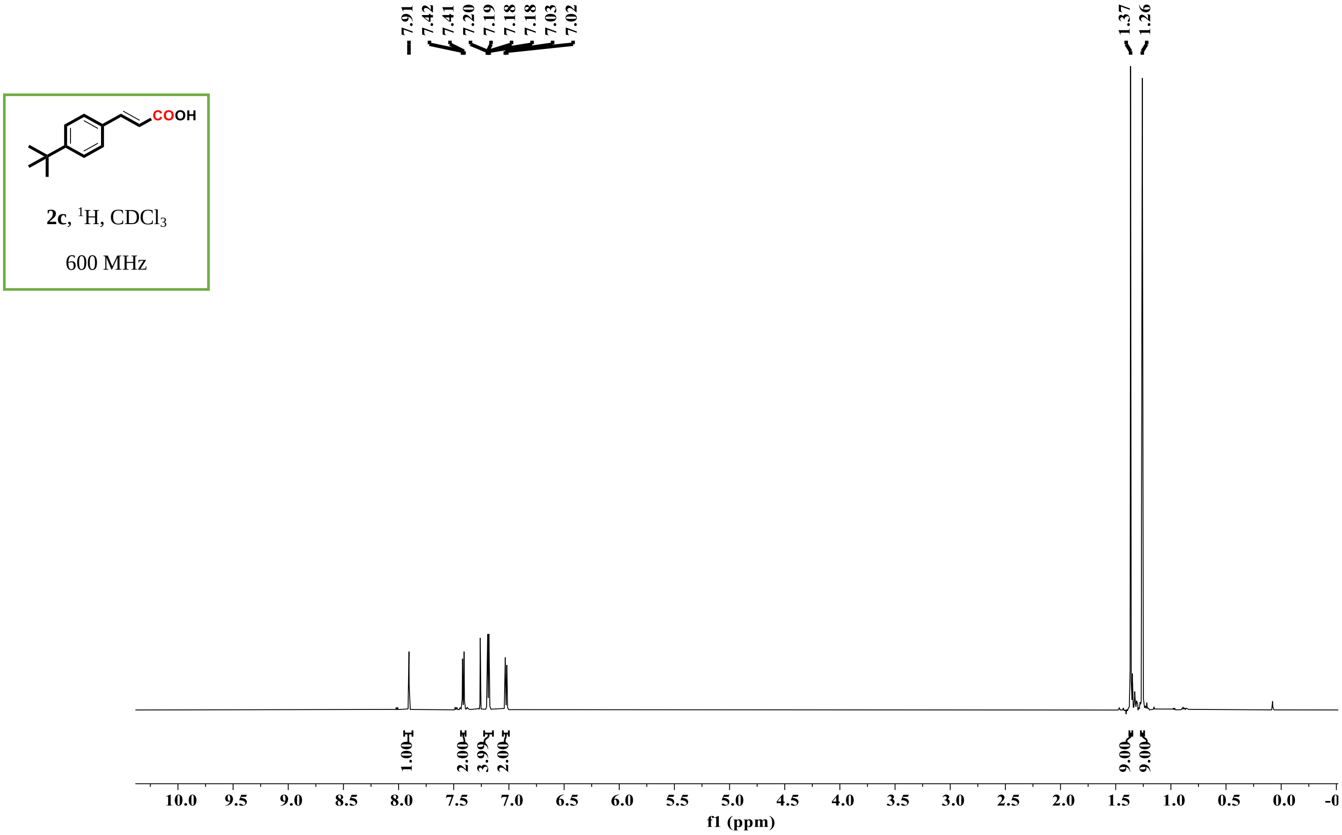
7.91
7.42
7.41
7.20
7.19
7.18
7.18
7.03
7.02
1.37
1.26
COOH
2c, ^{1}H, CDCl$_3$
600 MHz
1.00
2.00
3.99
2.00
9.00
9.00
10.0 9.5 9.0 8.5 8.0 7.5 7.0 6.5 6.0 5.5 5.0 4.5 4.0 3.5 3.0 2.5 2.0 1.5 1.0 0.5 0.0 -0
f1 (ppm)

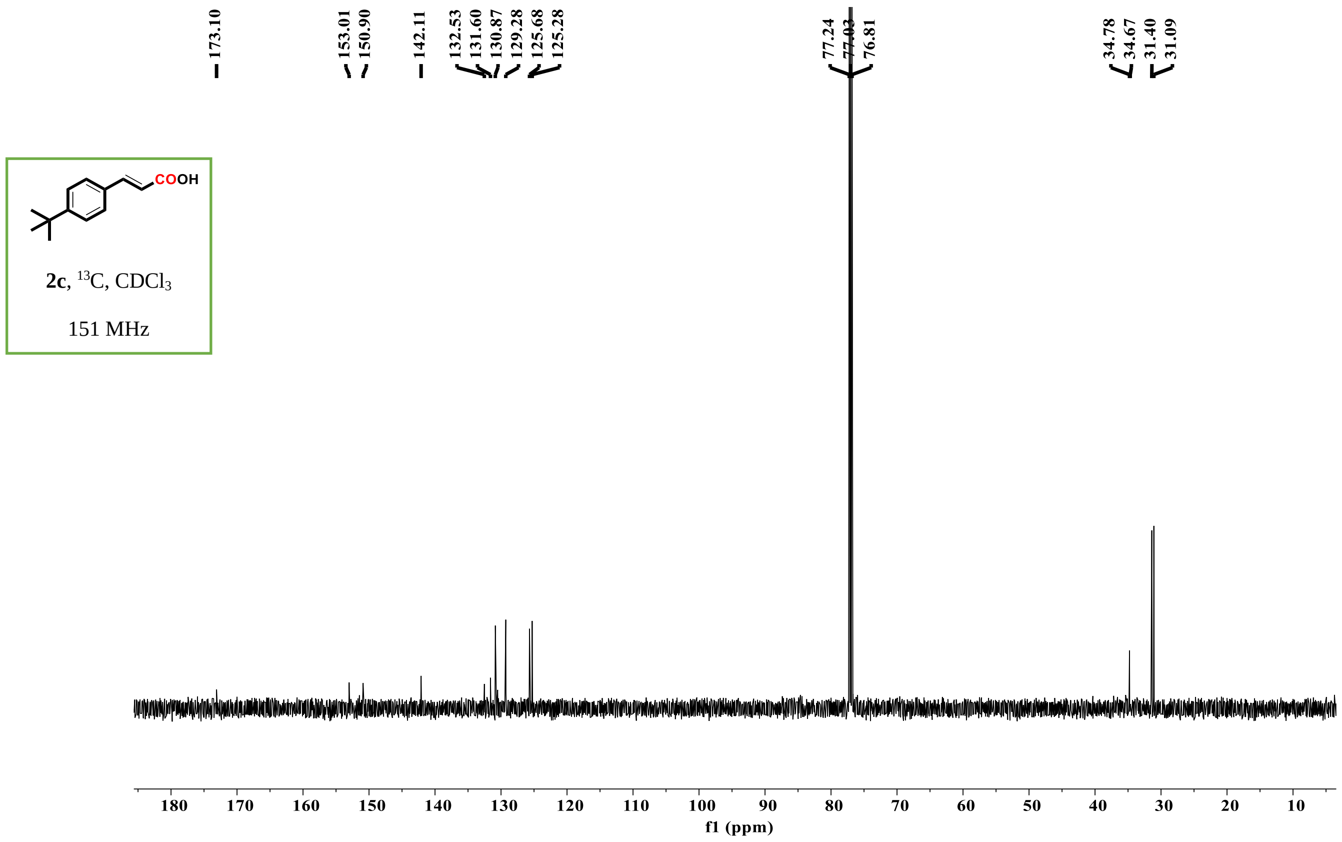
173.10
153.01
150.90
142.11
132.53
131.60
130.87
129.28
125.68
125.28
77.24
77.03
76.81
34.78
34.67
31.40
31.09
COOH
2c, 13C, CDCl3
151 MHz
180
170
160
150
140
130
120
110
100
90
80
70
60
50
40
30
20
10
f1 (ppm)

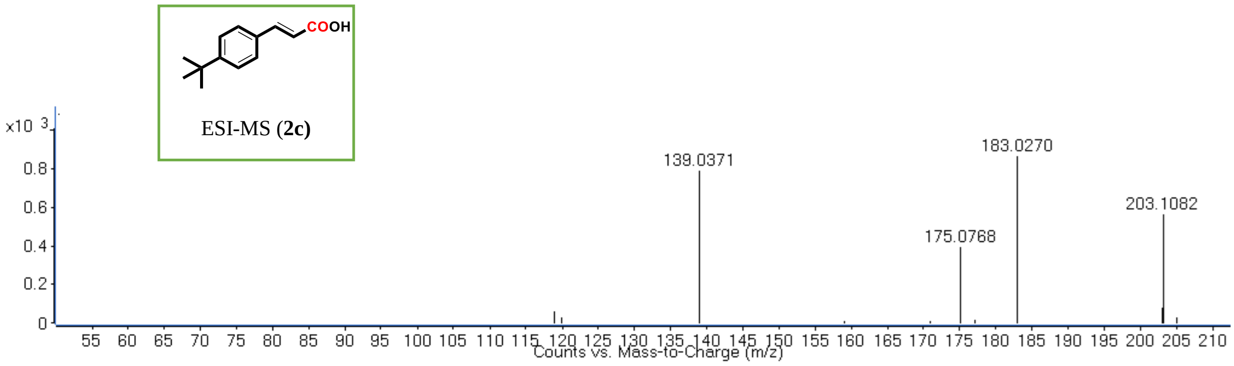
COOH
ESI-MS (2c)
x10 3
0.8
0.6
0.4
0.2
0
139.0371
183.0270
203.1082
175.0768
55 60 65 70 75 80 85 90 95 100 105 110 115 120 125 130 135 140 145 150 155 160 165 170 175 180 185 190 195 200 205 210
Counts vs. Mass-to-Charge (m/z)

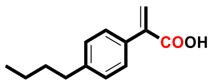

2d, ^{1}H, $CDCl_3$

500 MHz

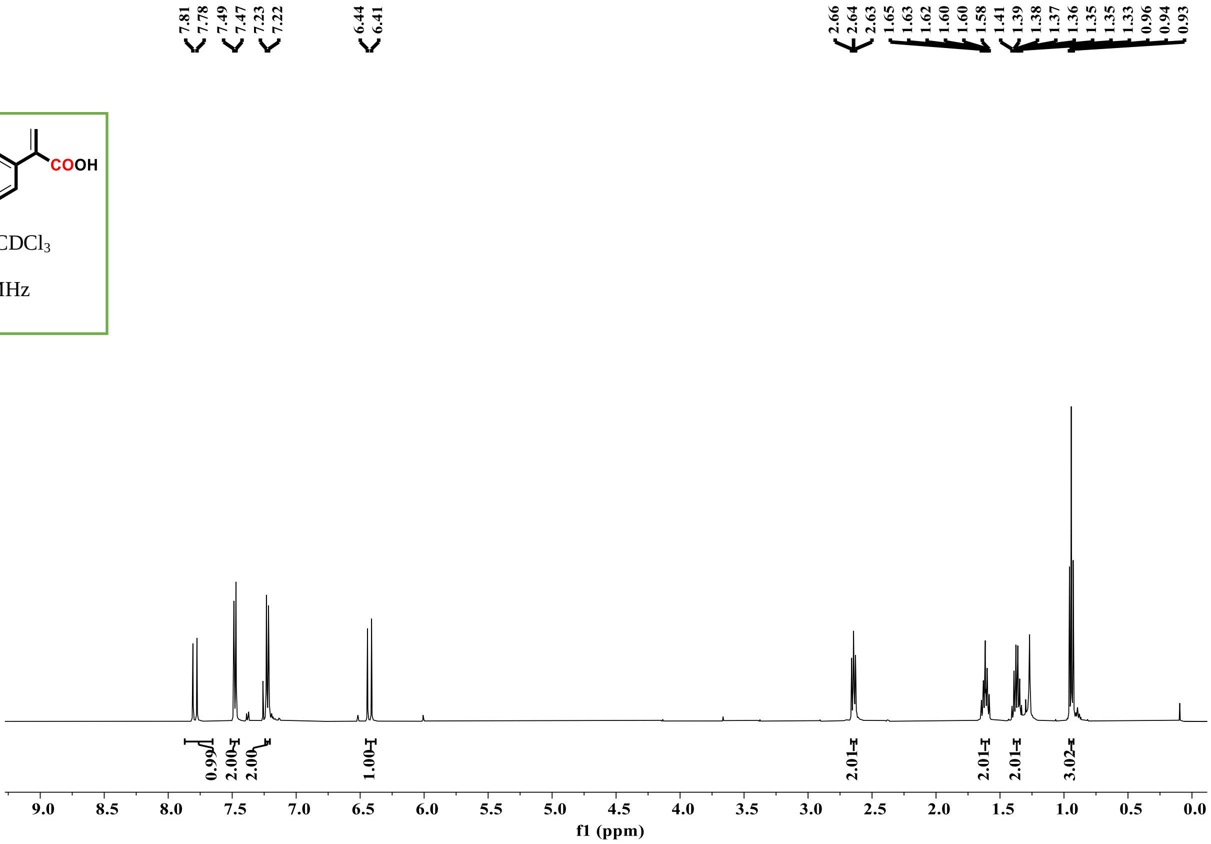

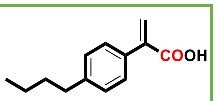

2d, ^{13}C, $CDCl_3$

126 MHz

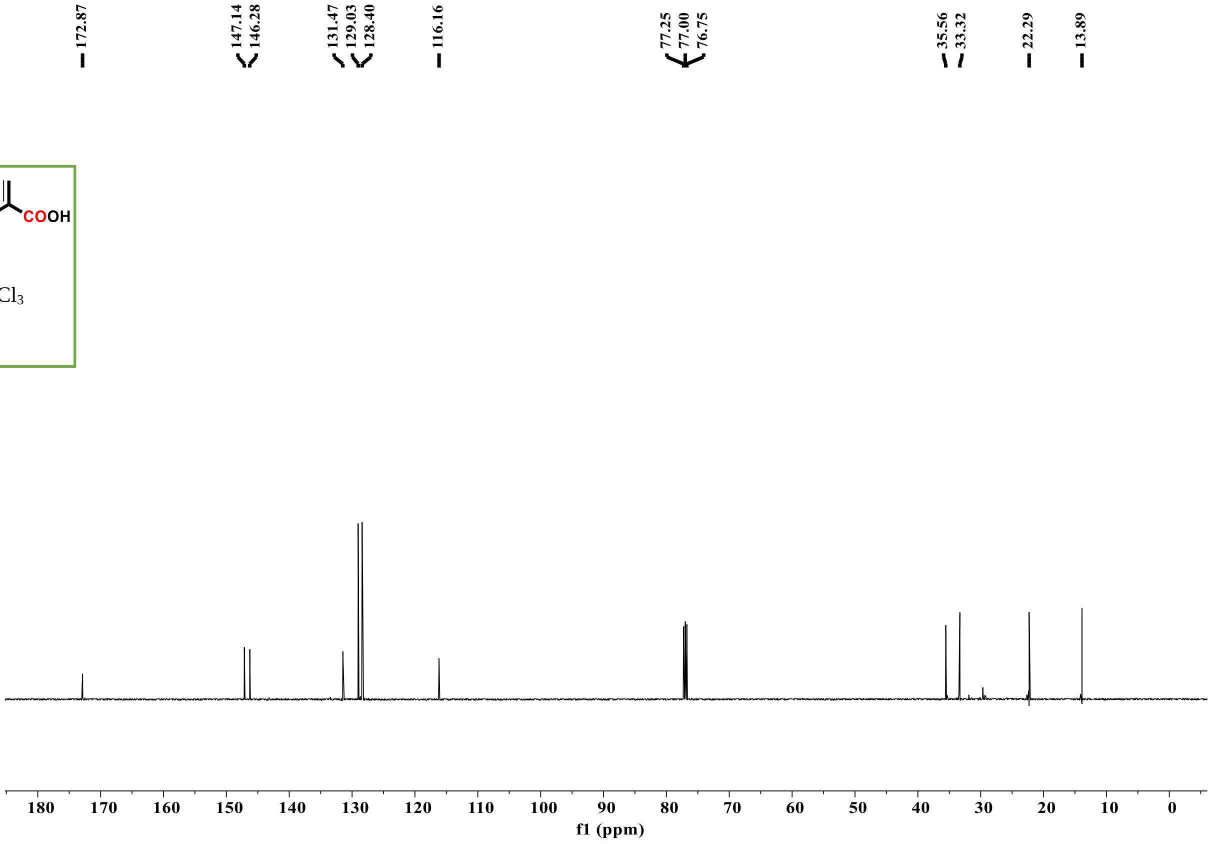

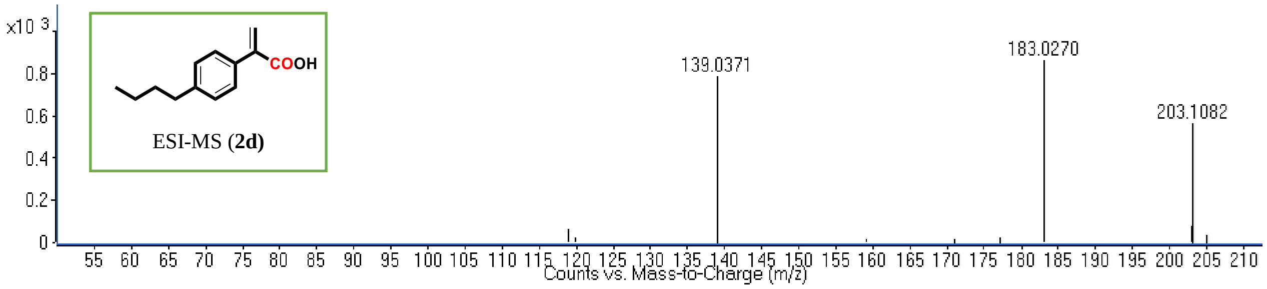
x10 3
0.8
0.6
0.4
0.2
0
COOH
ESI-MS (2d)
139.0371
183.0270
203.1082
55 60 65 70 75 80 85 90 95 100 105 110 115 120 125 130 135 140 145 150 155 160 165 170 175 180 185 190 195 200 205 210
Counts vs. Mass-to-Charge (m/z)

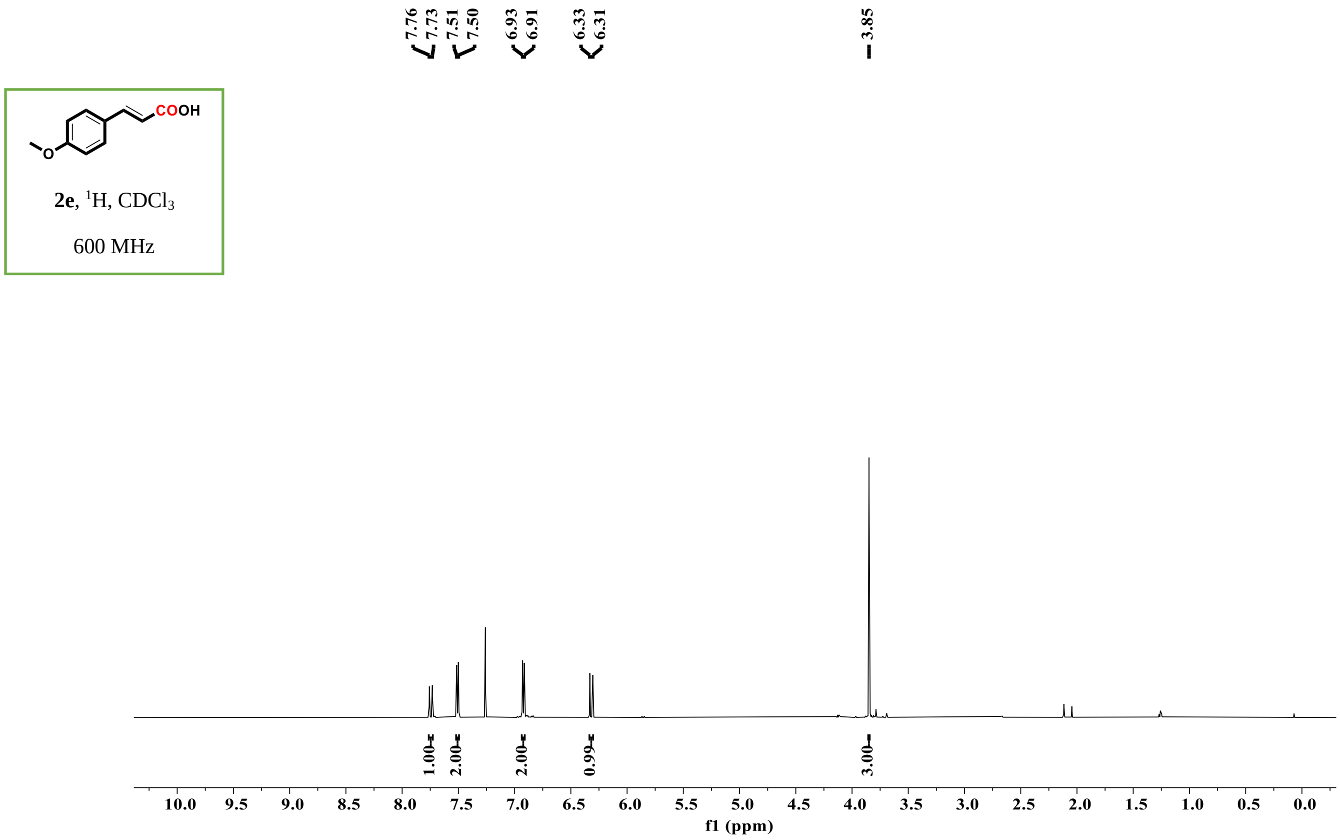
7.76
7.73
7.51
7.50
6.93
6.91
6.33
6.31
3.85
COOH
O
2e, 1H, CDCl3
600 MHz
1.00
2.00
2.00
0.99
3.00
10.0 9.5 9.0 8.5 8.0 7.5 7.0 6.5 6.0 5.5 5.0 4.5 4.0 3.5 3.0 2.5 2.0 1.5 1.0 0.5 0.0
f1 (ppm)

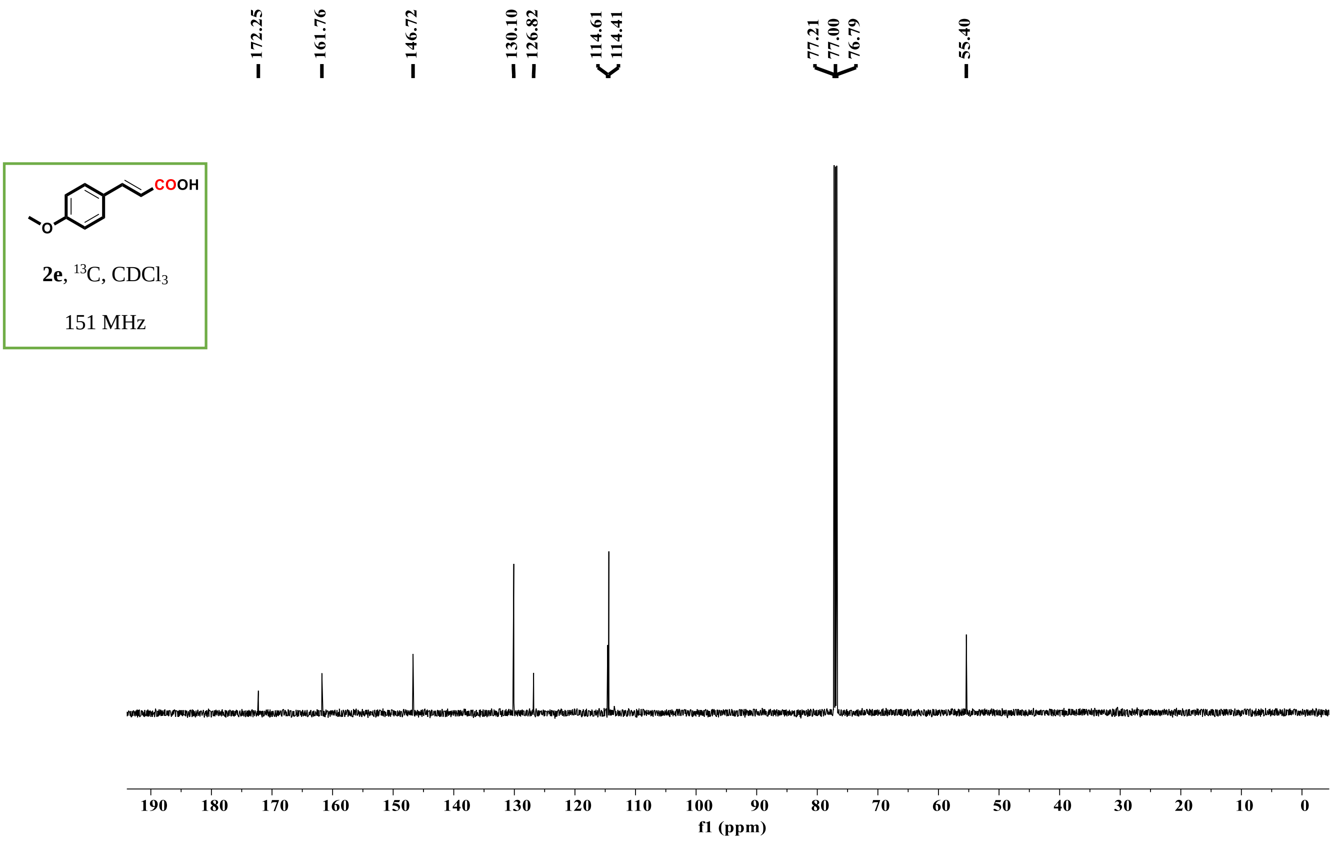

172.25
161.76
146.72
130.10
126.82
114.61
114.41
77.21
77.00
76.79
55.40
COOH
O
2e, 13C, CDCl3
151 MHz
190 180 170 160 150 140 130 120 110 100 90 80 70 60 50 40 30 20 10 0
f1 (ppm)

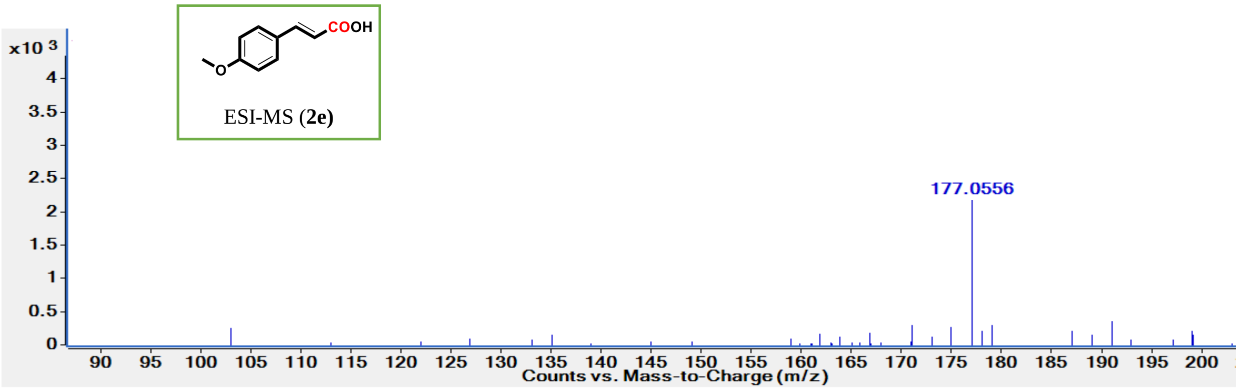

x10 3
4
3.5
3
2.5
2
1.5
1
0.5
0
COOH
O
ESI-MS (2e)
177.0556
90 95 100 105 110 115 120 125 130 135 140 145 150 155 160 165 170 175 180 185 190 195 200
Counts vs. Mass-to-Charge (m/z)

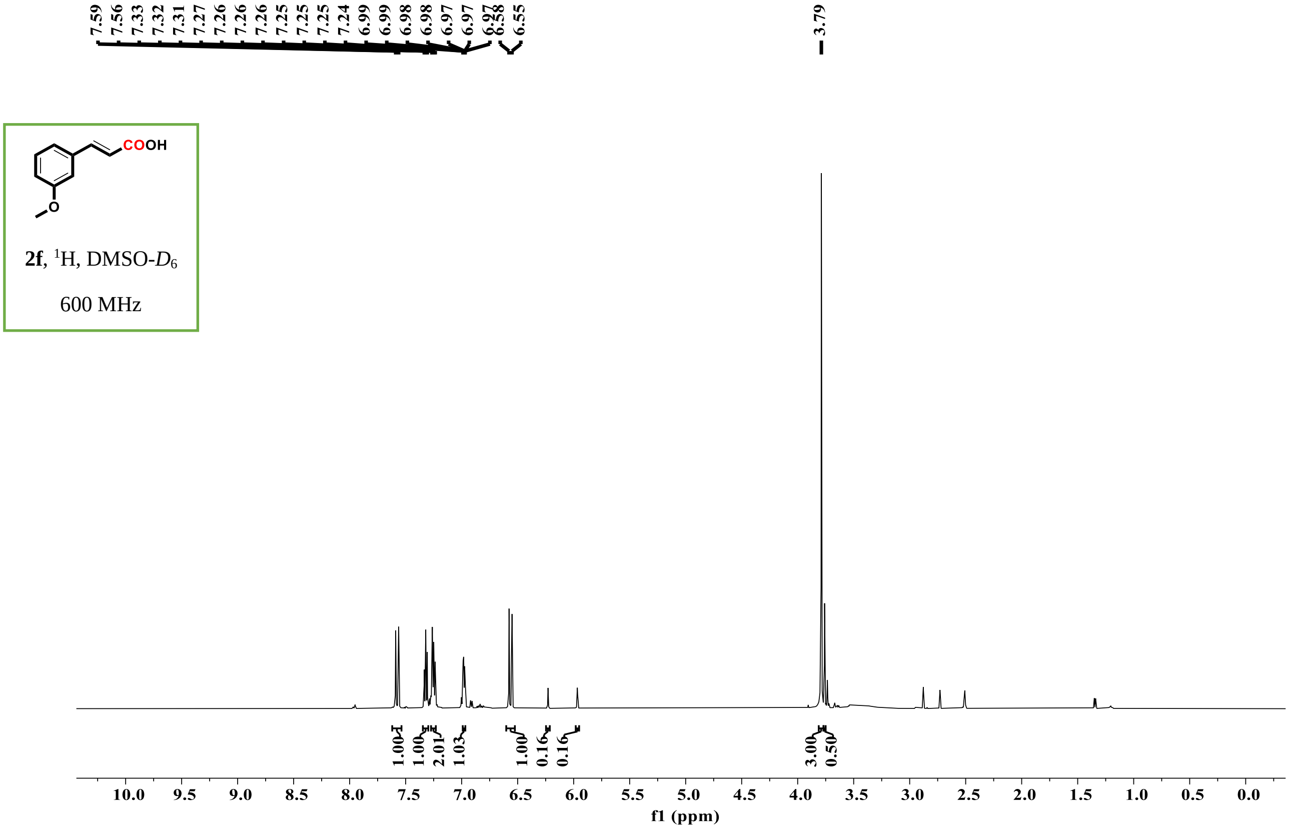
7.59
7.56
7.33
7.32
7.31
7.27
7.26
7.26
7.26
7.25
7.25
7.25
7.24
6.99
6.99
6.98
6.98
6.97
6.97
6.97
6.58
6.55
3.79
COOH
O
2f, 1H, DMSO-D6
600 MHz
1.00
1.00
2.01
1.03
1.00
0.16
0.16
3.00
0.50
10.0 9.5 9.0 8.5 8.0 7.5 7.0 6.5 6.0 5.5 5.0 4.5 4.0 3.5 3.0 2.5 2.0 1.5 1.0 0.5 0.0
f1 (ppm)

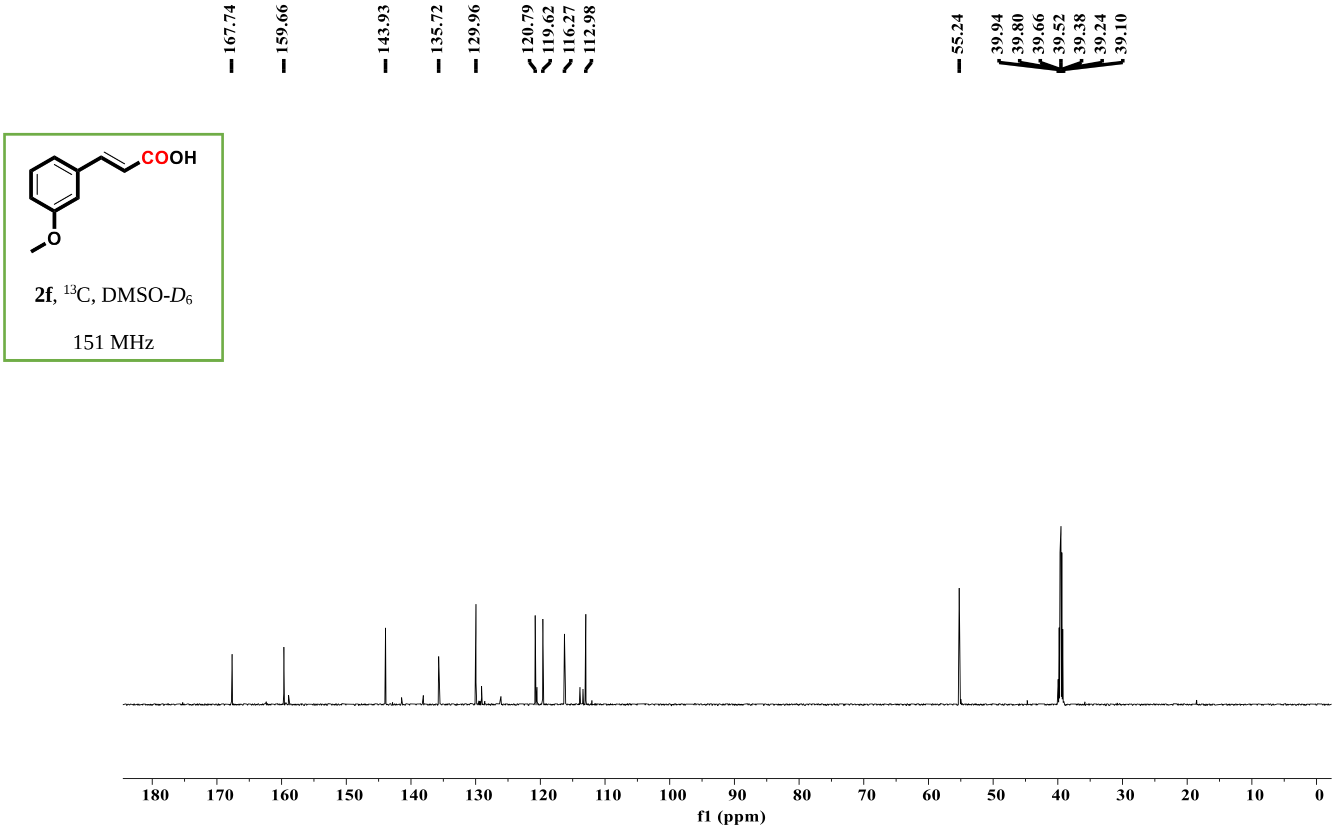
167.74
159.66
143.93
135.72
129.96
120.79
119.62
116.27
112.98
55.24
39.94
39.80
39.66
39.52
39.38
39.24
39.10
COOH
O
2f, 13C, DMSO-D6
151 MHz
180 170 160 150 140 130 120 110 100 90 80 70 60 50 40 30 20 10 0
f1 (ppm)

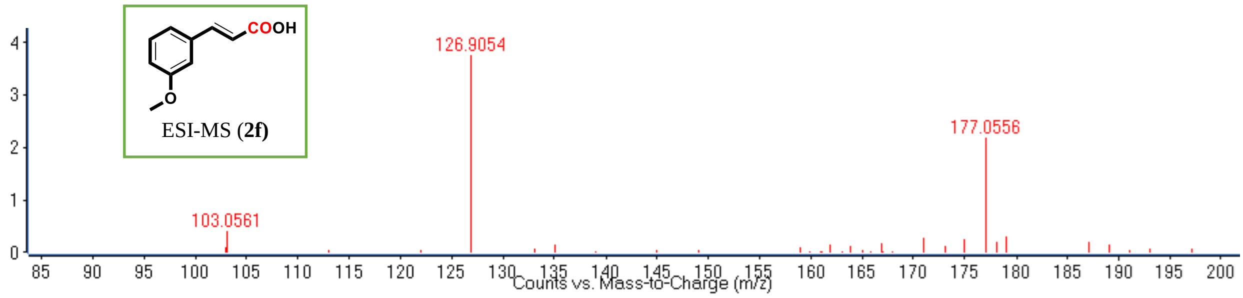

COOH
O
ESI-MS (2f)
126.9054
177.0556
103.0561
Counts vs. Mass-to-Charge (m/z)

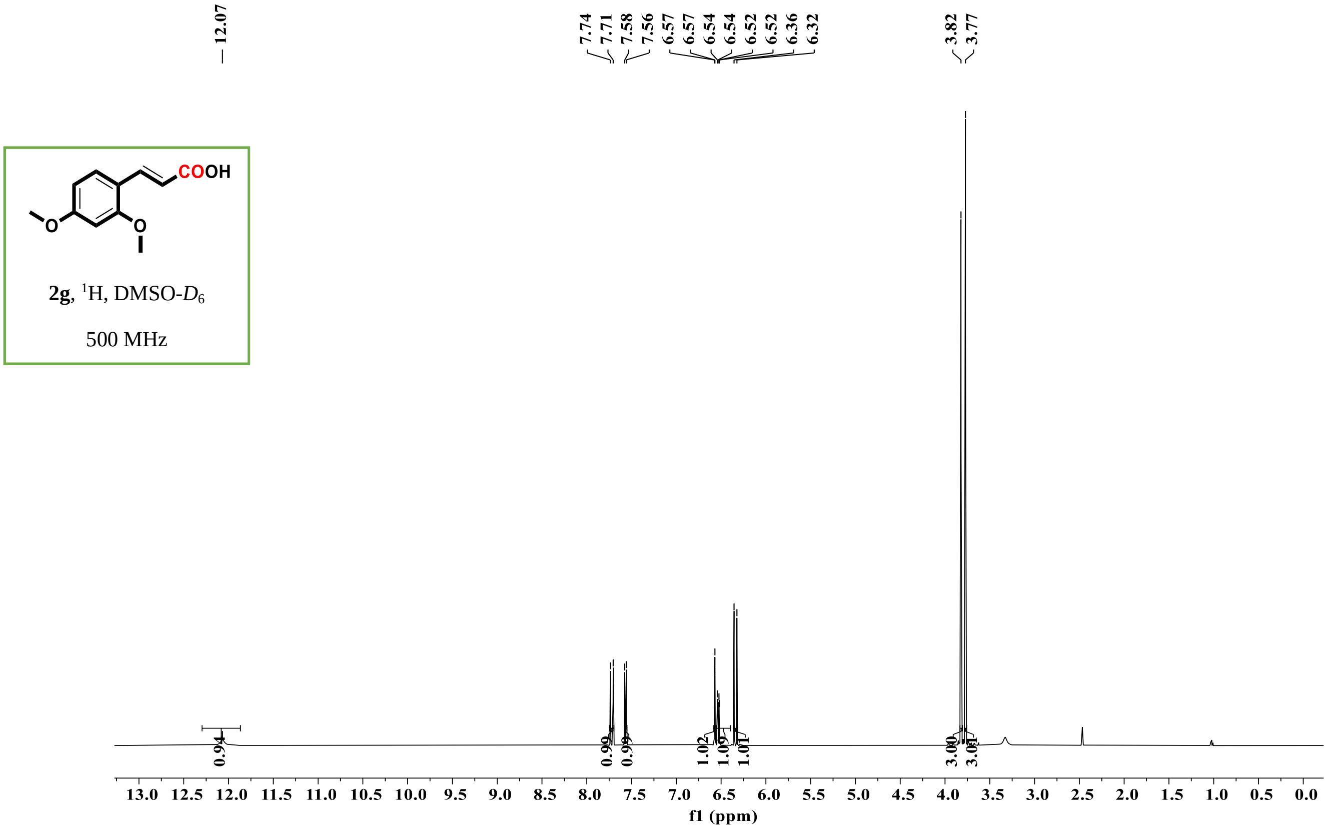

12.07
7.74
7.71
7.58
7.56
6.57
6.57
6.54
6.54
6.52
6.52
6.36
6.32
3.82
3.77
COOH
O
O
2g, ¹H, DMSO-D6
500 MHz
0.94
0.99
0.99
1.02
1.09
1.01
3.00
3.01
f1 (ppm)

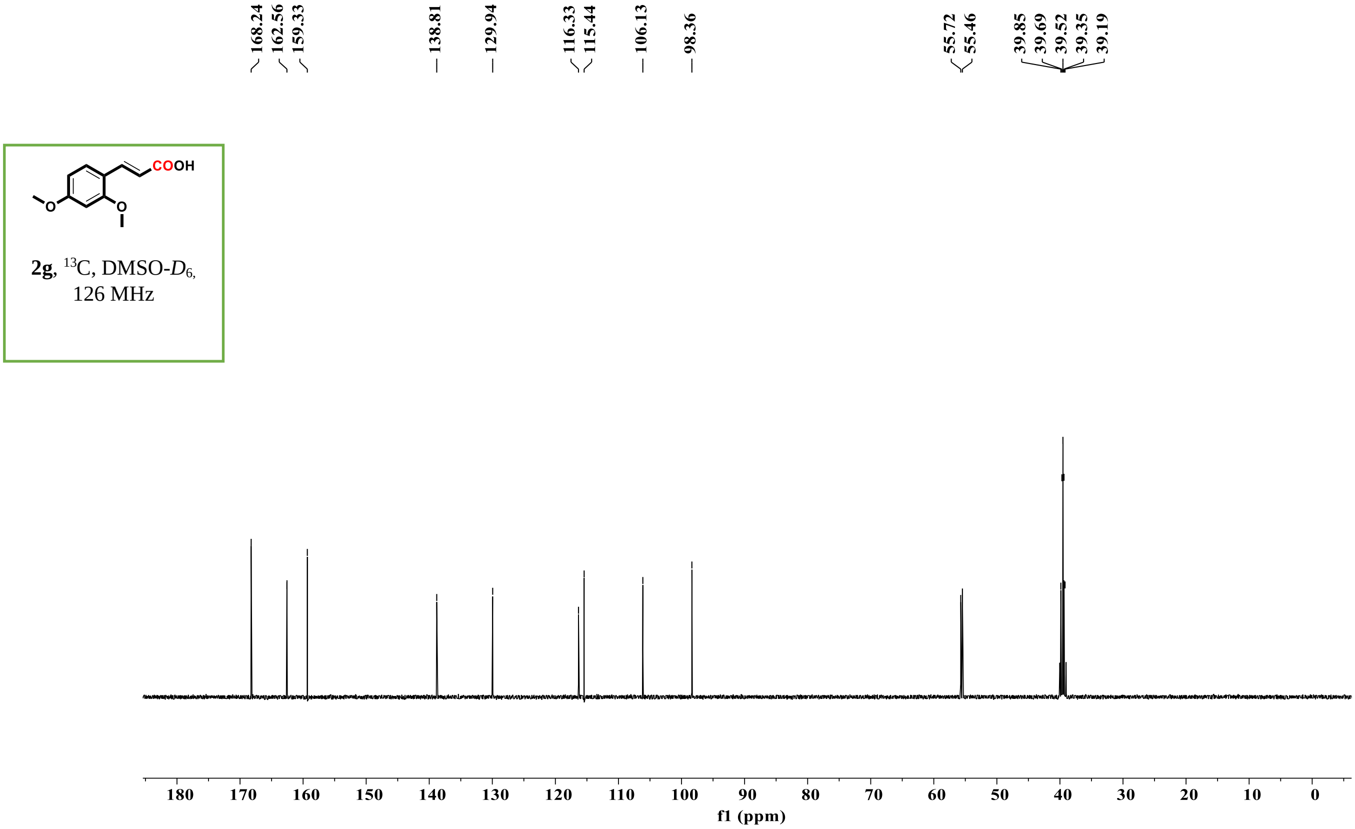

168.24
162.56
159.33
138.81
129.94
116.33
115.44
106.13
98.36
55.72
55.46
39.85
39.69
39.52
39.35
39.19
COOH
2g, ^{13}C, DMSO-D_6,
126 MHz
180 170 160 150 140 130 120 110 100 90 80 70 60 50 40 30 20 10 0
f1 (ppm)

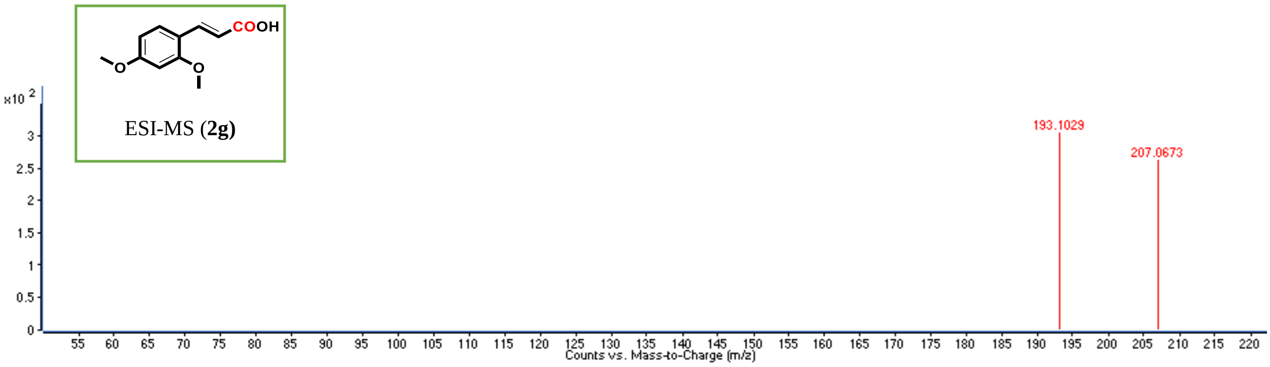

COOH
ESI-MS (2g)
x10 2
193.1029
207.0673
Counts vs. Mass-to-Charge (m/z)

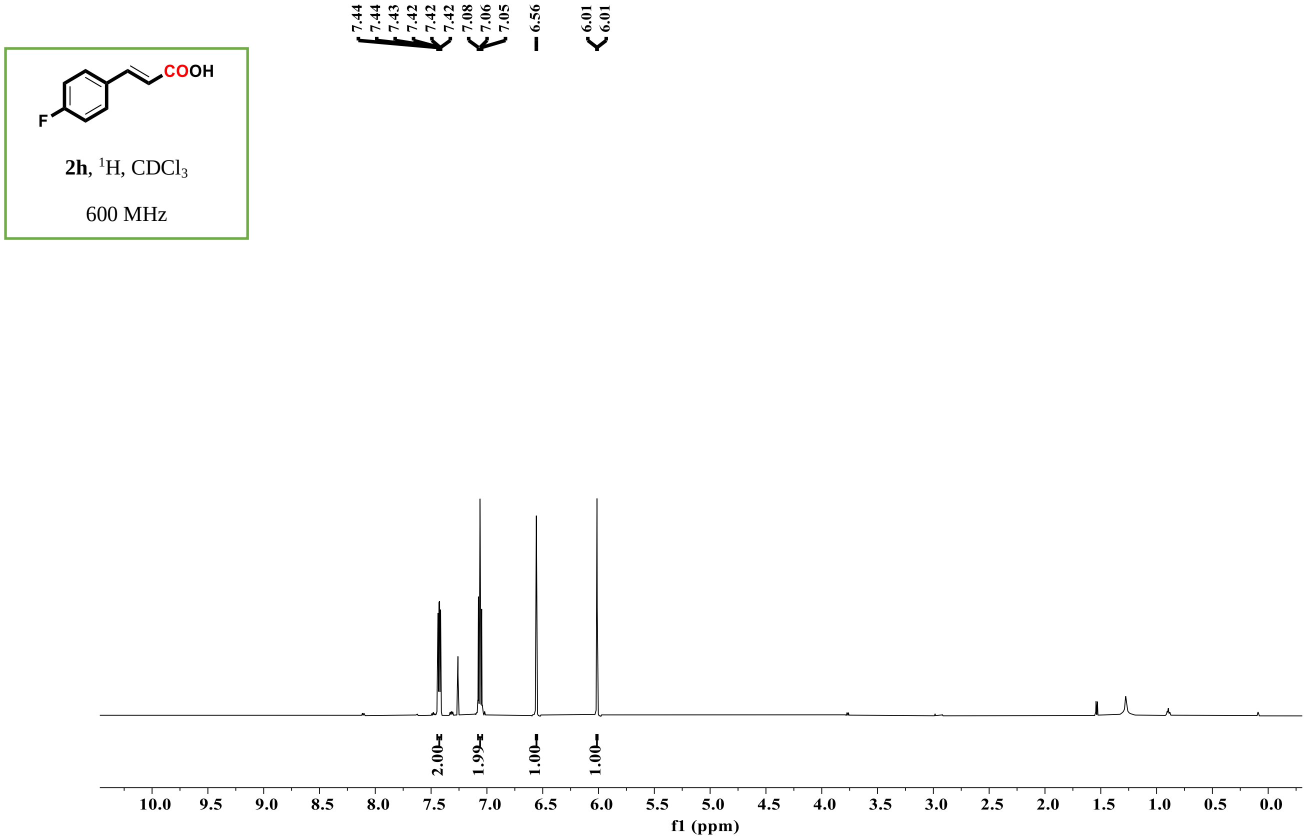
7.44
7.44
7.43
7.42
7.42
7.42
7.08
7.06
7.05
6.56
6.01
6.01
COOH
F
2h, 1H, CDCl3
600 MHz
2.00
1.99
1.00
1.00
10.0 9.5 9.0 8.5 8.0 7.5 7.0 6.5 6.0 5.5 5.0 4.5 4.0 3.5 3.0 2.5 2.0 1.5 1.0 0.5 0.0
f1 (ppm)

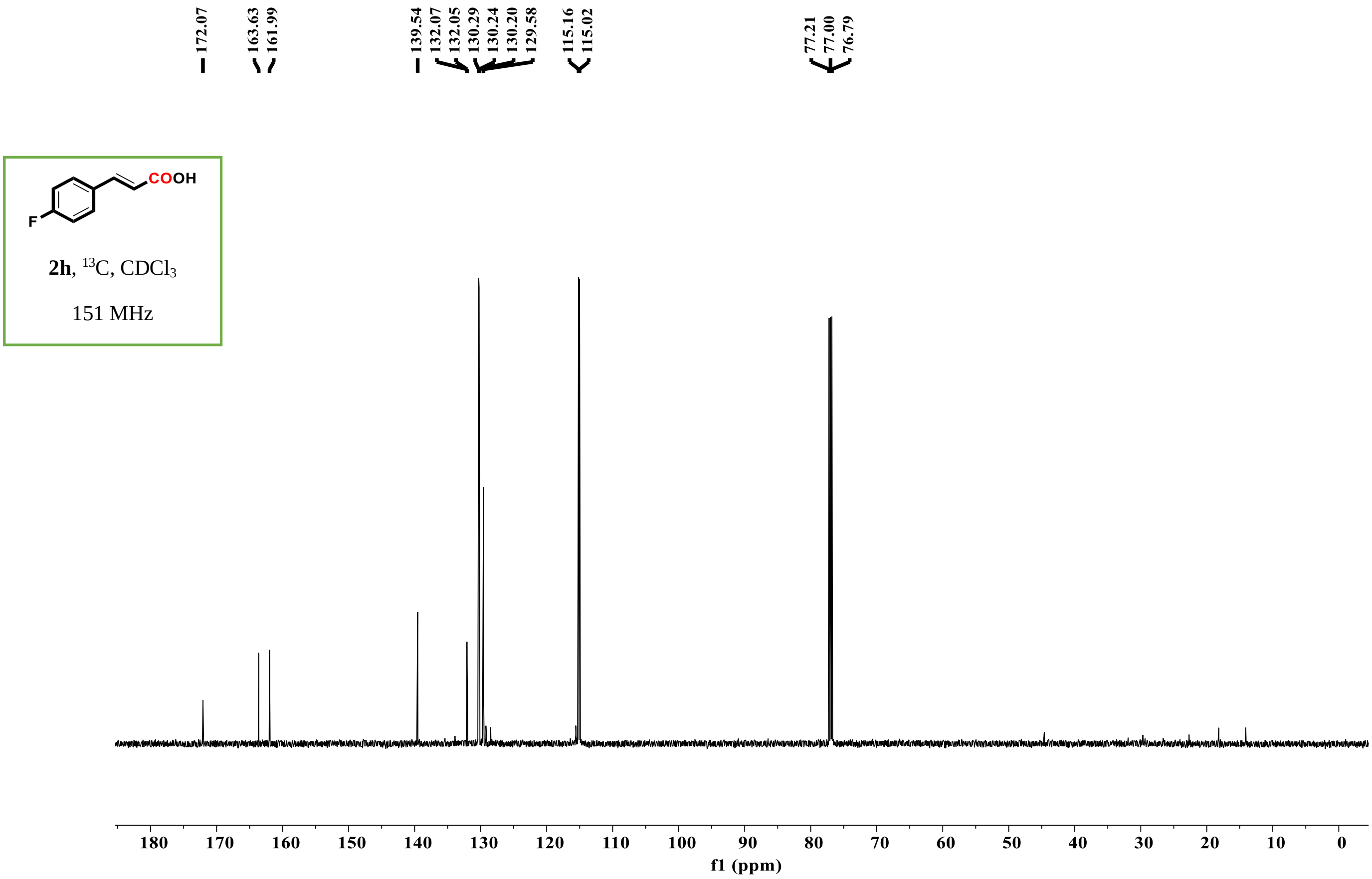
172.07
163.63
161.99
139.54
132.07
132.05
130.29
130.24
130.20
129.58
115.16
115.02
77.21
77.00
76.79
COOH
F
2h, 13C, CDCl3
151 MHz
180 170 160 150 140 130 120 110 100 90 80 70 60 50 40 30 20 10 0
f1 (ppm)

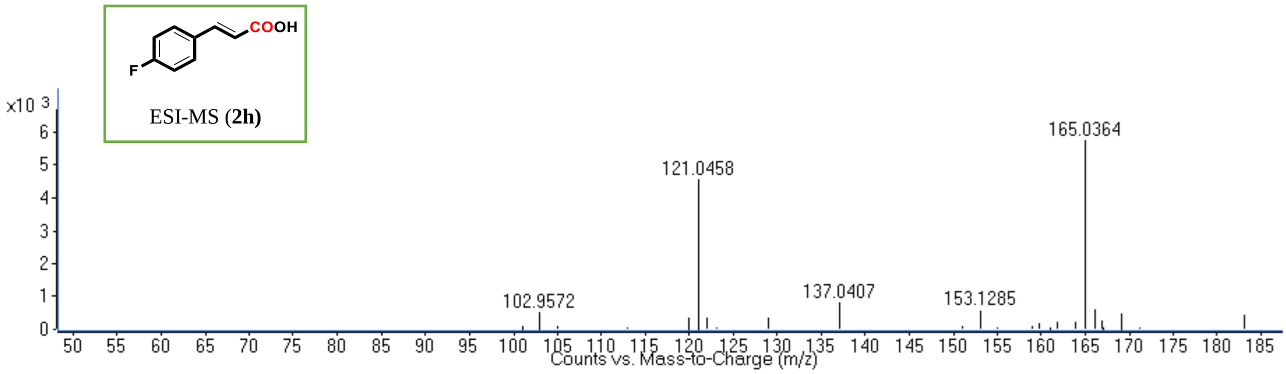
COOH
F
ESI-MS (2h)
x10 3
165.0364
121.0458
137.0407
153.1285
102.9572
Counts vs. Mass-to-Charge (m/z)

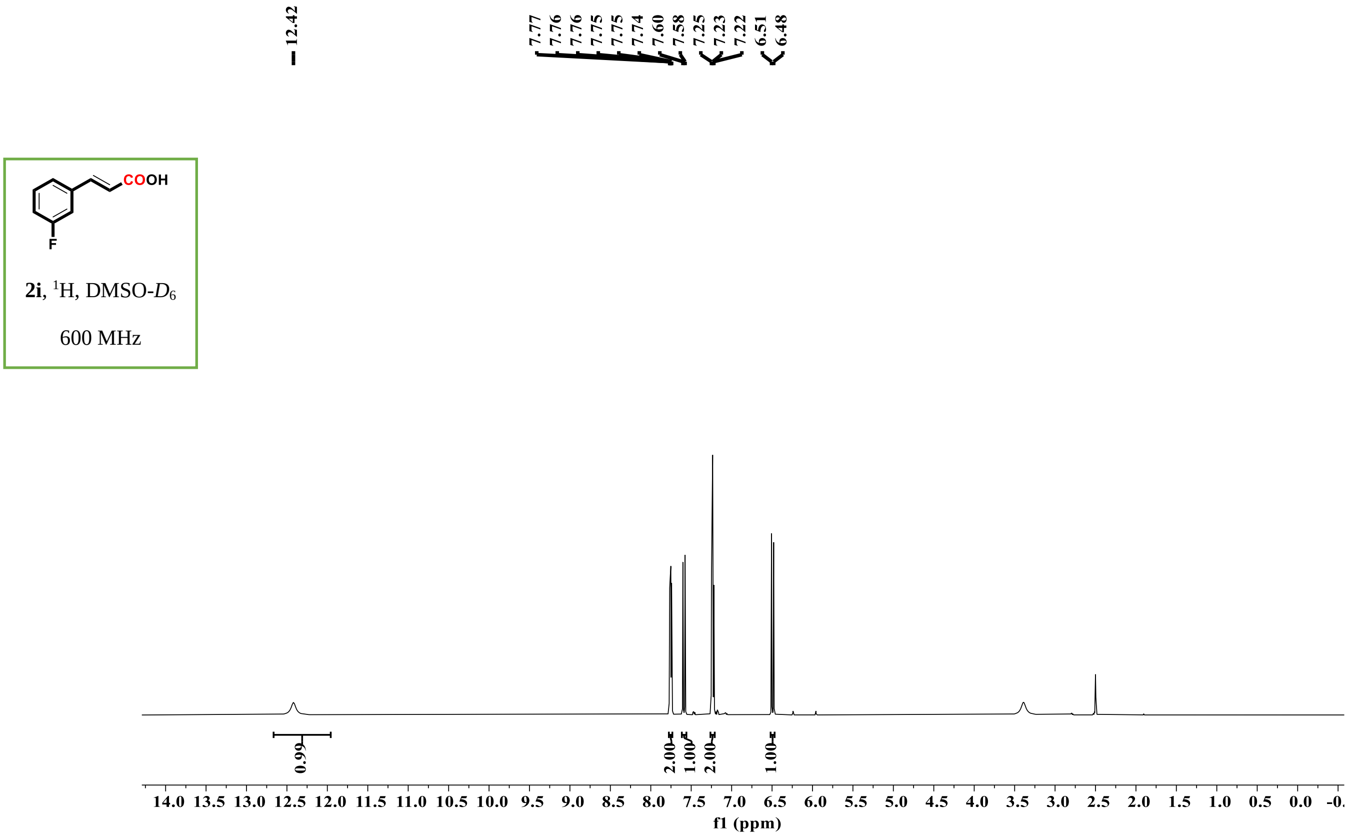
12.42
7.77
7.76
7.76
7.75
7.75
7.74
7.60
7.58
7.25
7.23
7.22
6.51
6.48
COOH
F
2i, ¹H, DMSO-D6
600 MHz
0.99
2.00
1.00
2.00
1.00
f1 (ppm)

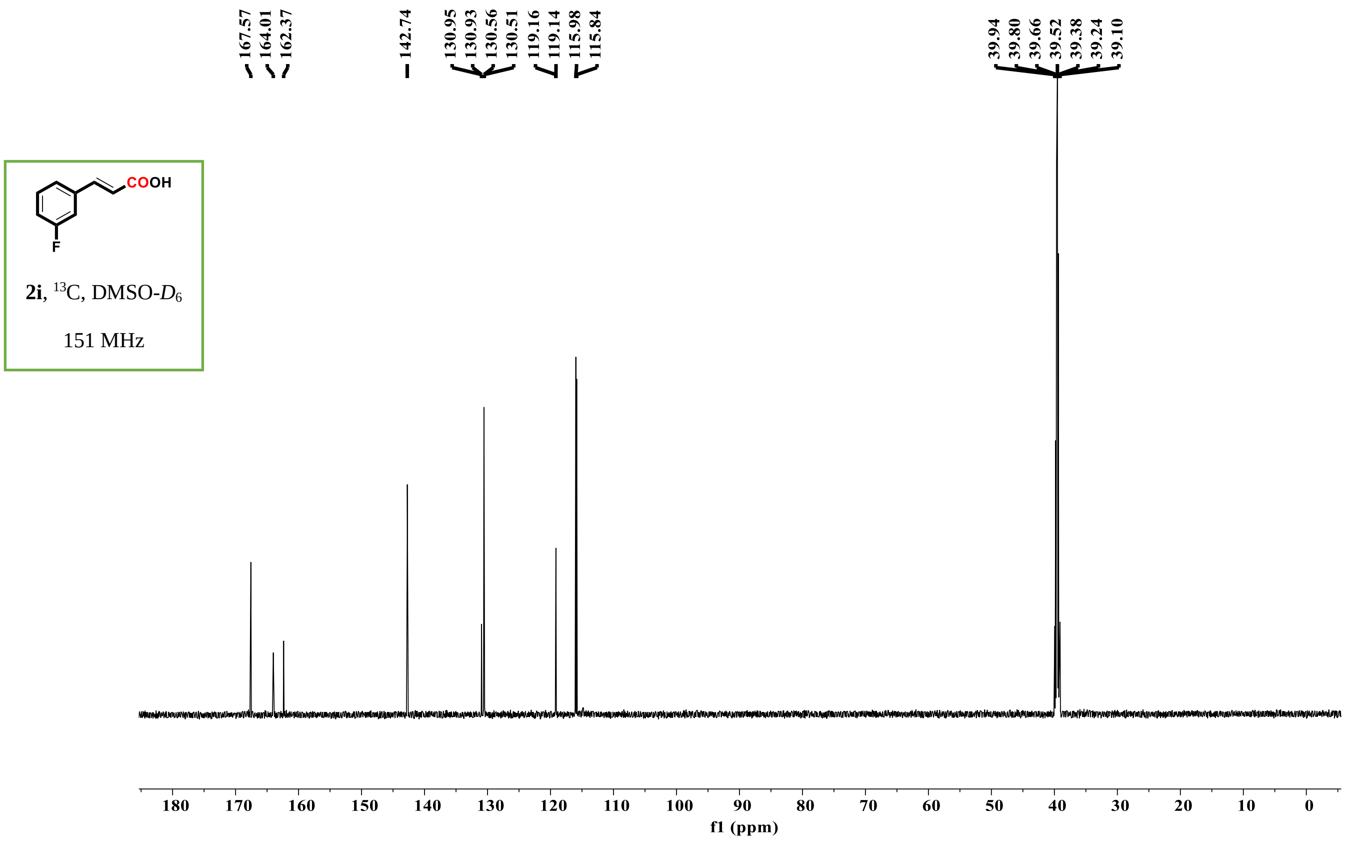
167.57
164.01
162.37
142.74
130.95
130.93
130.56
130.51
119.16
119.14
115.98
115.84
39.94
39.80
39.66
39.52
39.38
39.24
39.10
COOH
F
2i, 13C, DMSO-D6
151 MHz
180 170 160 150 140 130 120 110 100 90 80 70 60 50 40 30 20 10 0
f1 (ppm)

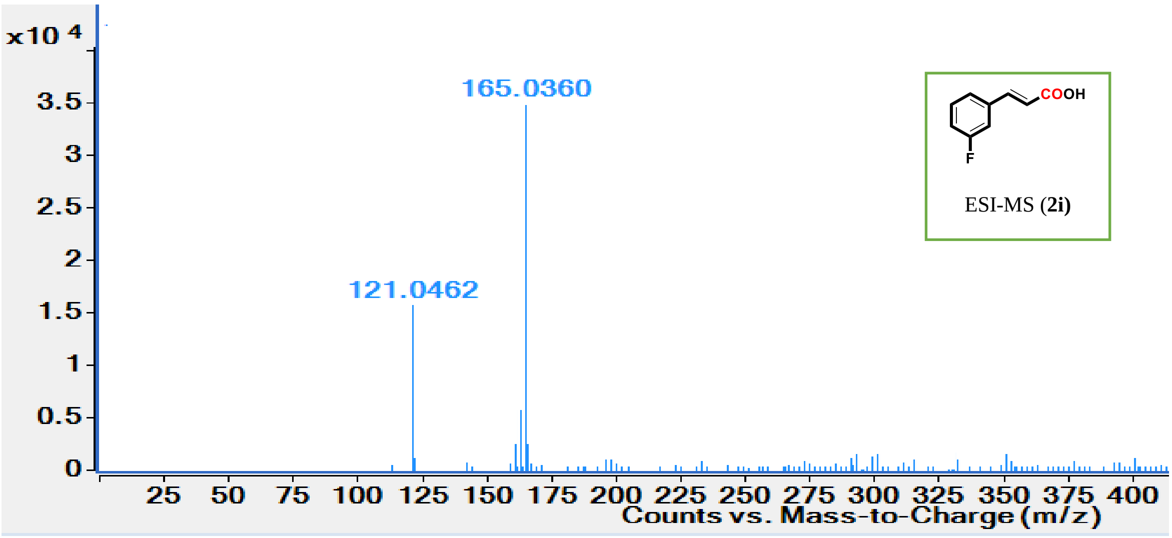
x10 4
3.5
3
2.5
2
1.5
1
0.5
0
165.0360
121.0462
25 50 75 100 125 150 175 200 225 250 275 300 325 350 375 400
Counts vs. Mass-to-Charge (m/z)
COOH
F
ESI-MS (2i)

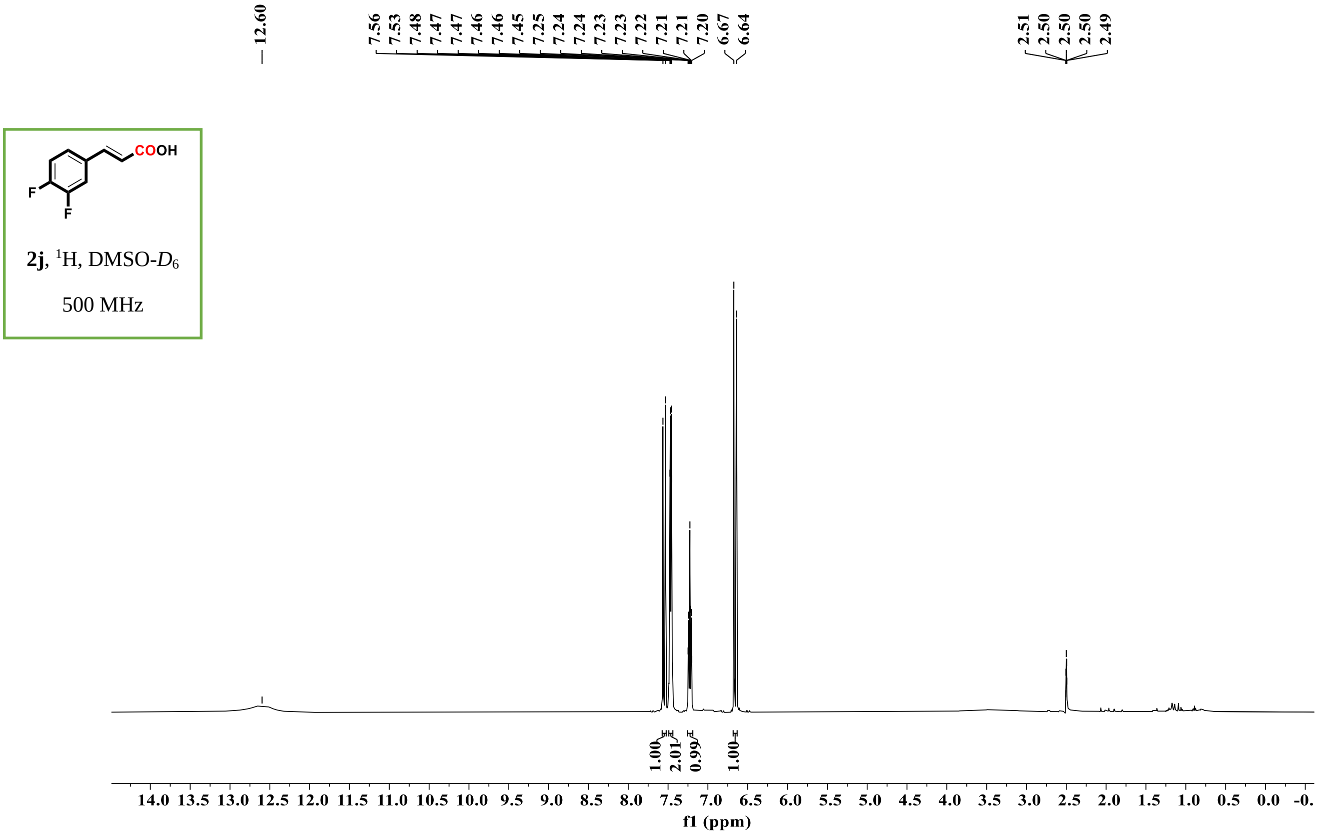

COOH
F
F
2j, 1H, DMSO-D6
500 MHz
12.60
7.56 7.53 7.48 7.47 7.46 7.46 7.45 7.25 7.24 7.24 7.23 7.23 7.22 7.21 7.21 7.20 6.67 6.64
2.51 2.50 2.50 2.50 2.49
1.00 2.01 0.99 1.00
14.0 13.5 13.0 12.5 12.0 11.5 11.0 10.5 10.0 9.5 9.0 8.5 8.0 7.5 7.0 6.5 6.0 5.5 5.0 4.5 4.0 3.5 3.0 2.5 2.0 1.5 1.0 0.5 0.0 -0.
f1 (ppm)

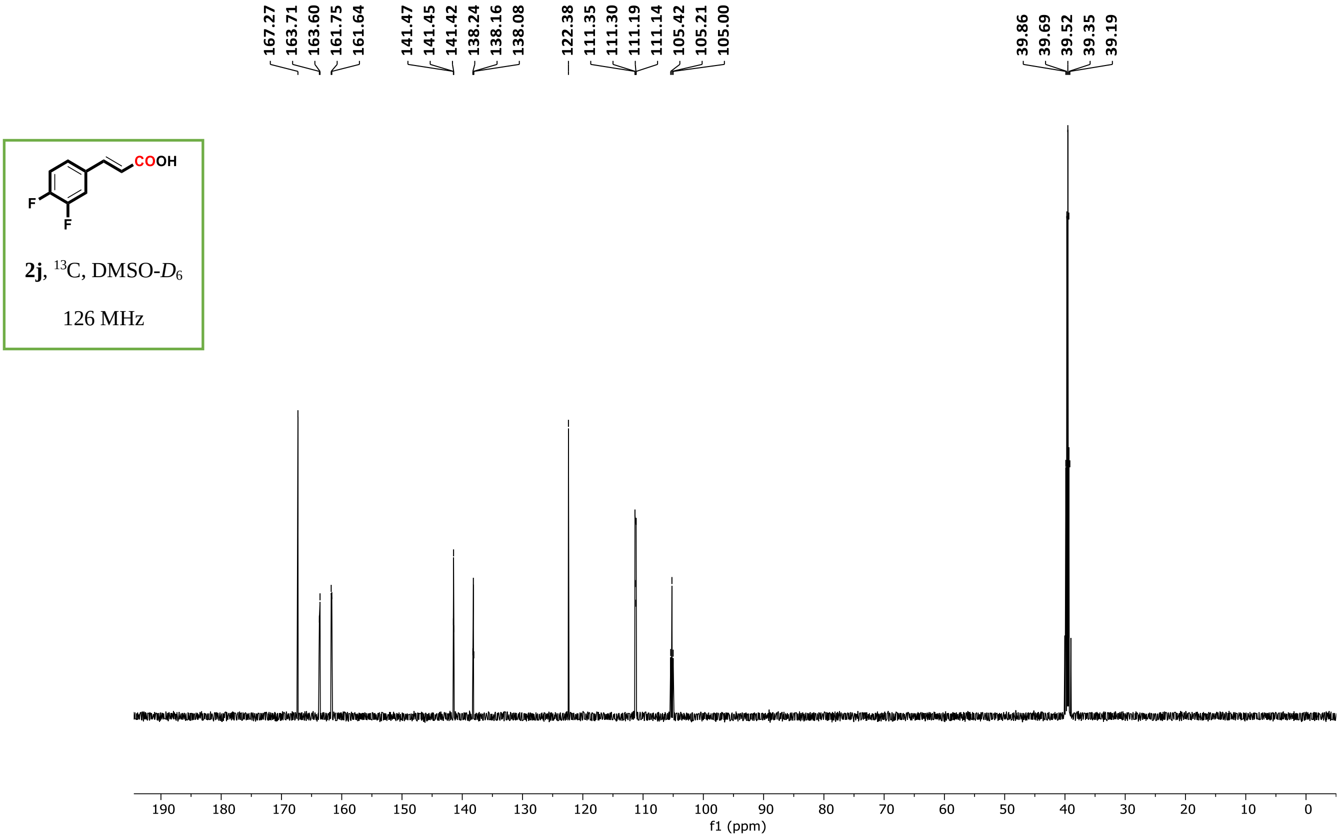

COOH
F
F
2j, 13C, DMSO-D6
126 MHz
167.27 163.71 163.60 161.75 161.64
141.47 141.45 141.42 138.24 138.16 138.08
122.38 111.35 111.30 111.19 111.14 105.42 105.21 105.00
39.86 39.69 39.52 39.35 39.19
190 180 170 160 150 140 130 120 110 100 90 80 70 60 50 40 30 20 10 0
f1 (ppm)

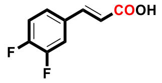

2j, ^{19}F, DMSO-D_6

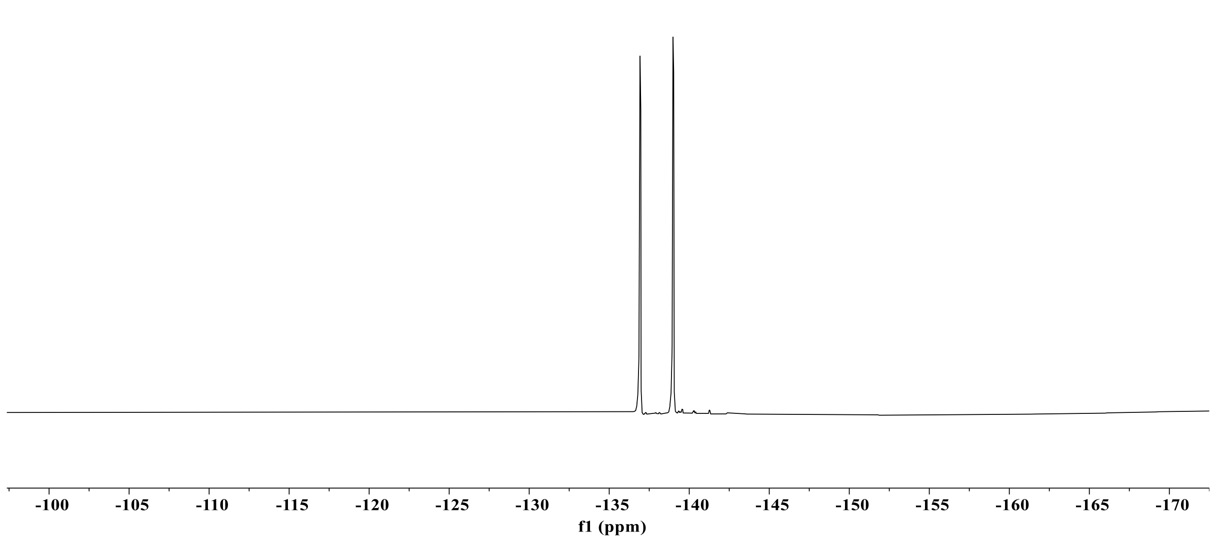

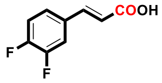

ESI-MS (**2j**)

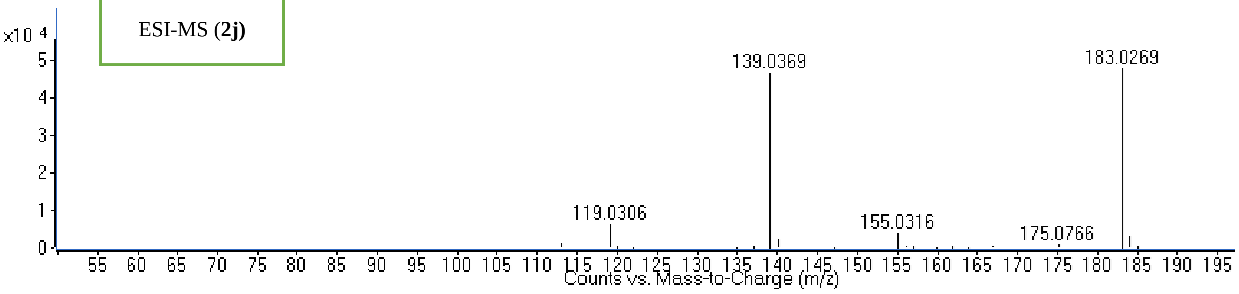

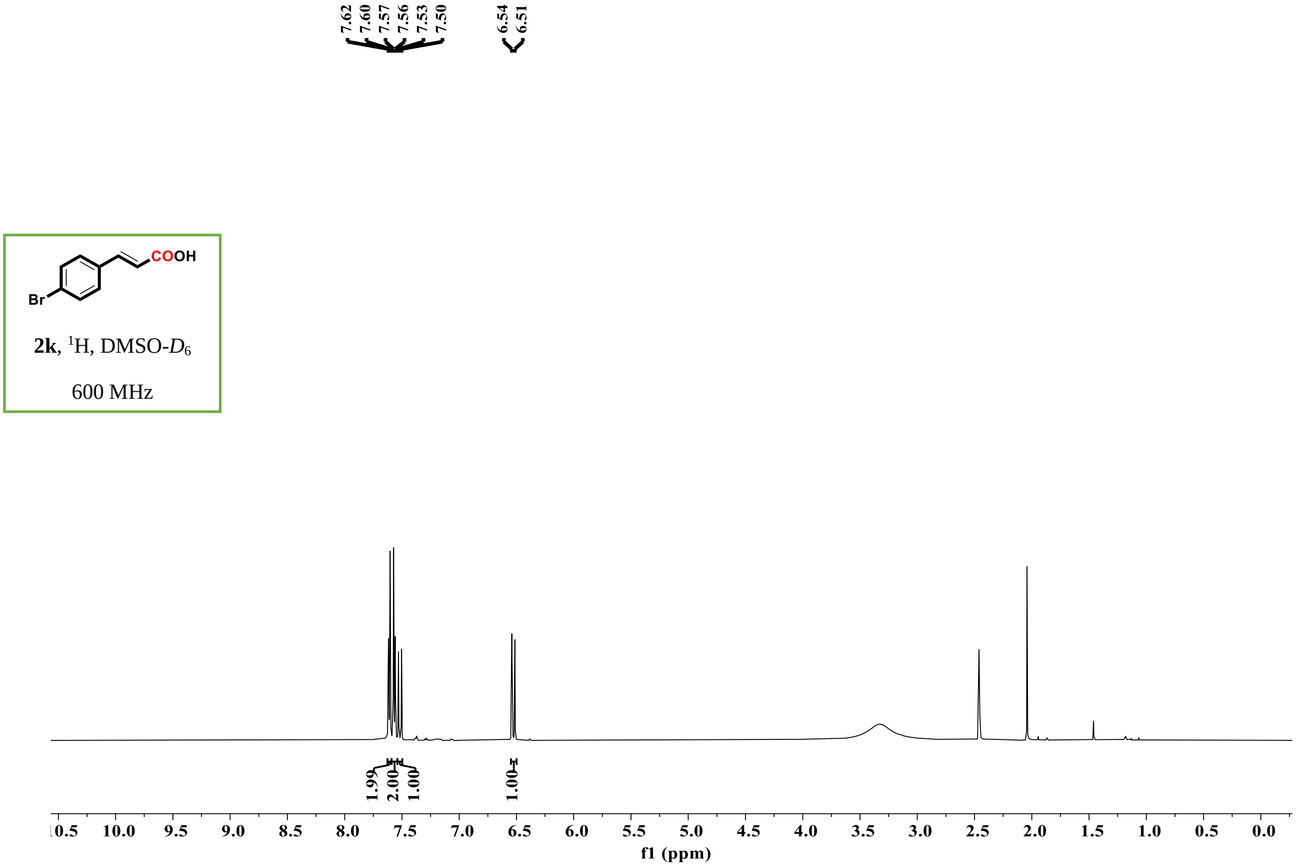
7.62
7.60
7.57
7.56
7.53
7.50
6.54
6.51
COOH
Br
2k, ¹H, DMSO-D6
600 MHz
1.99
2.00
1.00
1.00
f1 (ppm)

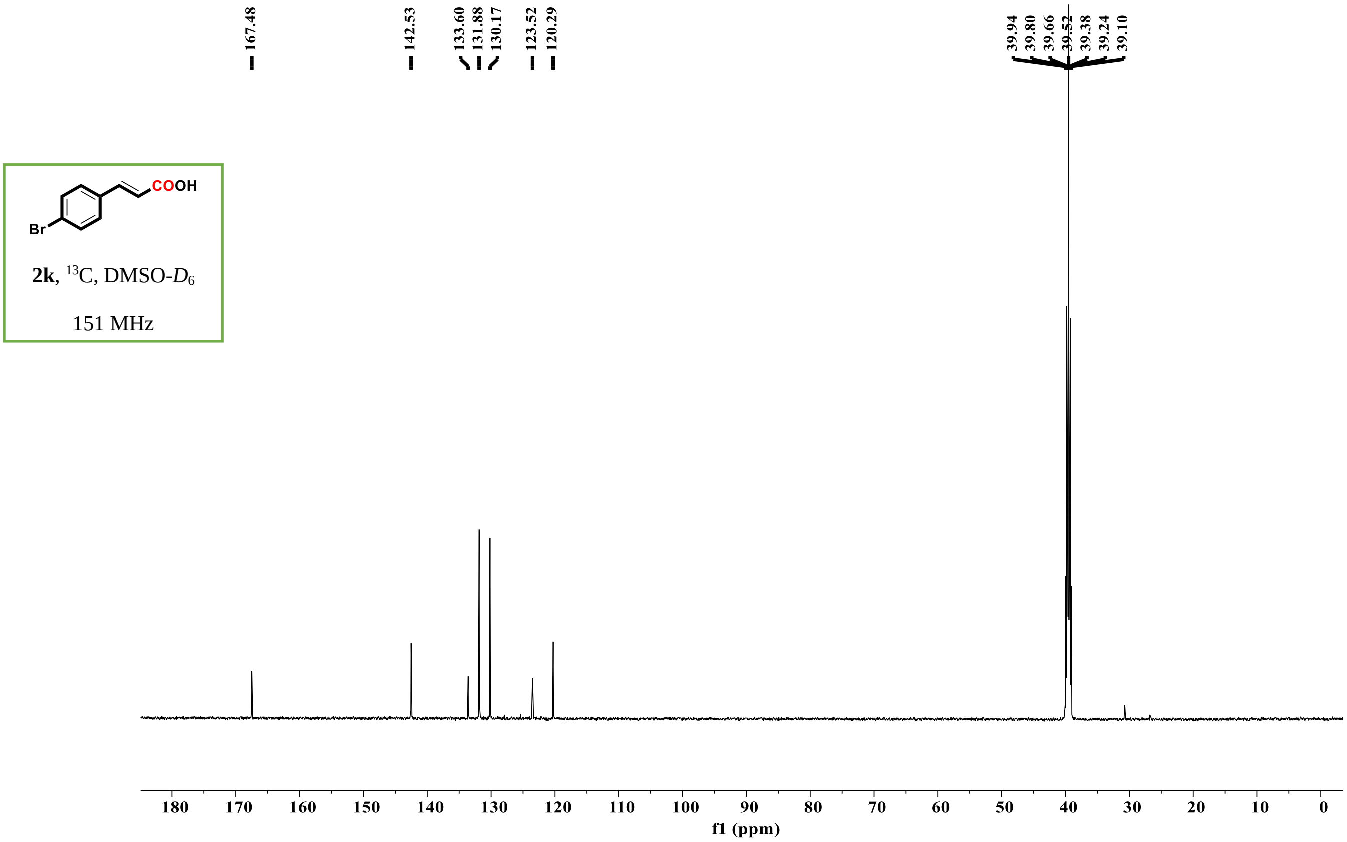
167.48
142.53
133.60
131.88
130.17
123.52
120.29
39.94
39.80
39.66
39.52
39.38
39.24
39.10
COOH
Br
2k, ¹³C, DMSO-D6
151 MHz
f1 (ppm)

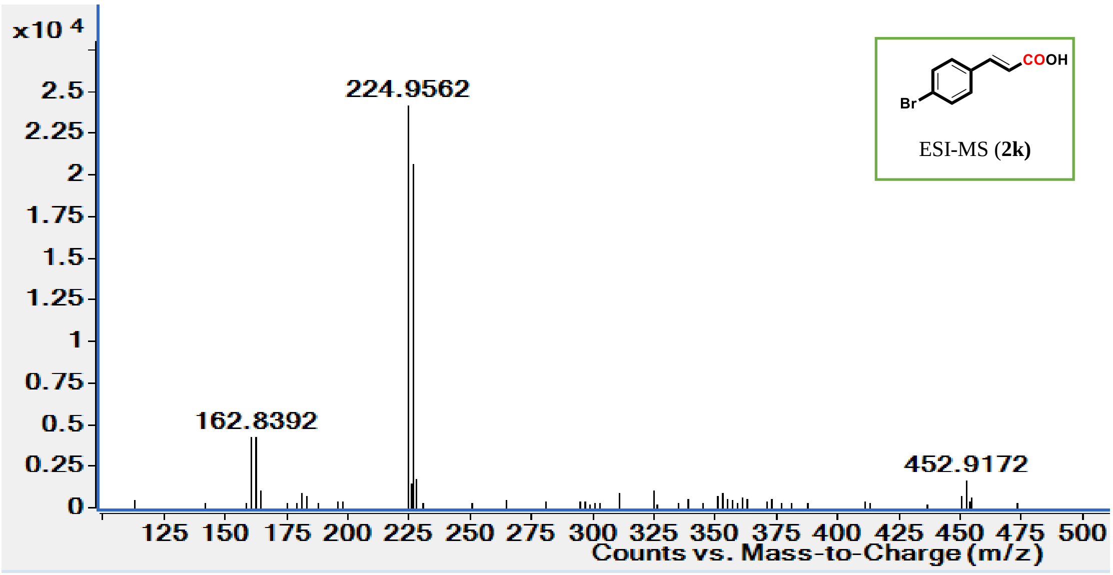

x10 4
2.5
2.25
2
1.75
1.5
1.25
1
0.75
0.5
0.25
0
224.9562
162.8392
452.9172
125 150 175 200 225 250 275 300 325 350 375 400 425 450 475 500
Counts vs. Mass-to-Charge (m/z)
COOH
Br
ESI-MS (**2k**)

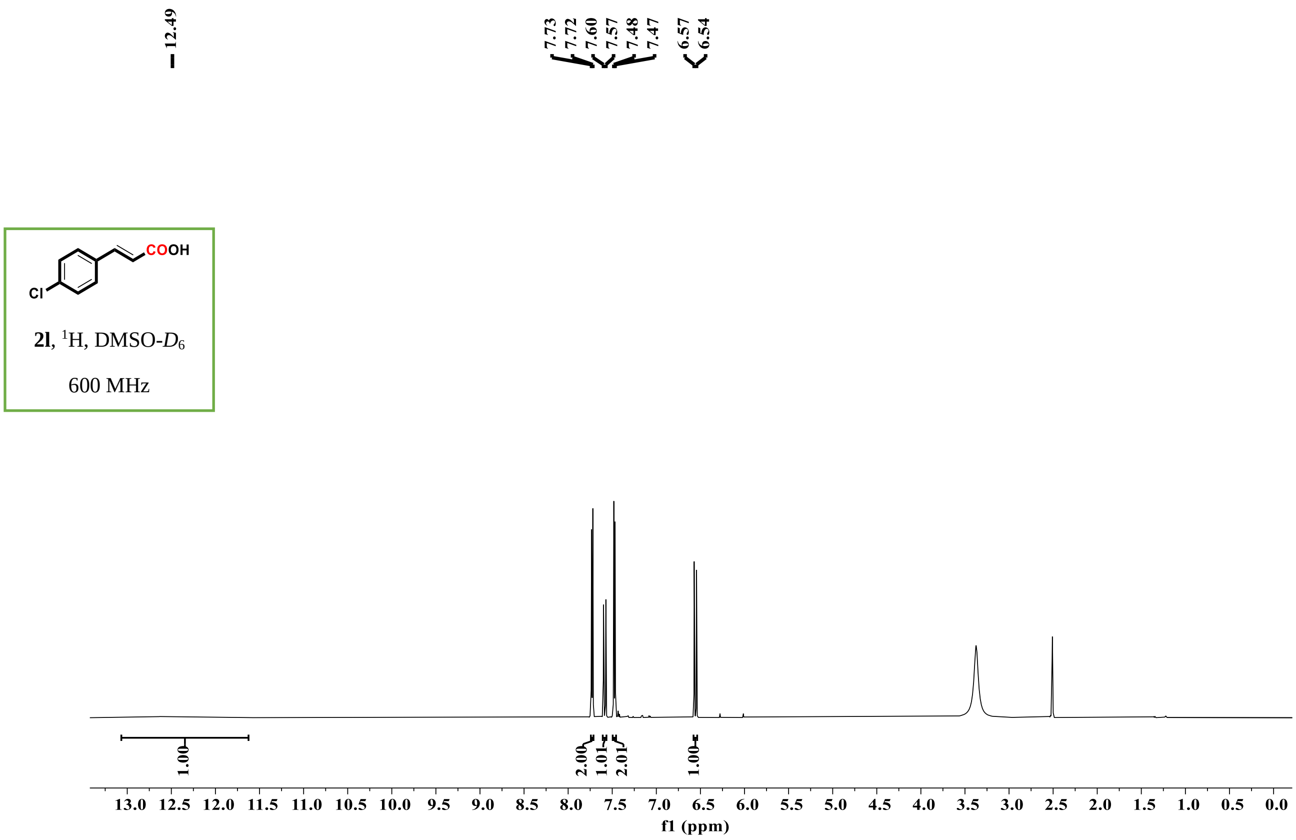

12.49
7.73
7.72
7.60
7.57
7.48
7.47
6.57
6.54
COOH
Cl
2l, 1H, DMSO-D6
600 MHz
1.00
2.00
1.01
2.01
1.00
13.0 12.5 12.0 11.5 11.0 10.5 10.0 9.5 9.0 8.5 8.0 7.5 7.0 6.5 6.0 5.5 5.0 4.5 4.0 3.5 3.0 2.5 2.0 1.5 1.0 0.5 0.0
f1 (ppm)

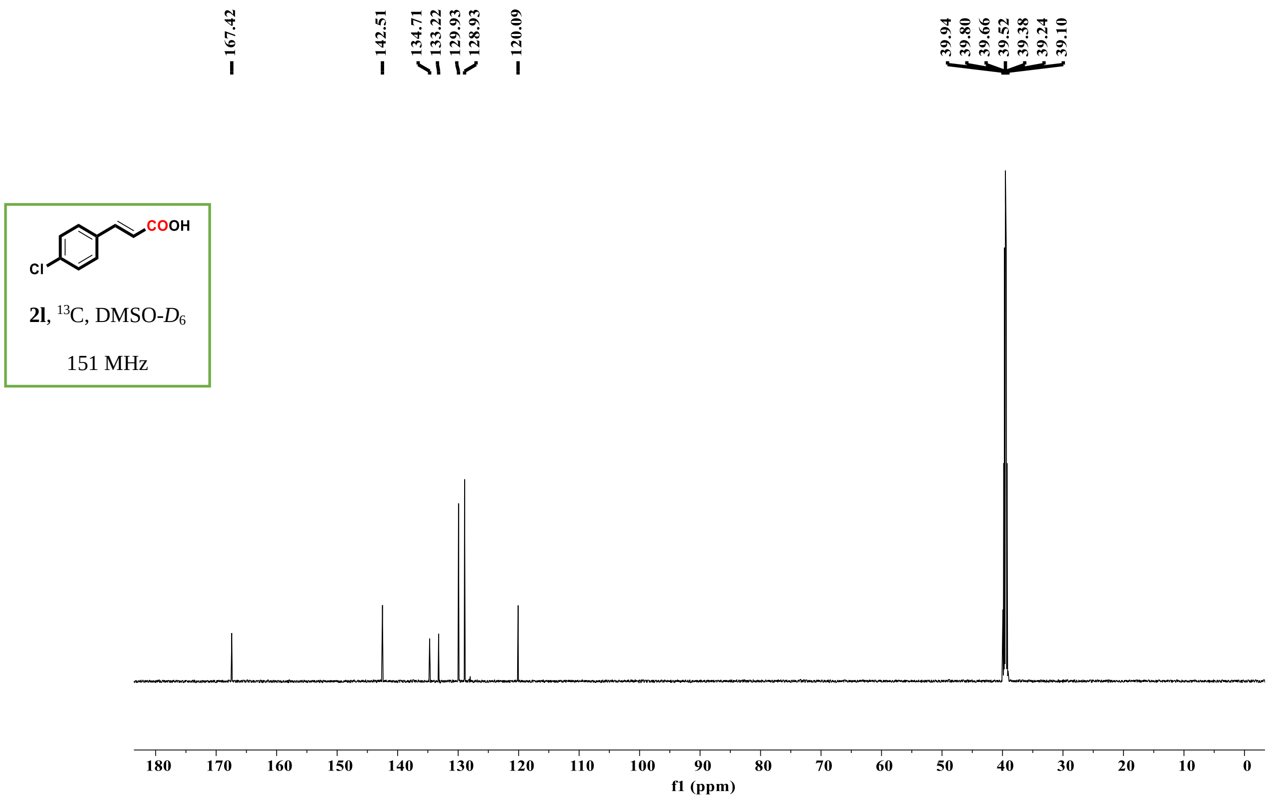
167.42
142.51
134.71
133.22
129.93
128.93
120.09
39.94
39.80
39.66
39.52
39.38
39.24
39.10
COOH
Cl
2l, 13C, DMSO-D6
151 MHz
f1 (ppm)

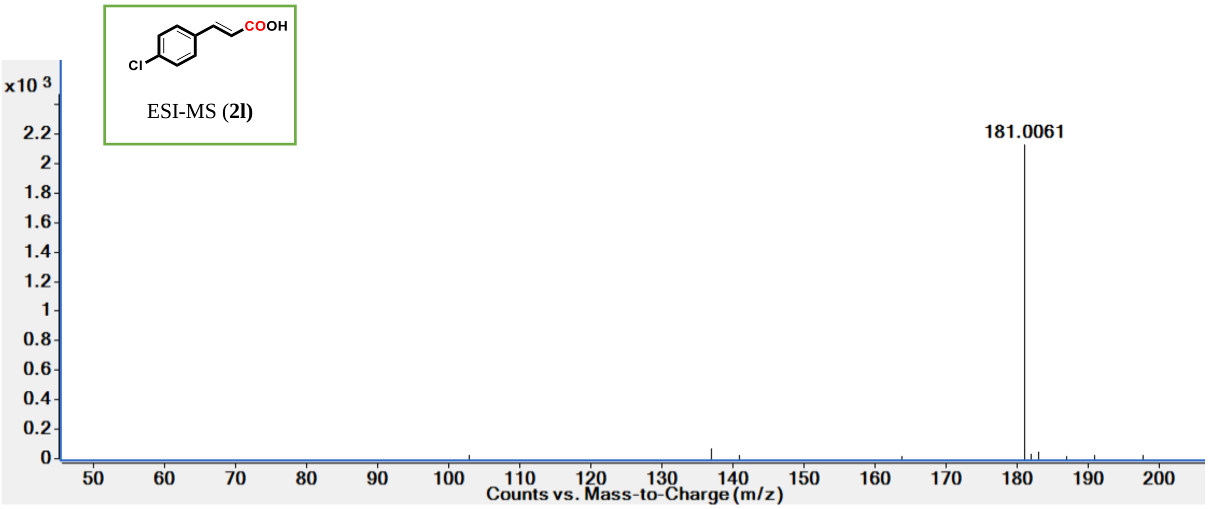
COOH
Cl
ESI-MS (2l)
x10 3
181.0061
Counts vs. Mass-to-Charge (m/z)

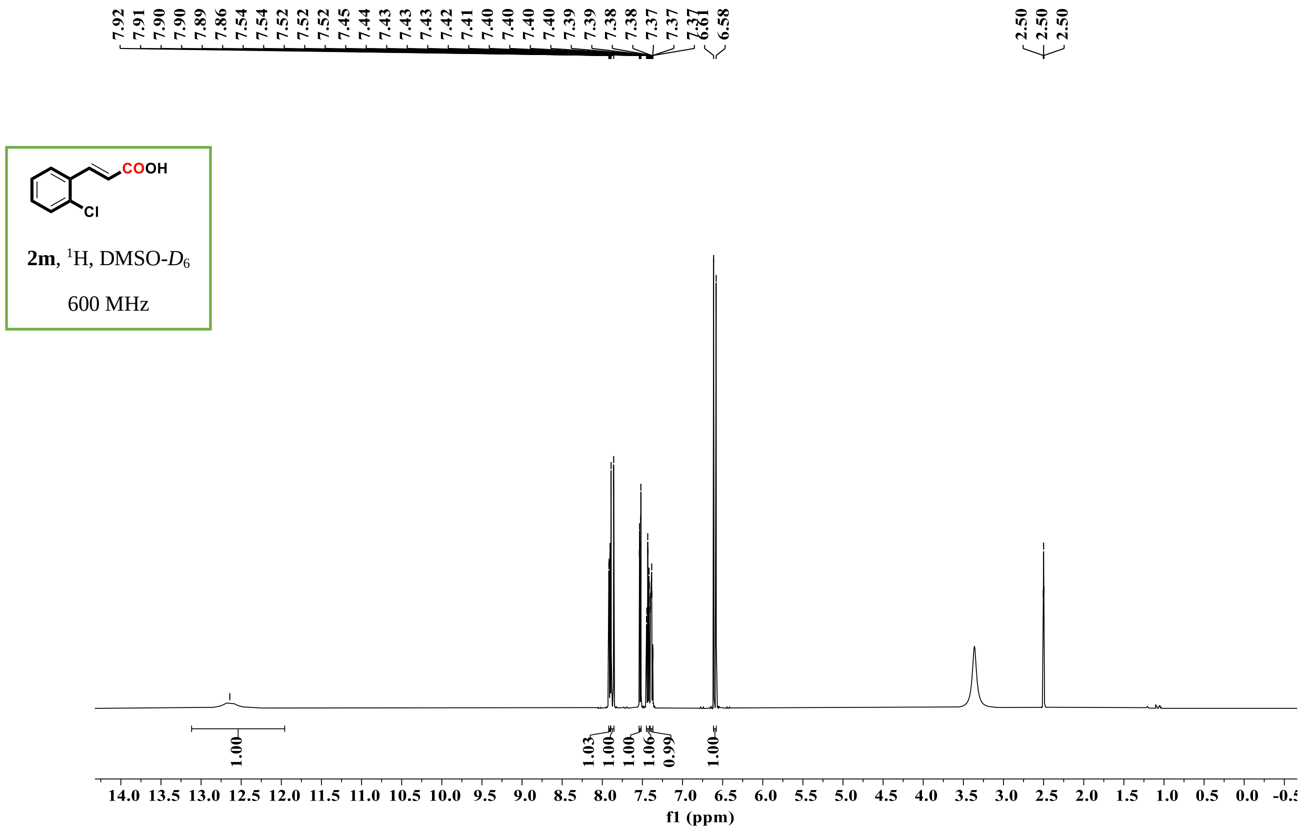
7.92 7.91 7.90 7.90 7.89 7.86 7.54 7.54 7.52 7.52 7.52 7.45 7.44 7.43 7.43 7.43 7.42 7.41 7.40 7.40 7.40 7.40 7.39 7.39 7.38 7.38 7.37 7.37 7.37 6.61 6.58
2.50 2.50 2.50
COOH
Cl
2m, 1H, DMSO-D6
600 MHz
1.00
1.03 1.00 1.00 1.06 0.99
1.00
14.0 13.5 13.0 12.5 12.0 11.5 11.0 10.5 10.0 9.5 9.0 8.5 8.0 7.5 7.0 6.5 6.0 5.5 5.0 4.5 4.0 3.5 3.0 2.5 2.0 1.5 1.0 0.5 0.0
f1 (ppm)

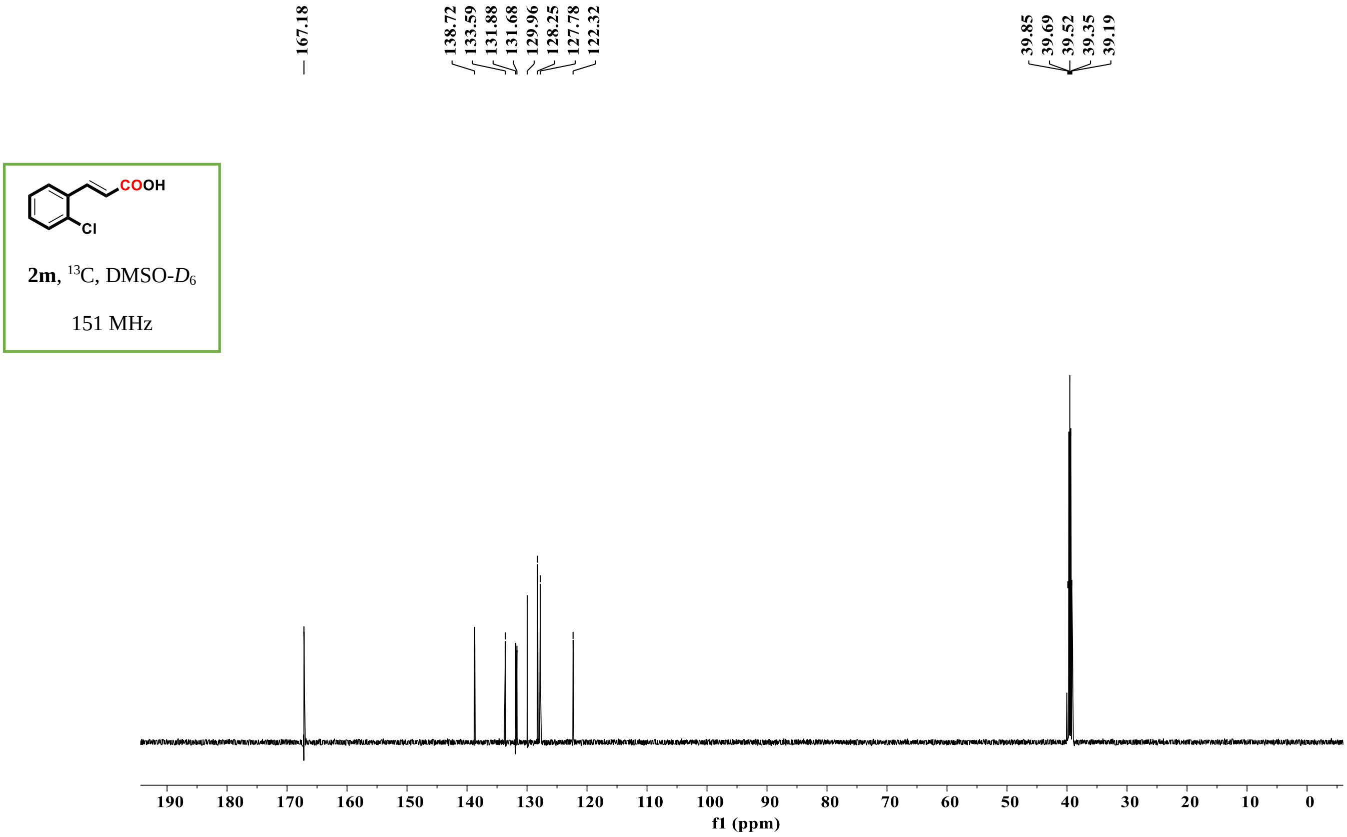
167.18
138.72 133.59 131.88 131.68 129.96 128.25 127.78 122.32
39.85 39.69 39.52 39.35 39.19
COOH
Cl
2m, 13C, DMSO-D6
151 MHz
190 180 170 160 150 140 130 120 110 100 90 80 70 60 50 40 30 20 10 0
f1 (ppm)

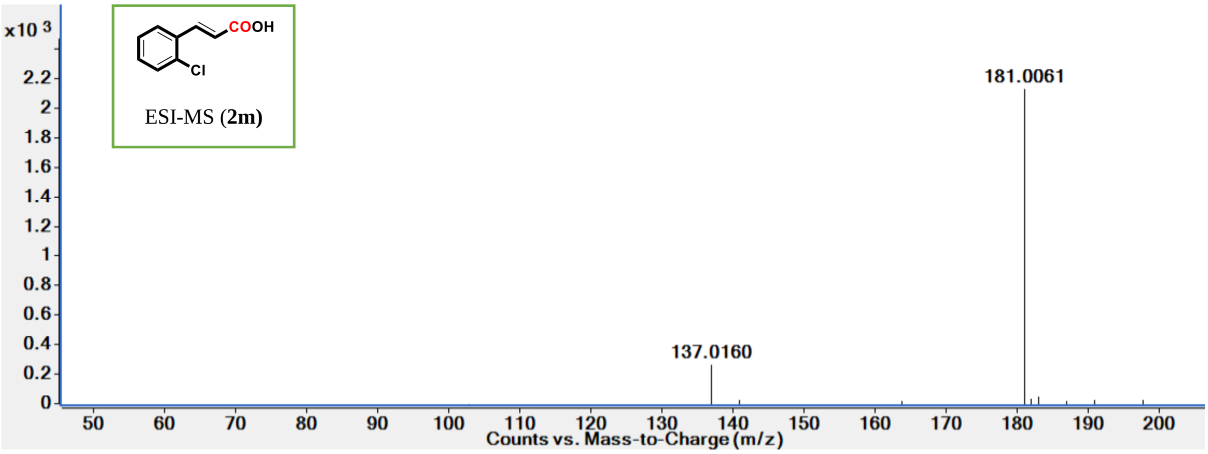

COOH
Cl
ESI-MS (2m)
x10 3
181.0061
137.0160
Counts vs. Mass-to-Charge (m/z)

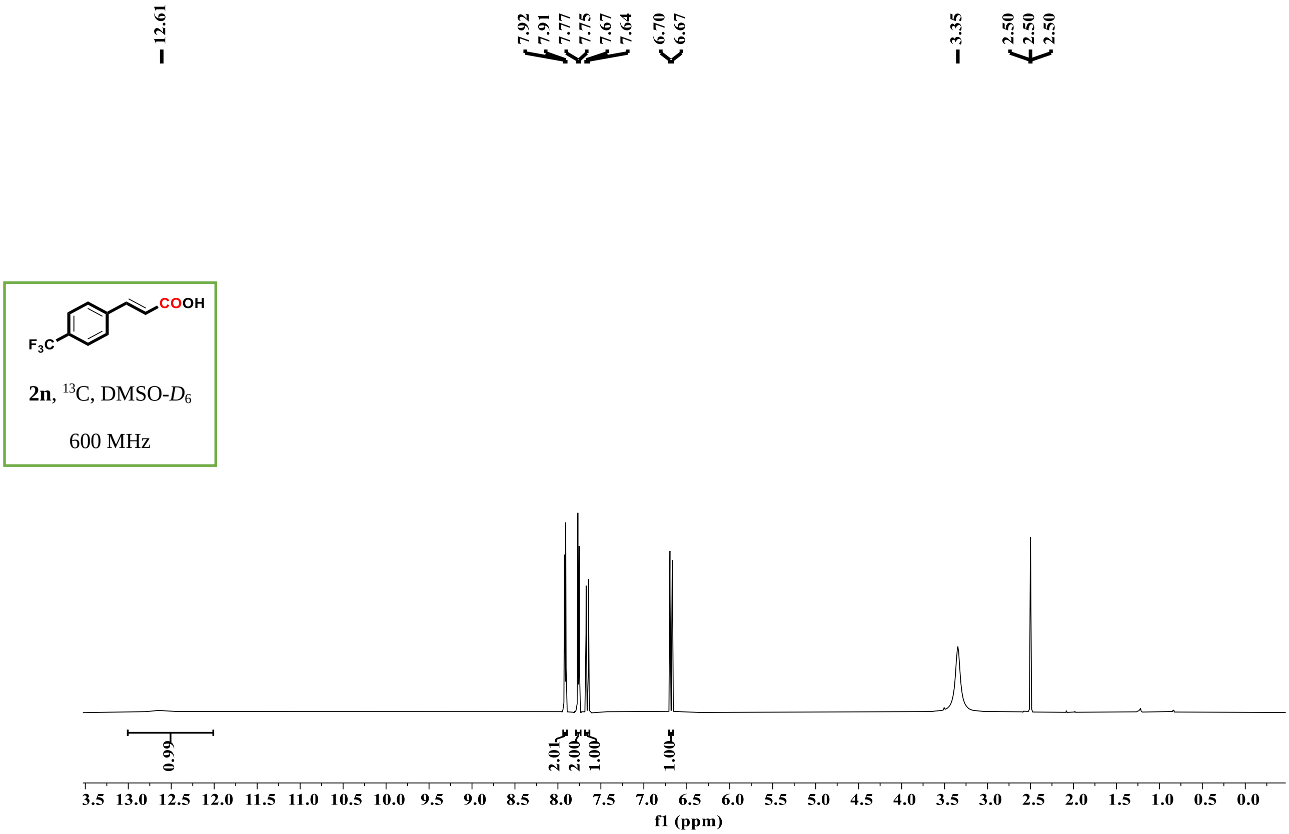

12.61
7.92
7.91
7.77
7.75
7.67
7.64
6.70
6.67
3.35
2.50
2.50
2.50
COOH
F3C
2n, 13C, DMSO-D6
600 MHz
0.99
2.01
2.00
1.00
1.00
f1 (ppm)

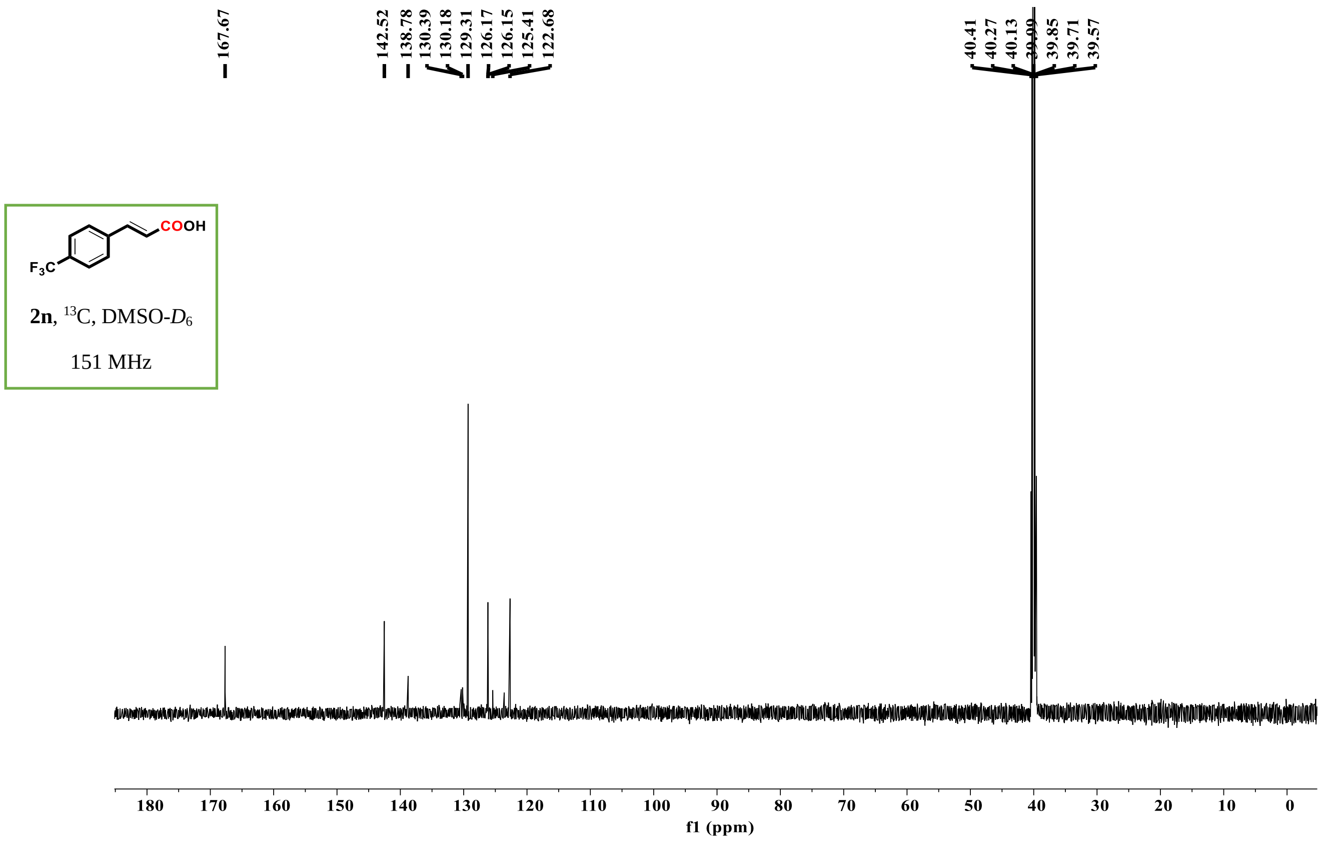
167.67
142.52
138.78
130.39
130.18
129.31
126.17
126.15
125.41
122.68
40.41
40.27
40.13
39.99
39.85
39.71
39.57
COOH
F3C
2n, 13C, DMSO-D6
151 MHz
f1 (ppm)

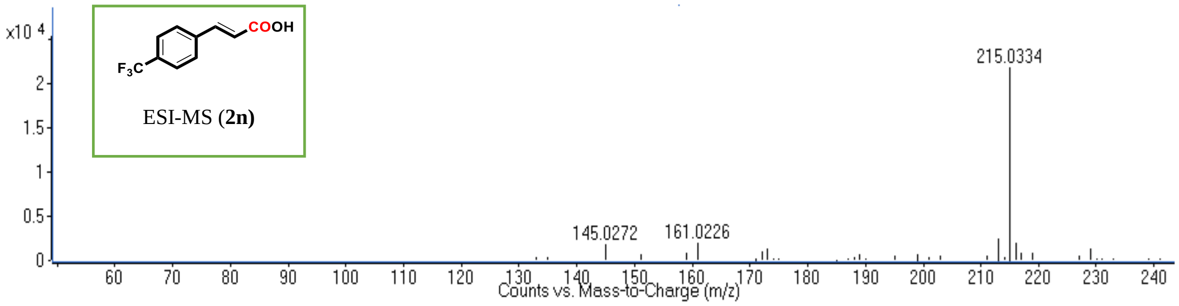
x10 4
COOH
F3C
ESI-MS (2n)
215.0334
145.0272
161.0226
Counts vs. Mass-to-Charge (m/z)

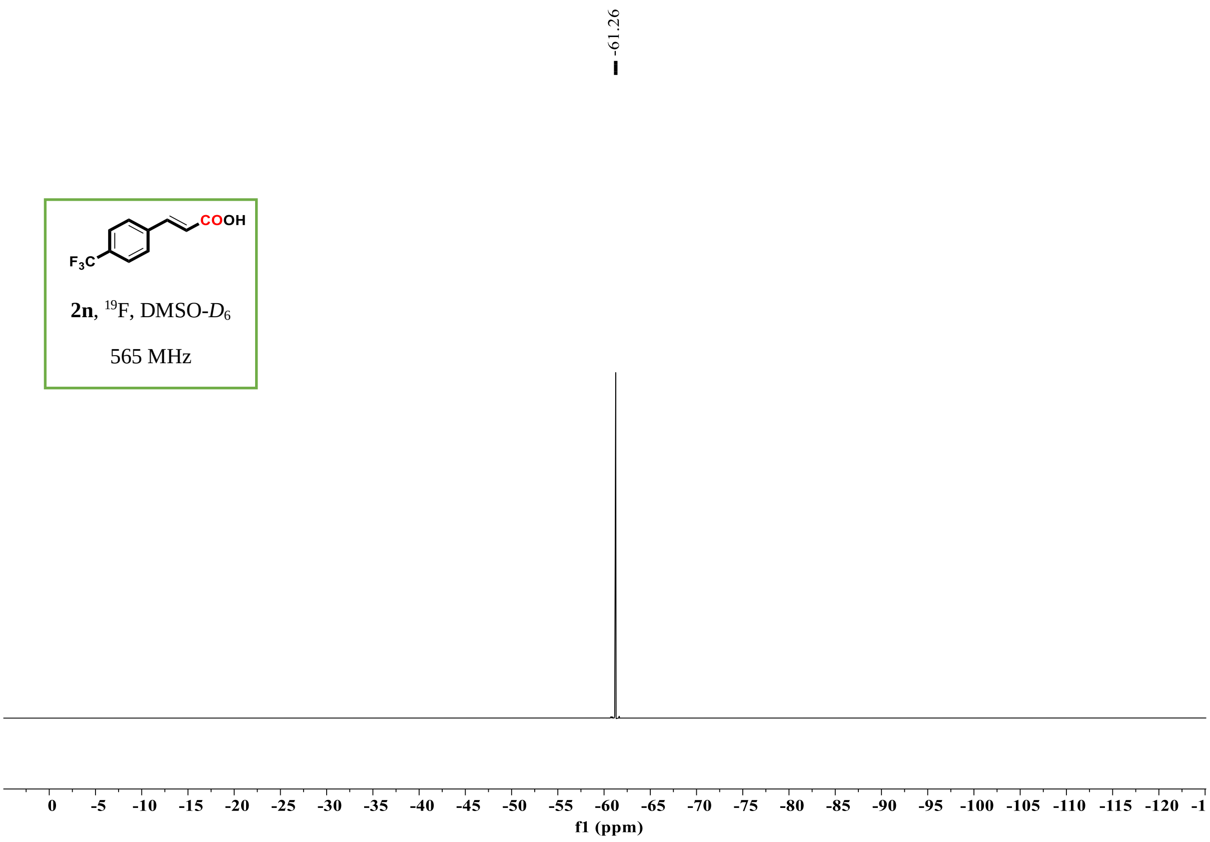
-61.26
COOH
F3C
2n, 19F, DMSO-D6
565 MHz
0 -5 -10 -15 -20 -25 -30 -35 -40 -45 -50 -55 -60 -65 -70 -75 -80 -85 -90 -95 -100 -105 -110 -115 -120 -1
f1 (ppm)

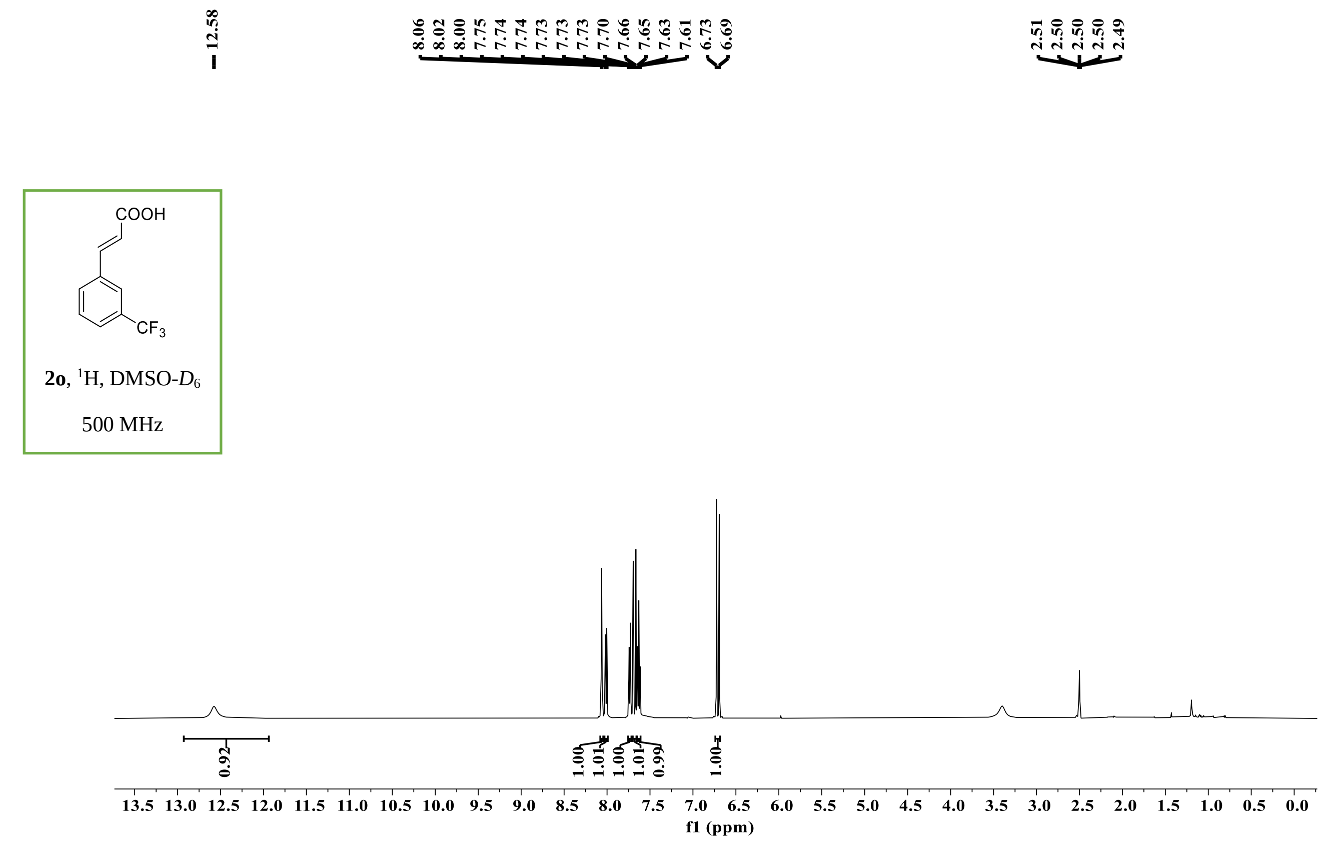
12.58
8.06 8.02 8.00 7.75 7.74 7.74 7.73 7.73 7.73 7.70 7.66 7.65 7.63 7.61 6.73 6.69
2.51 2.50 2.50 2.50 2.49
COOH
CF3
2o, 1H, DMSO-D6
500 MHz
0.92 1.00 1.01 1.00 1.01 0.99 1.00
13.5 13.0 12.5 12.0 11.5 11.0 10.5 10.0 9.5 9.0 8.5 8.0 7.5 7.0 6.5 6.0 5.5 5.0 4.5 4.0 3.5 3.0 2.5 2.0 1.5 1.0 0.5 0.0
f1 (ppm)

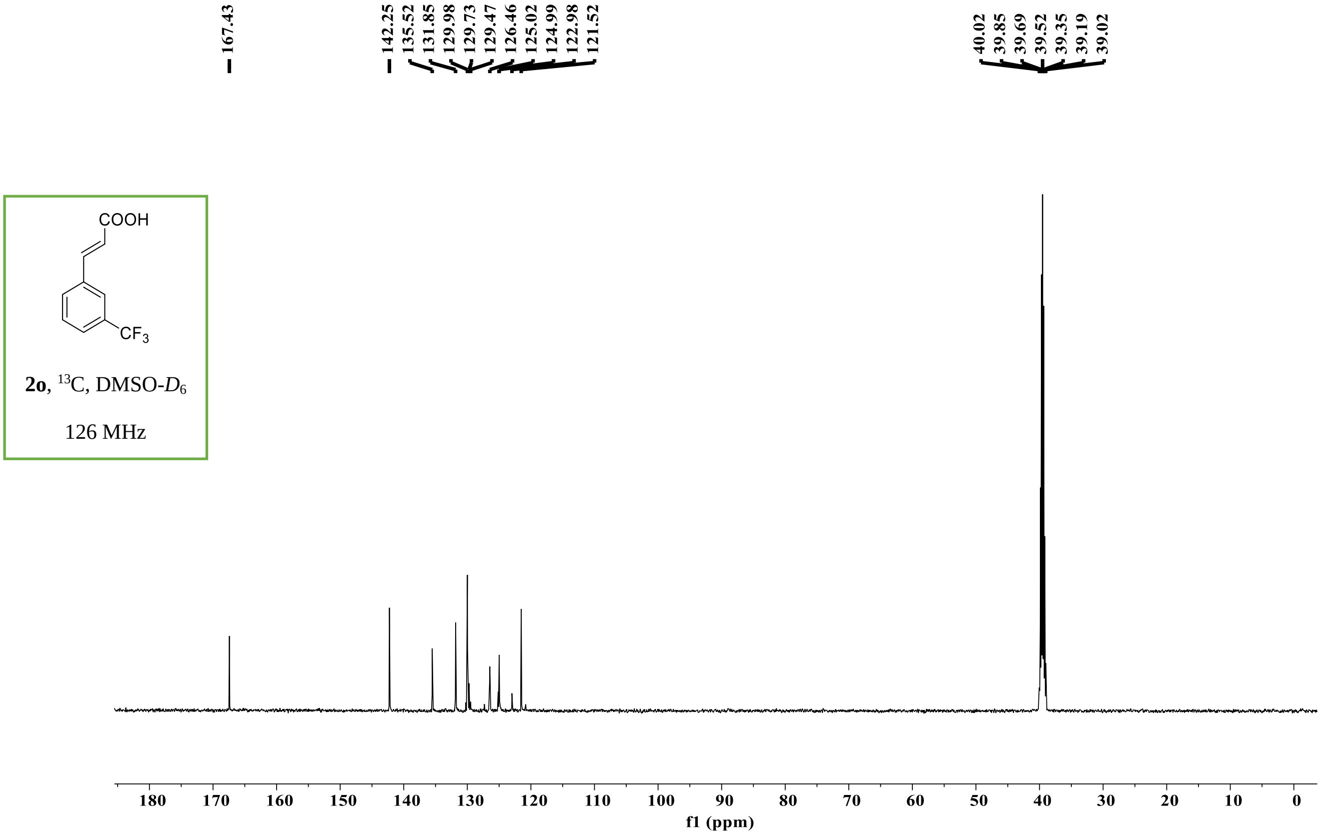
167.43
142.25 135.52 131.85 129.98 129.73 129.47 126.46 125.02 124.99 122.98 121.52
40.02 39.85 39.69 39.52 39.35 39.19 39.02
COOH
CF3
2o, 13C, DMSO-D6
126 MHz
180 170 160 150 140 130 120 110 100 90 80 70 60 50 40 30 20 10 0
f1 (ppm)

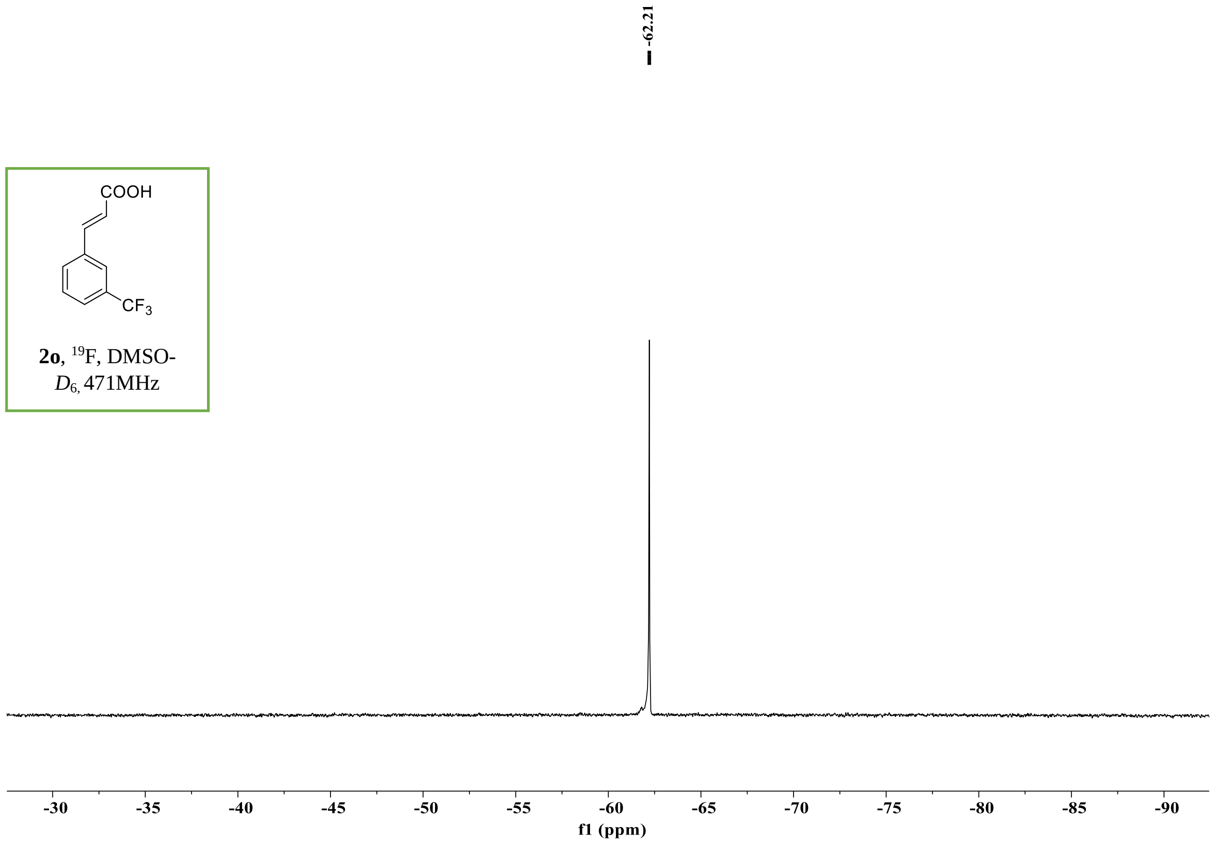

-62.21
COOH
CF3
2o, 19F, DMSO-D6, 471MHz
-30
-35
-40
-45
-50
-55
-60
-65
-70
-75
-80
-85
-90
f1 (ppm)

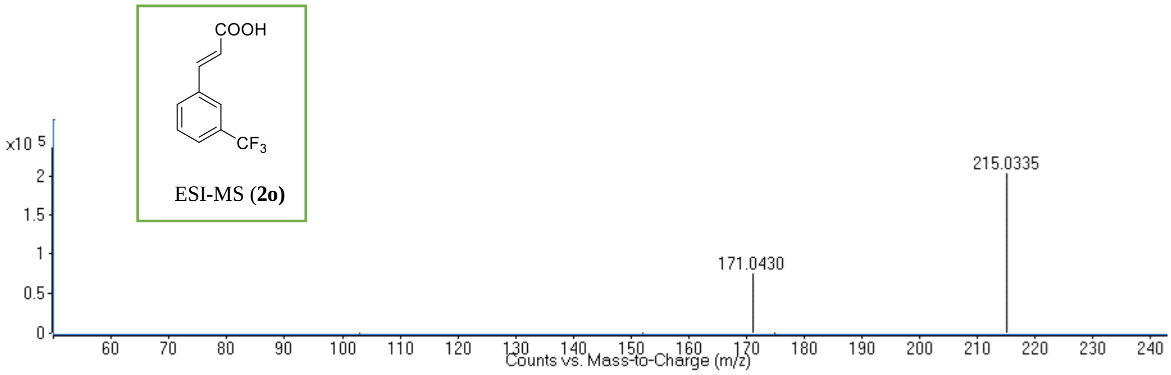

COOH
CF3
ESI-MS (2o)
x10 5
2
1.5
1
0.5
0
215.0335
171.0430
60
70
80
90
100
110
120
130
140
150
160
170
180
190
200
210
220
230
240
Counts vs. Mass-to-Charge (m/z)

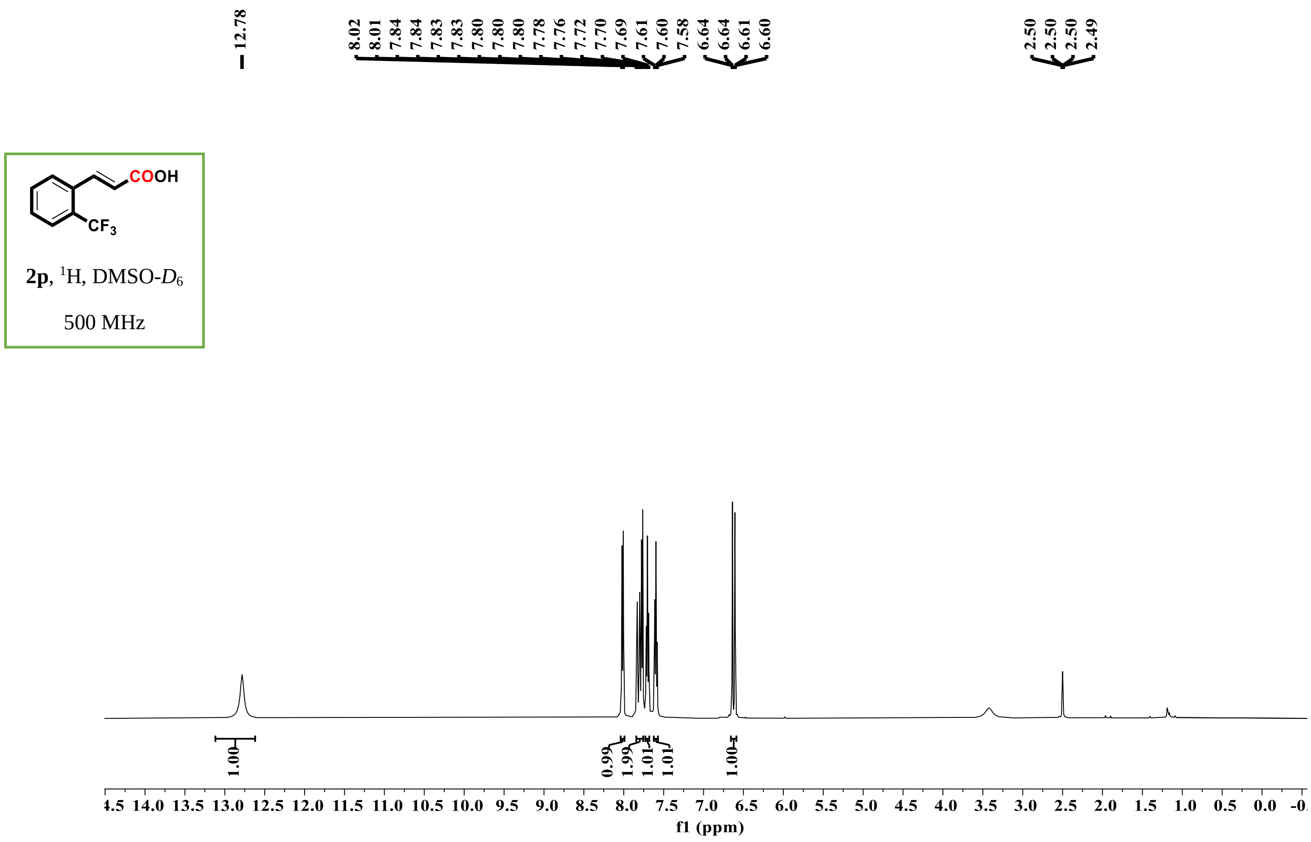

12.78
8.02
8.01
7.84
7.84
7.83
7.83
7.80
7.80
7.80
7.78
7.76
7.72
7.70
7.69
7.61
7.60
7.58
6.64
6.64
6.61
6.60
2.50
2.50
2.50
2.49
COOH
CF3
2p, 1H, DMSO-D6
500 MHz
1.00
0.99
1.99
1.01
1.01
1.00
f1 (ppm)

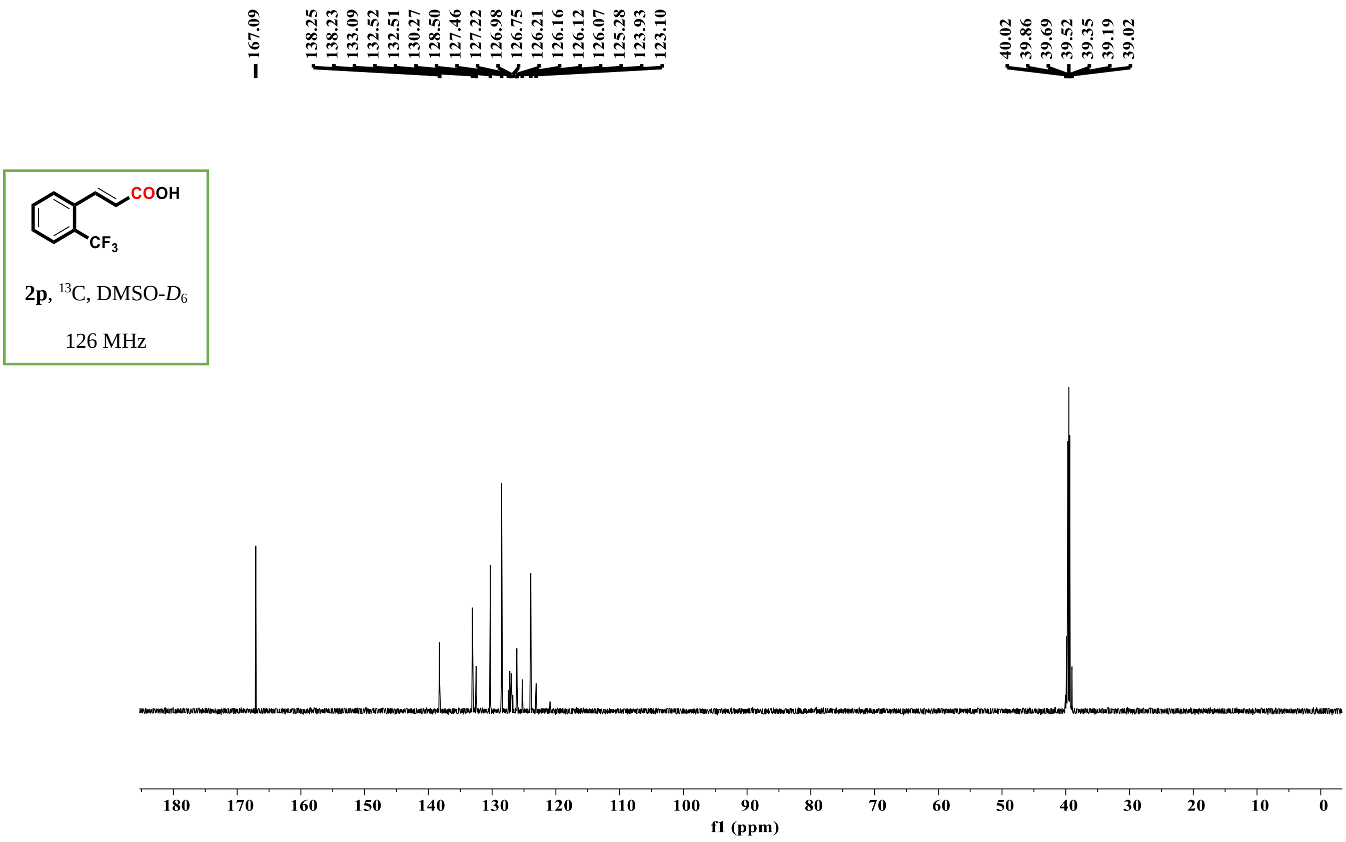

167.09
138.25
138.23
133.09
132.52
132.51
130.27
128.50
127.46
127.22
126.98
126.75
126.21
126.16
126.12
126.07
125.28
123.93
123.10
40.02
39.86
39.69
39.52
39.35
39.19
39.02
COOH
CF3
2p, 13C, DMSO-D6
126 MHz
f1 (ppm)

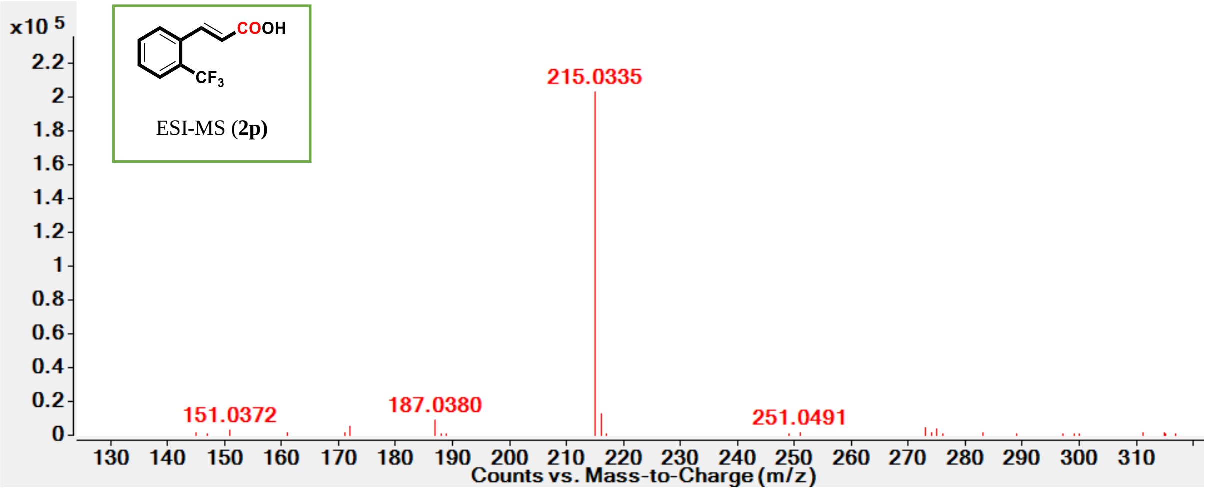
x10 5
COOH
CF3
ESI-MS (2p)
215.0335
151.0372
187.0380
251.0491
Counts vs. Mass-to-Charge (m/z)

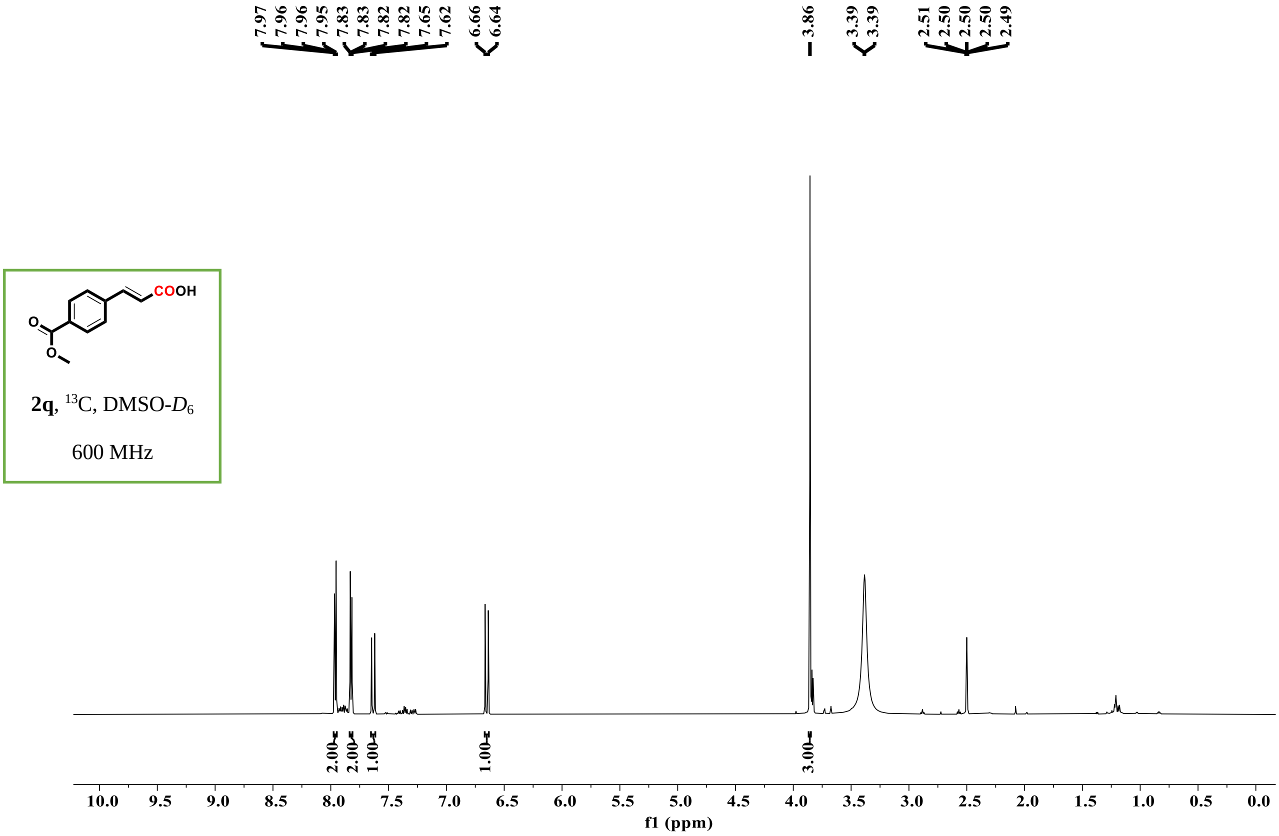
COOH
2q, 13C, DMSO-D6
600 MHz
f1 (ppm)

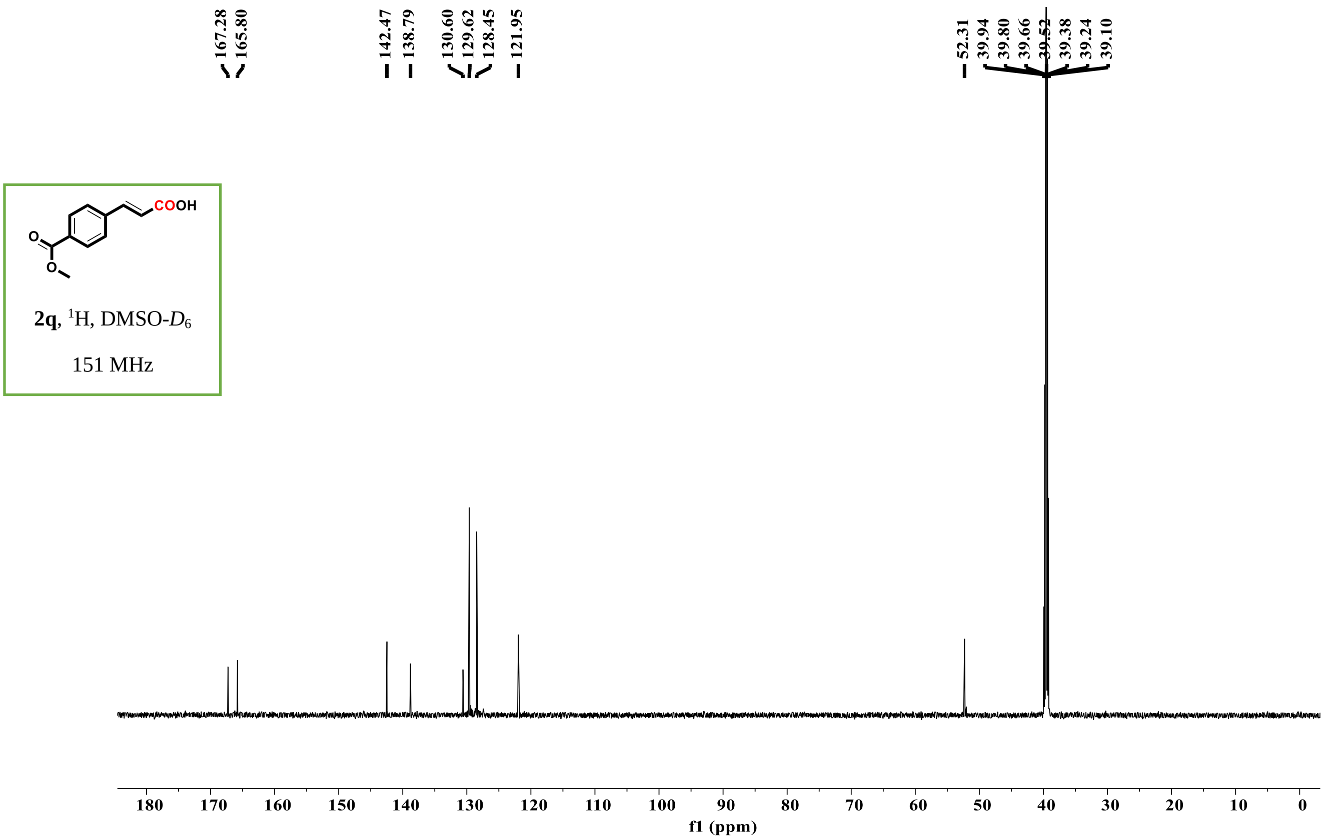
167.28
165.80
142.47
138.79
130.60
129.62
128.45
121.95
52.31
39.94
39.80
39.66
39.52
39.38
39.24
39.10
COOH
O
O
2q, ^{1}H, DMSO-D_6
151 MHz
180 170 160 150 140 130 120 110 100 90 80 70 60 50 40 30 20 10 0
f1 (ppm)

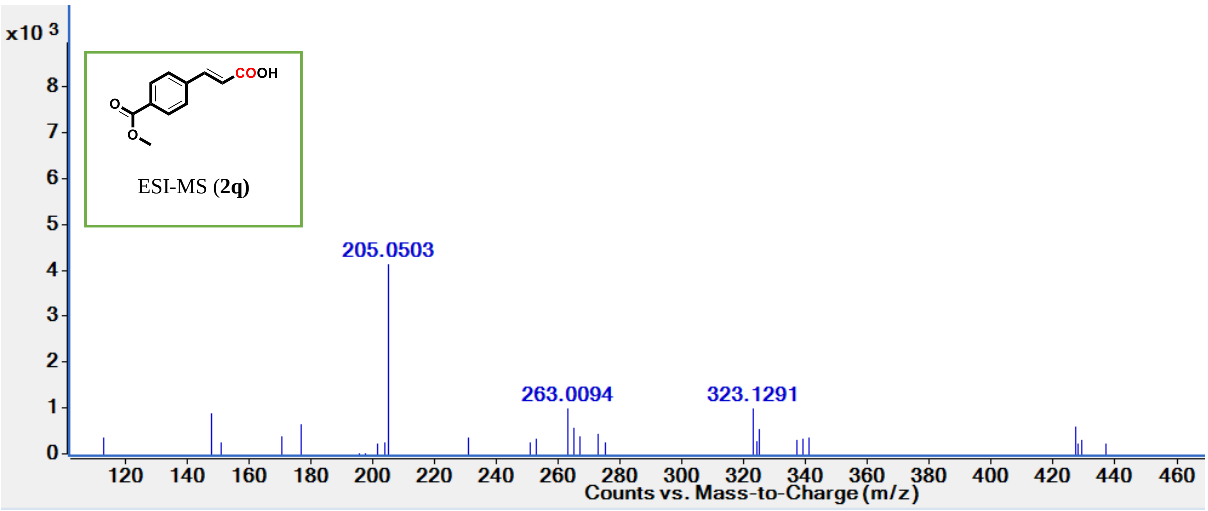
x10 3
COOH
O
O
ESI-MS (2q)
205.0503
263.0094
323.1291
Counts vs. Mass-to-Charge (m/z)

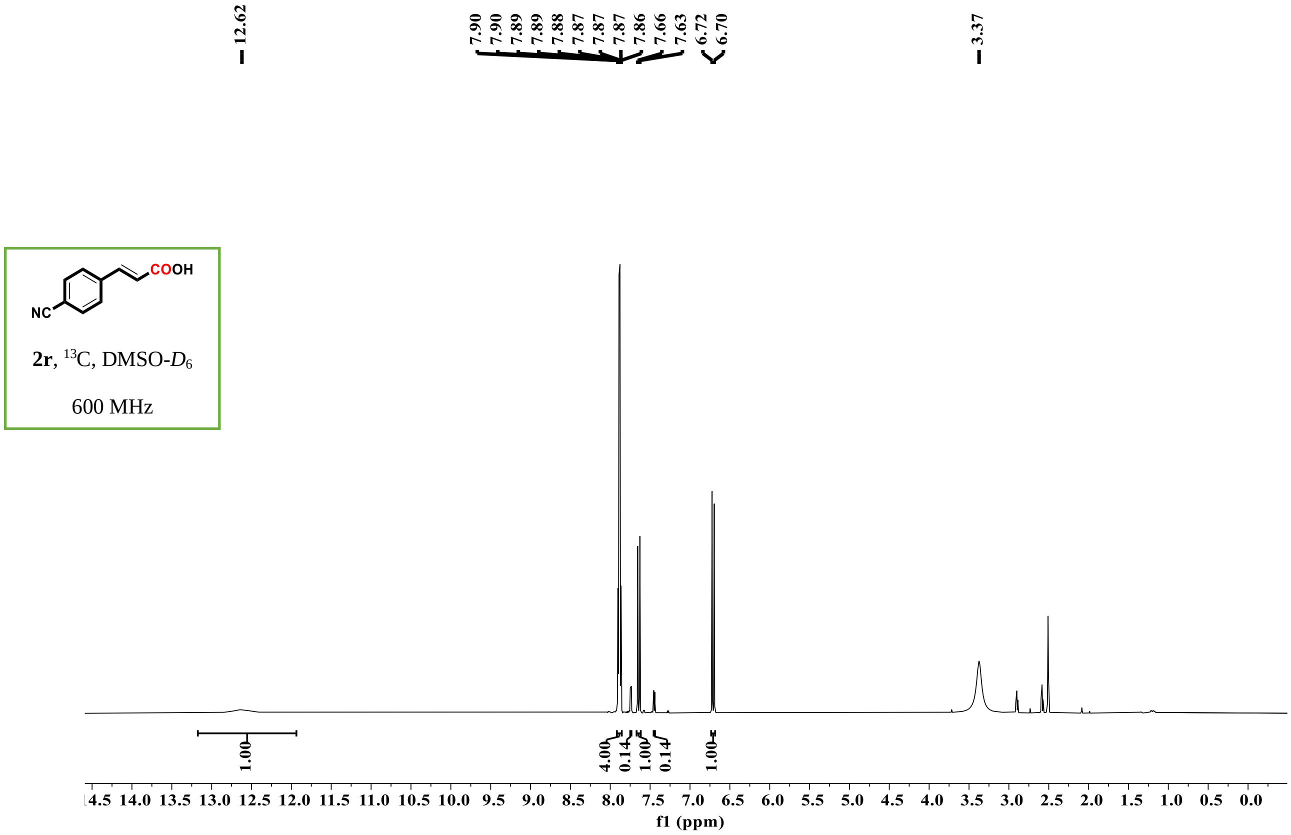
12.62
7.90
7.90
7.89
7.89
7.88
7.87
7.87
7.87
7.86
7.66
7.63
6.72
6.70
3.37
COOH
NC
2r, 13C, DMSO-D6
600 MHz
1.00
4.00
0.14
1.00
0.14
1.00
f1 (ppm)

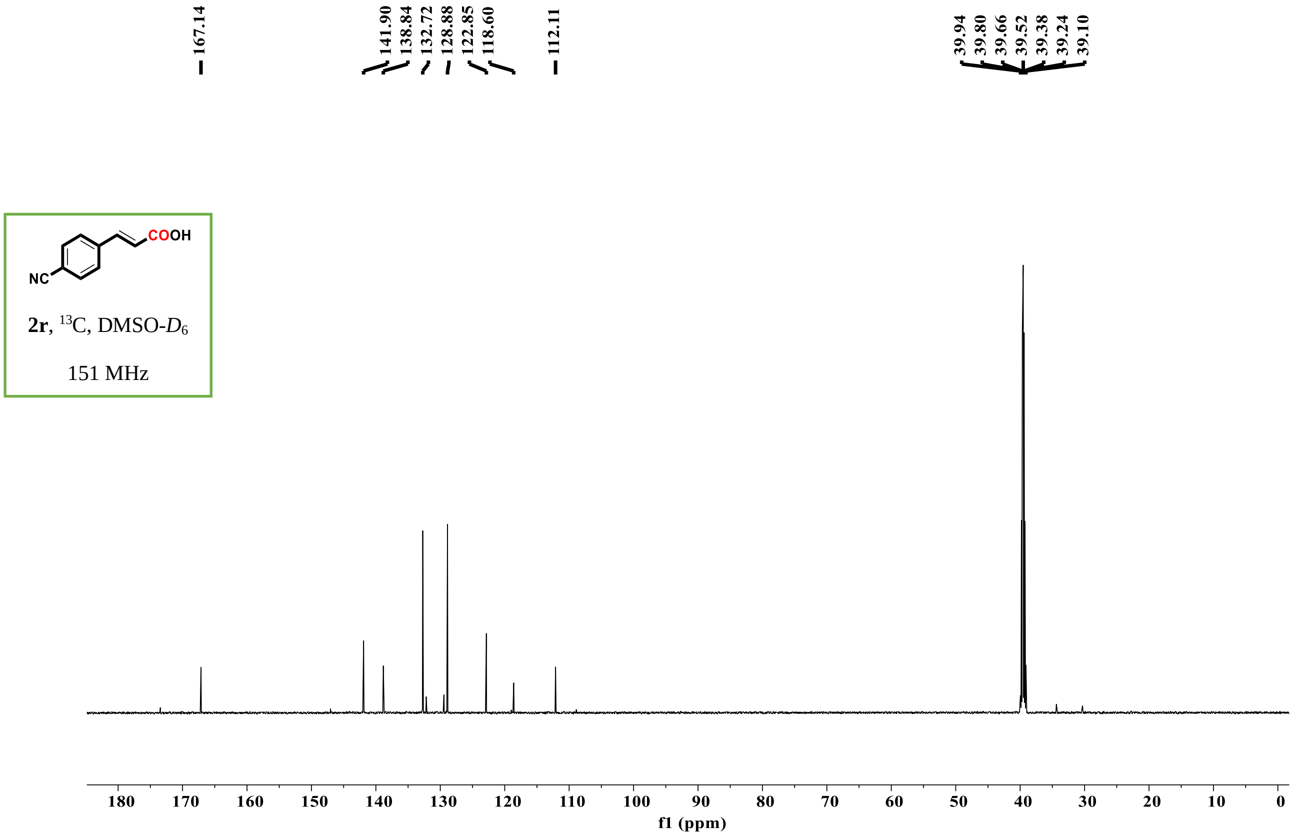
167.14
141.90
138.84
132.72
128.88
122.85
118.60
112.11
39.94
39.80
39.66
39.52
39.38
39.24
39.10
COOH
NC
2r, 13C, DMSO-D6
151 MHz
f1 (ppm)

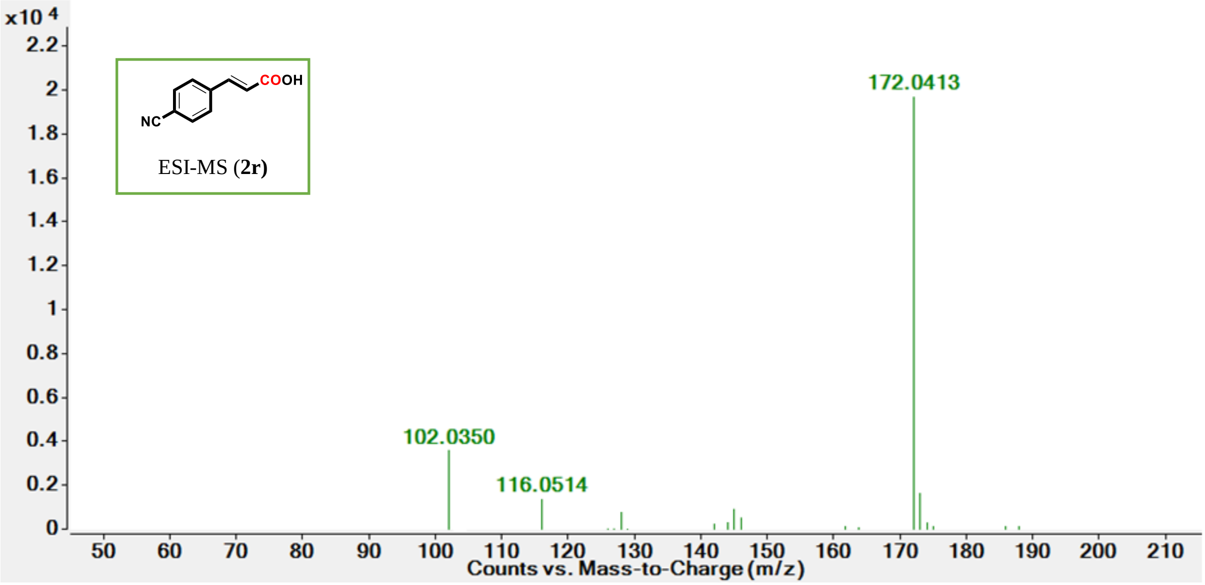
x10 4
172.0413
102.0350
116.0514
NC
COOH
ESI-MS (2r)
Counts vs. Mass-to-Charge (m/z)

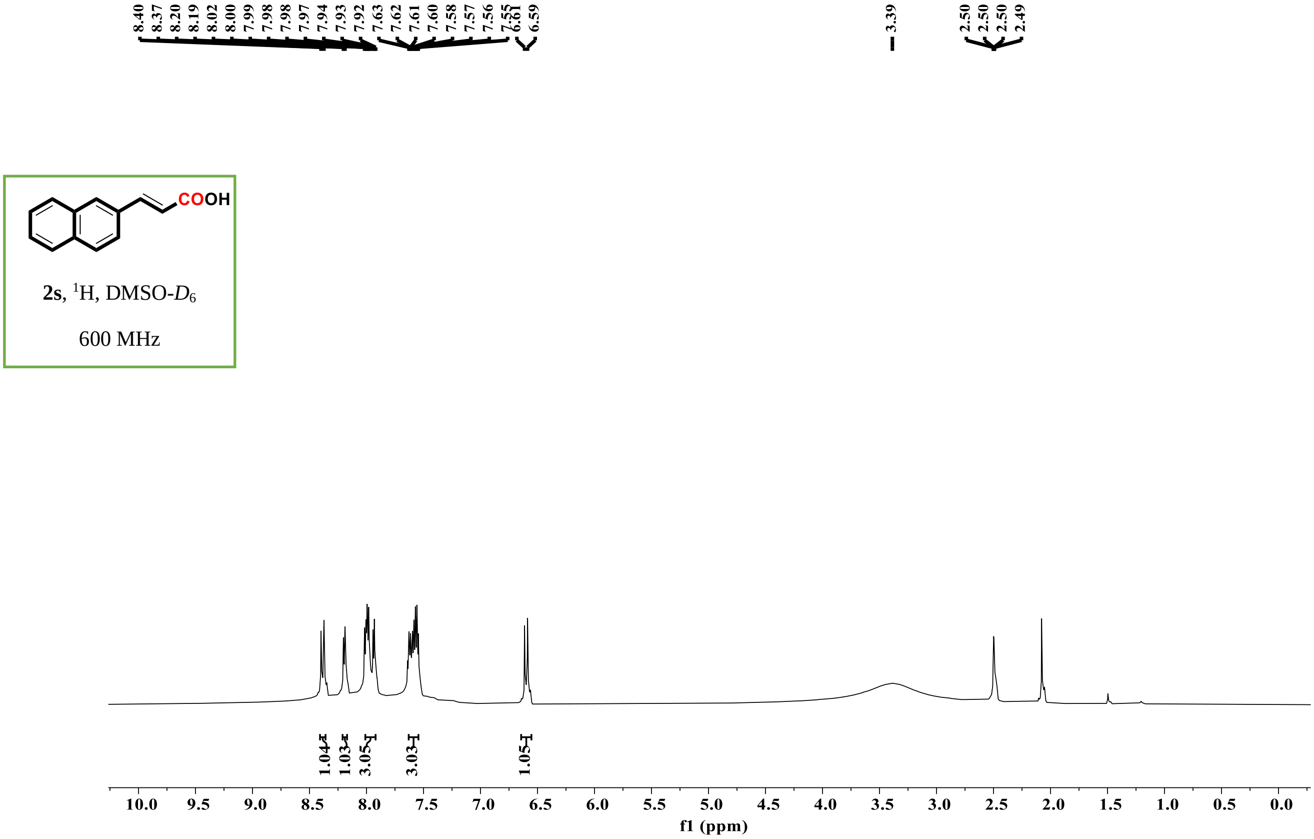
8.40
8.37
8.20
8.19
8.02
8.00
7.99
7.98
7.98
7.97
7.94
7.93
7.92
7.63
7.62
7.61
7.60
7.58
7.57
7.56
7.55
6.61
6.59
3.39
2.50
2.50
2.50
2.49
COOH
2s, 1H, DMSO-D6
600 MHz
1.04
1.03
3.05
3.03
1.05
f1 (ppm)

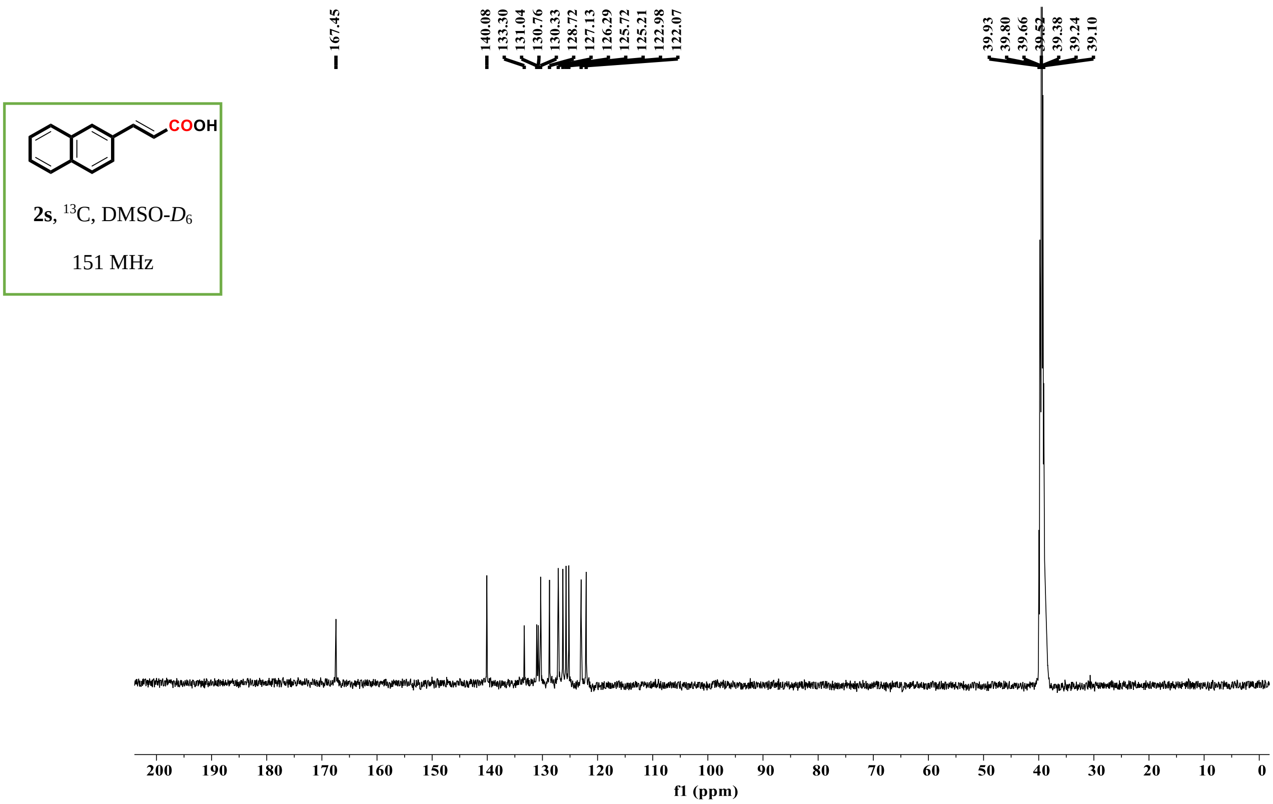

167.45
140.08
133.30
131.04
130.76
130.33
128.72
127.13
126.29
125.72
125.21
122.98
122.07
39.93
39.80
39.66
39.52
39.38
39.24
39.10
COOH
2s, ^{13}C, DMSO-D_6
151 MHz
200 190 180 170 160 150 140 130 120 110 100 90 80 70 60 50 40 30 20 10 0
f1 (ppm)

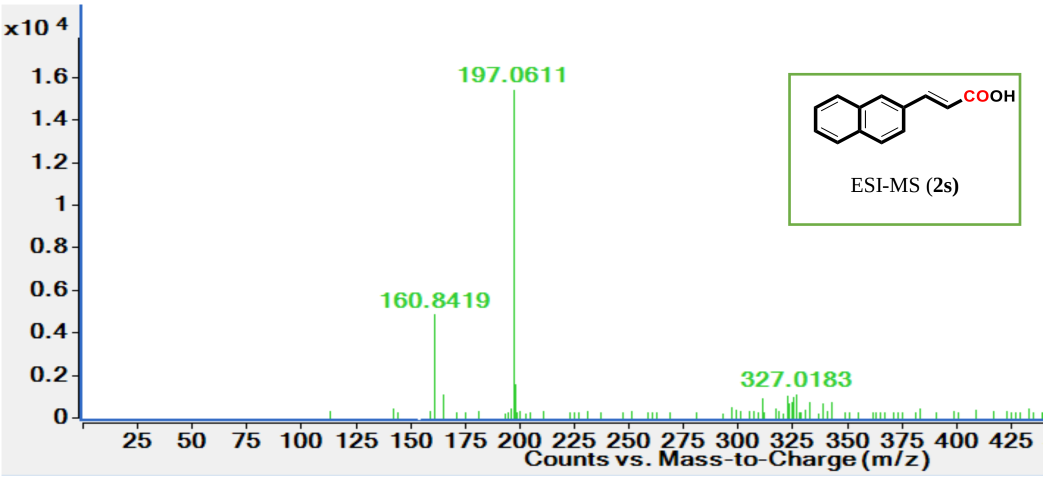

x10 4
1.6
1.4
1.2
1
0.8
0.6
0.4
0.2
0
197.0611
160.8419
327.0183
COOH
ESI-MS (2s)
25 50 75 100 125 150 175 200 225 250 275 300 325 350 375 400 425
Counts vs. Mass-to-Charge (m/z)

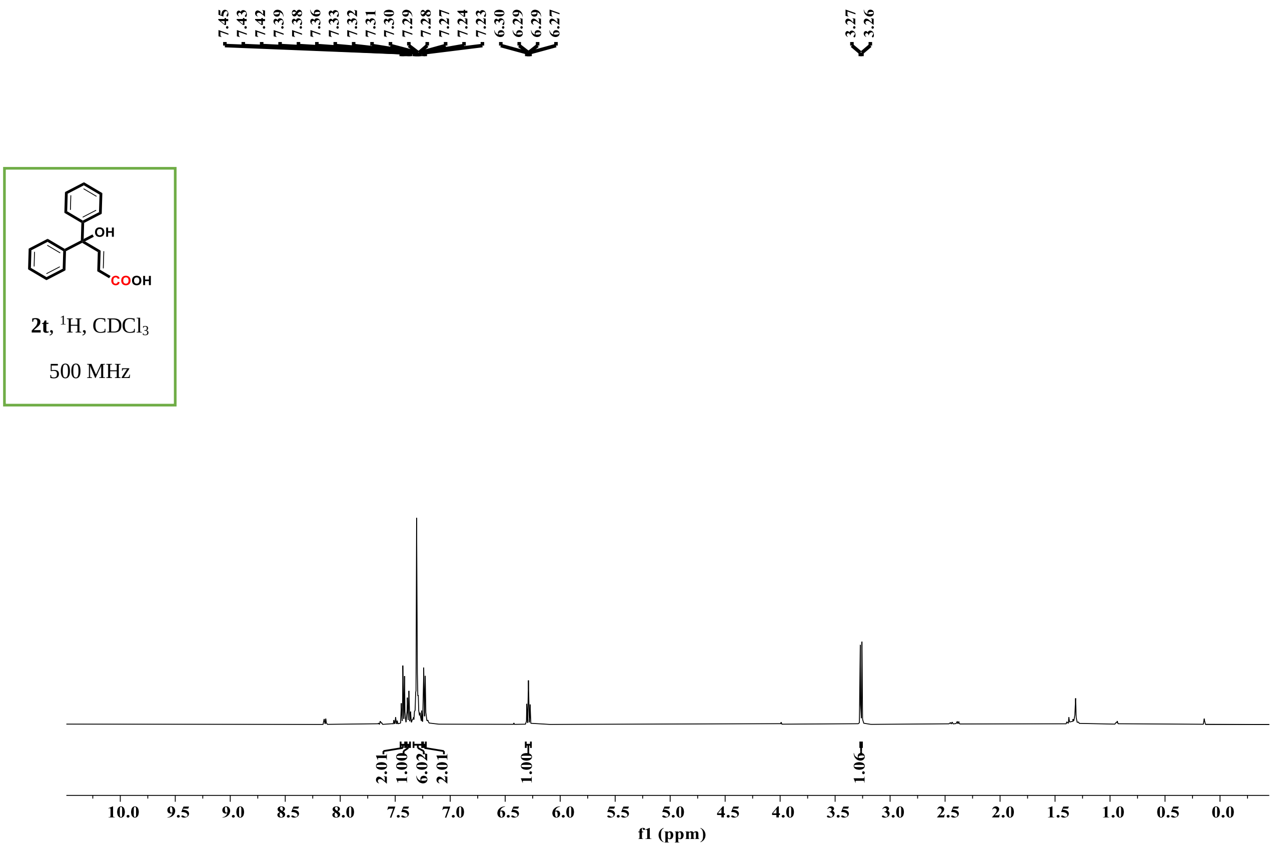
7.45
7.43
7.42
7.39
7.38
7.36
7.33
7.32
7.31
7.30
7.29
7.28
7.27
7.24
7.23
6.30
6.29
6.29
6.27
3.27
3.26
OH
COOH
2t, ^{1}H, CDCl$_3$
500 MHz
2.01
1.00
6.02
2.01
1.00
1.06
10.0 9.5 9.0 8.5 8.0 7.5 7.0 6.5 6.0 5.5 5.0 4.5 4.0 3.5 3.0 2.5 2.0 1.5 1.0 0.5 0.0
f1 (ppm)

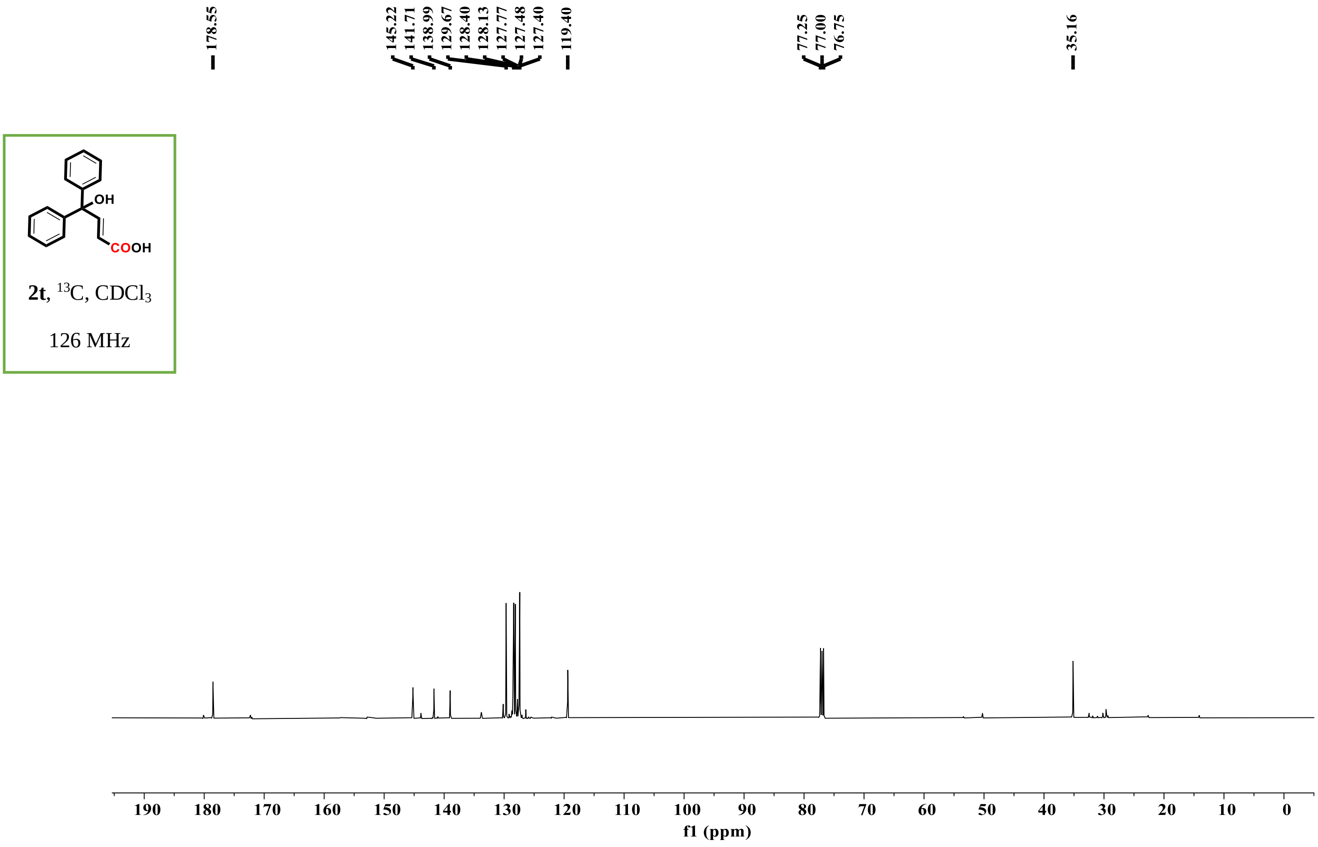
178.55
145.22
141.71
138.99
129.67
128.40
128.13
127.77
127.48
127.40
119.40
77.25
77.00
76.75
35.16
OH
COOH
2t, ^{13}C, CDCl$_3$
126 MHz
190 180 170 160 150 140 130 120 110 100 90 80 70 60 50 40 30 20 10 0
f1 (ppm)

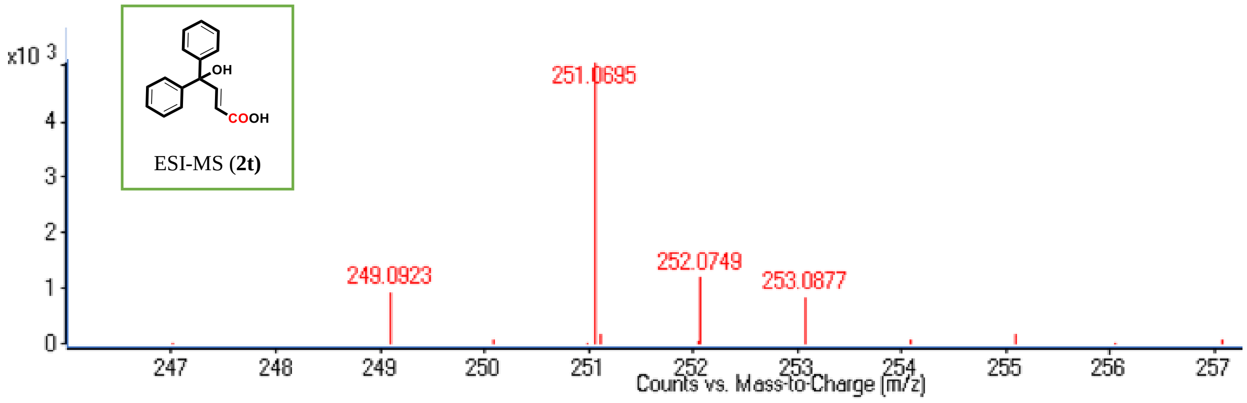

x10 3
OH
COOH
ESI-MS (2t)
251.0695
249.0923
252.0749
253.0877
247 248 249 250 251 252 253 254 255 256 257
Counts vs. Mass-to-Charge (m/z)

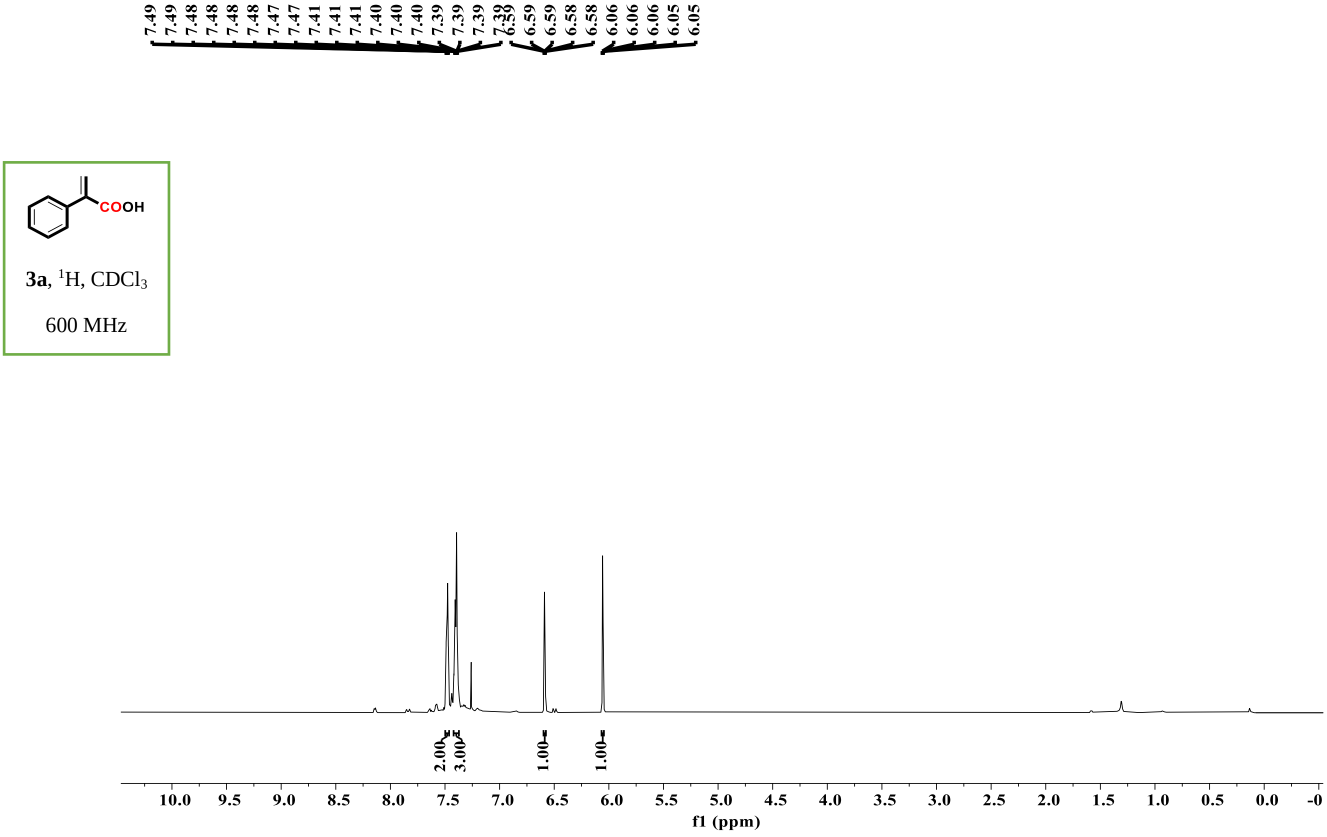

7.49 7.49 7.48 7.48 7.48 7.48 7.47 7.47 7.41 7.41 7.41 7.40 7.40 7.40 7.39 7.39 7.39 7.39 6.59 6.59 6.59 6.58 6.58 6.06 6.06 6.06 6.05 6.05
COOH
3a, 1H, CDCl3
600 MHz
2.00 3.00 1.00 1.00
10.0 9.5 9.0 8.5 8.0 7.5 7.0 6.5 6.0 5.5 5.0 4.5 4.0 3.5 3.0 2.5 2.0 1.5 1.0 0.5 0.0 -0
f1 (ppm)

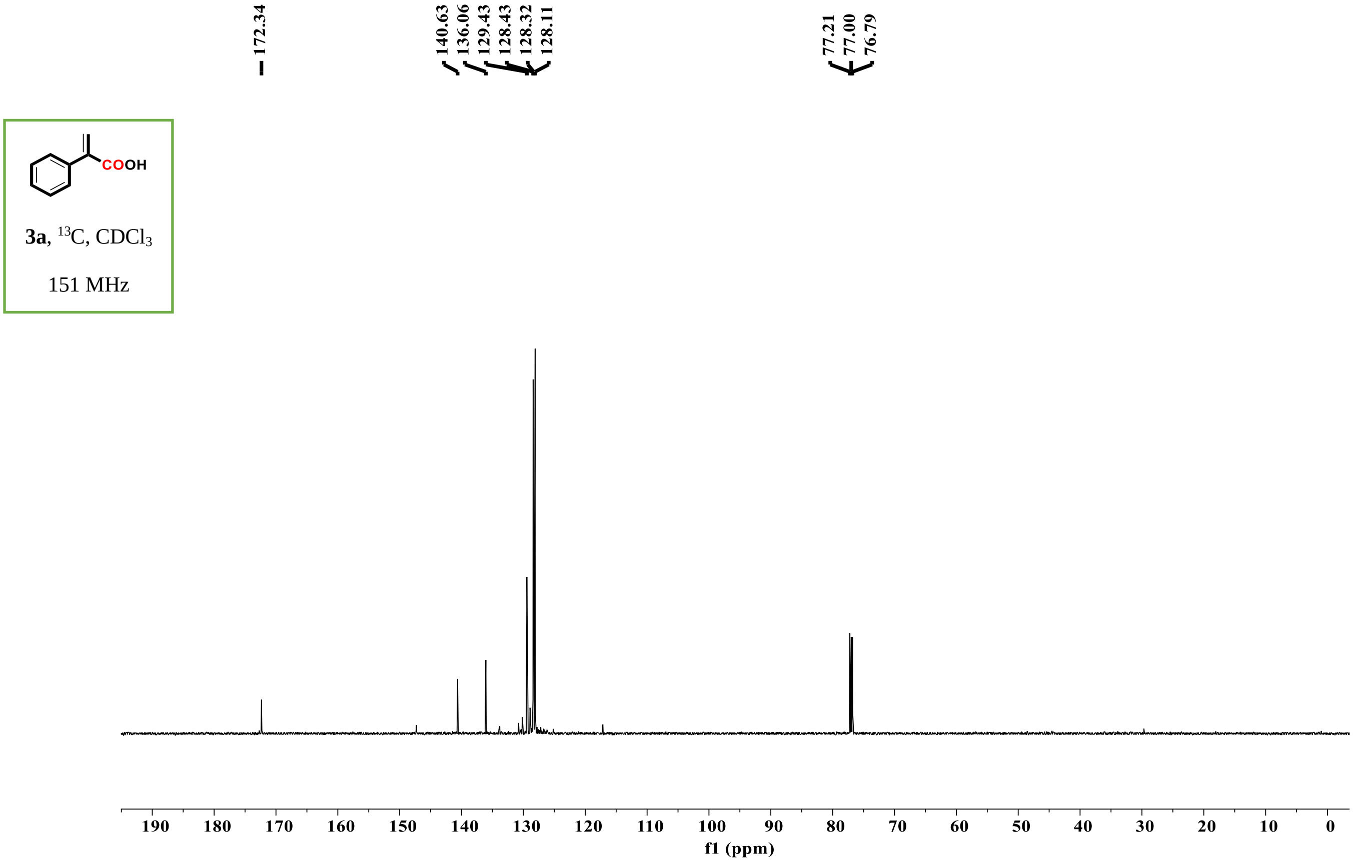

172.34
140.63
136.06
129.43
128.43
128.32
128.11
77.21
77.00
76.79
COOH
3a, ^{13}C, CDCl$_3$
151 MHz
190 180 170 160 150 140 130 120 110 100 90 80 70 60 50 40 30 20 10 0
f1 (ppm)

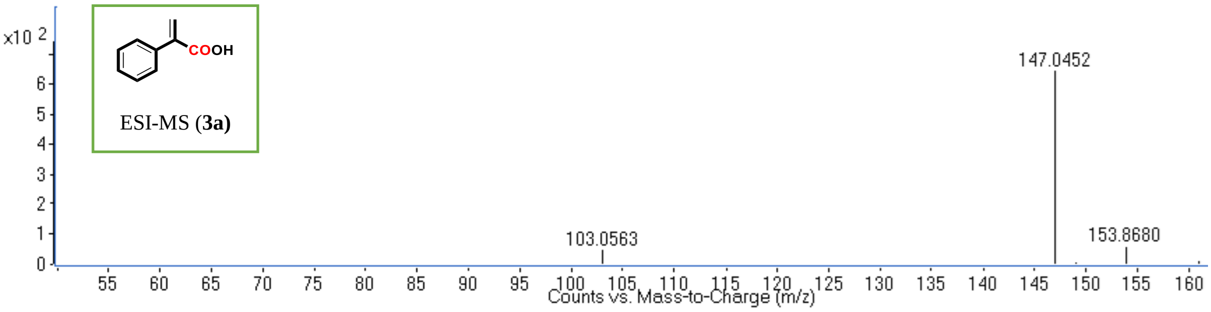

x10 2
COOH
ESI-MS (3a)
147.0452
103.0563
153.8680
Counts vs. Mass-to-Charge (m/z)

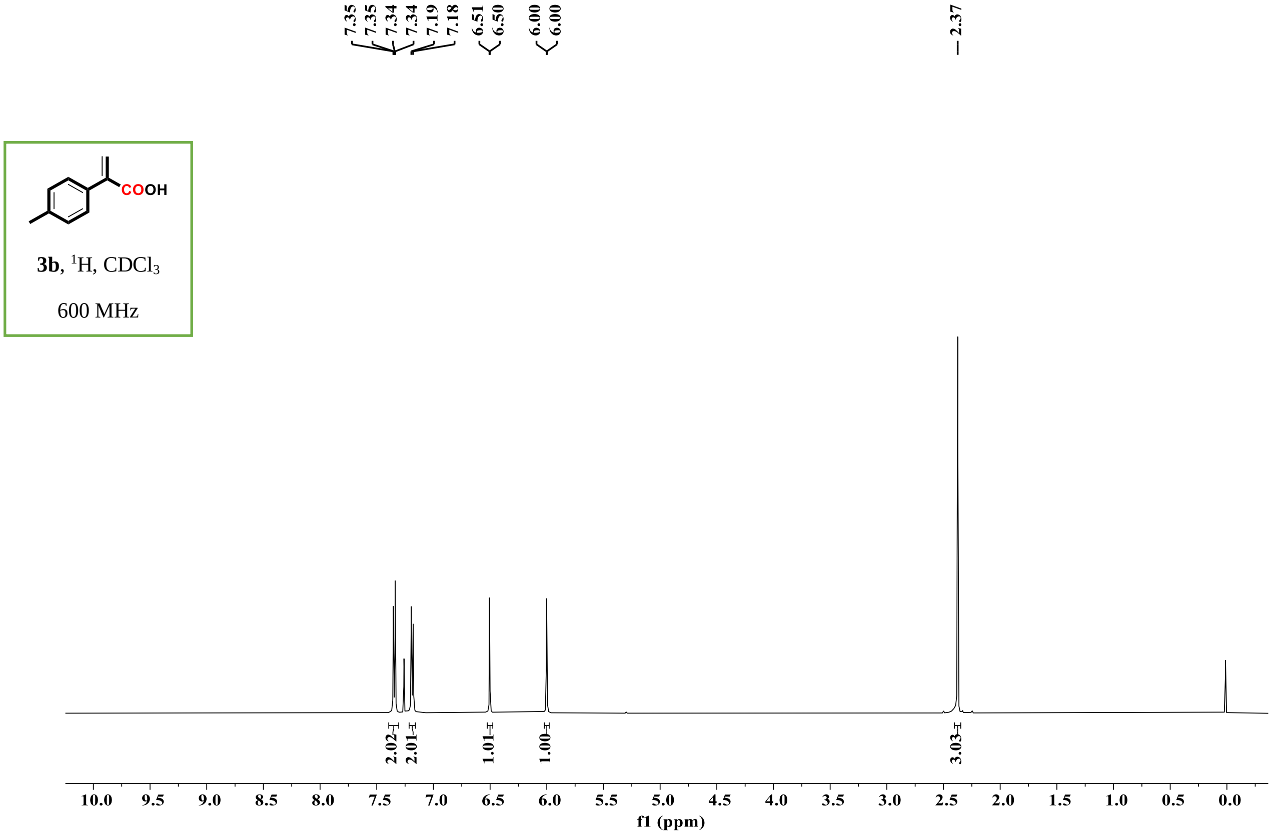
7.35
7.35
7.34
7.34
7.19
7.18
6.51
6.50
6.00
6.00
2.37
COOH
3b, ^{1}H, $CDCl_3$
600 MHz
2.02
2.01
1.01
1.00
3.03
10.0
9.5
9.0
8.5
8.0
7.5
7.0
6.5
6.0
5.5
5.0
4.5
4.0
3.5
3.0
2.5
2.0
1.5
1.0
0.5
0.0
f1 (ppm)

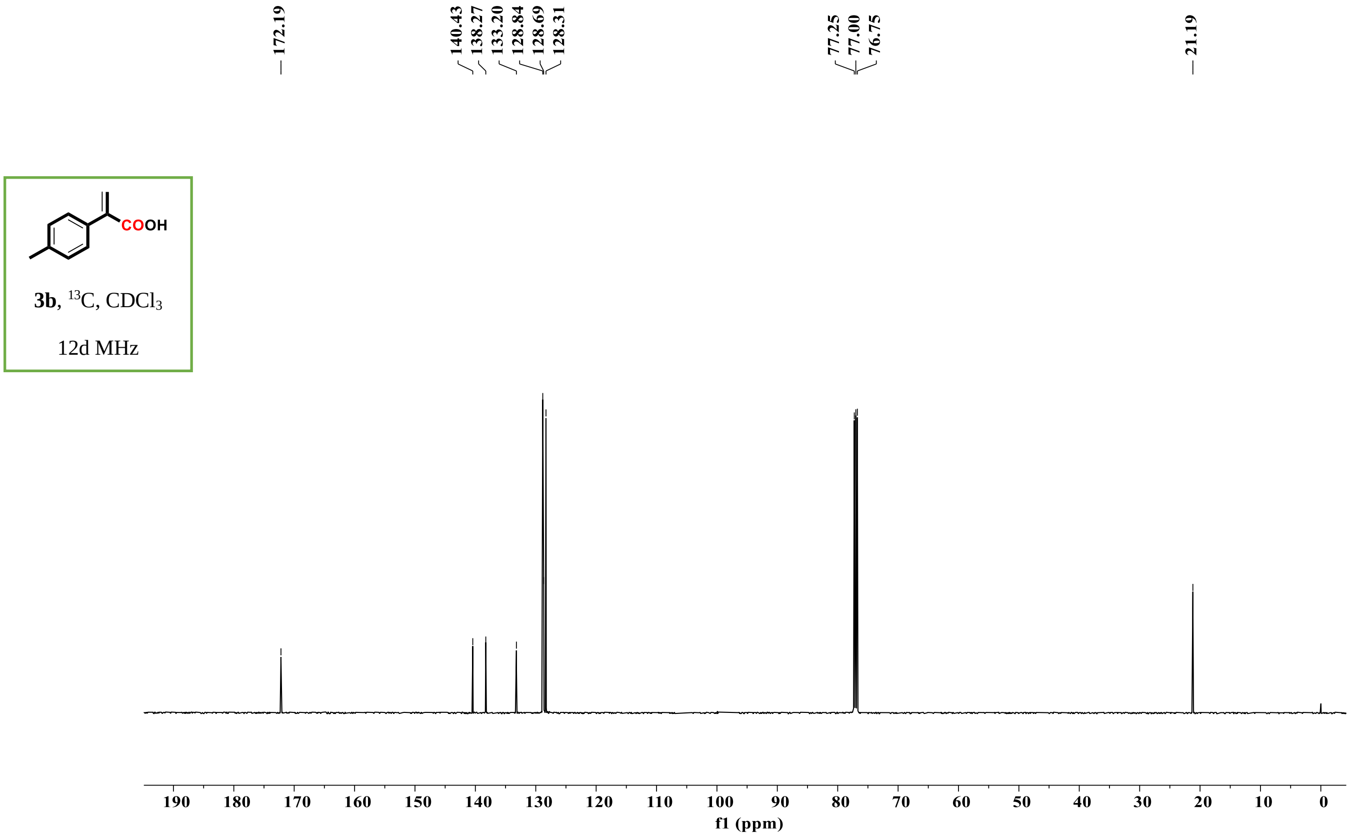
172.19
140.43
138.27
133.20
128.84
128.69
128.31
77.25
77.00
76.75
21.19
COOH
3b, ^{13}C, $CDCl_3$
12d MHz
190
180
170
160
150
140
130
120
110
100
90
80
70
60
50
40
30
20
10
0
f1 (ppm)

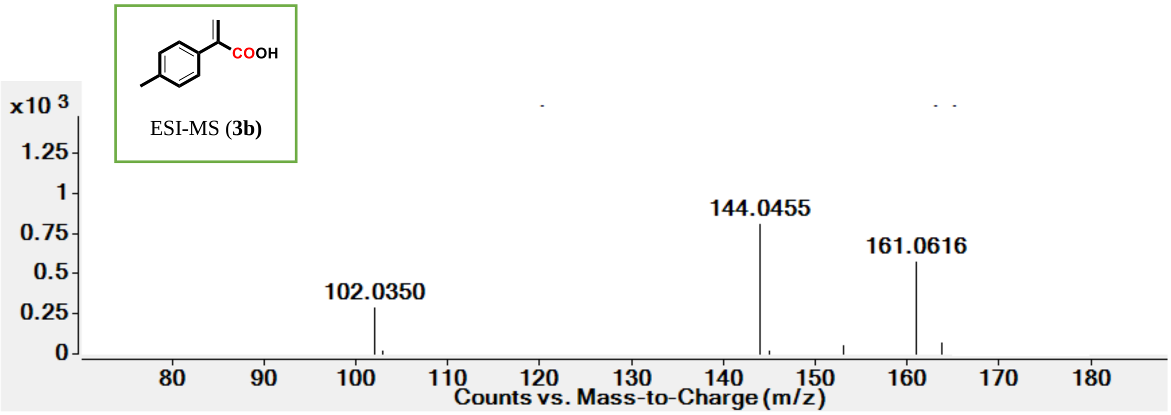
COOH
ESI-MS (3b)
x10 3
1.25
1
0.75
0.5
0.25
0
102.0350
144.0455
161.0616
80 90 100 110 120 130 140 150 160 170 180
Counts vs. Mass-to-Charge (m/z)

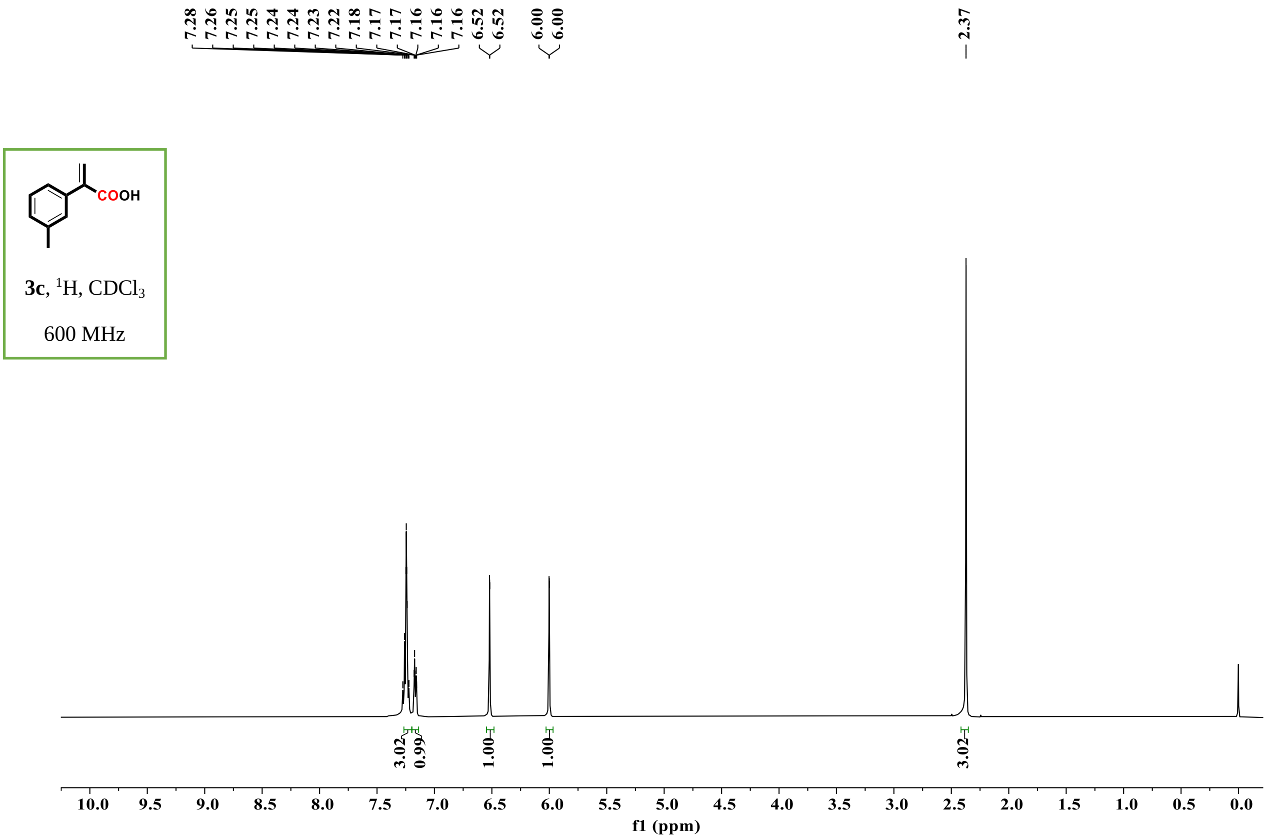
7.28 7.26 7.25 7.25 7.24 7.24 7.23 7.22 7.18 7.17 7.17 7.16 7.16 7.16 6.52 6.52 6.00 6.00
2.37
COOH
3c, 1H, CDCl3
600 MHz
3.02 0.99 1.00 1.00 3.02
10.0 9.5 9.0 8.5 8.0 7.5 7.0 6.5 6.0 5.5 5.0 4.5 4.0 3.5 3.0 2.5 2.0 1.5 1.0 0.5 0.0
f1 (ppm)

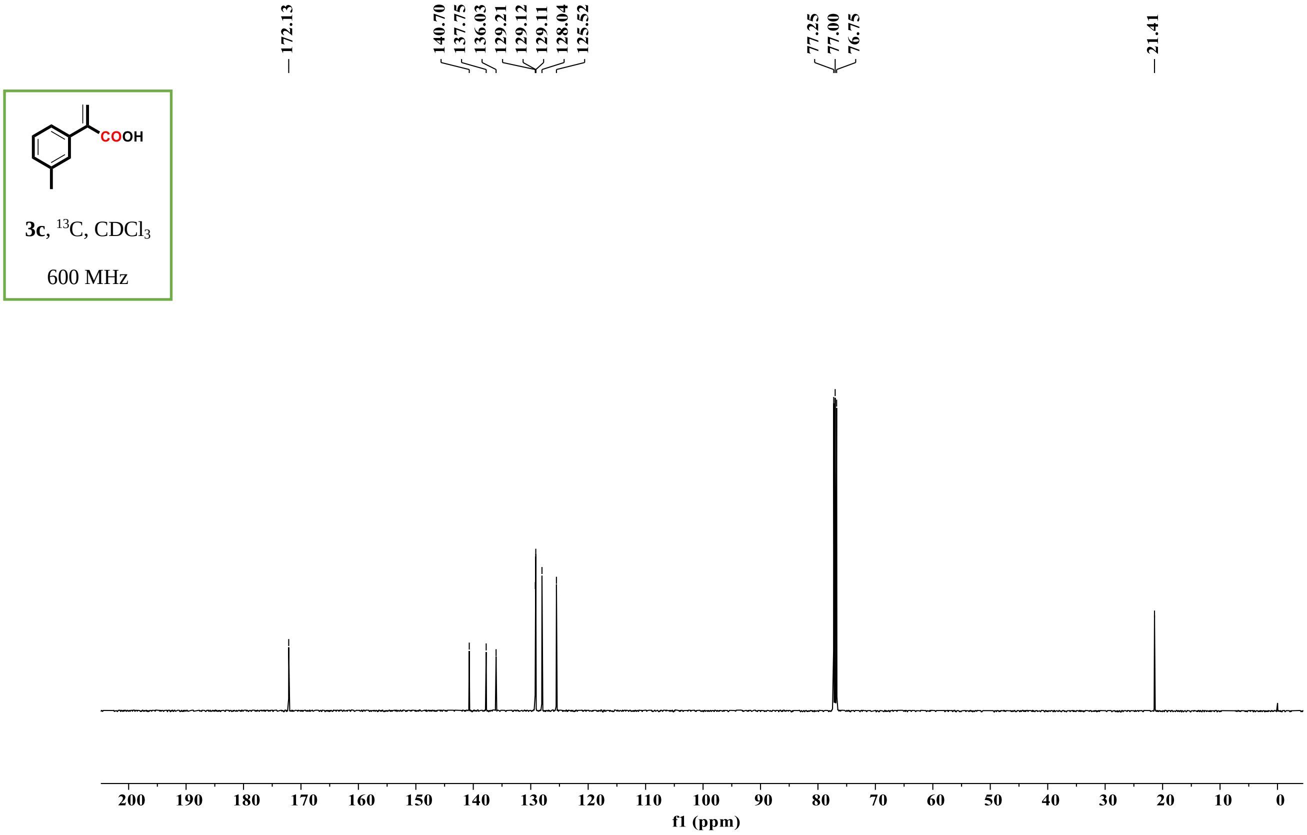
172.13
140.70
137.75
136.03
129.21
129.12
129.11
128.04
125.52
77.25
77.00
76.75
21.41
COOH
3c, ^{13}C, $CDCl_3$
600 MHz
f1 (ppm)

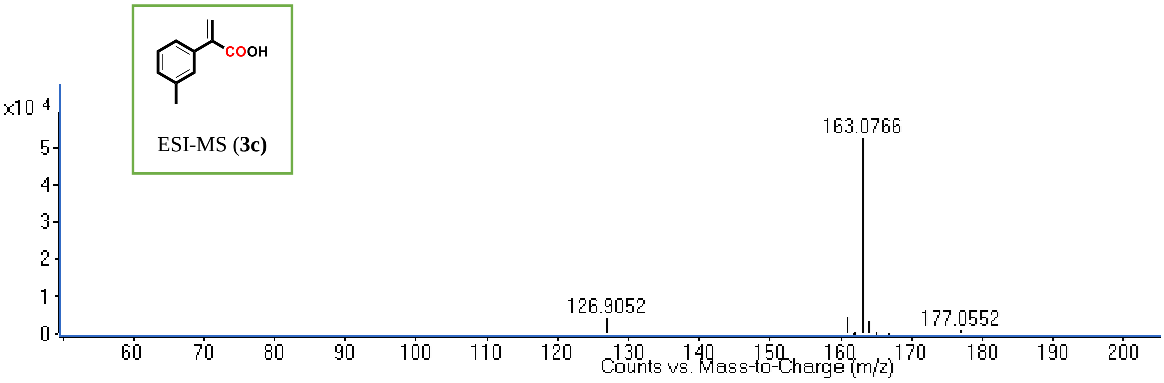
COOH
ESI-MS (3c)
x10 4
163.0766
126.9052
177.0552
Counts vs. Mass-to-Charge (m/z)

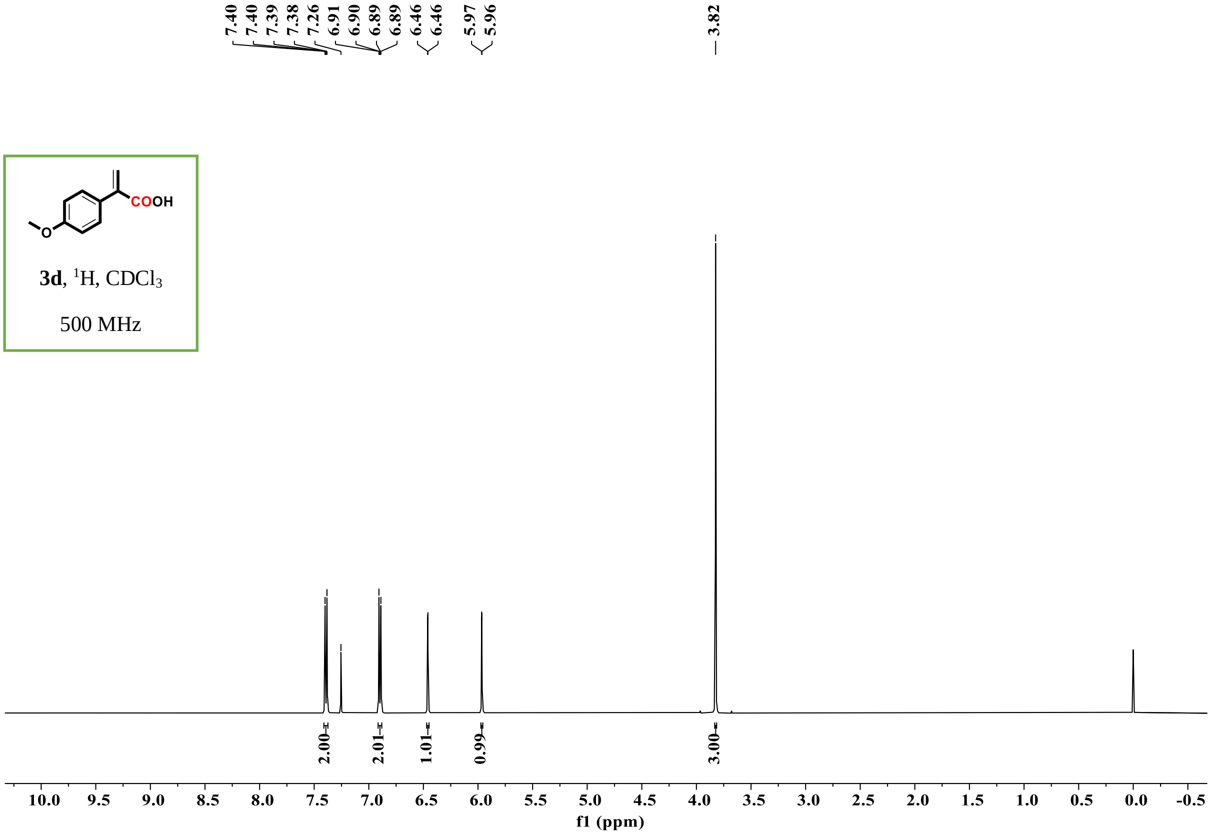
7.40
7.40
7.39
7.38
7.26
6.91
6.90
6.89
6.89
6.46
6.46
5.97
5.96
3.82
COOH
O
3d, 1H, CDCl3
500 MHz
2.00
2.01
1.01
0.99
3.00
10.0 9.5 9.0 8.5 8.0 7.5 7.0 6.5 6.0 5.5 5.0 4.5 4.0 3.5 3.0 2.5 2.0 1.5 1.0 0.5 0.0 -0.5
f1 (ppm)

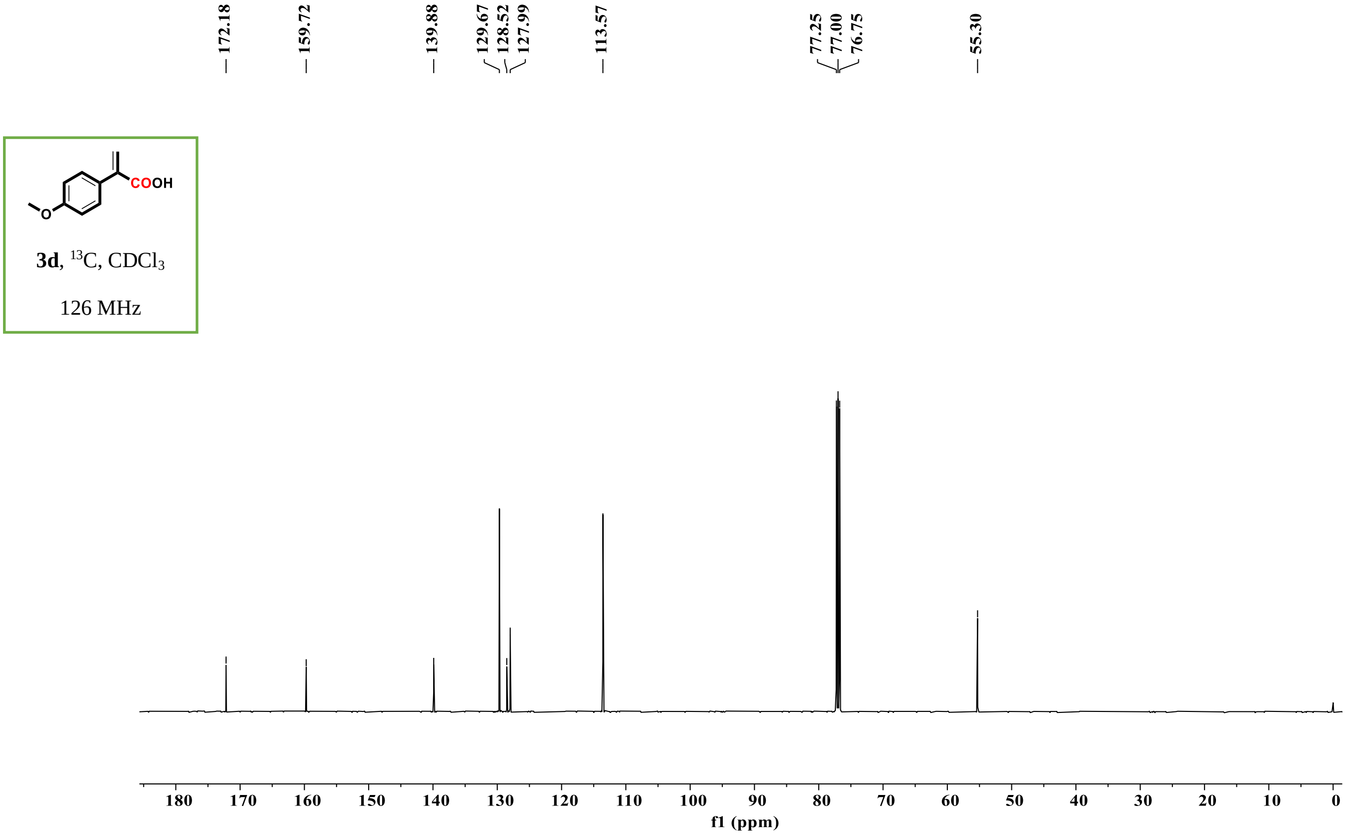
172.18
159.72
139.88
129.67
128.52
127.99
113.57
77.25
77.00
76.75
55.30
COOH
O
3d, 13C, CDCl3
126 MHz
180 170 160 150 140 130 120 110 100 90 80 70 60 50 40 30 20 10 0
f1 (ppm)

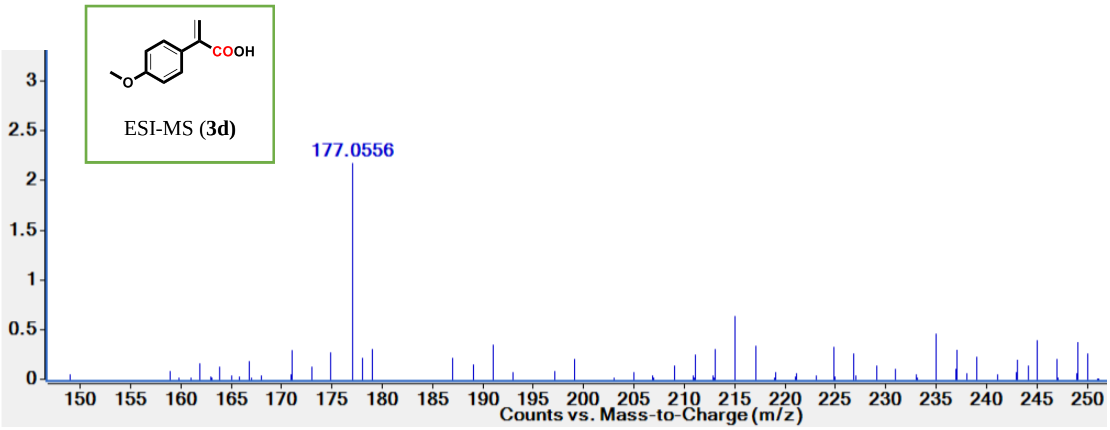
COOH
ESI-MS (**3d**)
177.0556
3
2.5
2
1.5
1
0.5
0
150 155 160 165 170 175 180 185 190 195 200 205 210 215 220 225 230 235 240 245 250
Counts vs. Mass-to-Charge (m/z)

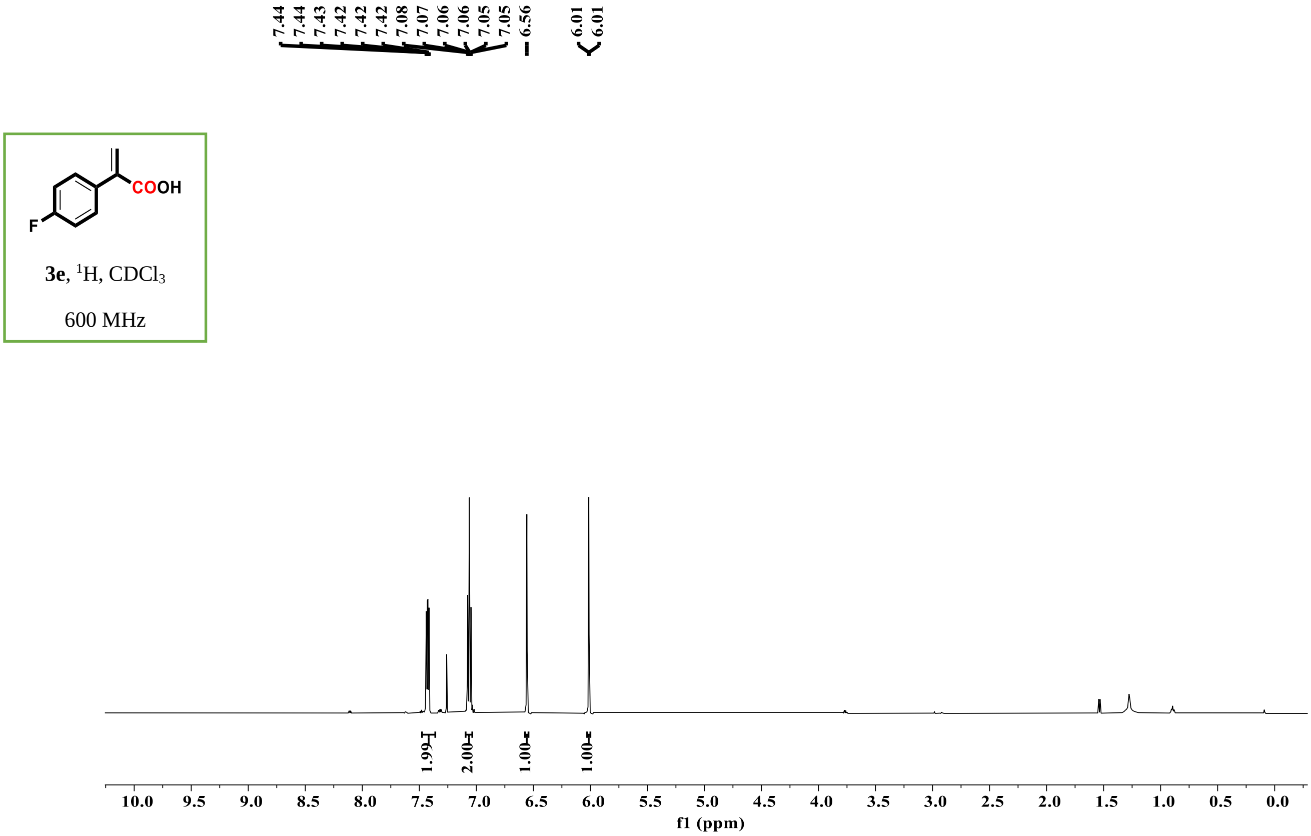
7.44 7.44 7.43 7.42 7.42 7.42 7.08 7.07 7.06 7.06 7.05 7.05 6.56 6.01 6.01
COOH
F
3e, ^{1}H, $CDCl_3$
600 MHz
1.99
2.00
1.00
1.00
10.0 9.5 9.0 8.5 8.0 7.5 7.0 6.5 6.0 5.5 5.0 4.5 4.0 3.5 3.0 2.5 2.0 1.5 1.0 0.5 0.0
f1 (ppm)

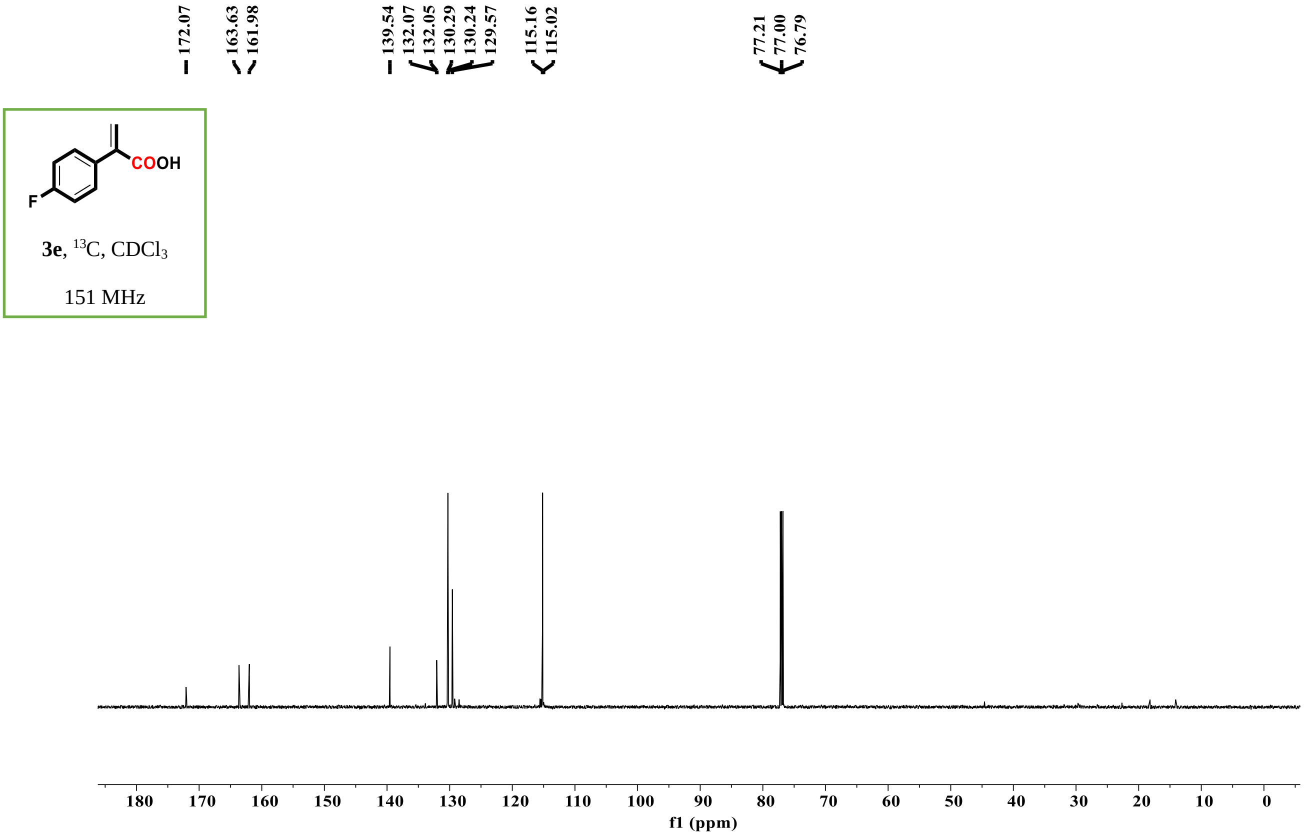
172.07
163.63
161.98
139.54
132.07
132.05
130.29
130.24
129.57
115.16
115.02
77.21
77.00
76.79
F
COOH
3e, 13C, CDCl3
151 MHz
180
170
160
150
140
130
120
110
100
90
80
70
60
50
40
30
20
10
0
f1 (ppm)

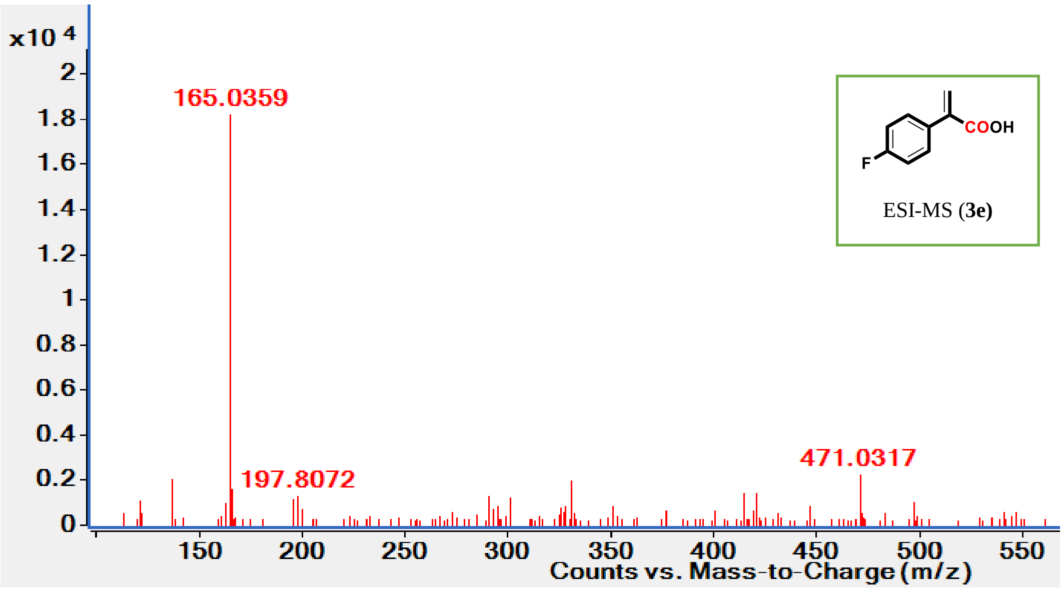
x10 4
2
1.8
1.6
1.4
1.2
1
0.8
0.6
0.4
0.2
0
165.0359
197.8072
471.0317
150
200
250
300
350
400
450
500
550
Counts vs. Mass-to-Charge (m/z)
F
COOH
ESI-MS (3e)

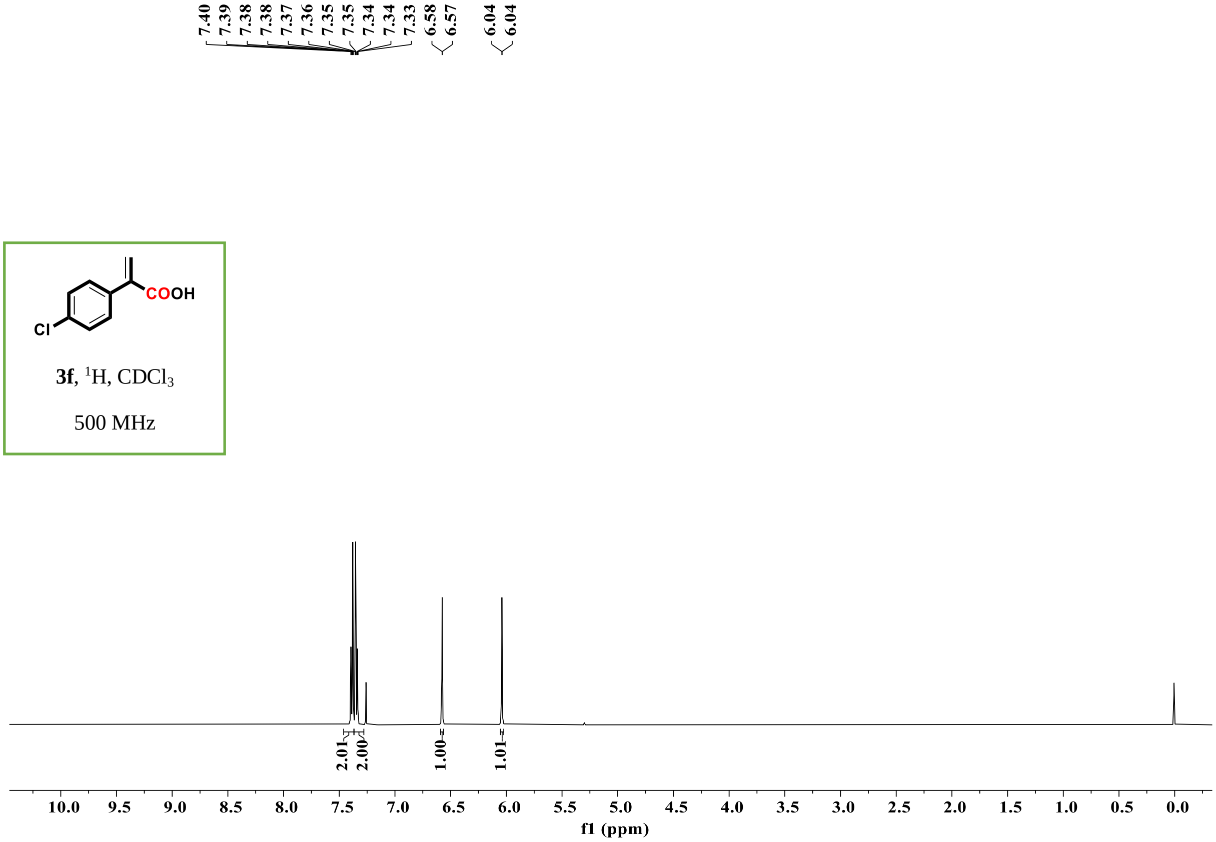
7.40
7.39
7.38
7.38
7.37
7.36
7.35
7.35
7.34
7.34
7.33
6.58
6.57
6.04
6.04
COOH
Cl
3f, 1H, CDCl3
500 MHz
2.01
2.00
1.00
1.01
10.0 9.5 9.0 8.5 8.0 7.5 7.0 6.5 6.0 5.5 5.0 4.5 4.0 3.5 3.0 2.5 2.0 1.5 1.0 0.5 0.0
f1 (ppm)

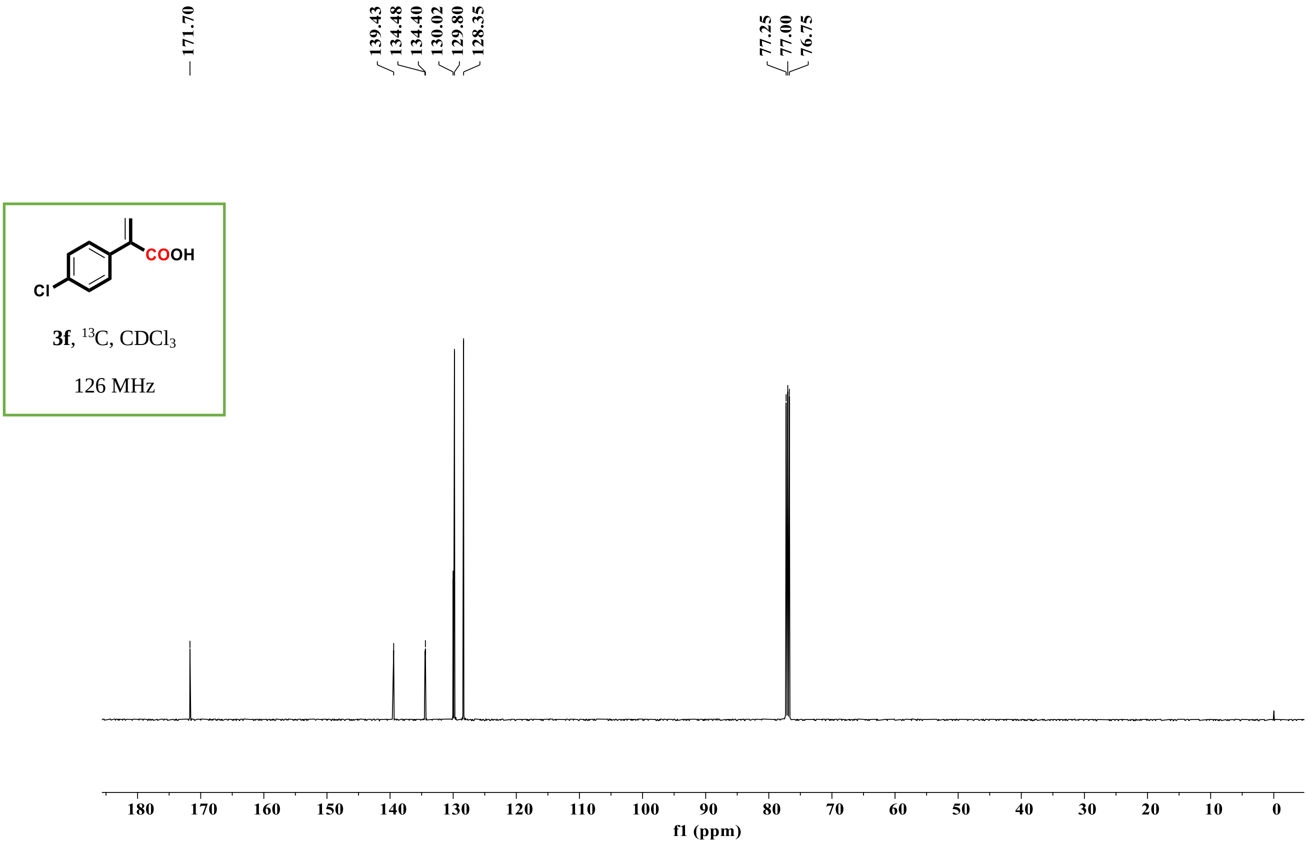
171.70
139.43
134.48
134.40
130.02
129.80
128.35
77.25
77.00
76.75
COOH
Cl
3f, 13C, CDCl3
126 MHz
180 170 160 150 140 130 120 110 100 90 80 70 60 50 40 30 20 10 0
f1 (ppm)

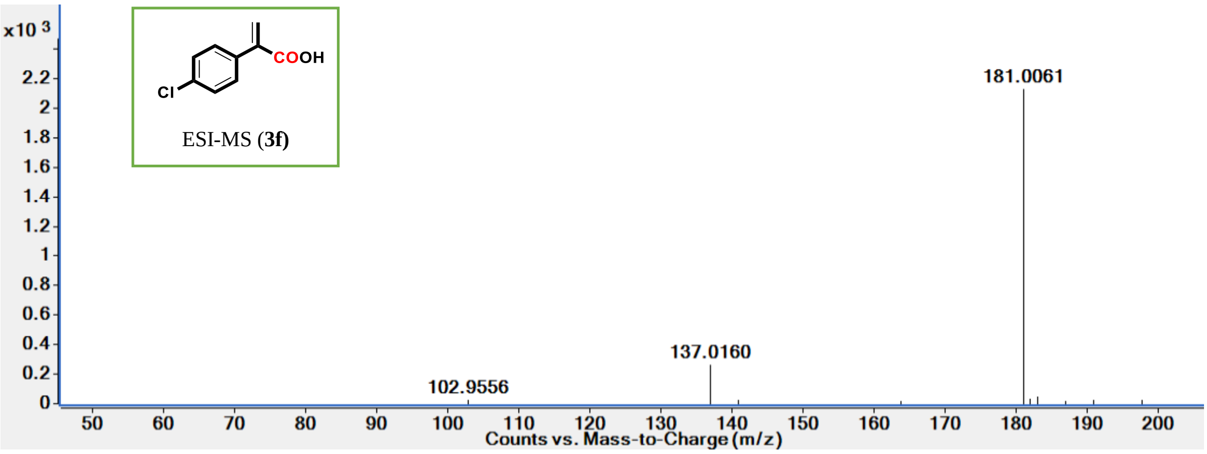

ESI-MS (**3f**)

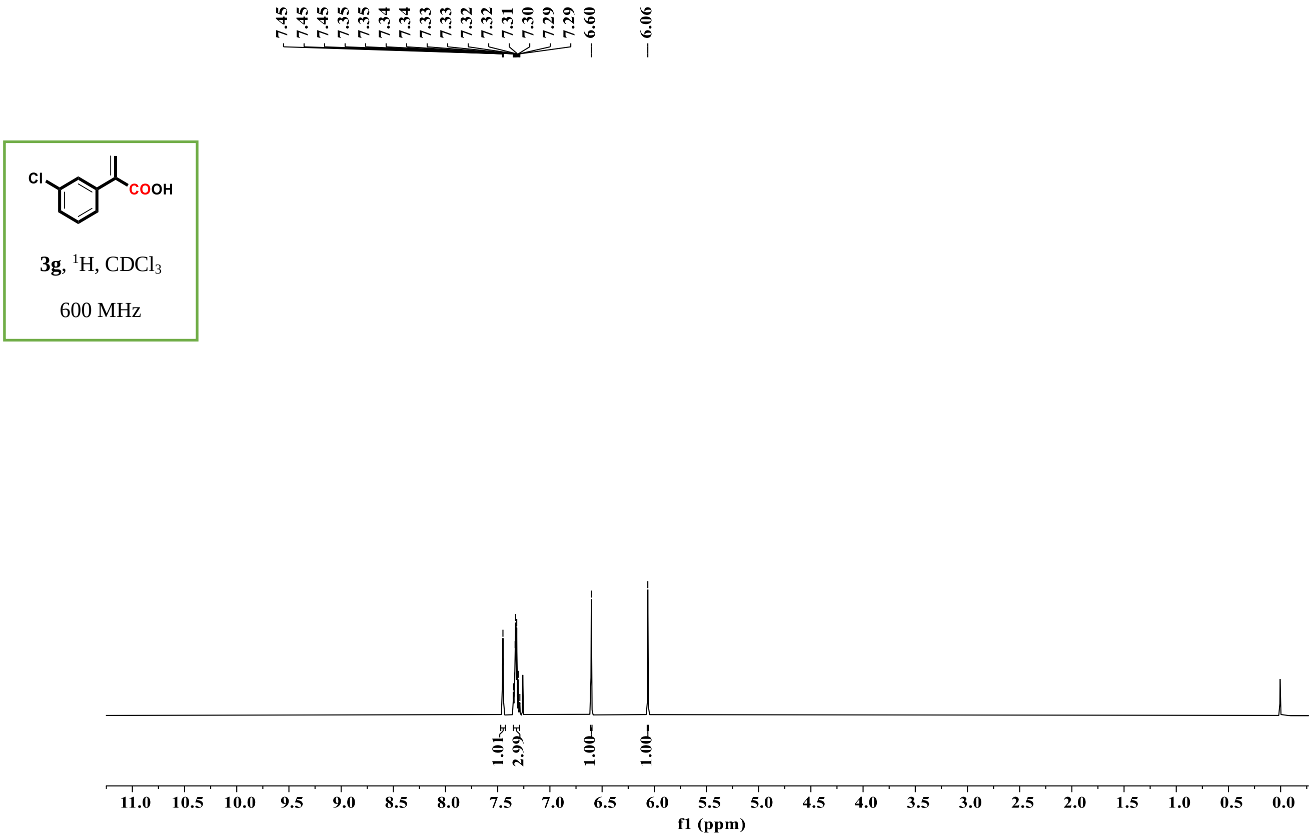

3g, ^{1}H, $CDCl_3$

600 MHz

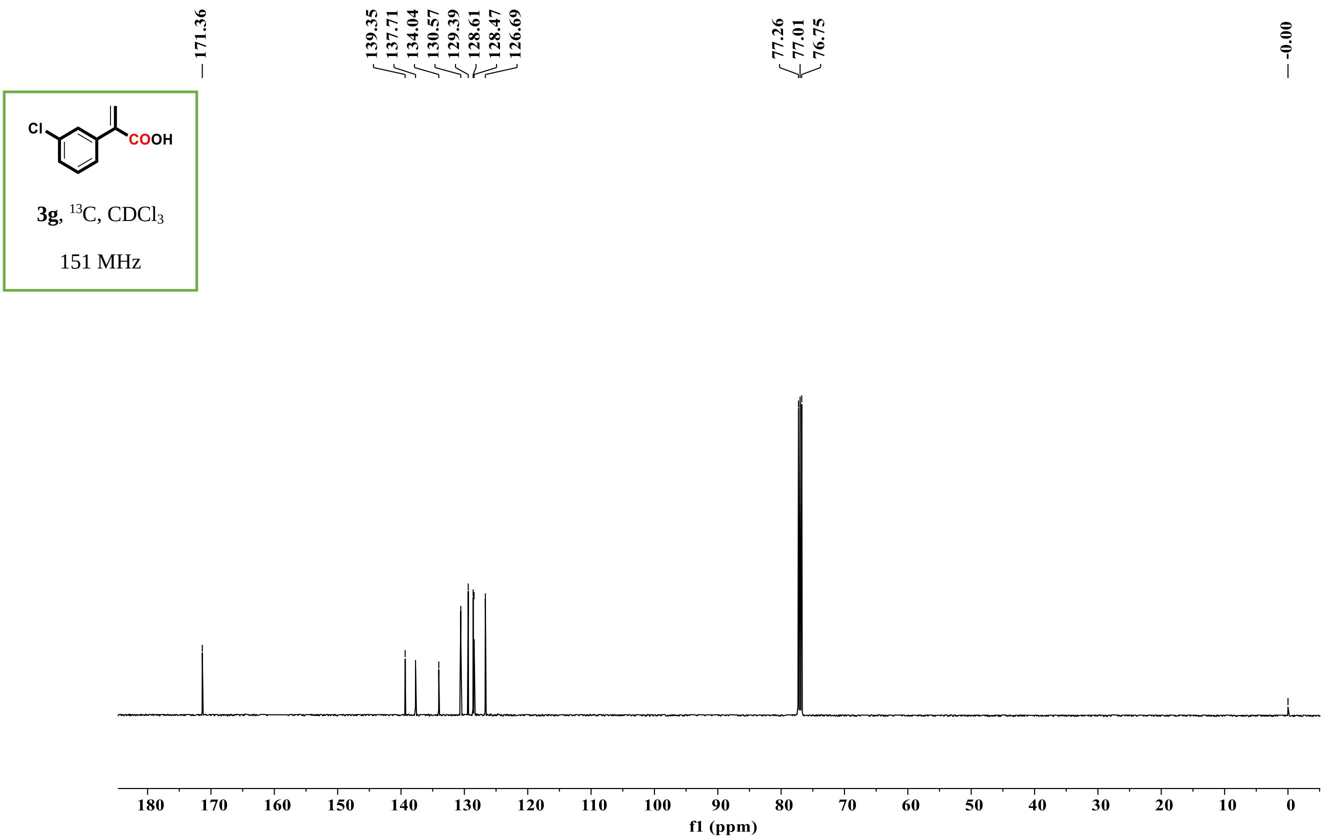

171.36
139.35
137.71
134.04
130.57
129.39
128.61
128.47
126.69
77.26
77.01
76.75
-0.00
Cl
COOH
3g, 13C, CDCl3
151 MHz
180
170
160
150
140
130
120
110
100
90
80
70
60
50
40
30
20
10
0
f1 (ppm)

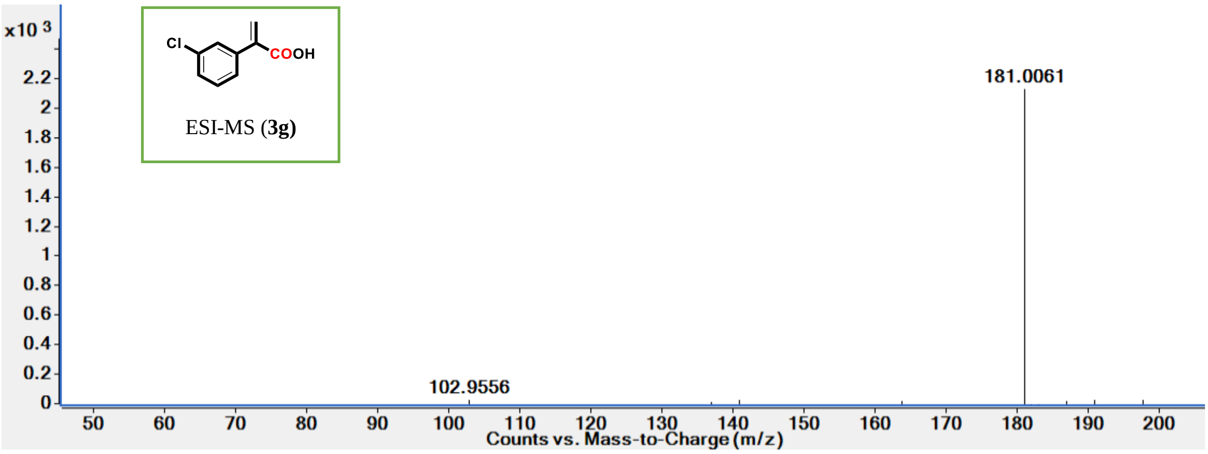

x10 3
2.2
2
1.8
1.6
1.4
1.2
1
0.8
0.6
0.4
0.2
0
Cl
COOH
ESI-MS (3g)
181.0061
102.9556
50
60
70
80
90
100
110
120
130
140
150
160
170
180
190
200
Counts vs. Mass-to-Charge (m/z)

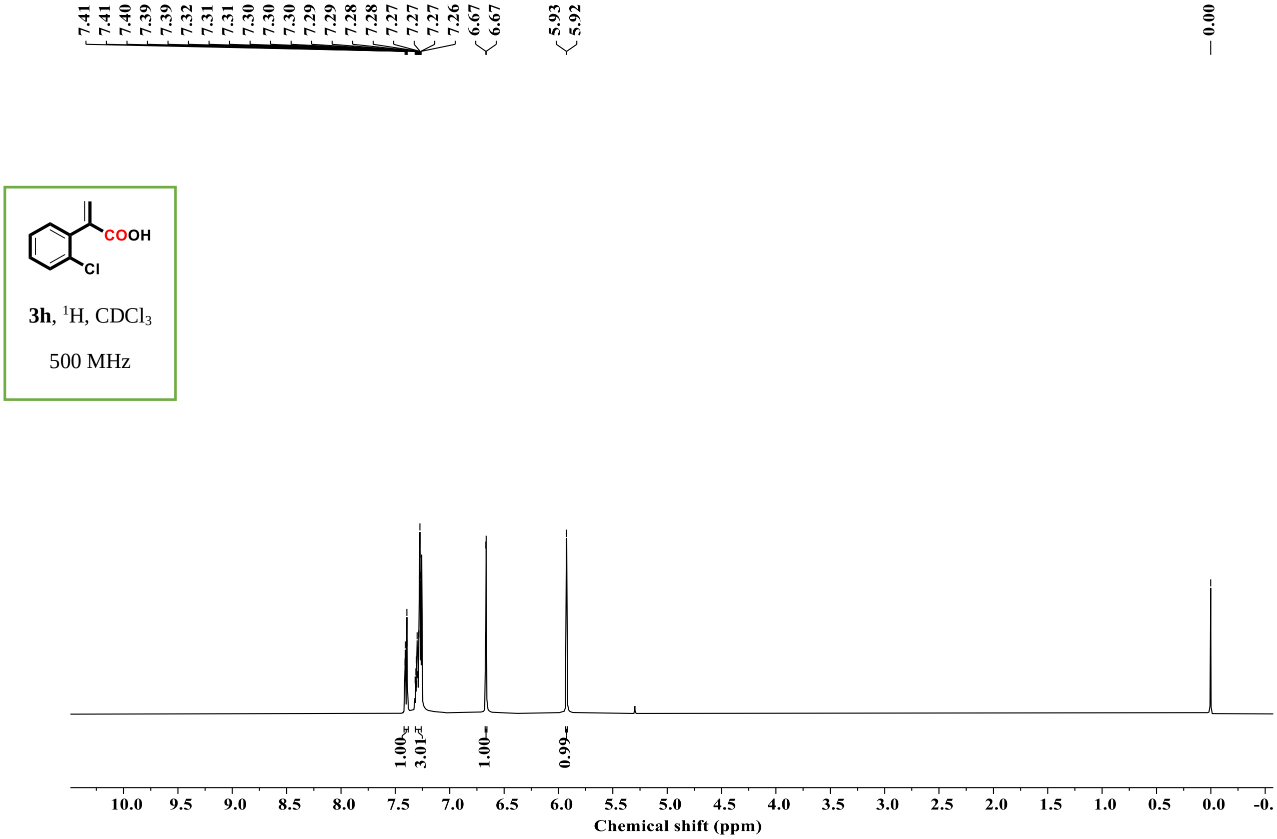
7.41
7.41
7.40
7.39
7.39
7.32
7.31
7.31
7.30
7.30
7.30
7.29
7.29
7.28
7.28
7.27
7.27
7.27
7.26
6.67
6.67
5.93
5.92
0.00
COOH
Cl
3h, ^{1}H, $CDCl_3$
500 MHz
1.00
3.01
1.00
0.99
10.0 9.5 9.0 8.5 8.0 7.5 7.0 6.5 6.0 5.5 5.0 4.5 4.0 3.5 3.0 2.5 2.0 1.5 1.0 0.5 0.0 -0.
Chemical shift (ppm)

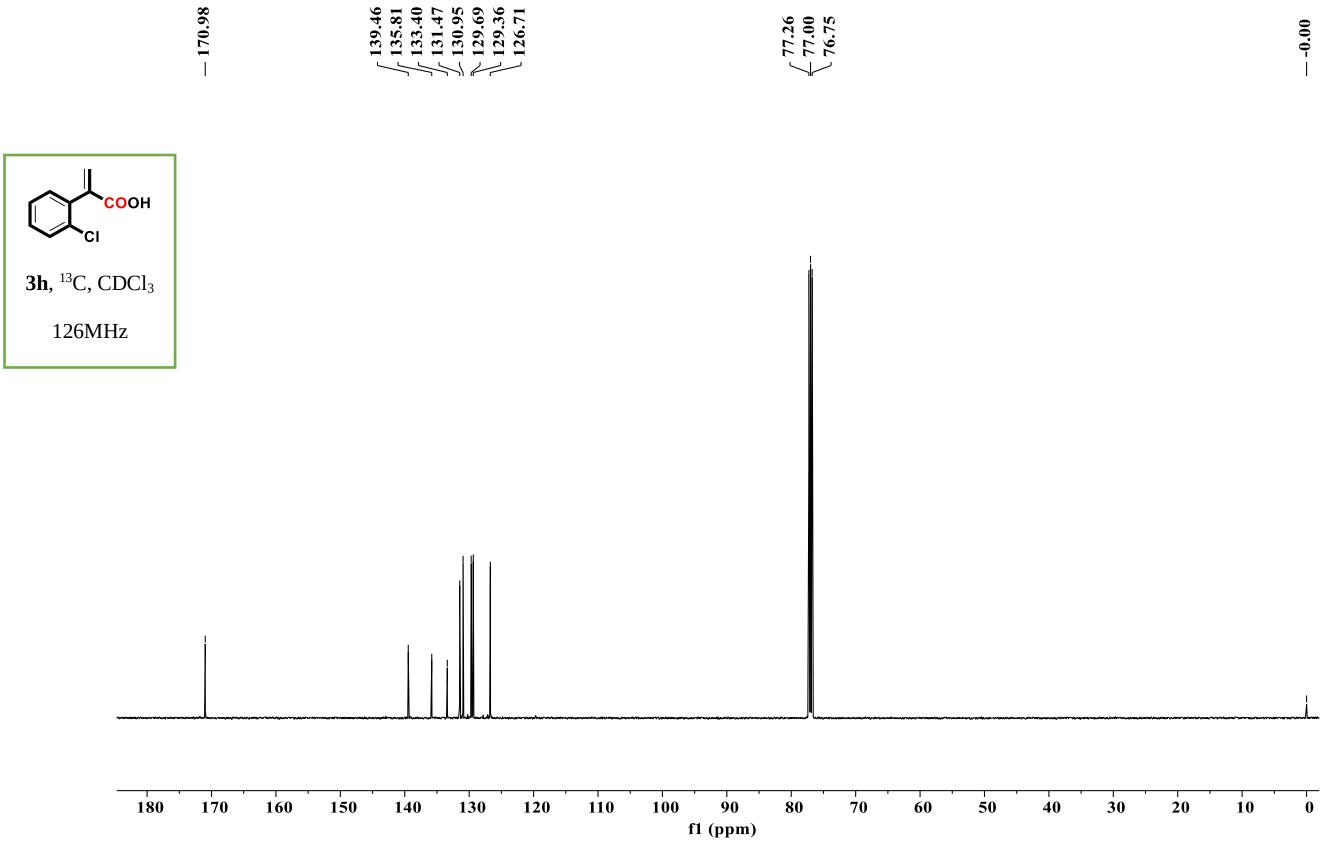
170.98
139.46
135.81
133.40
131.47
130.95
129.69
129.36
126.71
77.26
77.00
76.75
-0.00
COOH
Cl
3h, ^{13}C, $CDCl_3$
126MHz
180 170 160 150 140 130 120 110 100 90 80 70 60 50 40 30 20 10 0
f1 (ppm)

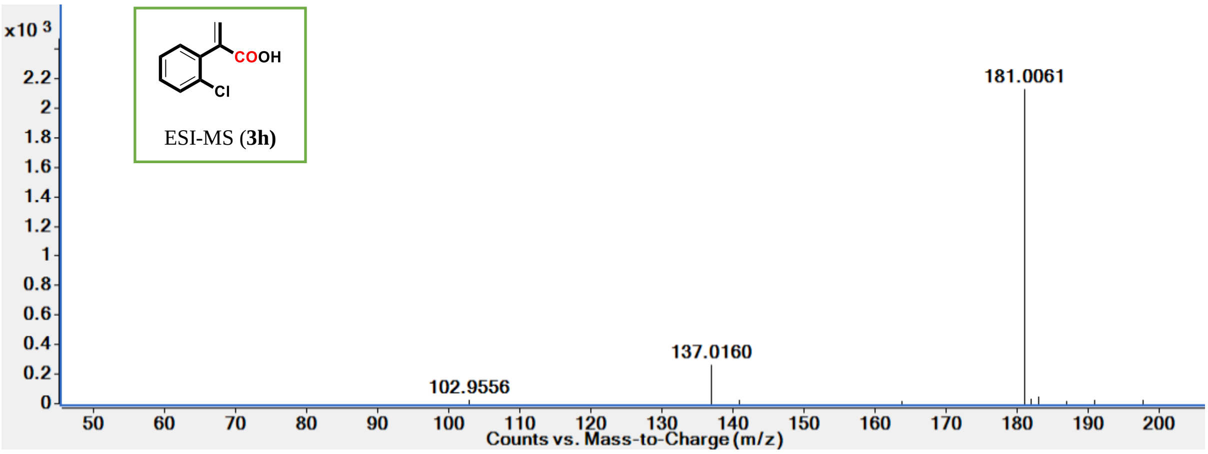
x10 3
2.2
2
1.8
1.6
1.4
1.2
1
0.8
0.6
0.4
0.2
0
COOH
Cl
ESI-MS (3h)
181.0061
137.0160
102.9556
50
60
70
80
90
100
110
120
130
140
150
160
170
180
190
200
Counts vs. Mass-to-Charge (m/z)

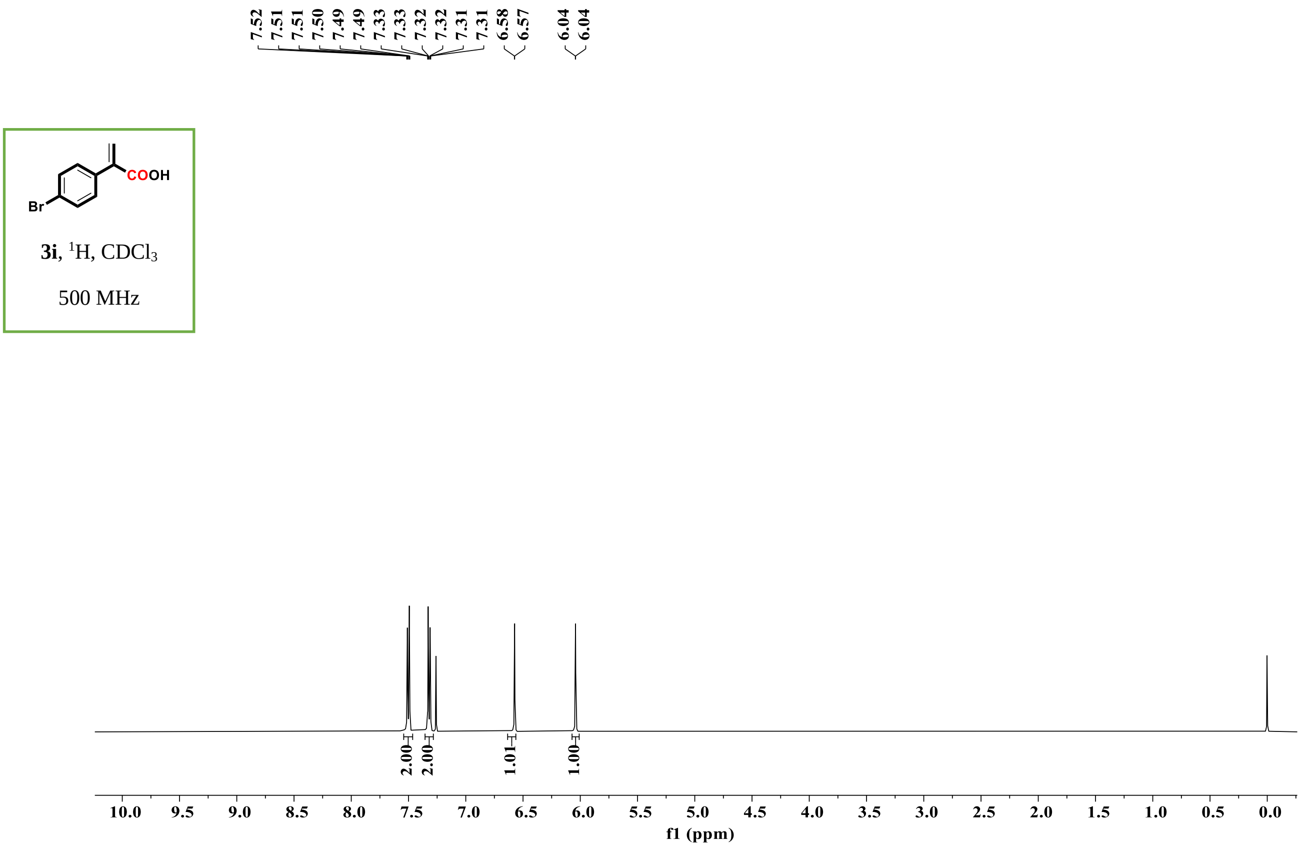
7.52
7.51
7.51
7.50
7.49
7.49
7.33
7.33
7.32
7.32
7.31
7.31
6.58
6.57
6.04
6.04
COOH
Br
3i, ¹H, CDCl3
500 MHz
2.00
2.00
1.01
1.00
10.0
9.5
9.0
8.5
8.0
7.5
7.0
6.5
6.0
5.5
5.0
4.5
4.0
3.5
3.0
2.5
2.0
1.5
1.0
0.5
0.0
f1 (ppm)

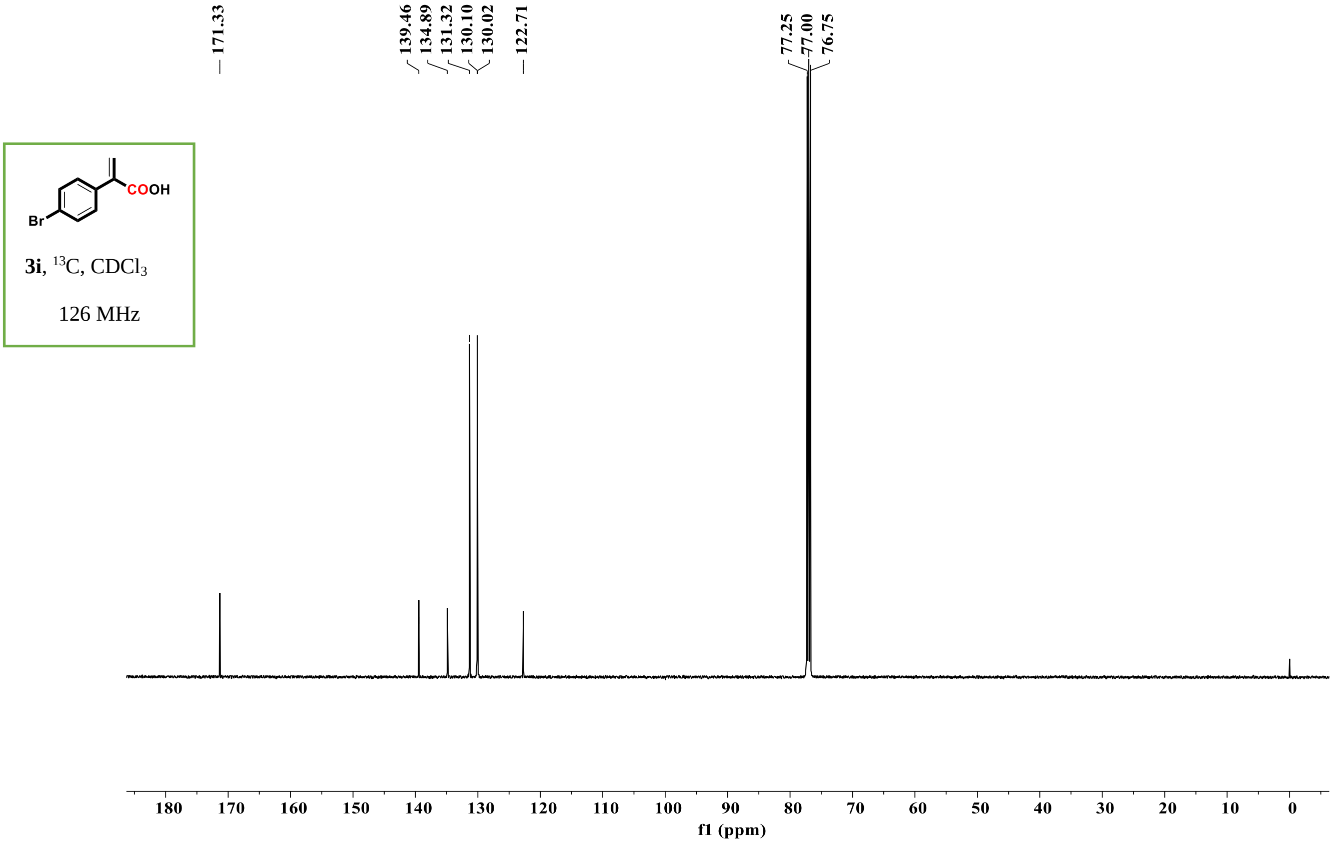
171.33
139.46
134.89
131.32
130.10
130.02
122.71
77.25
77.00
76.75
Br
COOH
3i, 13C, CDCl3
126 MHz
180 170 160 150 140 130 120 110 100 90 80 70 60 50 40 30 20 10 0
f1 (ppm)

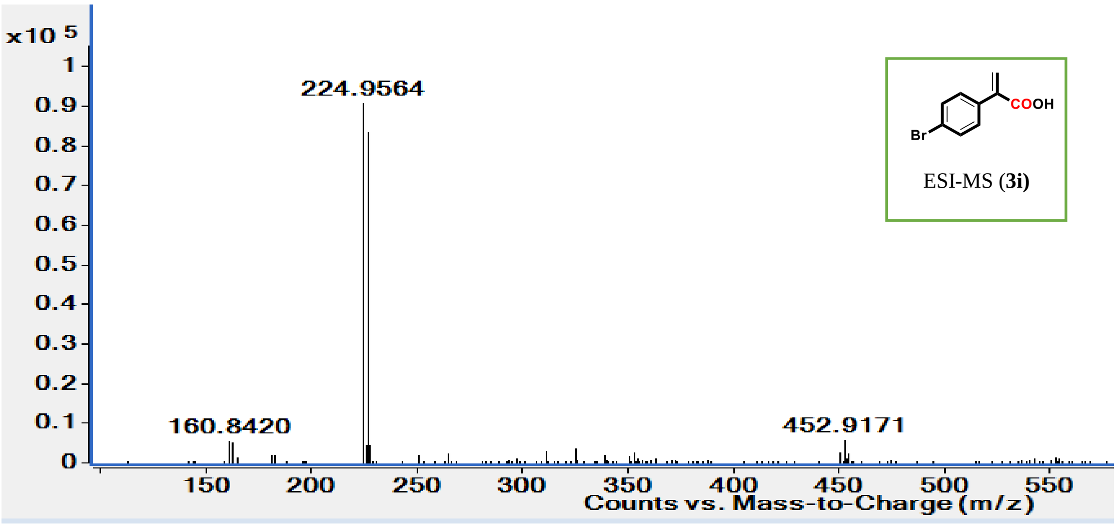
x10 5
1
0.9
0.8
0.7
0.6
0.5
0.4
0.3
0.2
0.1
0
224.9564
160.8420
452.9171
Br
COOH
ESI-MS (3i)
150 200 250 300 350 400 450 500 550
Counts vs. Mass-to-Charge (m/z)

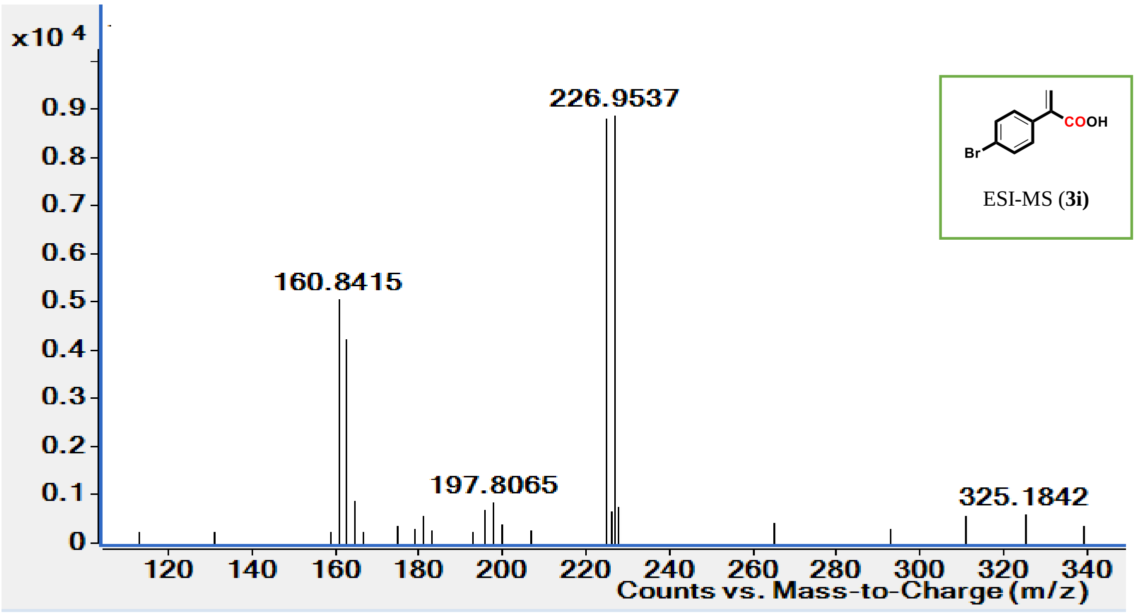
x10 4
0.9
0.8
0.7
0.6
0.5
0.4
0.3
0.2
0.1
0
226.9537
160.8415
197.8065
325.1842
120 140 160 180 200 220 240 260 280 300 320 340
Counts vs. Mass-to-Charge (m/z)
Br
COOH
ESI-MS (3i)

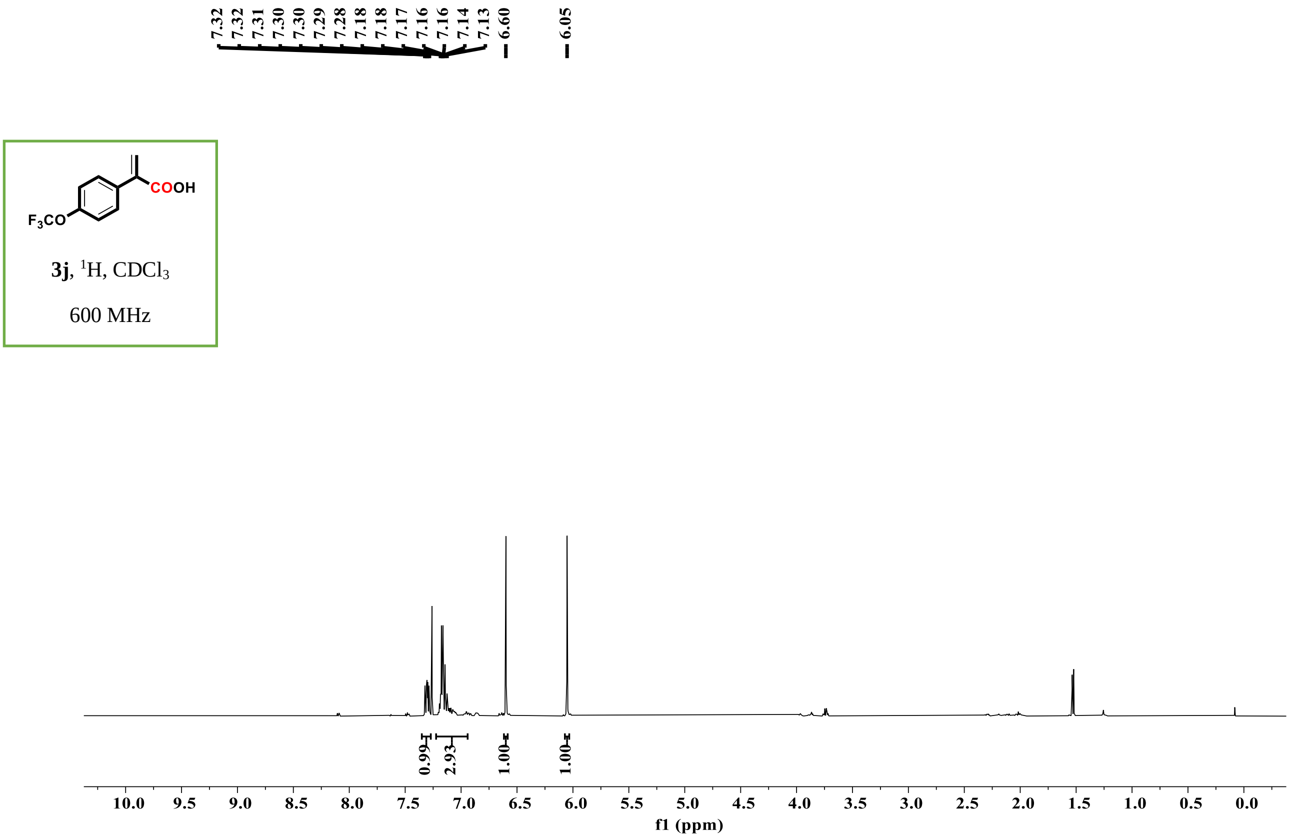
7.32
7.32
7.31
7.30
7.30
7.29
7.28
7.18
7.18
7.17
7.16
7.16
7.14
7.13
6.60
6.05
F3CO
COOH
3j, 1H, CDCl3
600 MHz
0.99
2.93
1.00
1.00
10.0 9.5 9.0 8.5 8.0 7.5 7.0 6.5 6.0 5.5 5.0 4.5 4.0 3.5 3.0 2.5 2.0 1.5 1.0 0.5 0.0
f1 (ppm)

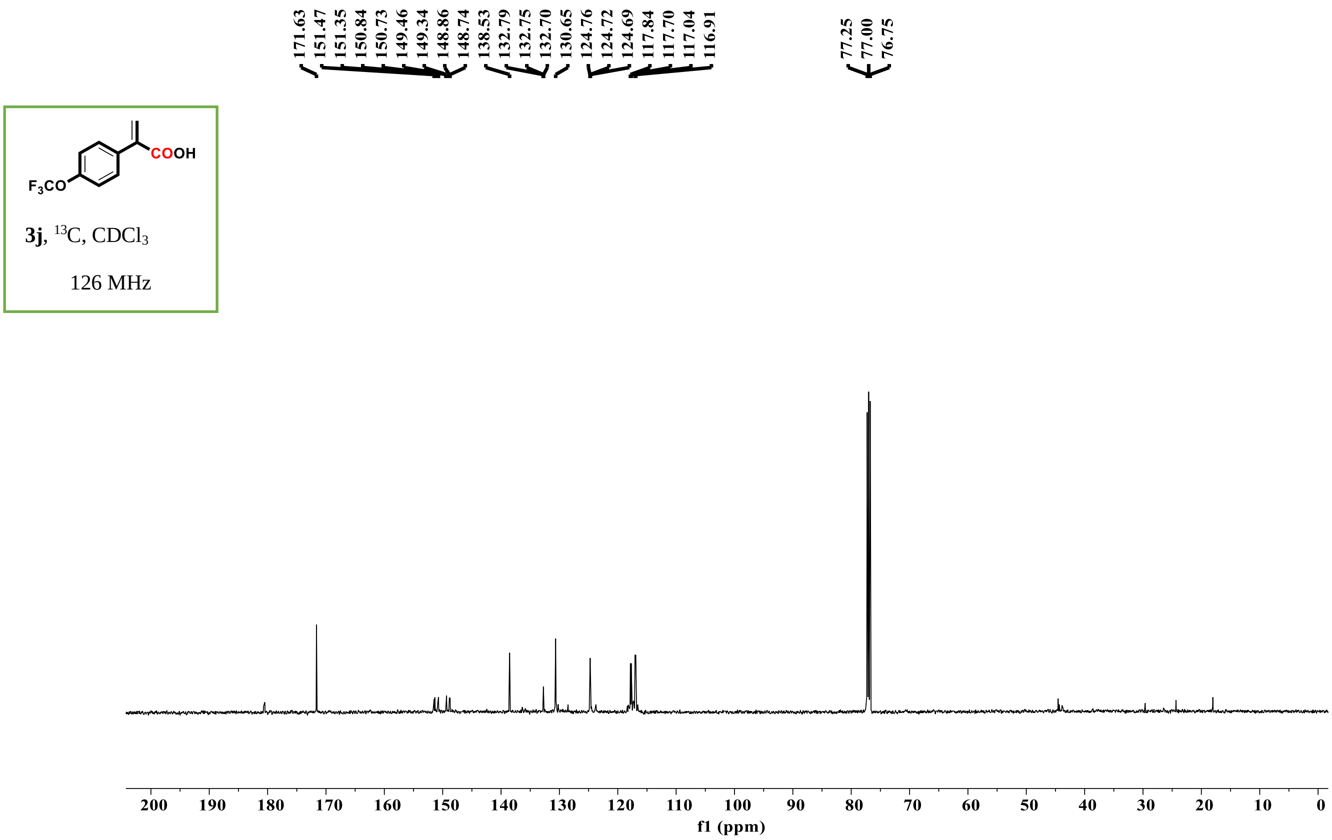
171.63
151.47
151.35
150.84
150.73
149.46
149.34
148.86
148.74
138.53
132.79
132.75
132.70
130.65
124.76
124.72
124.69
117.84
117.70
117.04
116.91
77.25
77.00
76.75
COOH
F3CO
3j, 13C, CDCl3
126 MHz
f1 (ppm)

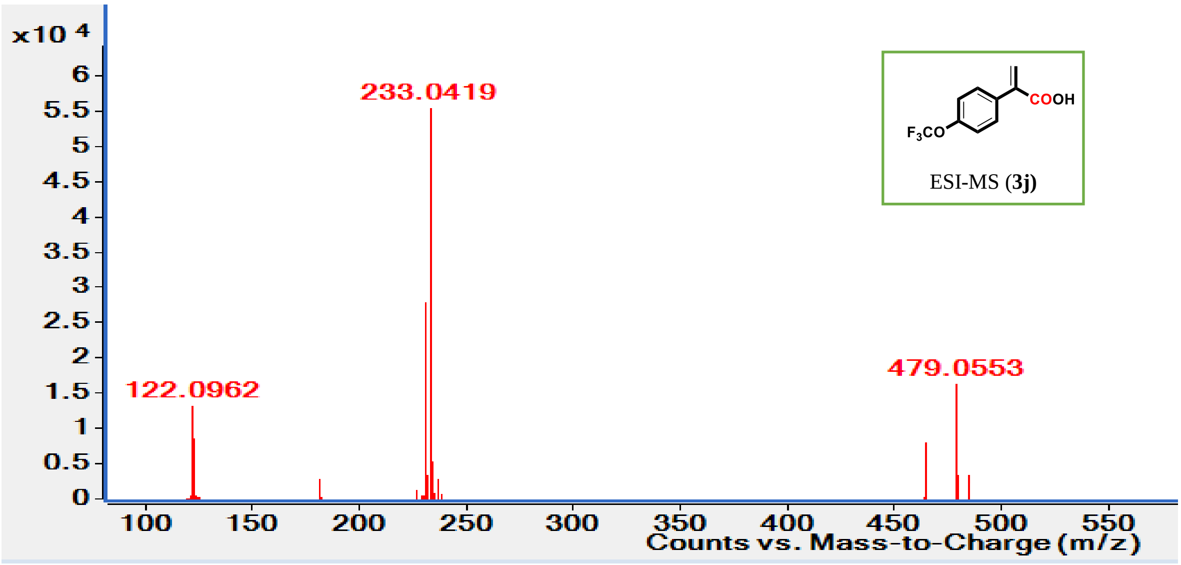
x10 4
233.0419
122.0962
479.0553
COOH
F3CO
ESI-MS (3j)
Counts vs. Mass-to-Charge (m/z)

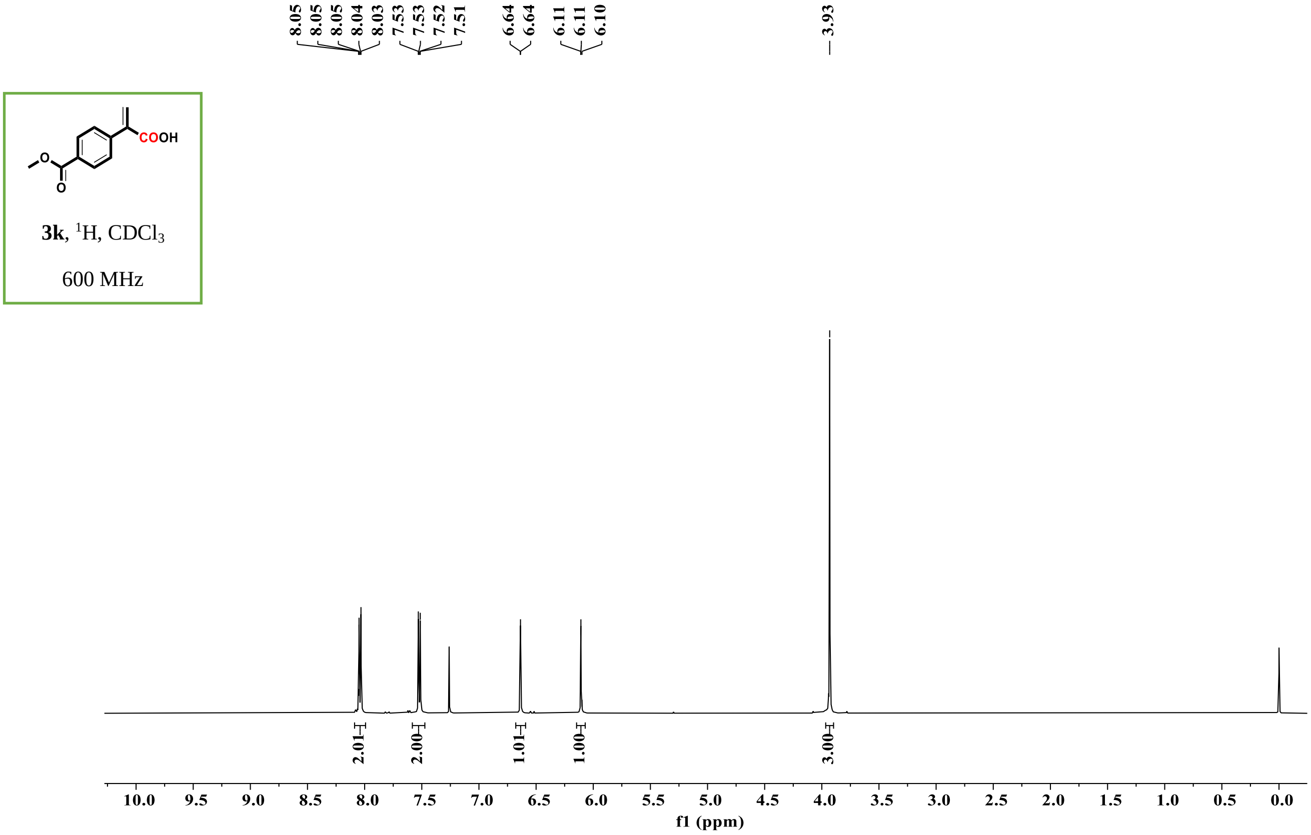
COOH
O
O
3k, ^{1}H, CDCl$_3$
600 MHz
8.05
8.05
8.05
8.04
8.03
7.53
7.53
7.52
7.51
6.64
6.64
6.11
6.11
6.10
3.93
2.01
2.00
1.01
1.00
3.00
10.0 9.5 9.0 8.5 8.0 7.5 7.0 6.5 6.0 5.5 5.0 4.5 4.0 3.5 3.0 2.5 2.0 1.5 1.0 0.5 0.0
f1 (ppm)

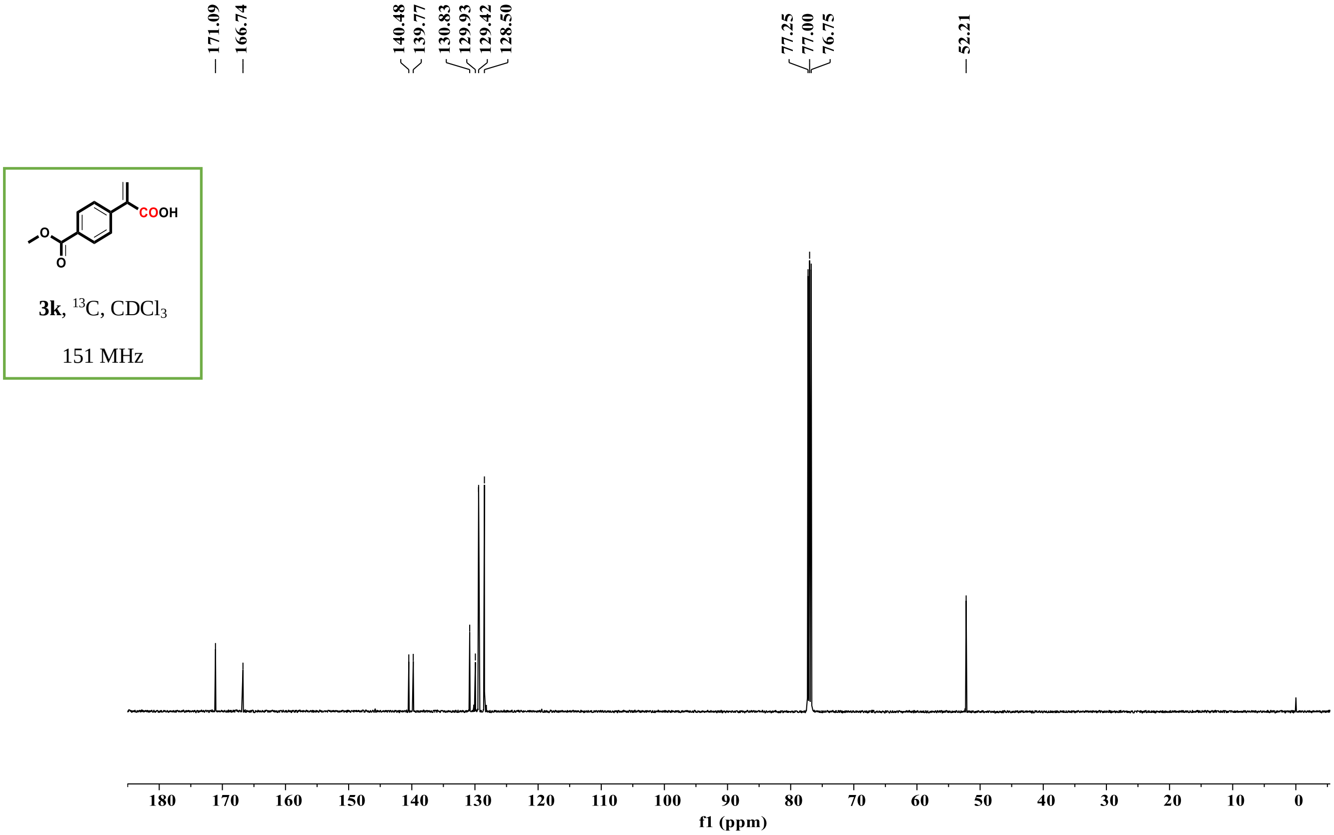
COOH
O
O
3k, ^{13}C, CDCl$_3$
151 MHz
171.09
166.74
140.48
139.77
130.83
129.93
129.42
128.50
77.25
77.00
76.75
52.21
180 170 160 150 140 130 120 110 100 90 80 70 60 50 40 30 20 10 0
f1 (ppm)

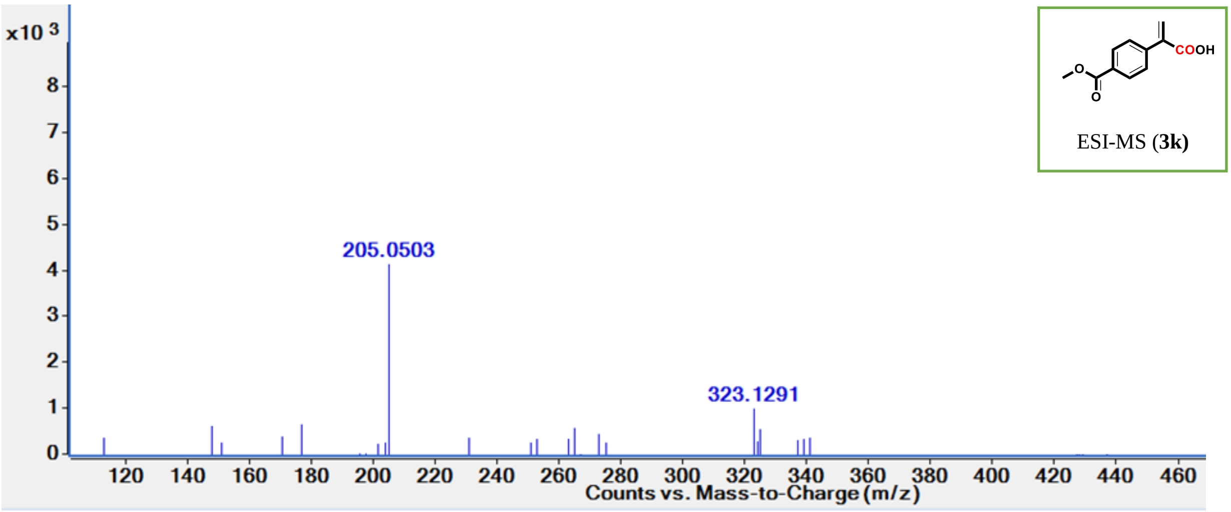

ESI-MS (**3k**)

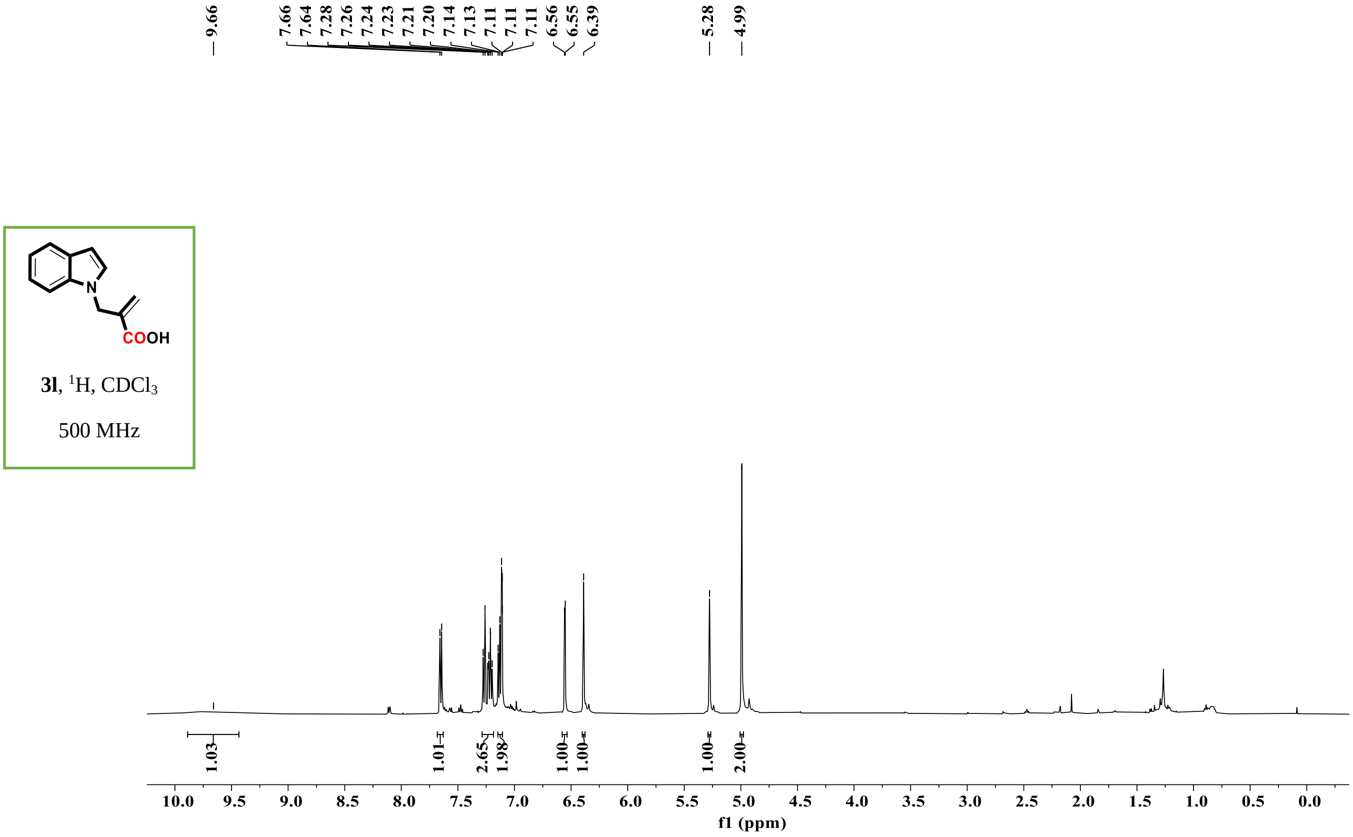

3l, ^{1}H, $CDCl_3$

500 MHz

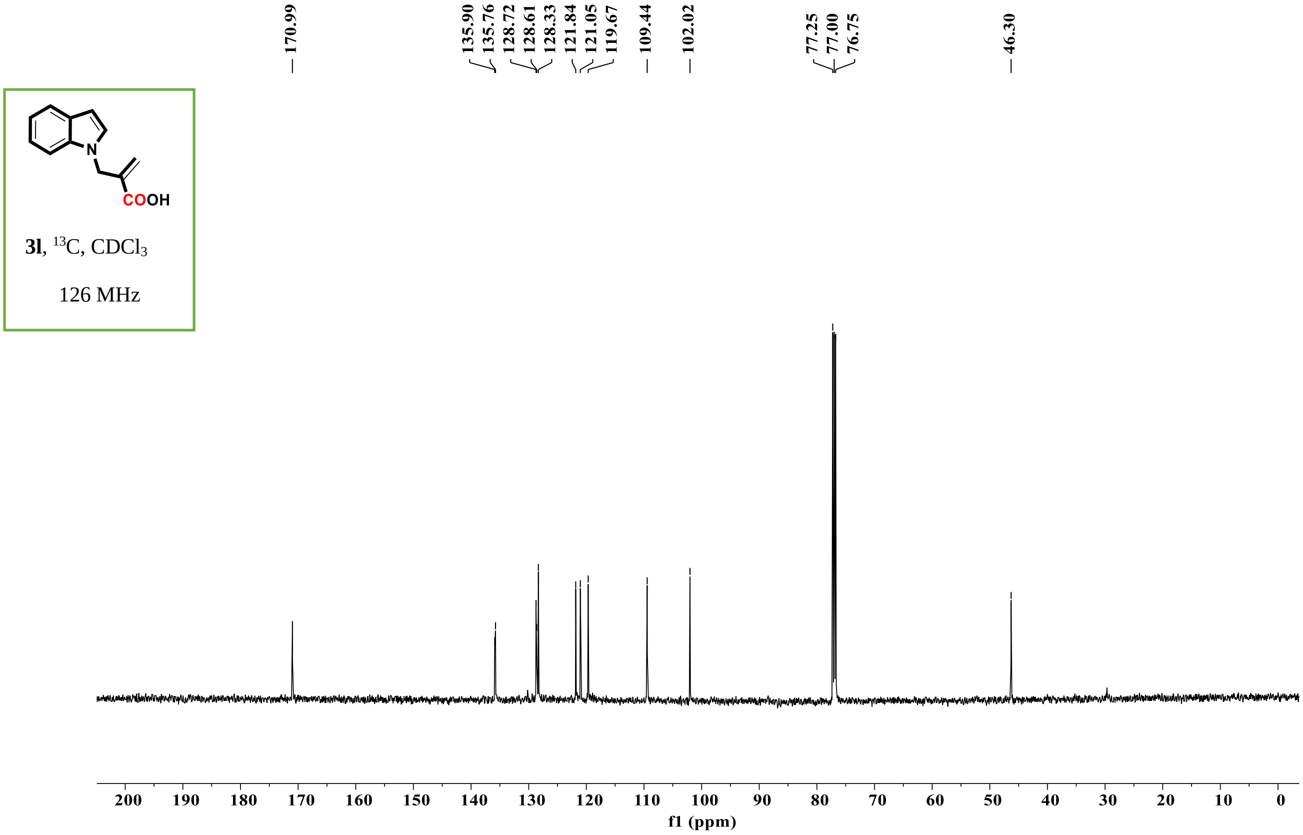

170.99
135.90
135.76
128.72
128.61
128.33
121.84
121.05
119.67
109.44
102.02
77.25
77.00
76.75
46.30
COOH
N
3l, ^{13}C, CDCl$_3$
126 MHz
200 190 180 170 160 150 140 130 120 110 100 90 80 70 60 50 40 30 20 10 0
f1 (ppm)

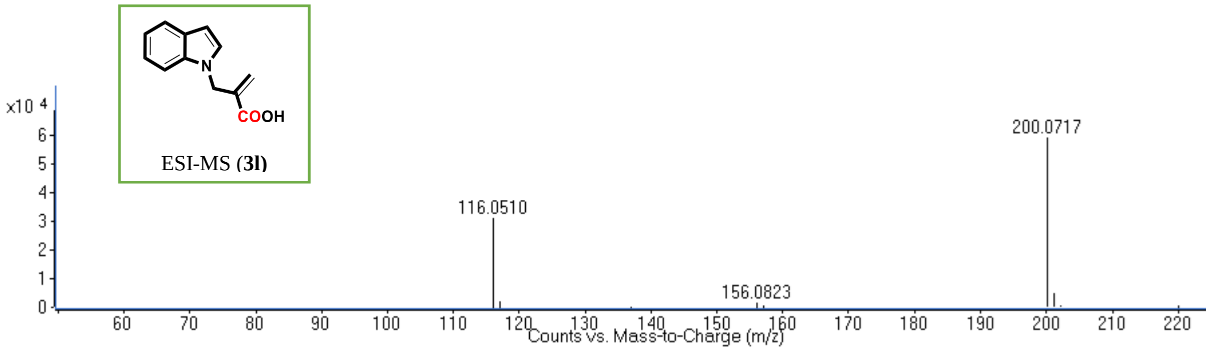

N
COOH
ESI-MS (3l)
x10 4
200.0717
116.0510
156.0823
60 70 80 90 100 110 120 130 140 150 160 170 180 190 200 210 220
Counts vs. Mass-to-Charge (m/z)

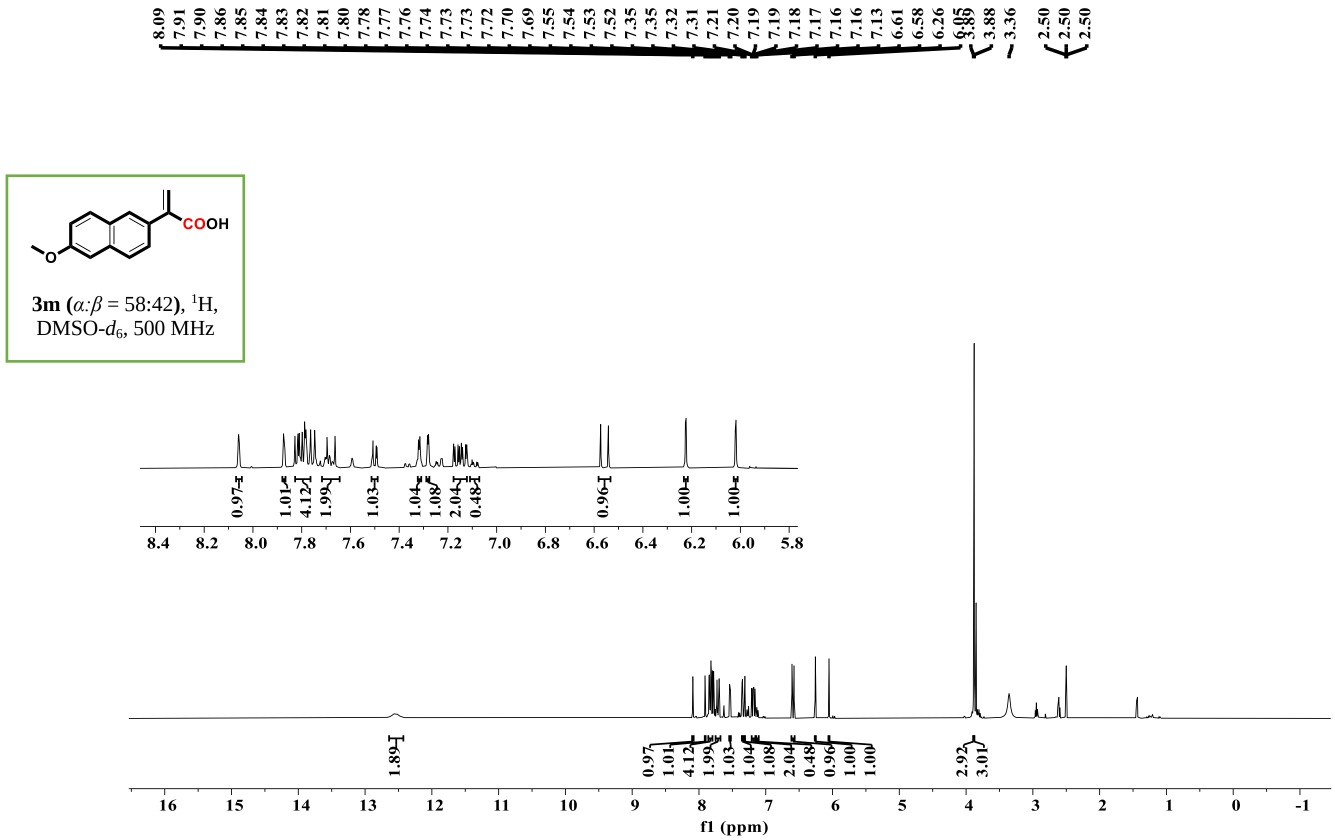
COOH
3m (α:β = 58:42), 1H, DMSO-d6, 500 MHz
f1 (ppm)

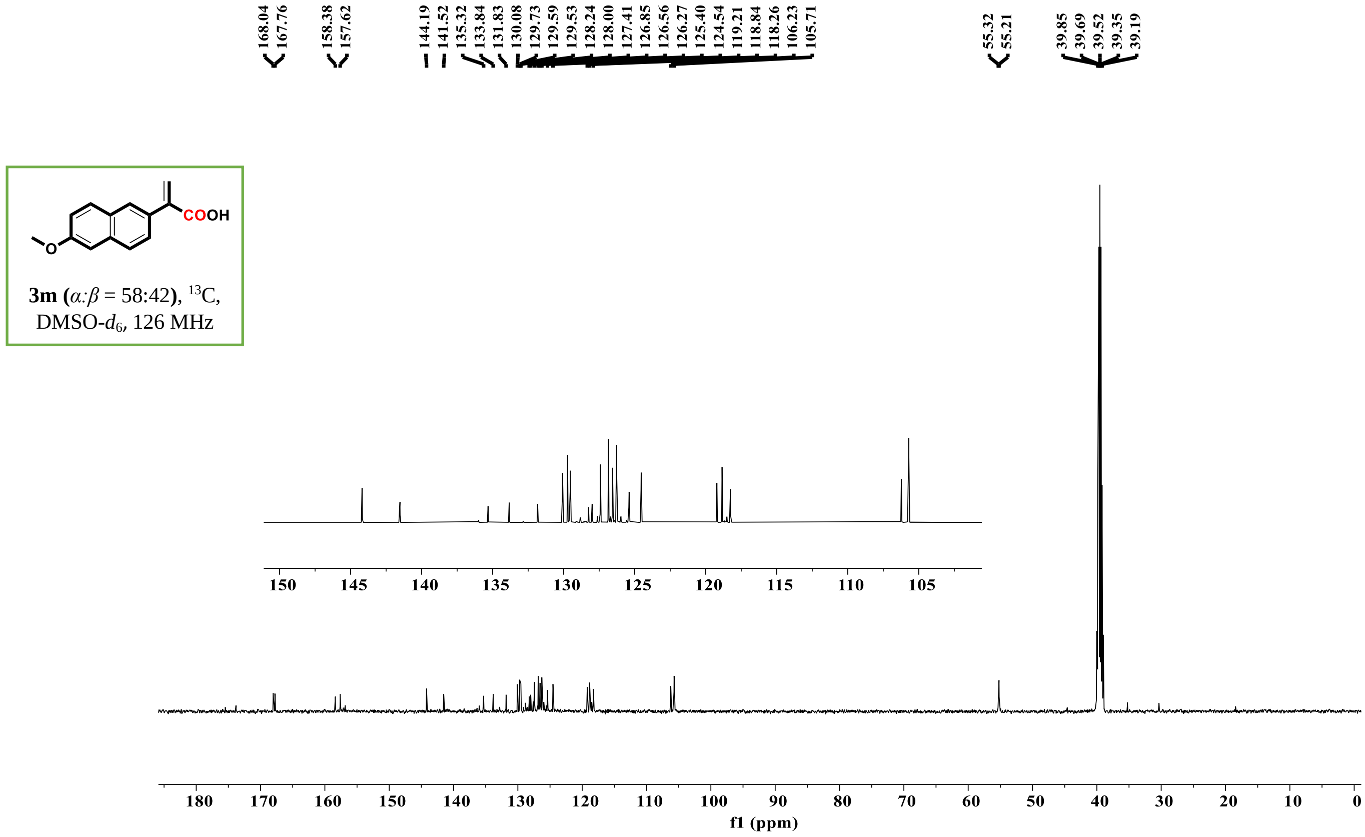
COOH
3m (α:β = 58:42), 13C, DMSO-d6, 126 MHz
f1 (ppm)

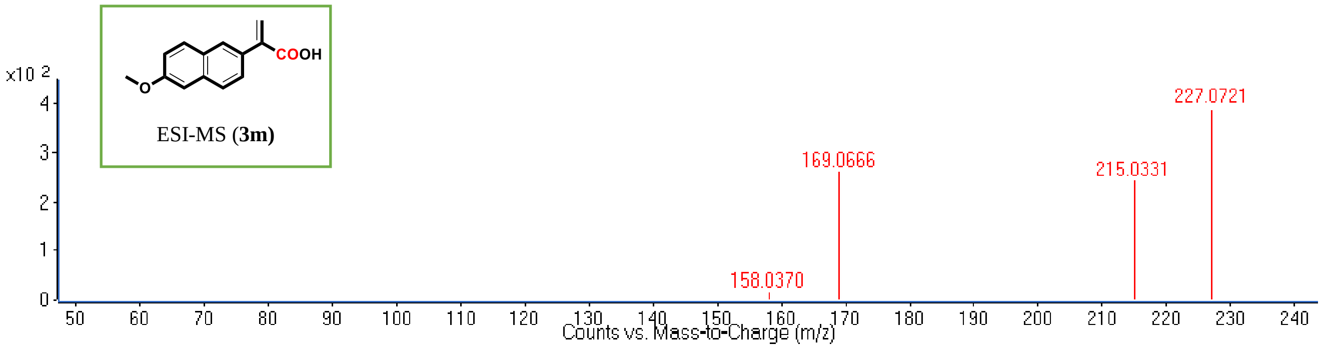

COOH
ESI-MS (3m)
x10 2
227.0721
169.0666
215.0331
158.0370
Counts vs. Mass-to-Charge (m/z)

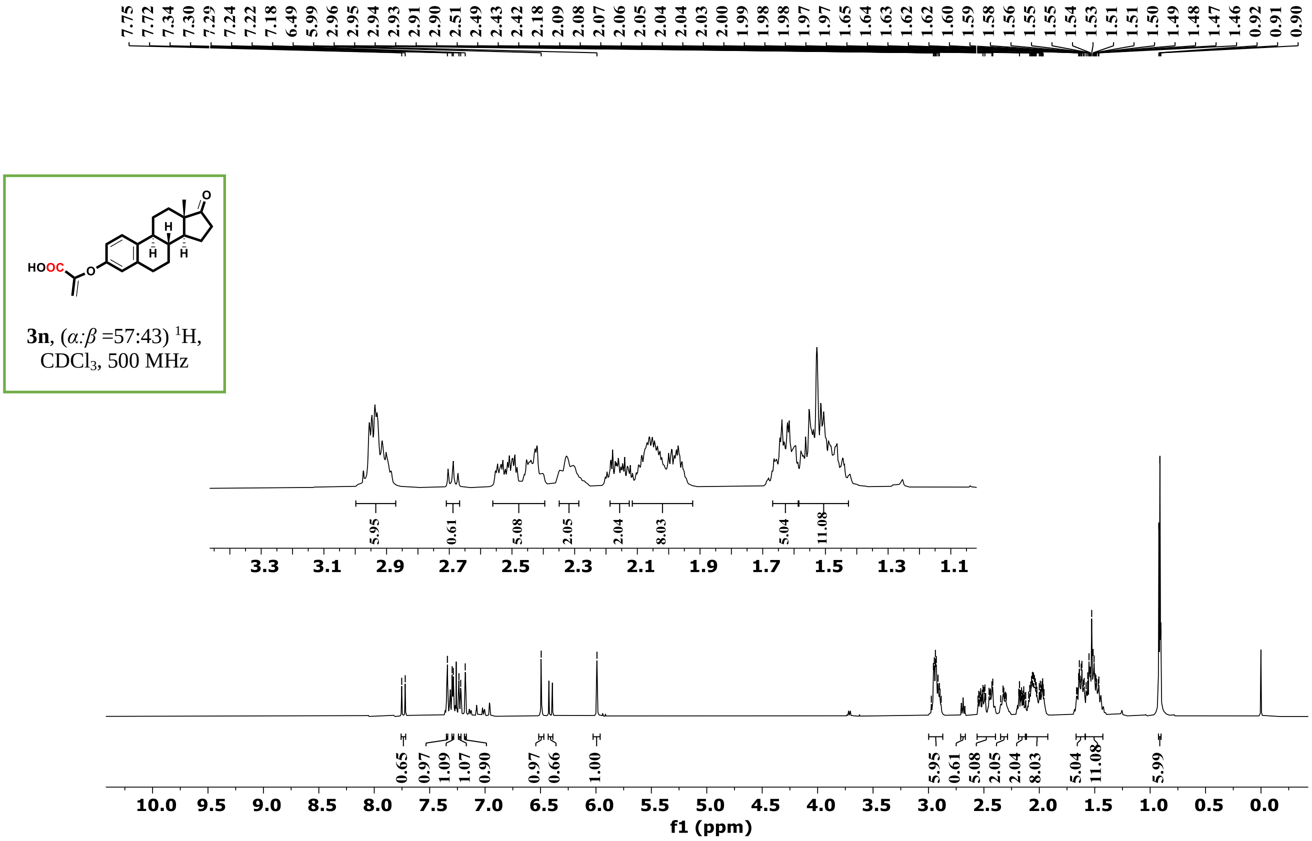

HOOC
3n, (α:β =57:43) ¹H, CDCl₃, 500 MHz
f1 (ppm)

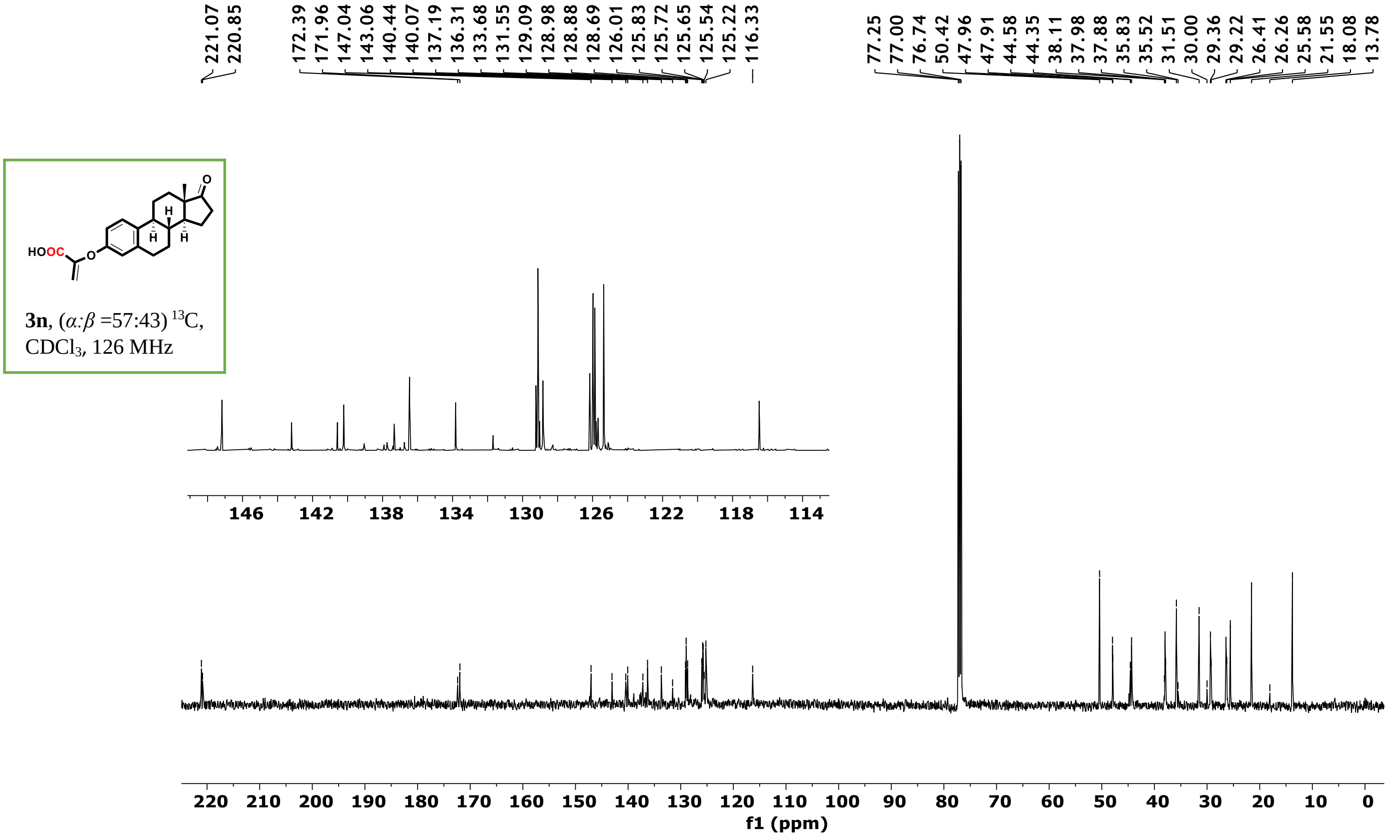

3n, (*α*:*β* =57:43) ^{13}C, $CDCl_3$, 126 MHz

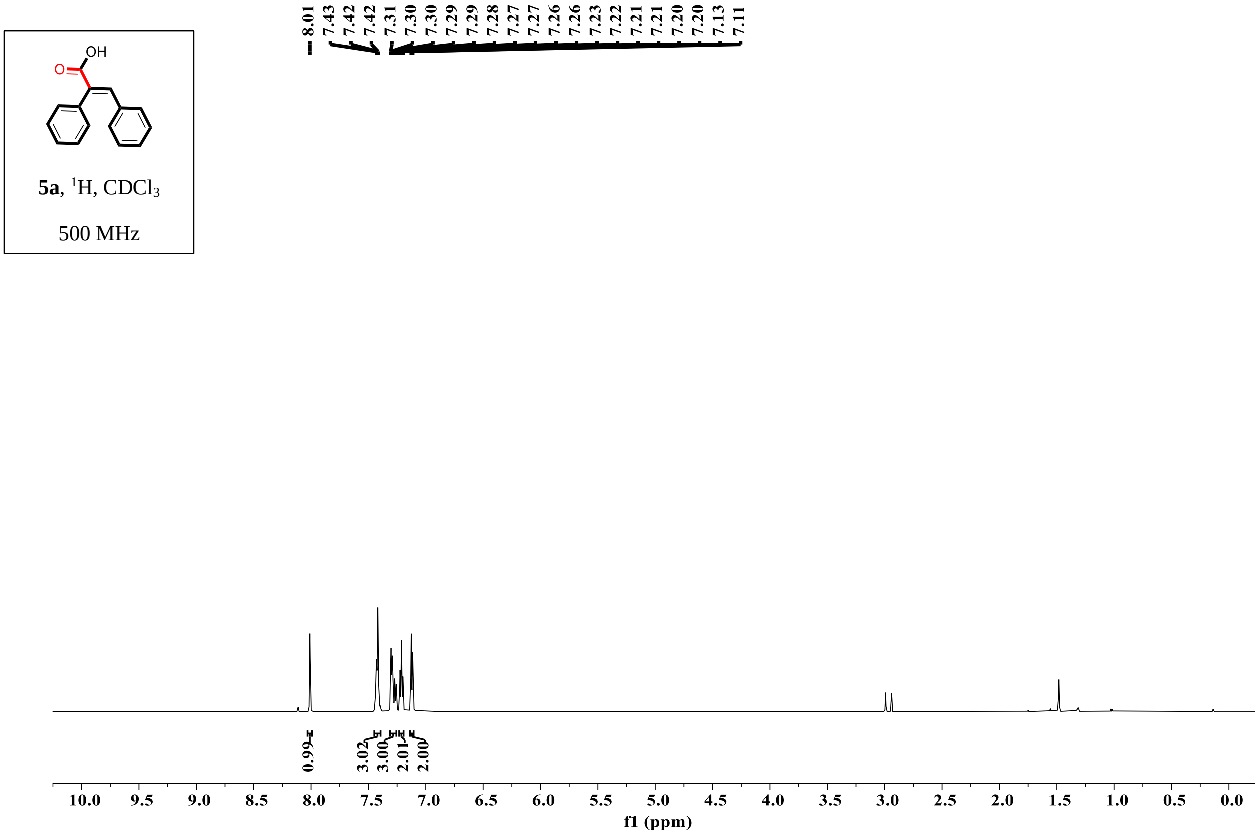

5a, ^{1}H, $CDCl_3$

500 MHz

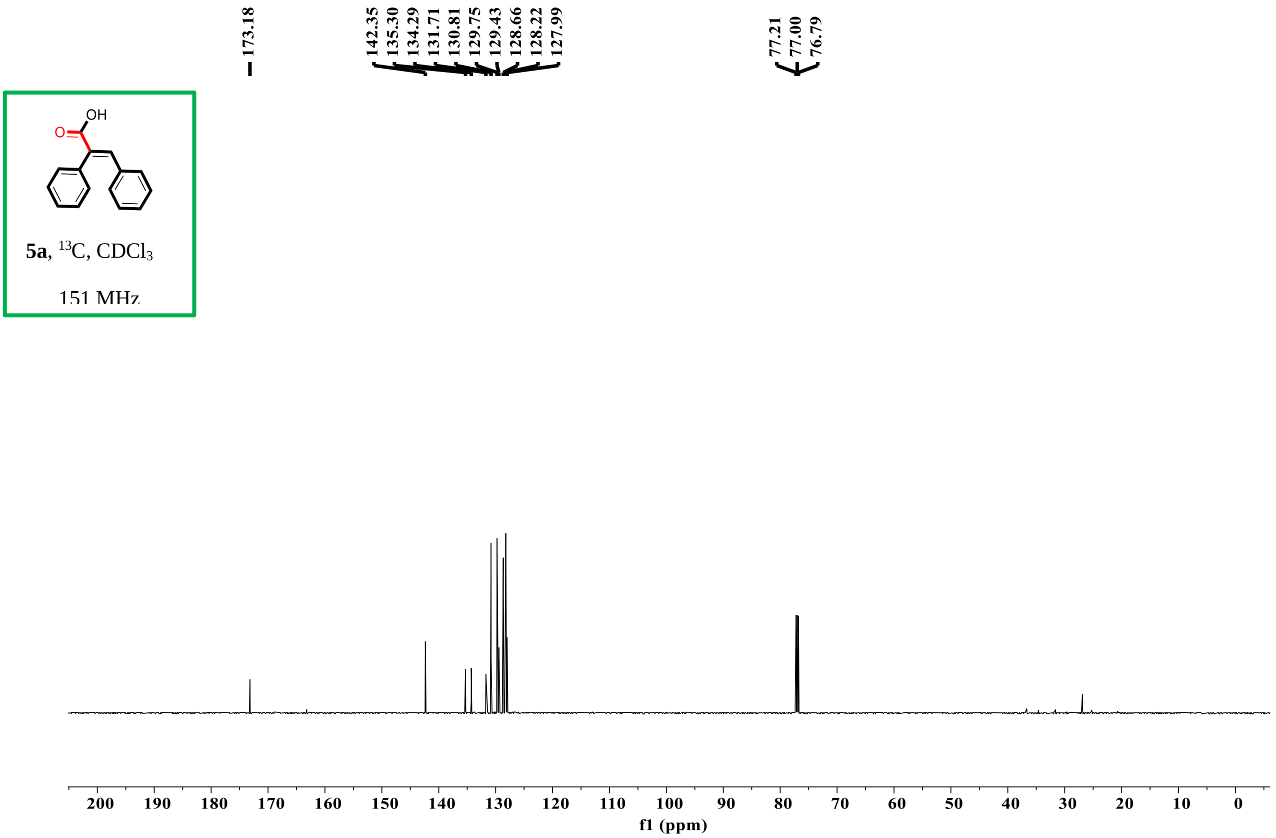
173.18
142.35
135.30
134.29
131.71
130.81
129.75
129.43
128.66
128.22
127.99
77.21
77.00
76.79
OH
O
5a, ¹³C, CDCl₃
151 MHz
200 190 180 170 160 150 140 130 120 110 100 90 80 70 60 50 40 30 20 10 0
f1 (ppm)

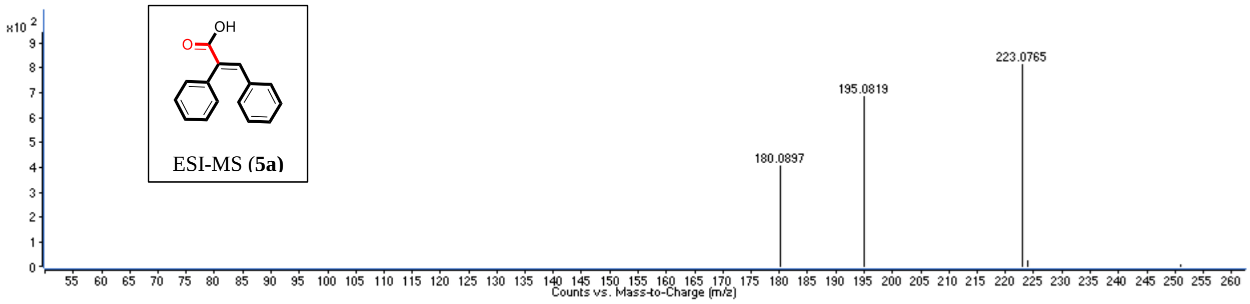
x10 2
OH
O
ESI-MS (5a)
180.0897
195.0819
223.0765
Counts vs. Mass-to-Charge (m/z)

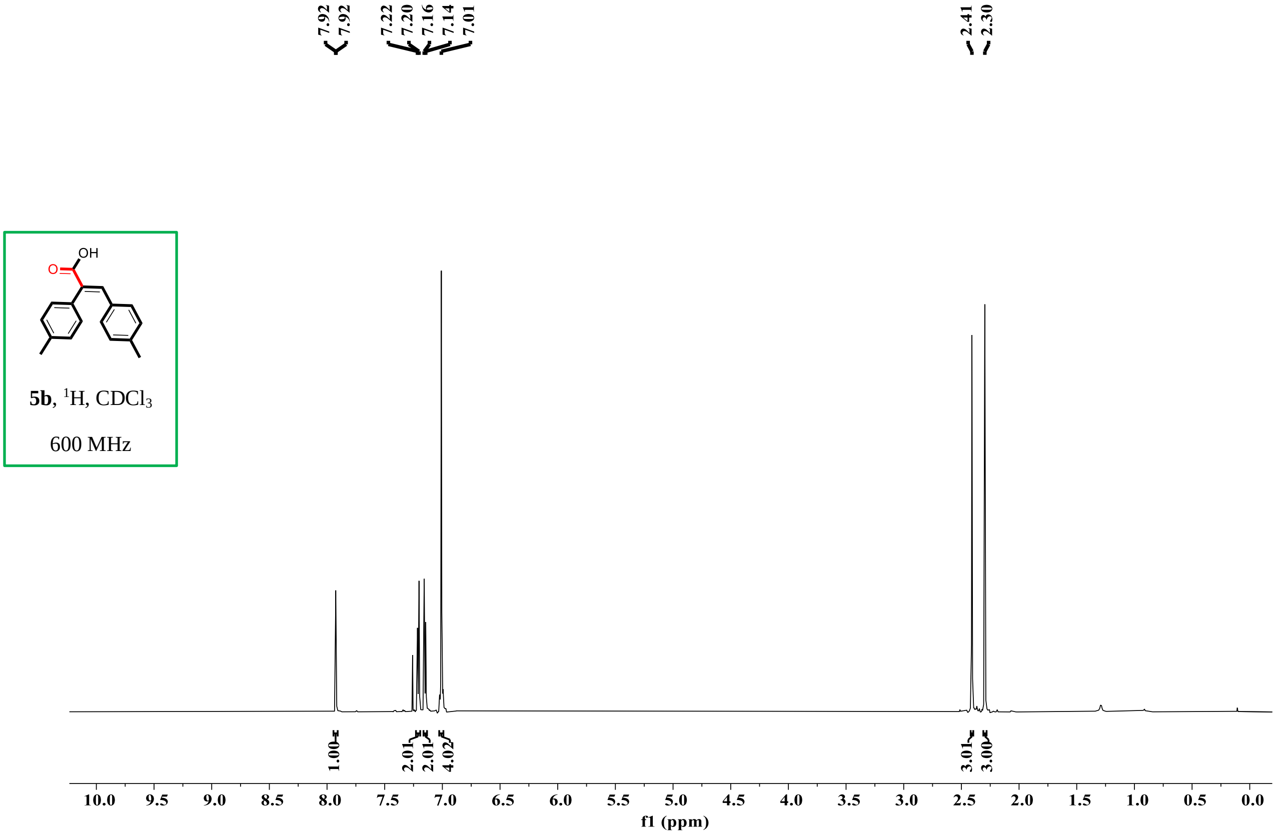
7.92
7.92
7.22
7.20
7.16
7.14
7.01
2.41
2.30
OH
O
5b, 1H, CDCl3
600 MHz
1.00
2.01
2.01
4.02
3.01
3.00
10.0 9.5 9.0 8.5 8.0 7.5 7.0 6.5 6.0 5.5 5.0 4.5 4.0 3.5 3.0 2.5 2.0 1.5 1.0 0.5 0.0
f1 (ppm)

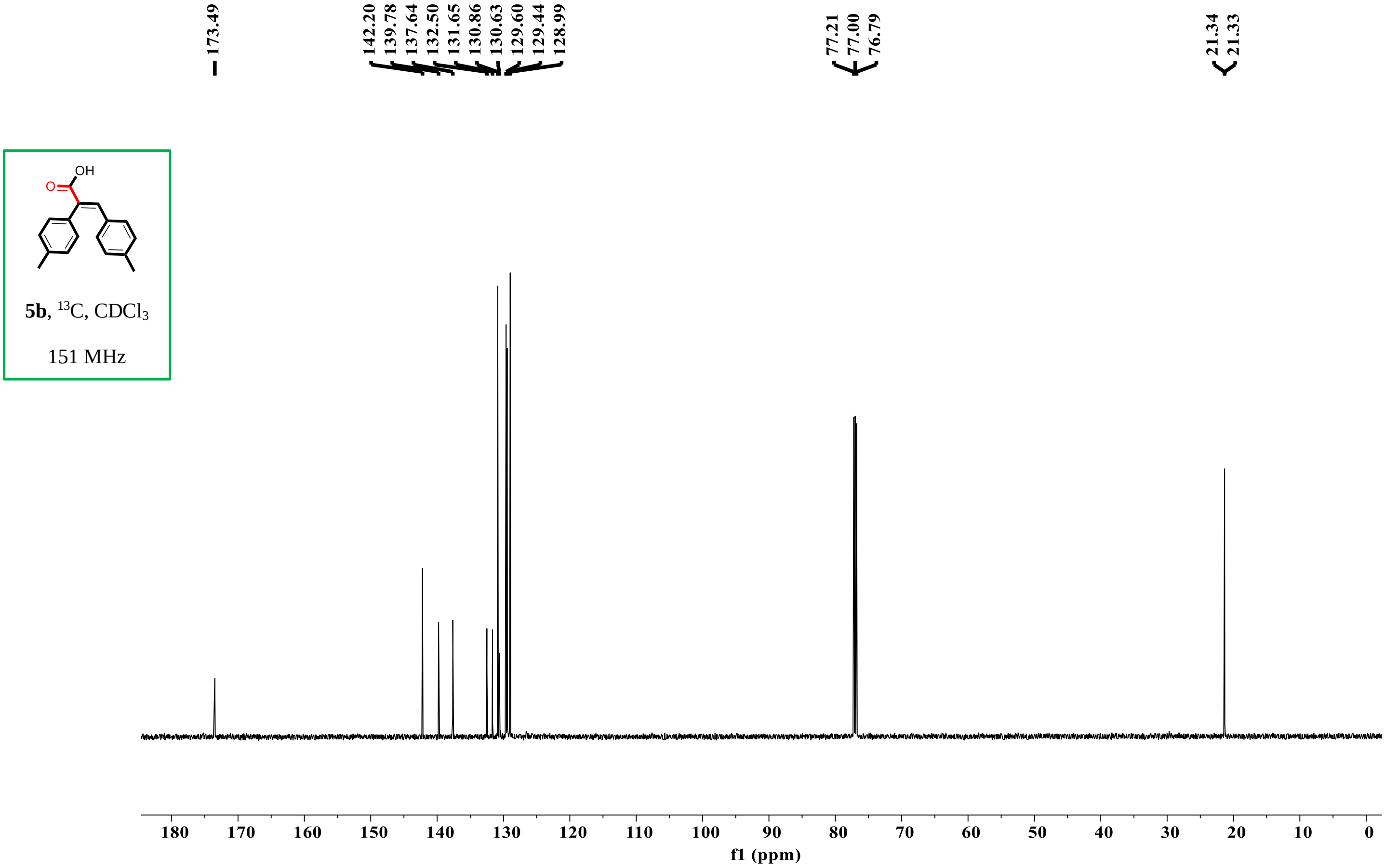
173.49
142.20
139.78
137.64
132.50
131.65
130.86
130.63
129.60
129.44
128.99
77.21
77.00
76.79
21.34
21.33
OH
O
5b, 13C, CDCl3
151 MHz
180 170 160 150 140 130 120 110 100 90 80 70 60 50 40 30 20 10 0
f1 (ppm)

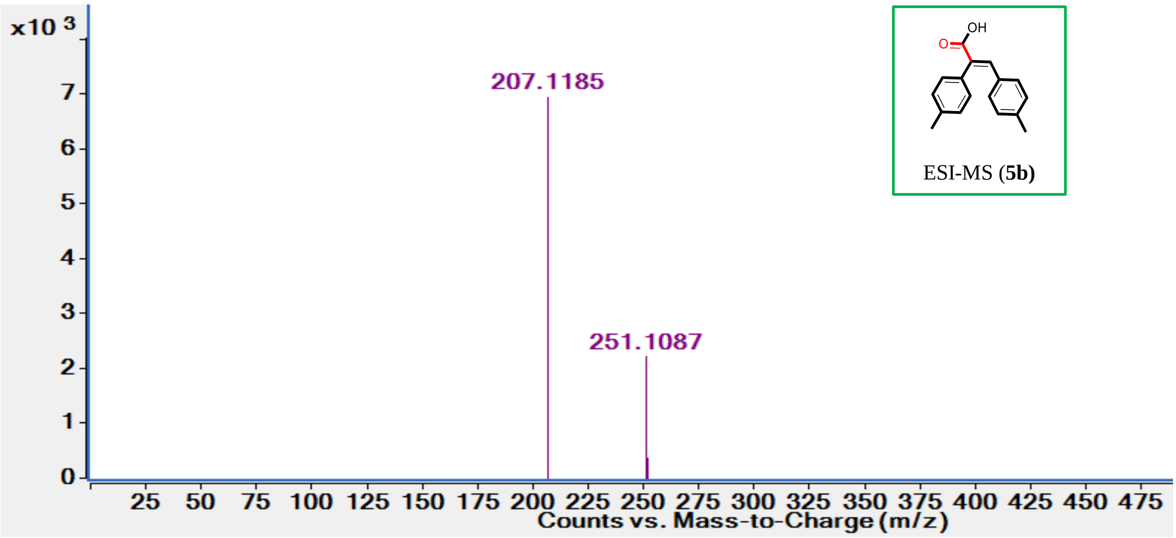
x10 3
207.1185
251.1087
Counts vs. Mass-to-Charge (m/z)
ESI-MS (5b)

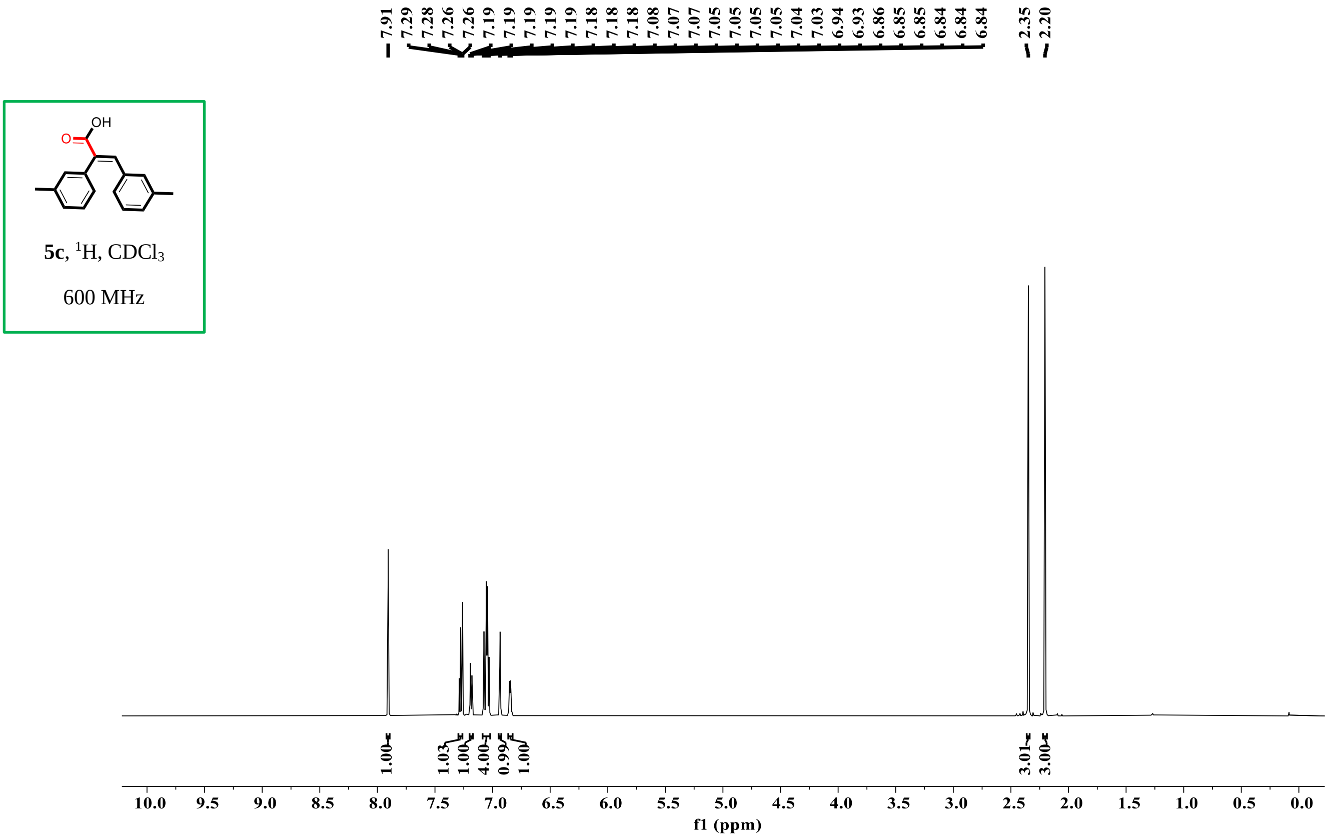
5c, 1H, CDCl3
600 MHz
f1 (ppm)

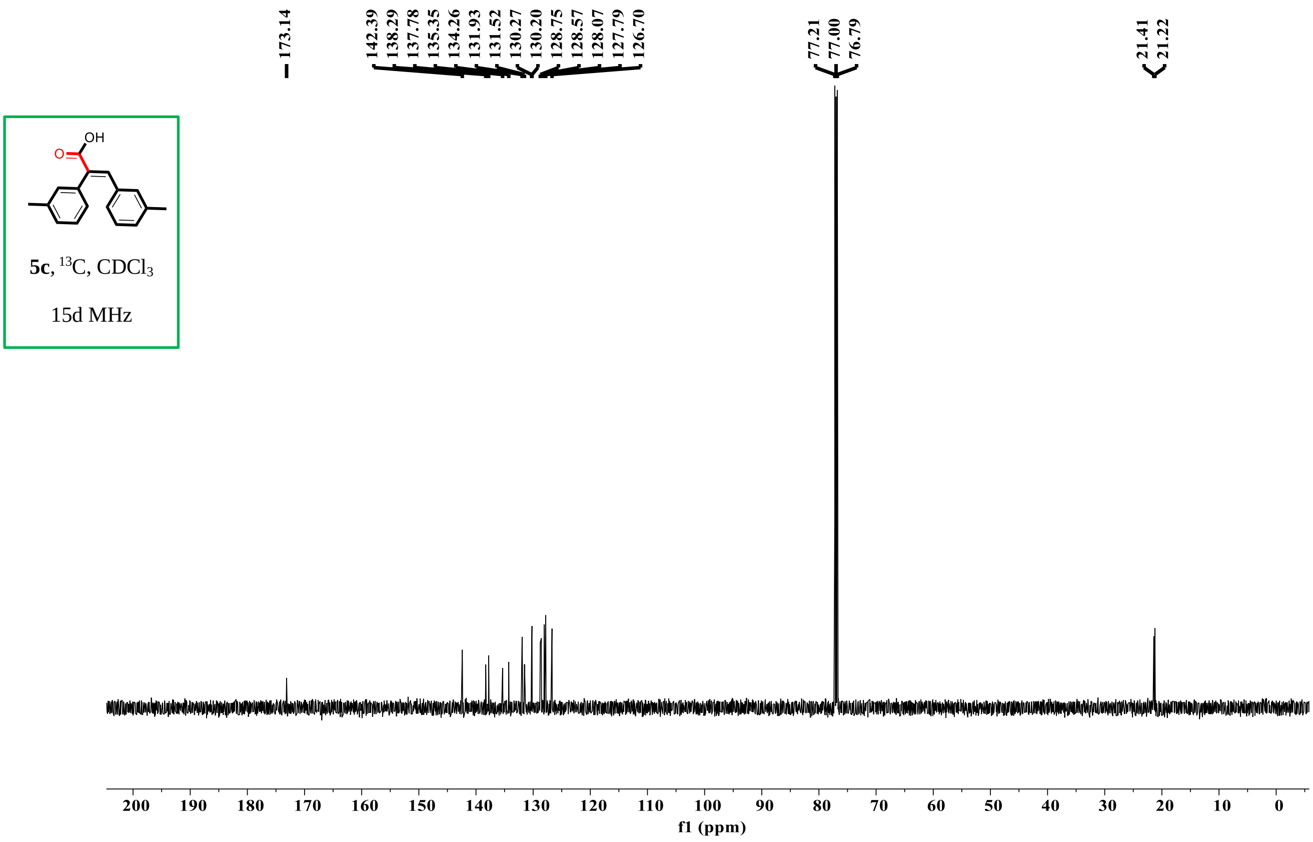
173.14
142.39
138.29
137.78
135.35
134.26
131.93
131.52
130.27
130.20
128.75
128.57
128.07
127.79
126.70
77.21
77.00
76.79
21.41
21.22
OH
O
5c, 13C, CDCl3
15d MHz
200 190 180 170 160 150 140 130 120 110 100 90 80 70 60 50 40 30 20 10 0
f1 (ppm)

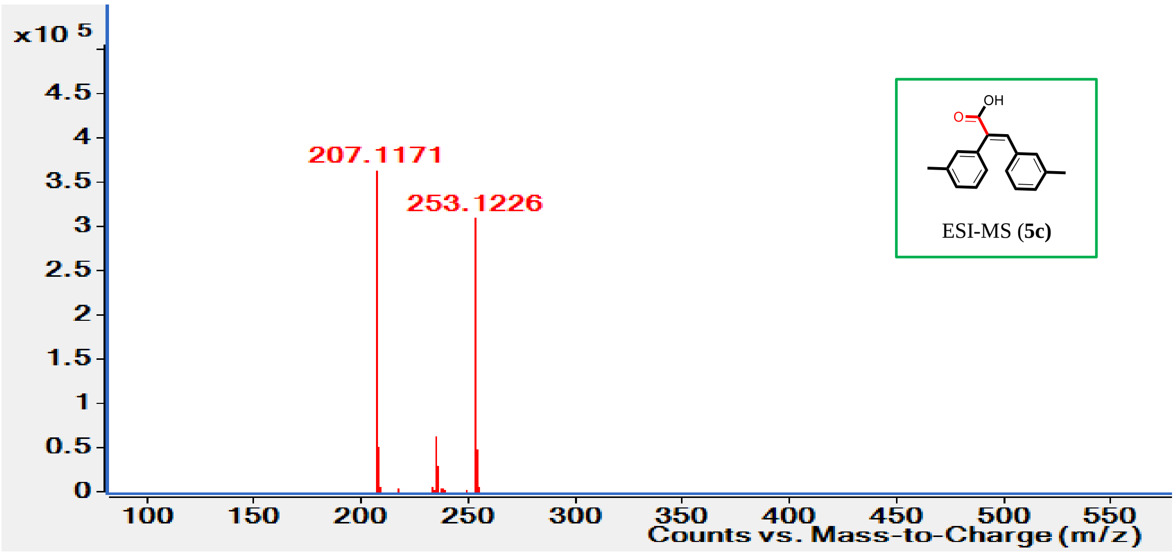
x10 5
4.5
4
3.5
3
2.5
2
1.5
1
0.5
0
207.1171
253.1226
OH
O
ESI-MS (5c)
100 150 200 250 300 350 400 450 500 550
Counts vs. Mass-to-Charge (m/z)

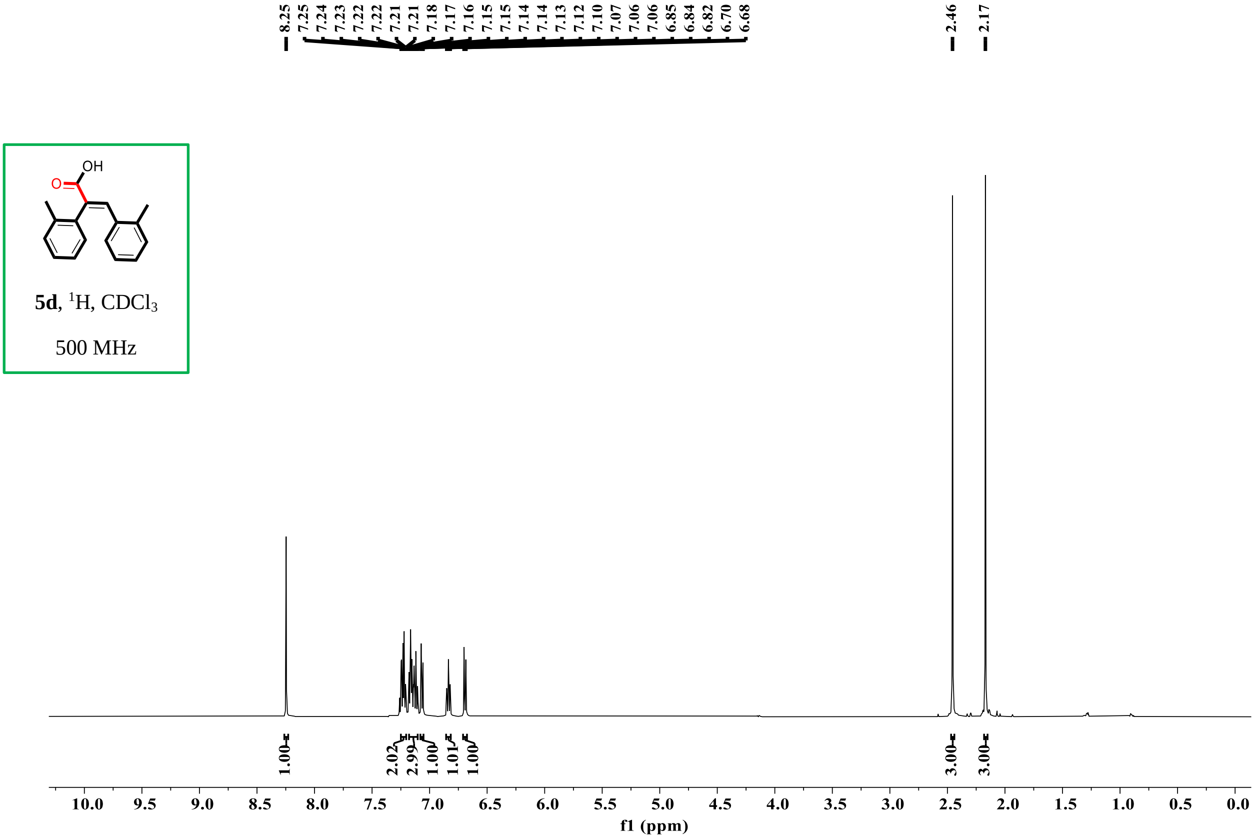
8.25 7.25 7.24 7.23 7.22 7.22 7.21 7.21 7.18 7.17 7.16 7.15 7.15 7.14 7.14 7.13 7.12 7.10 7.07 7.06 7.06 6.85 6.84 6.82 6.70 6.68
2.46 2.17
OH
O
5d, 1H, CDCl3
500 MHz
1.00
2.02 2.99 1.00 1.01 1.00
3.00 3.00
10.0 9.5 9.0 8.5 8.0 7.5 7.0 6.5 6.0 5.5 5.0 4.5 4.0 3.5 3.0 2.5 2.0 1.5 1.0 0.5 0.0
f1 (ppm)

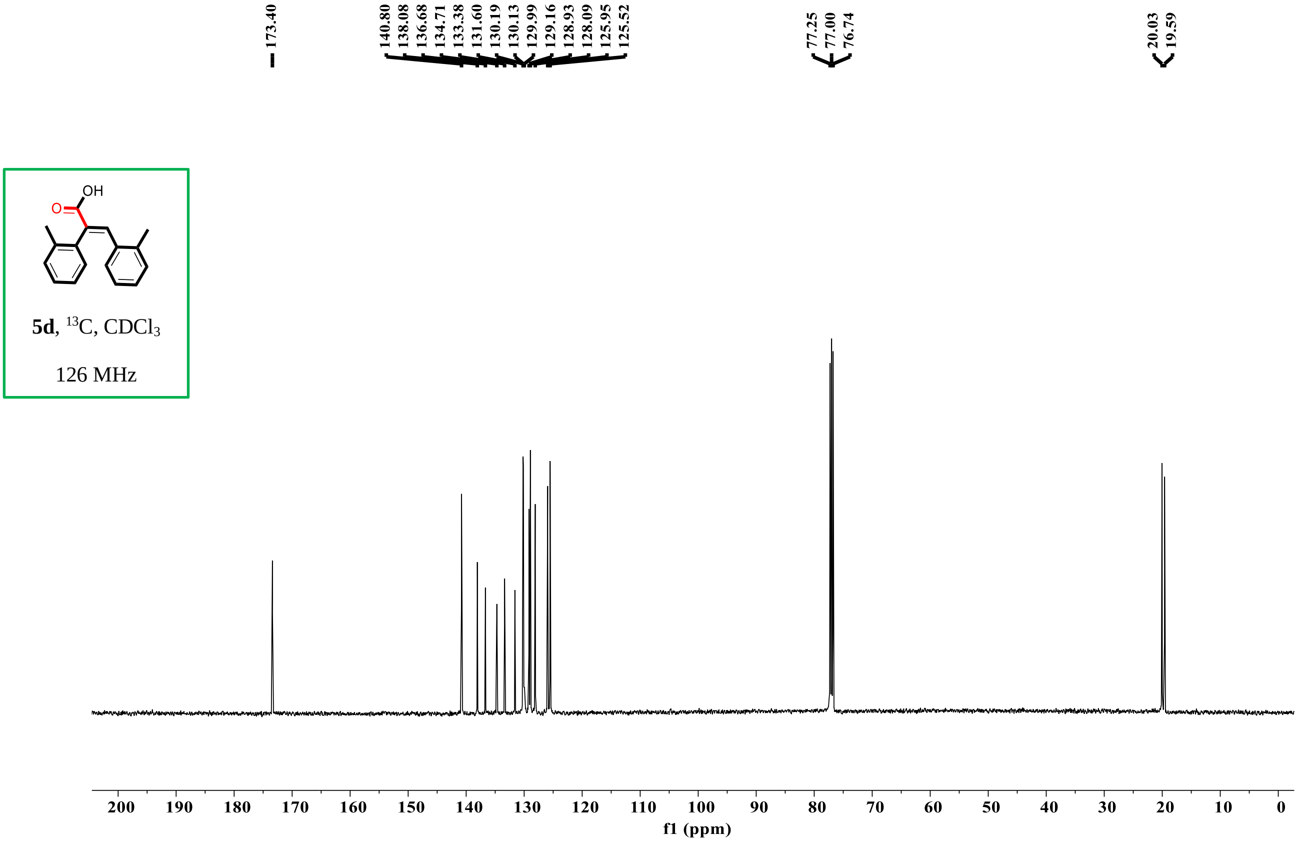
173.40
140.80 138.08 136.68 134.71 133.38 131.60 130.19 130.13 129.99 129.16 128.93 128.09 125.95 125.52
77.25 77.00 76.74
20.03 19.59
OH
O
5d, 13C, CDCl3
126 MHz
200 190 180 170 160 150 140 130 120 110 100 90 80 70 60 50 40 30 20 10 0
f1 (ppm)

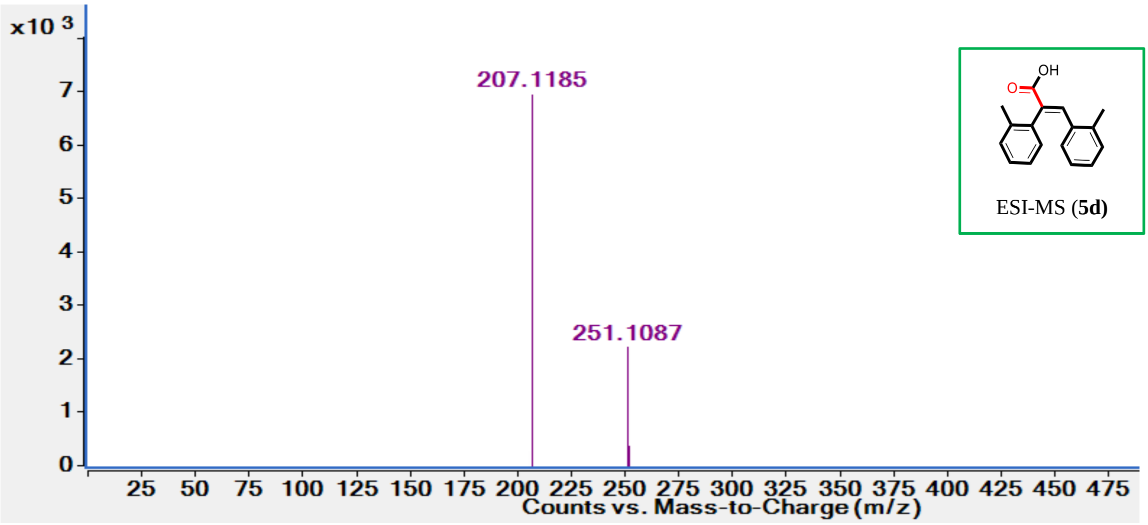
x10 3
207.1185
251.1087
Counts vs. Mass-to-Charge (m/z)
OH
O
ESI-MS (5d)

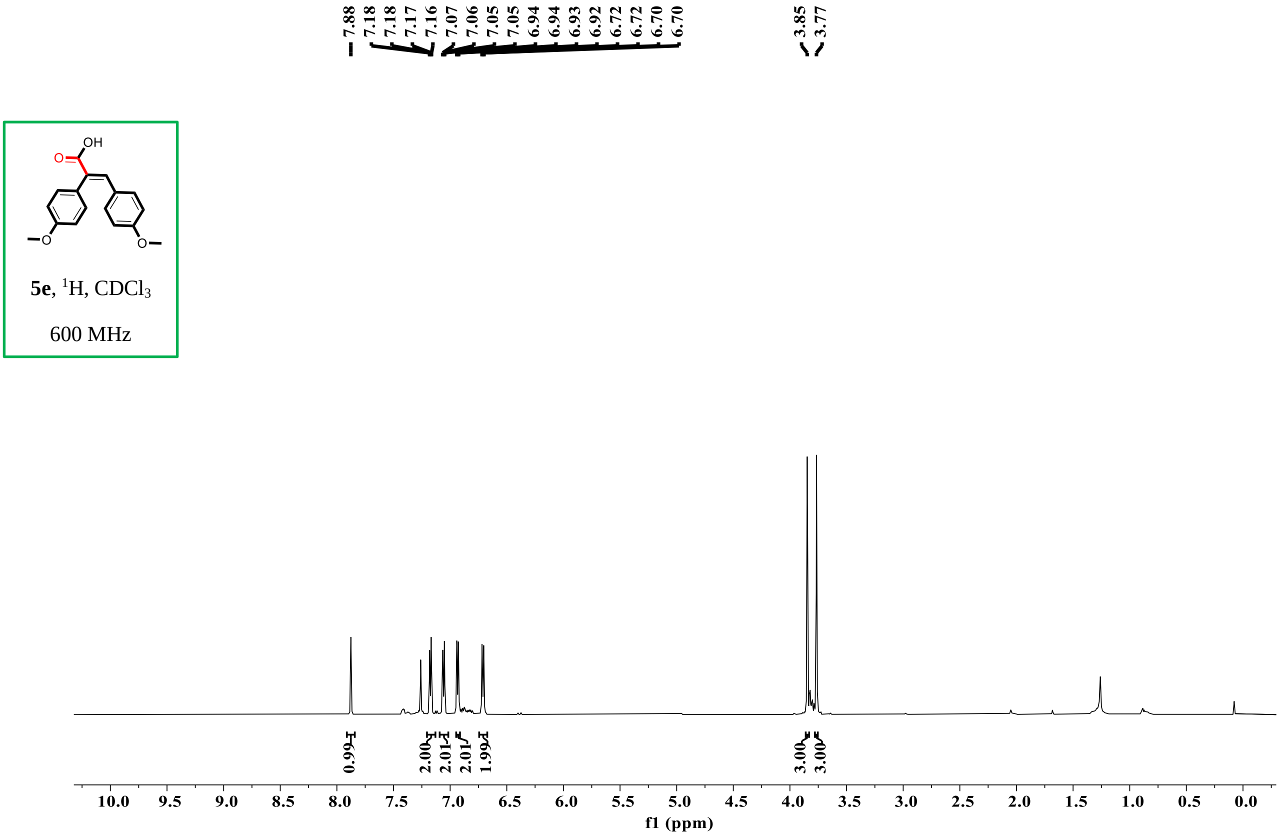
7.88
7.18
7.18
7.17
7.16
7.07
7.06
7.05
7.05
6.94
6.94
6.93
6.92
6.72
6.72
6.70
6.70
3.85
3.77
OH
O
O
O
5e, ¹H, CDCl₃
600 MHz
0.99
2.00
2.01
2.01
1.99
3.00
3.00
f1 (ppm)

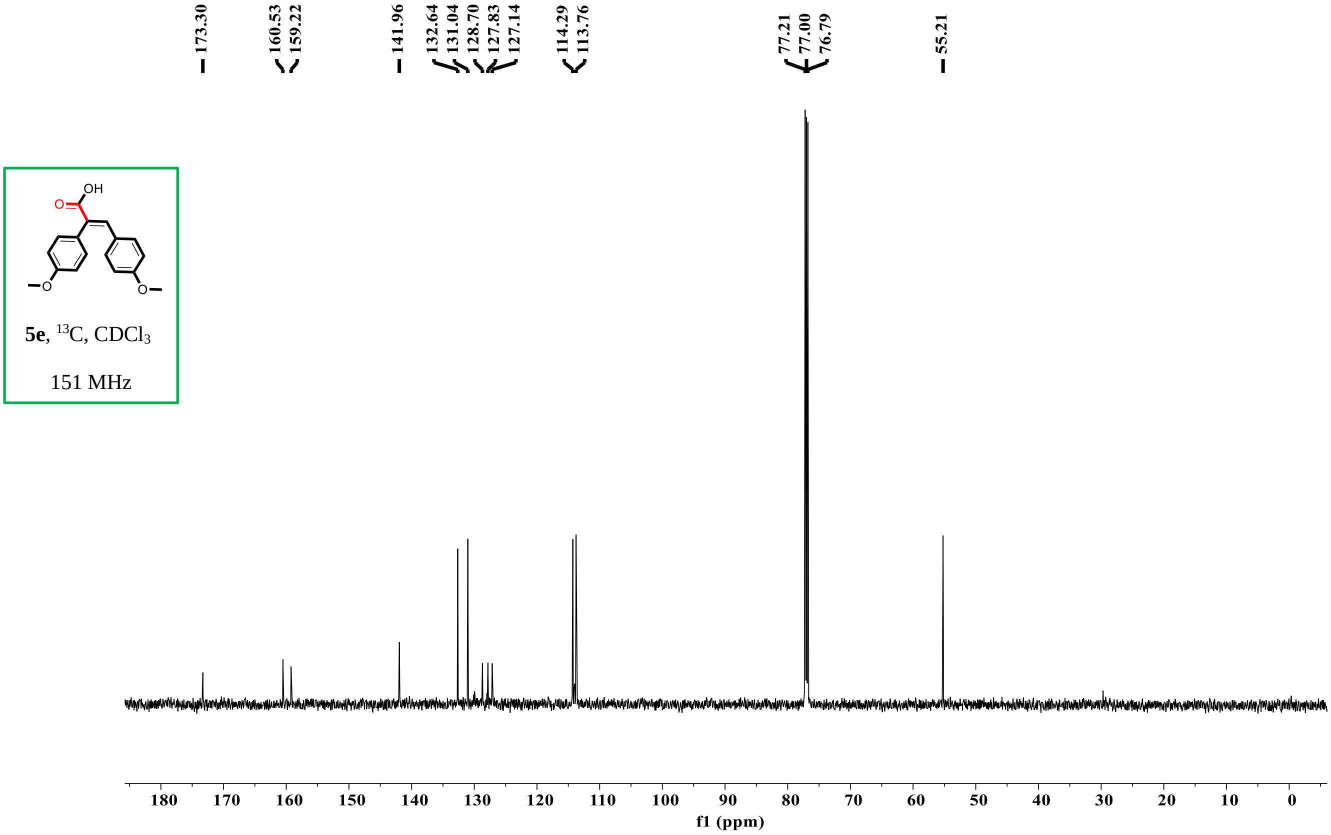
173.30
160.53
159.22
141.96
132.64
131.04
128.70
127.83
127.14
114.29
113.76
77.21
77.00
76.79
55.21
5e, 13C, CDCl3
151 MHz
180 170 160 150 140 130 120 110 100 90 80 70 60 50 40 30 20 10 0
f1 (ppm)

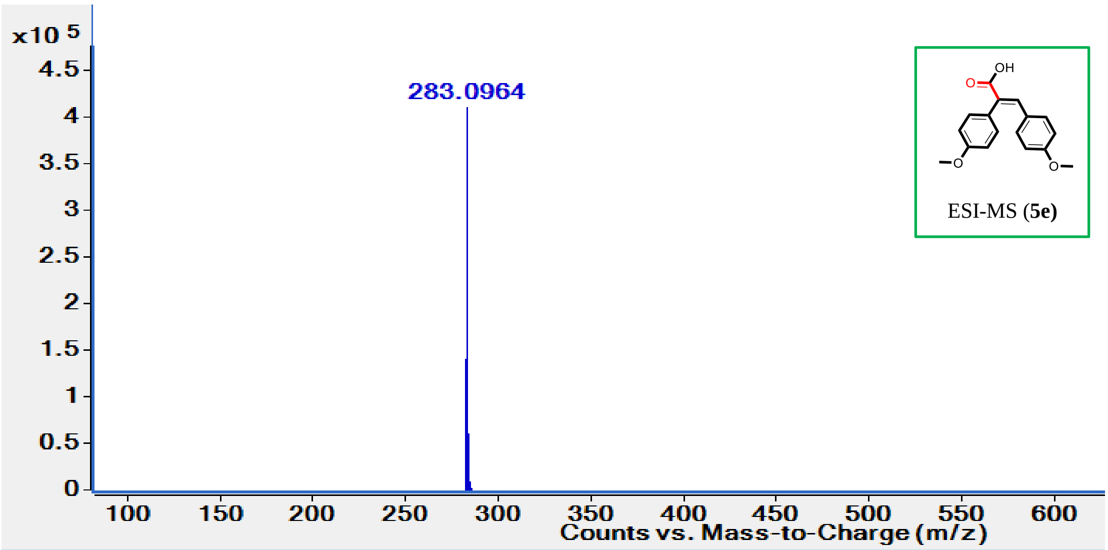
x10 5
4.5
4
3.5
3
2.5
2
1.5
1
0.5
0
283.0964
ESI-MS (5e)
100 150 200 250 300 350 400 450 500 550 600
Counts vs. Mass-to-Charge (m/z)

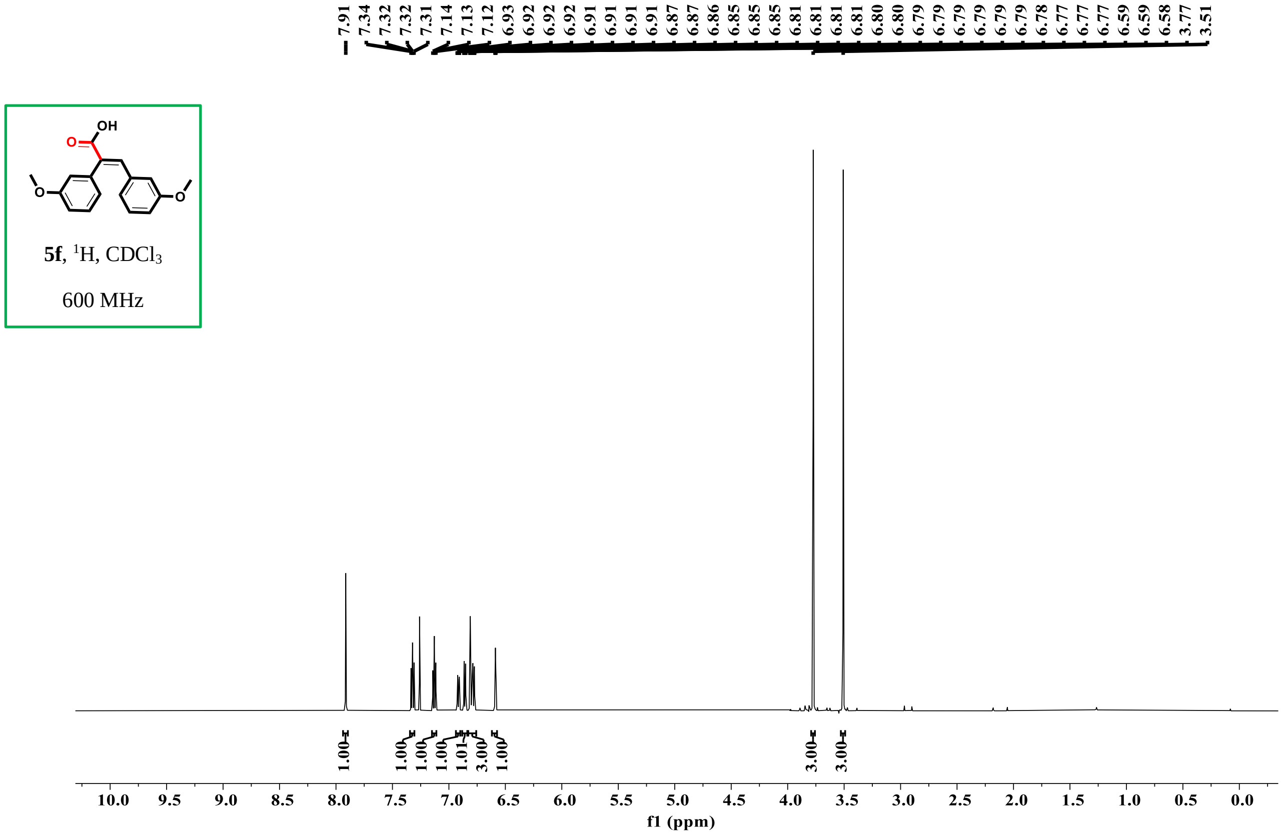
7.91 7.34 7.32 7.32 7.31 7.14 7.13 7.12 6.93 6.92 6.92 6.92 6.91 6.91 6.91 6.91 6.87 6.87 6.86 6.85 6.85 6.85 6.81 6.81 6.81 6.81 6.80 6.80 6.79 6.79 6.79 6.79 6.79 6.79 6.78 6.77 6.77 6.77 6.59 6.59 6.58 3.77 3.51
OH
O
5f, 1H, CDCl3
600 MHz
1.00 1.00 1.00 1.00 1.01 3.00 1.00 3.00 3.00
10.0 9.5 9.0 8.5 8.0 7.5 7.0 6.5 6.0 5.5 5.0 4.5 4.0 3.5 3.0 2.5 2.0 1.5 1.0 0.5 0.0
f1 (ppm)

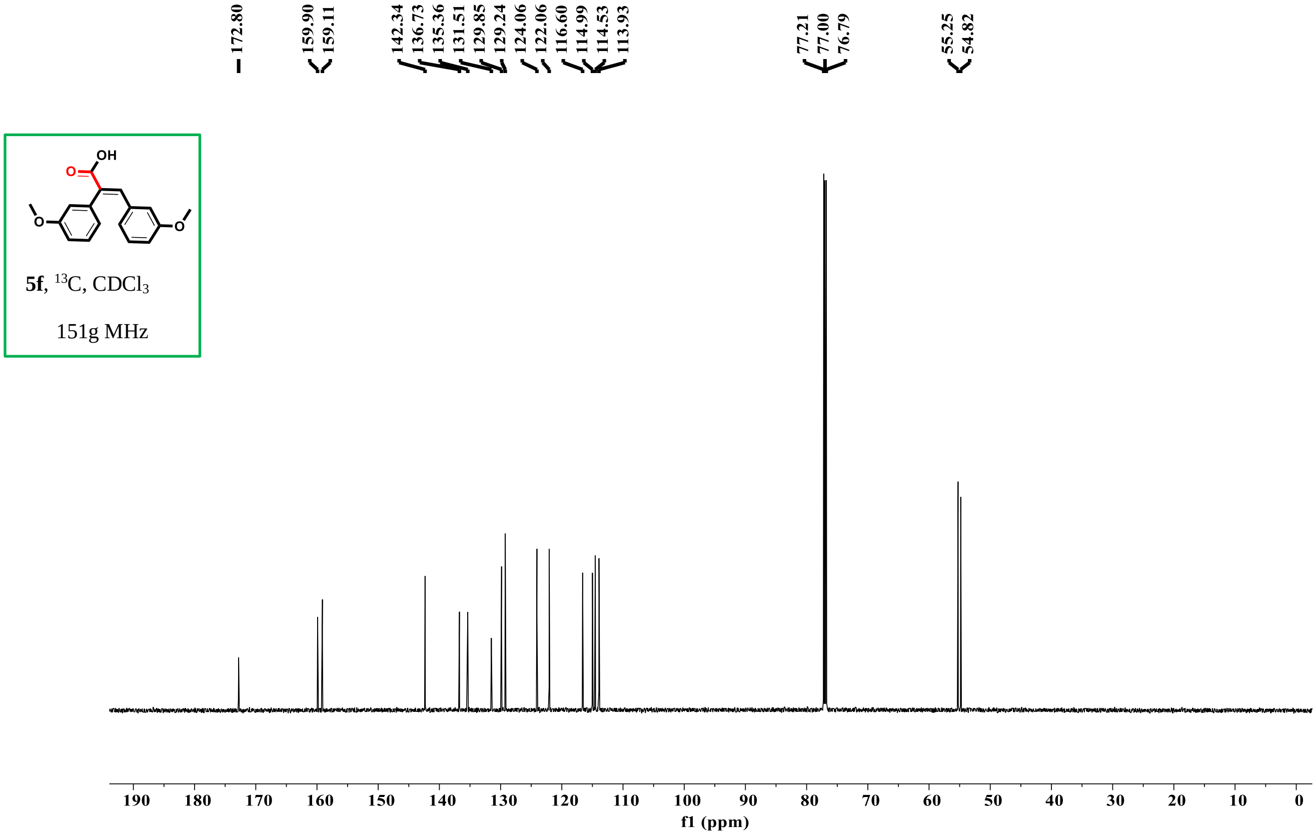
172.80 159.90 159.11 142.34 136.73 135.36 131.51 129.85 129.24 124.06 122.06 116.60 114.99 114.53 113.93 77.21 77.00 76.79 55.25 54.82
OH
O
5f, 13C, CDCl3
151g MHz
190 180 170 160 150 140 130 120 110 100 90 80 70 60 50 40 30 20 10 0
f1 (ppm)

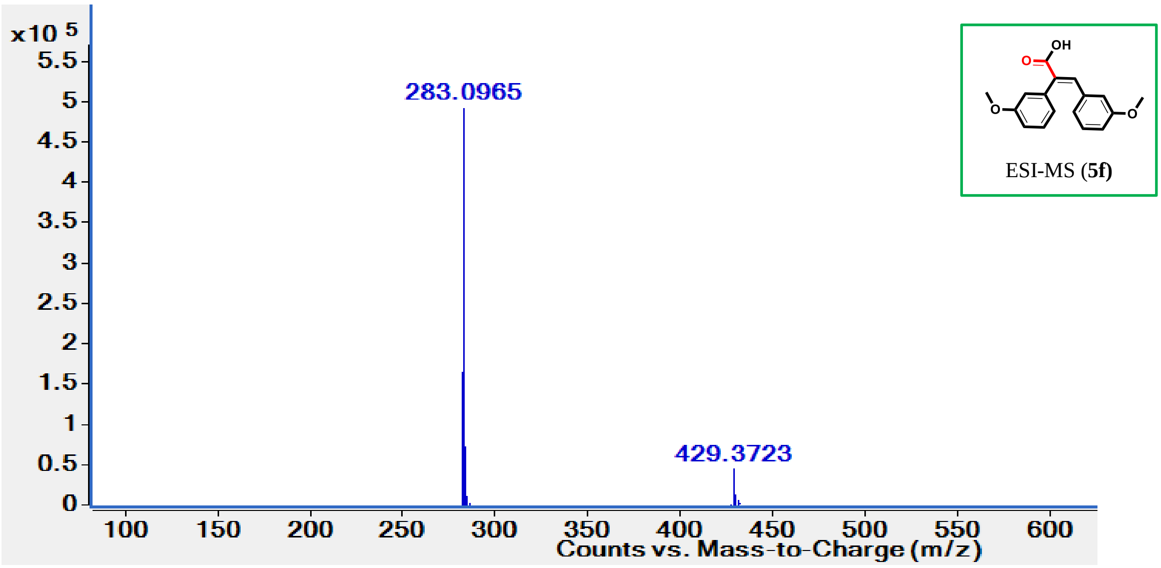
x10 5
283.0965
429.3723
Counts vs. Mass-to-Charge (m/z)
ESI-MS (5f)

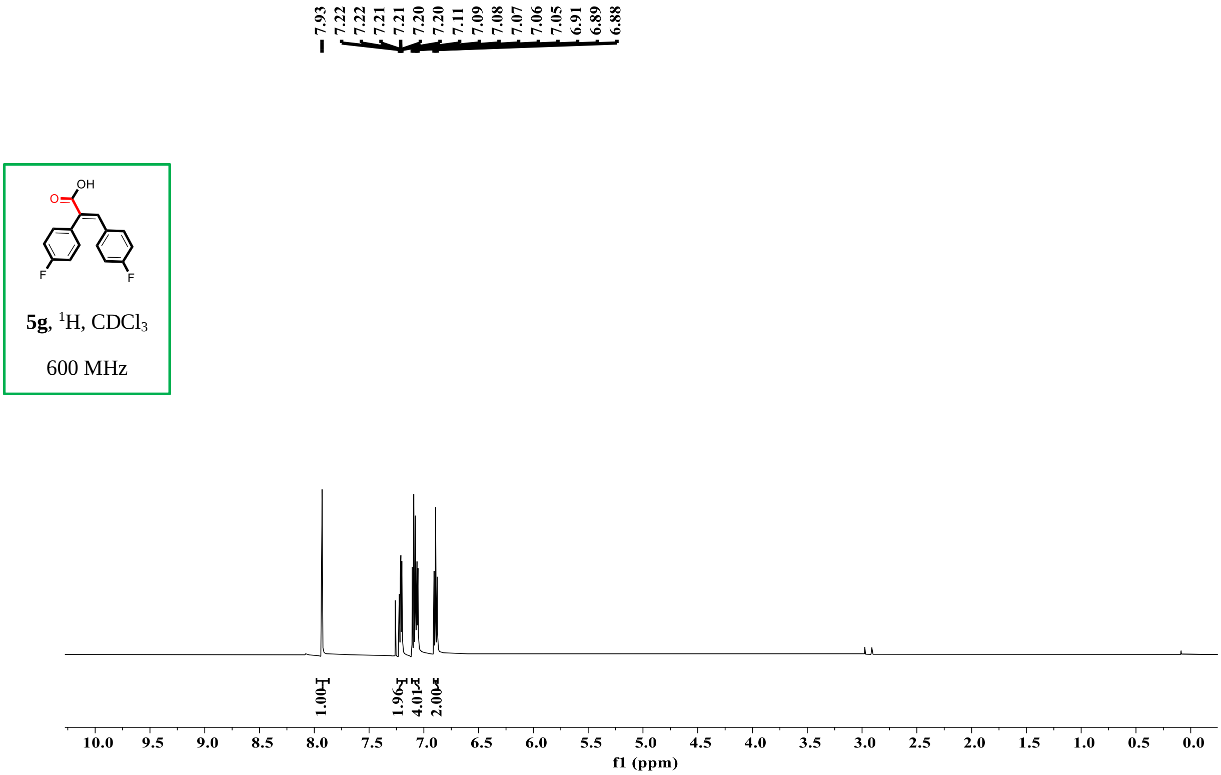
5g, 1H, CDCl3
600 MHz
f1 (ppm)

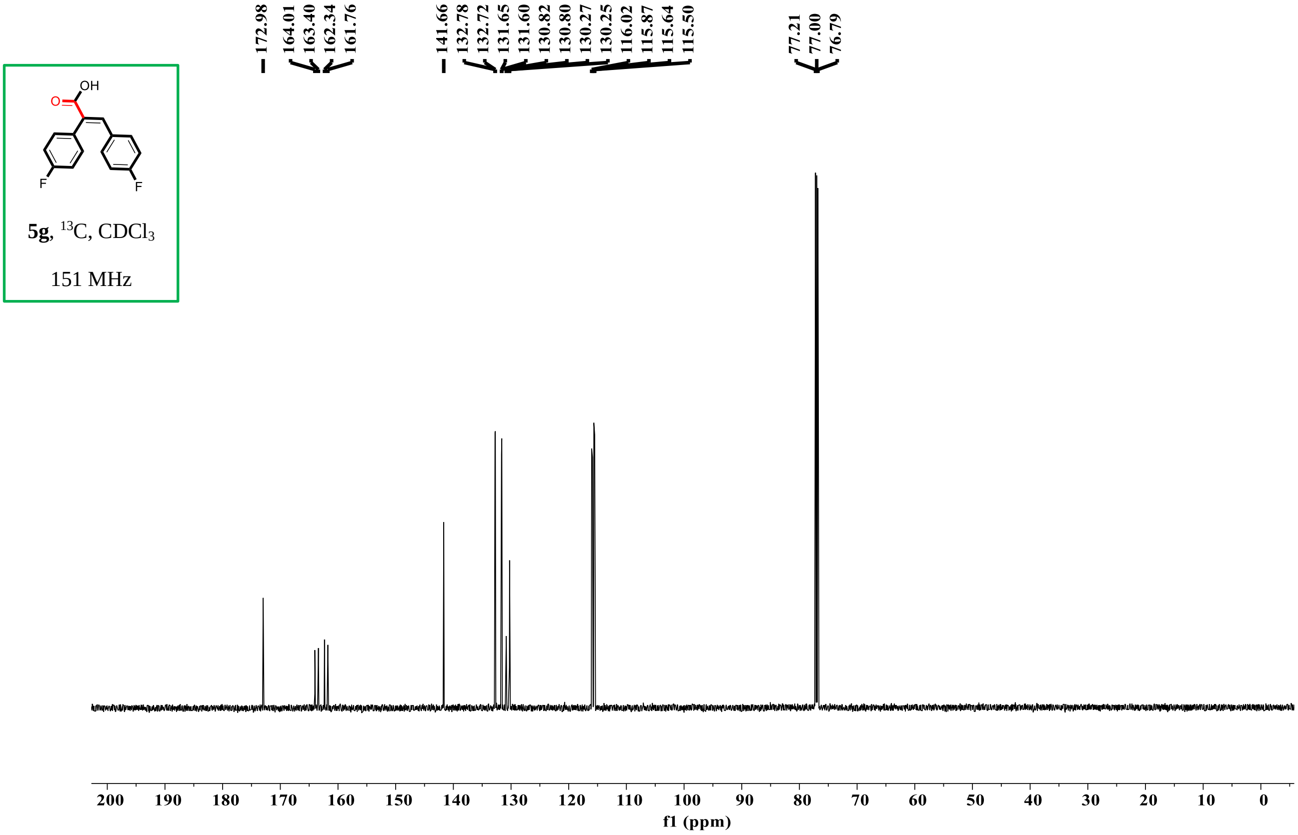

172.98
164.01
163.40
162.34
161.76
141.66
132.78
132.72
131.65
131.60
130.82
130.80
130.27
130.25
116.02
115.87
115.64
115.50
77.21
77.00
76.79
OH
O
F
F
5g, 13C, CDCl3
151 MHz
200 190 180 170 160 150 140 130 120 110 100 90 80 70 60 50 40 30 20 10 0
f1 (ppm)

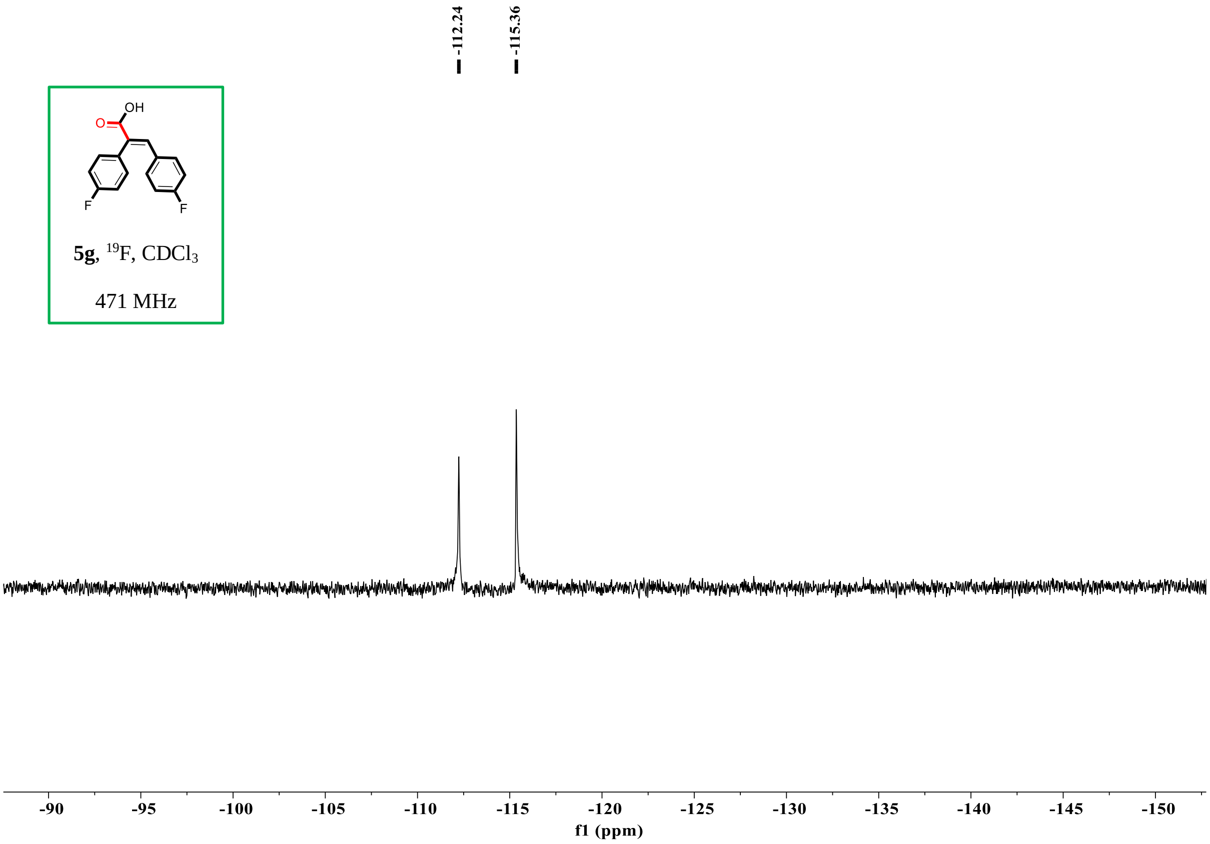

-112.24
-115.36
OH
O
F
F
5g, 19F, CDCl3
471 MHz
-90 -95 -100 -105 -110 -115 -120 -125 -130 -135 -140 -145 -150
f1 (ppm)

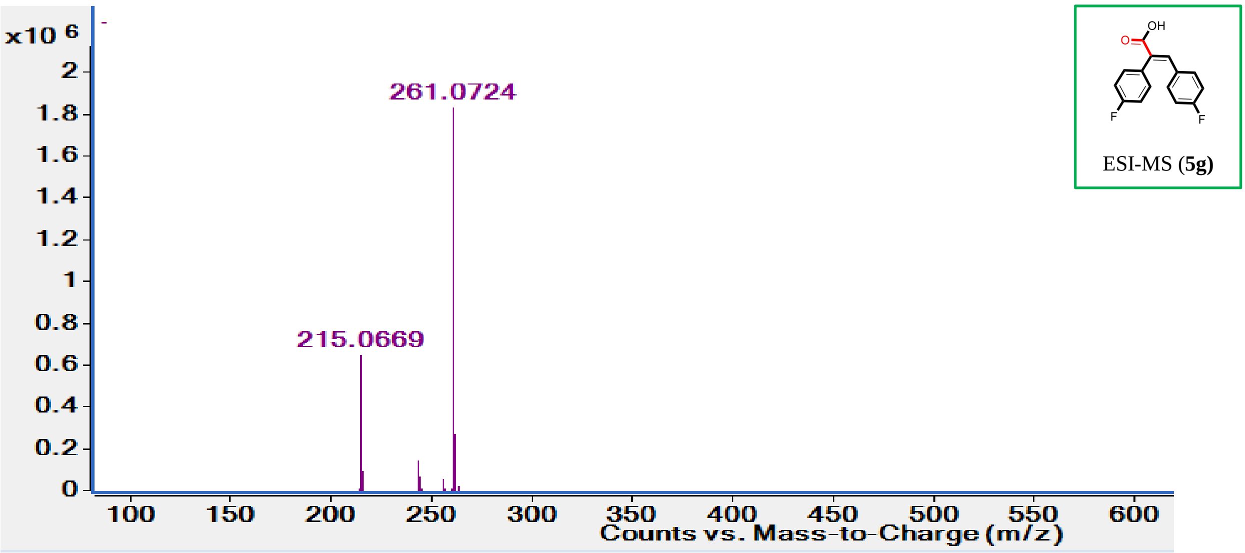

ESI-MS (**5g**)

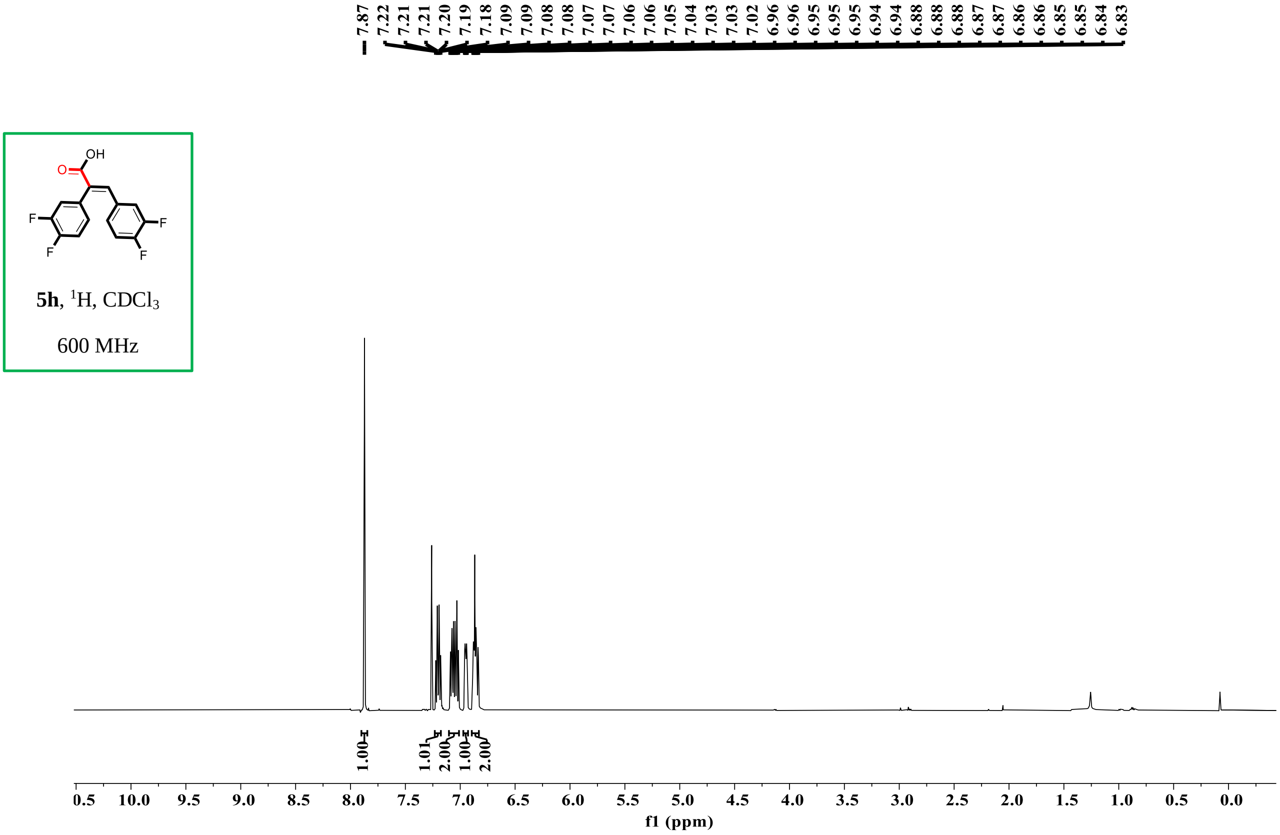

5h, ^{1}H, $CDCl_3$

600 MHz

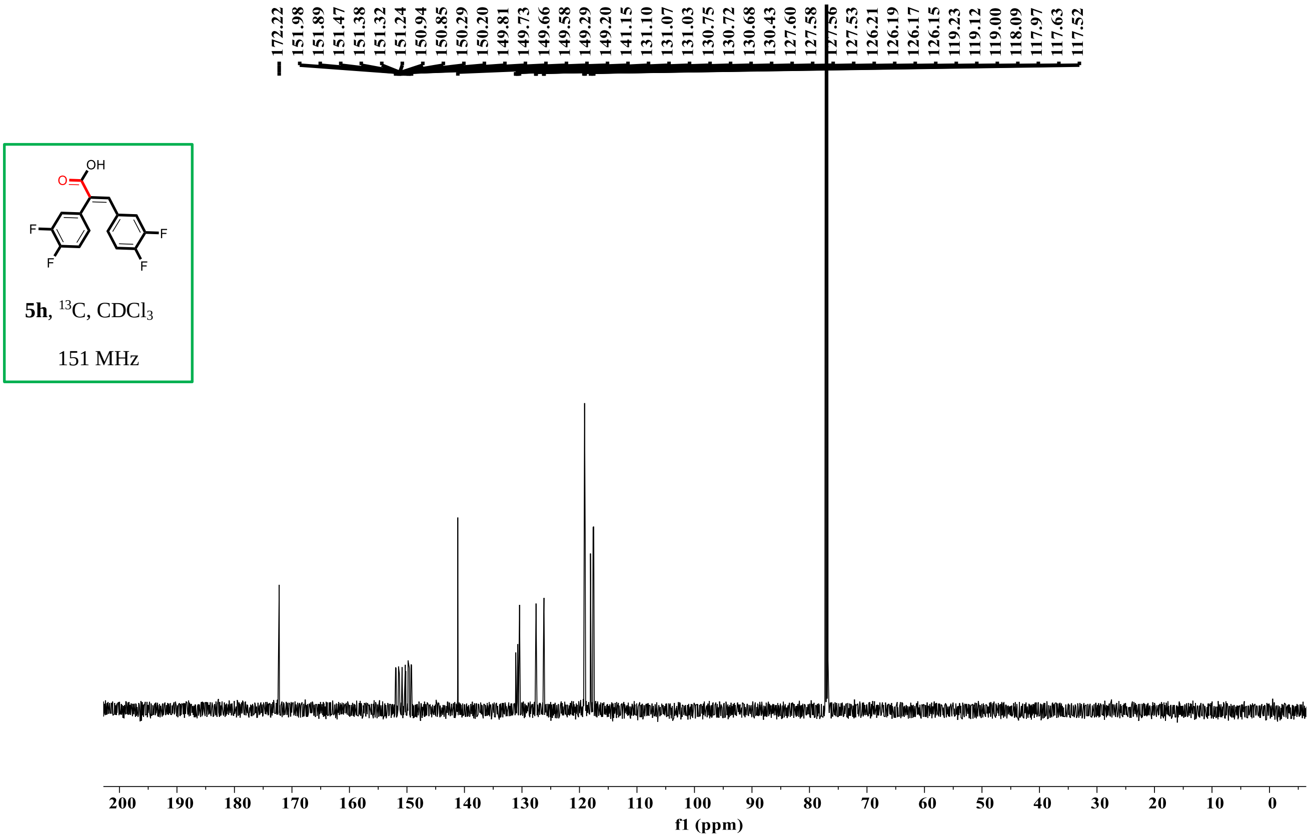
172.22
151.98
151.89
151.47
151.38
151.32
151.24
150.94
150.85
150.29
150.20
149.81
149.73
149.66
149.58
149.29
149.20
141.15
131.10
131.07
131.03
130.75
130.72
130.68
130.43
127.60
127.58
127.56
127.53
126.21
126.19
126.17
126.15
119.23
119.12
119.00
118.09
117.97
117.63
117.52
OH
O
F
F
F
F
5h, ^{13}C, $CDCl_3$
151 MHz
200 190 180 170 160 150 140 130 120 110 100 90 80 70 60 50 40 30 20 10 0
f1 (ppm)

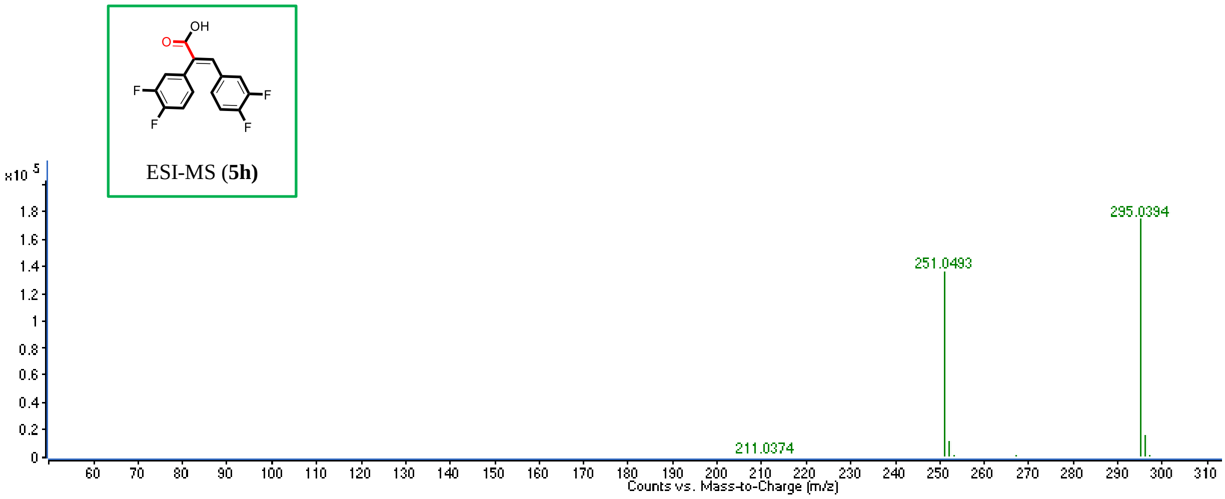
OH
O
F
F
F
F
ESI-MS (5h)
x10 5
1.8
1.6
1.4
1.2
1
0.8
0.6
0.4
0.2
0
295.0394
251.0493
211.0374
60 70 80 90 100 110 120 130 140 150 160 170 180 190 200 210 220 230 240 250 260 270 280 290 300 310
Counts vs. Mass-to-Charge (m/z)

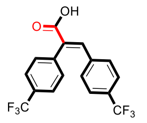

5i, ^{1}H, $CDCl_3$

600 MHz

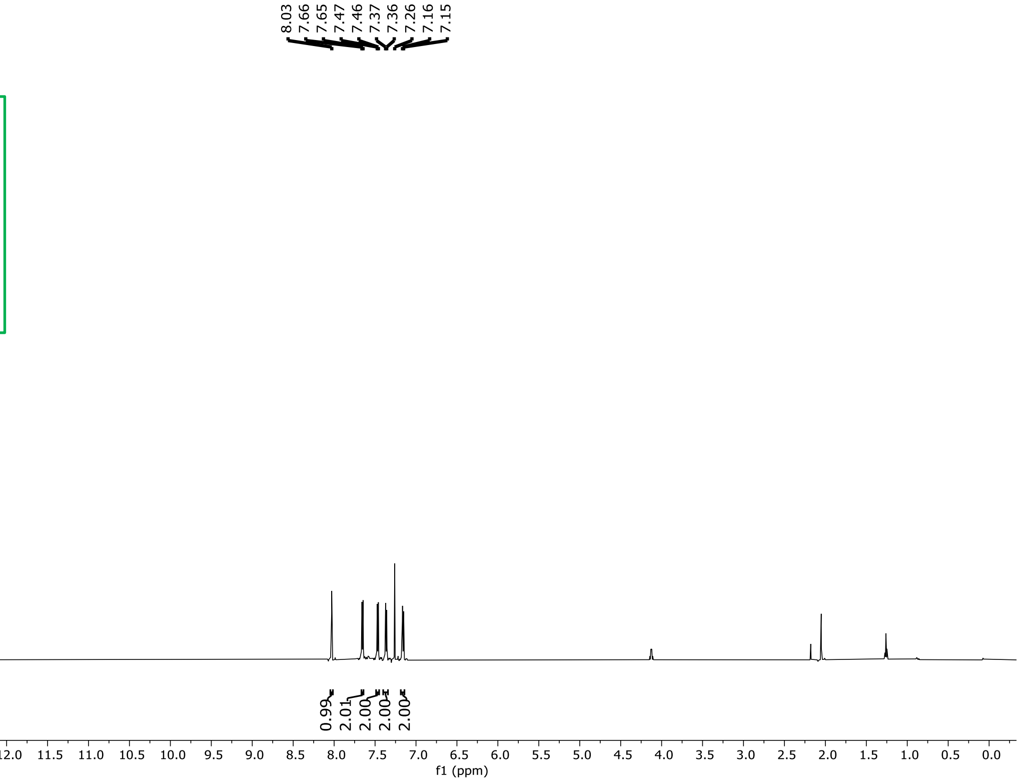

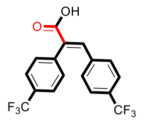

5i, ^{13}C, $CDCl_3$

151 MHz

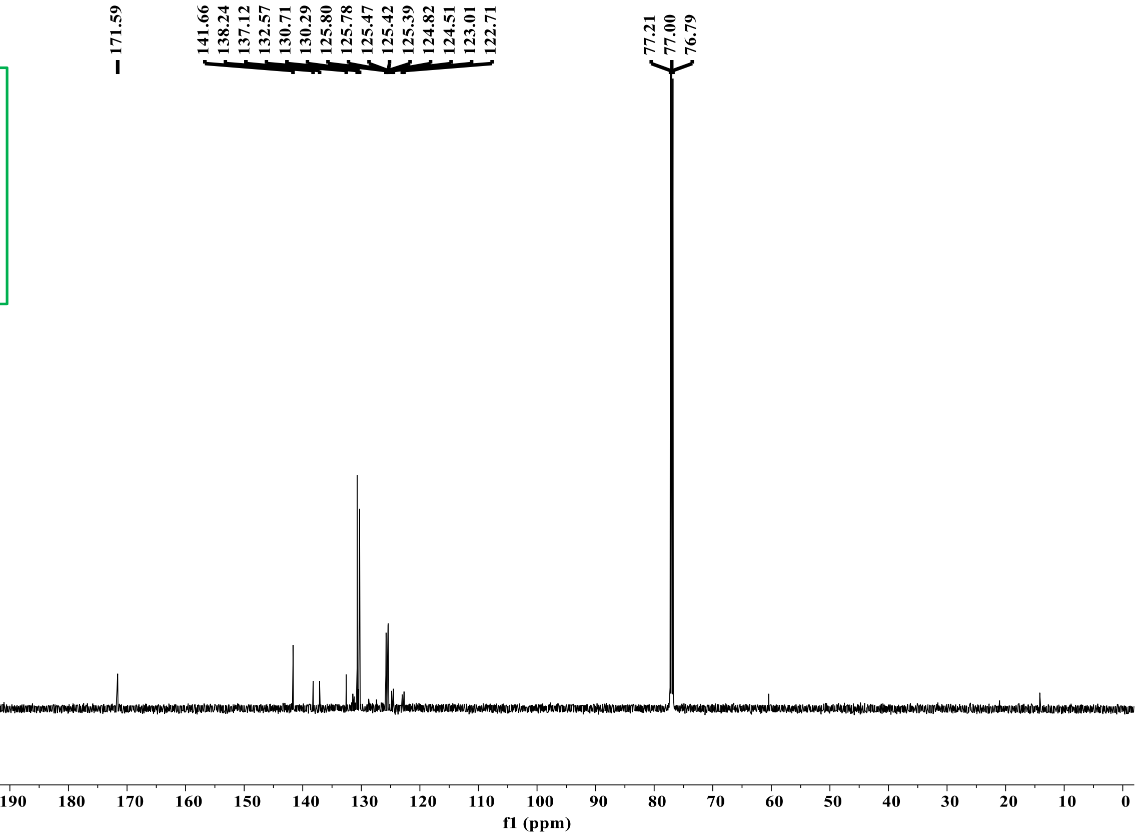

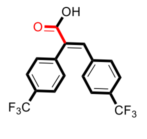

ESI-MS (**5i**)

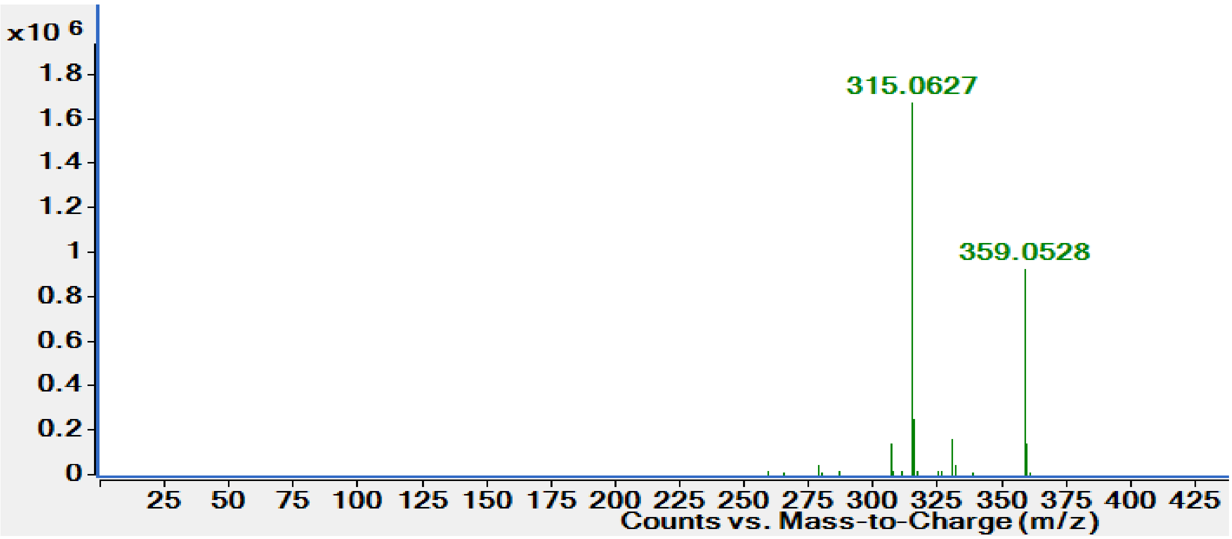
x10 6
1.8
1.6
1.4
1.2
1
0.8
0.6
0.4
0.2
0
315.0627
359.0528
25 50 75 100 125 150 175 200 225 250 275 300 325 350 375 400 425
Counts vs. Mass-to-Charge (m/z)

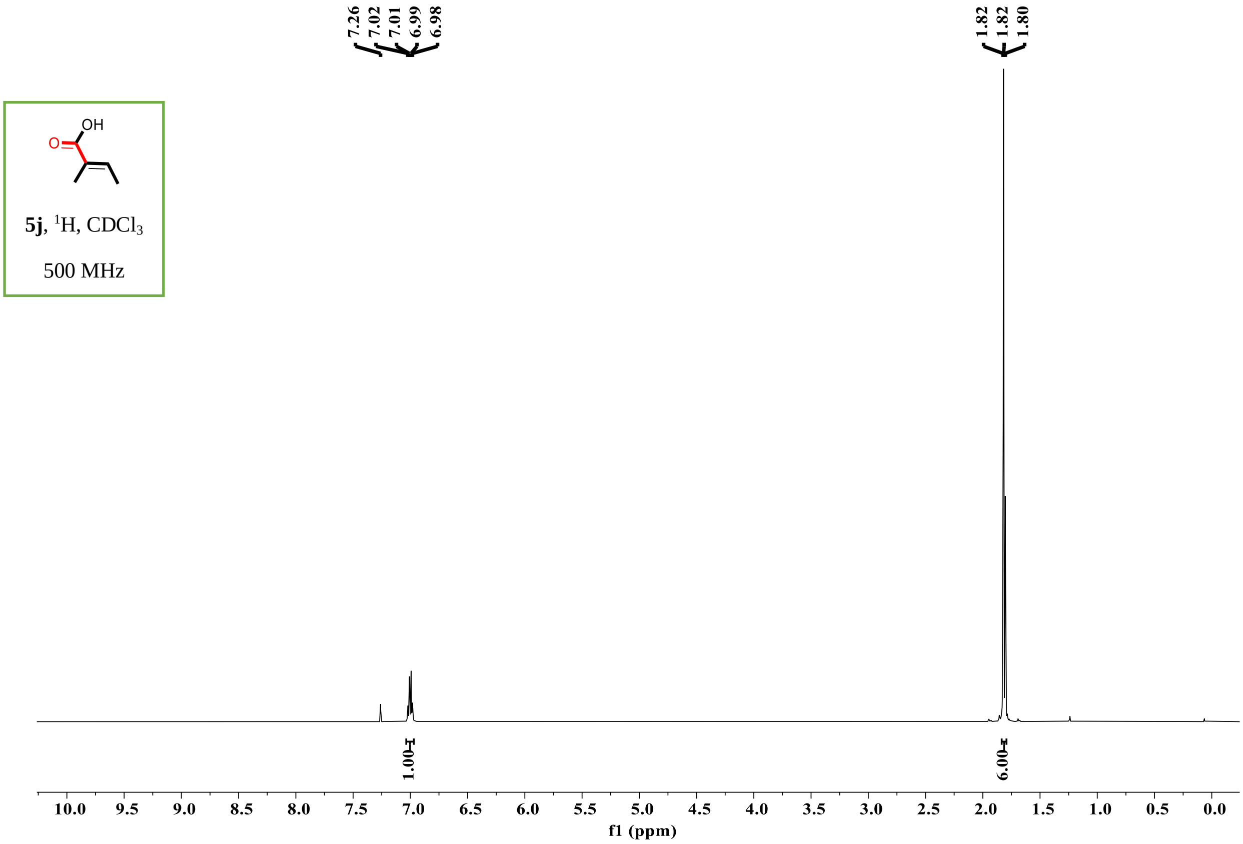
OH
O
5j, 1H, CDCl3
500 MHz
7.26
7.02
7.01
6.99
6.98
1.82
1.82
1.80
1.00
6.00
10.0 9.5 9.0 8.5 8.0 7.5 7.0 6.5 6.0 5.5 5.0 4.5 4.0 3.5 3.0 2.5 2.0 1.5 1.0 0.5 0.0
f1 (ppm)

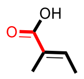

5j, ^{13}C, $CDCl_3$

126 MHz

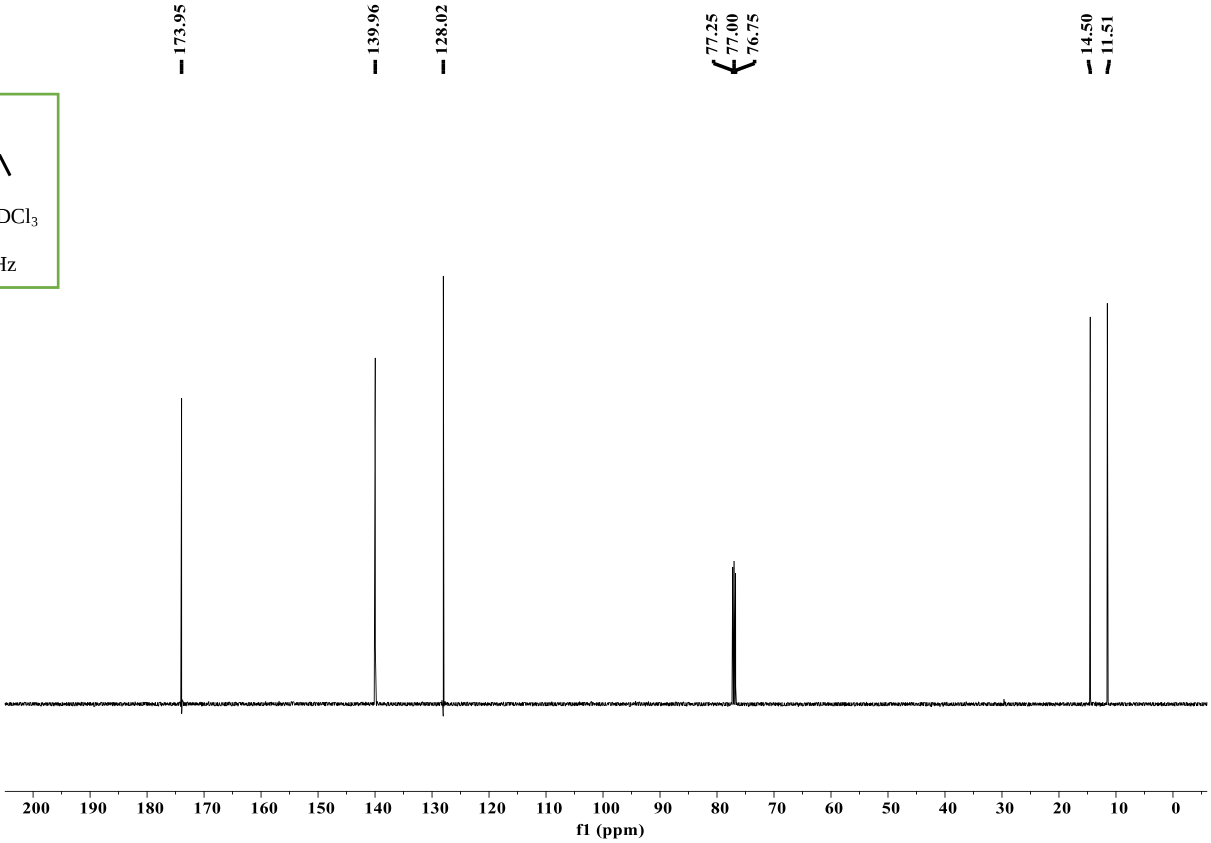

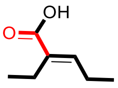

5k, ^{1}H, $CDCl_3$

600 MHz

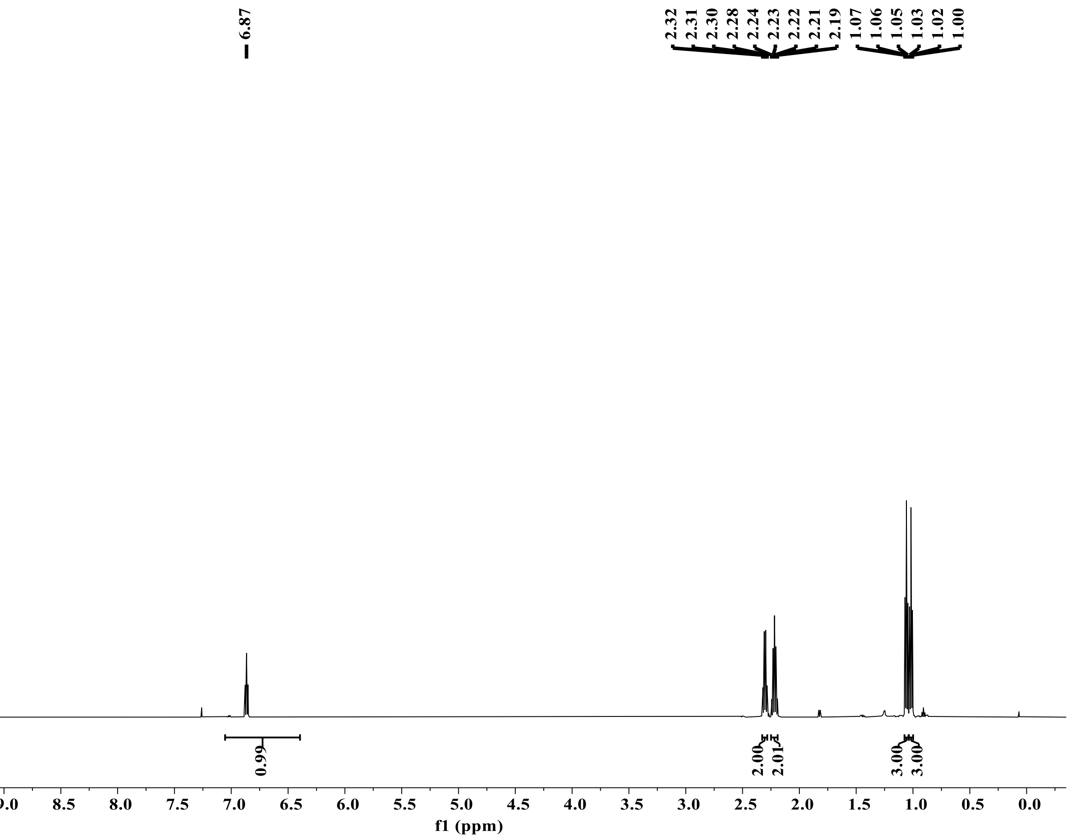

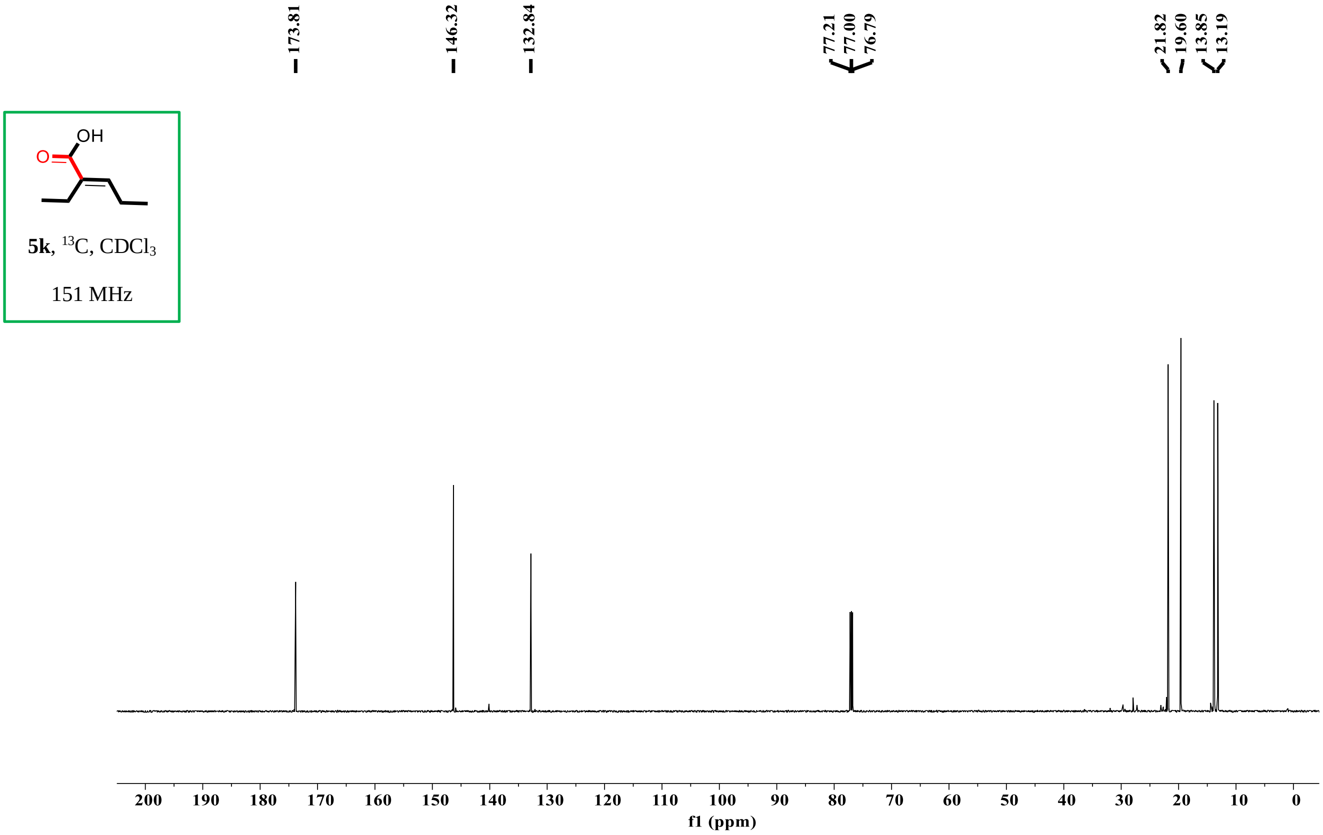
173.81
146.32
132.84
77.21
77.00
76.79
21.82
19.60
13.85
13.19
OH
O
5k, 13C, CDCl3
151 MHz
200 190 180 170 160 150 140 130 120 110 100 90 80 70 60 50 40 30 20 10 0
f1 (ppm)

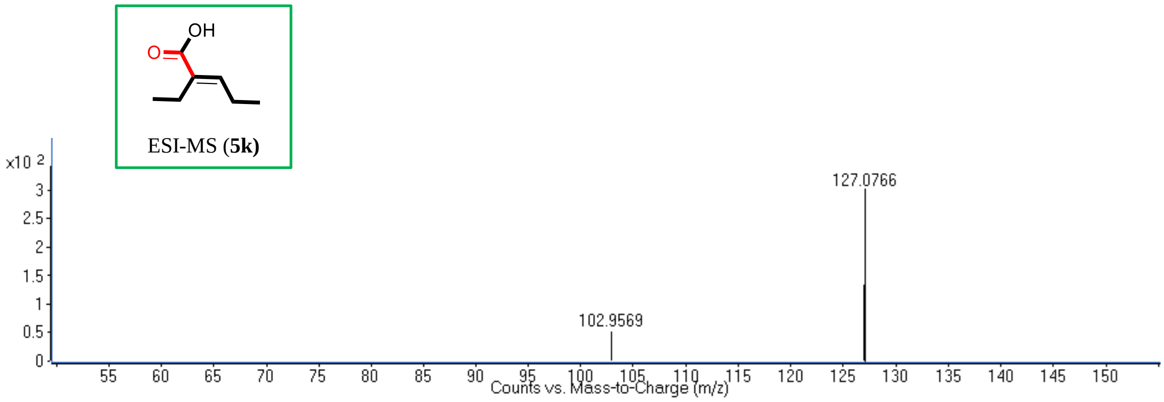
OH
O
ESI-MS (5k)
x10 2
3
2.5
2
1.5
1
0.5
0
127.0766
102.9569
55 60 65 70 75 80 85 90 95 100 105 110 115 120 125 130 135 140 145 150
Counts vs. Mass-to-Charge (m/z)

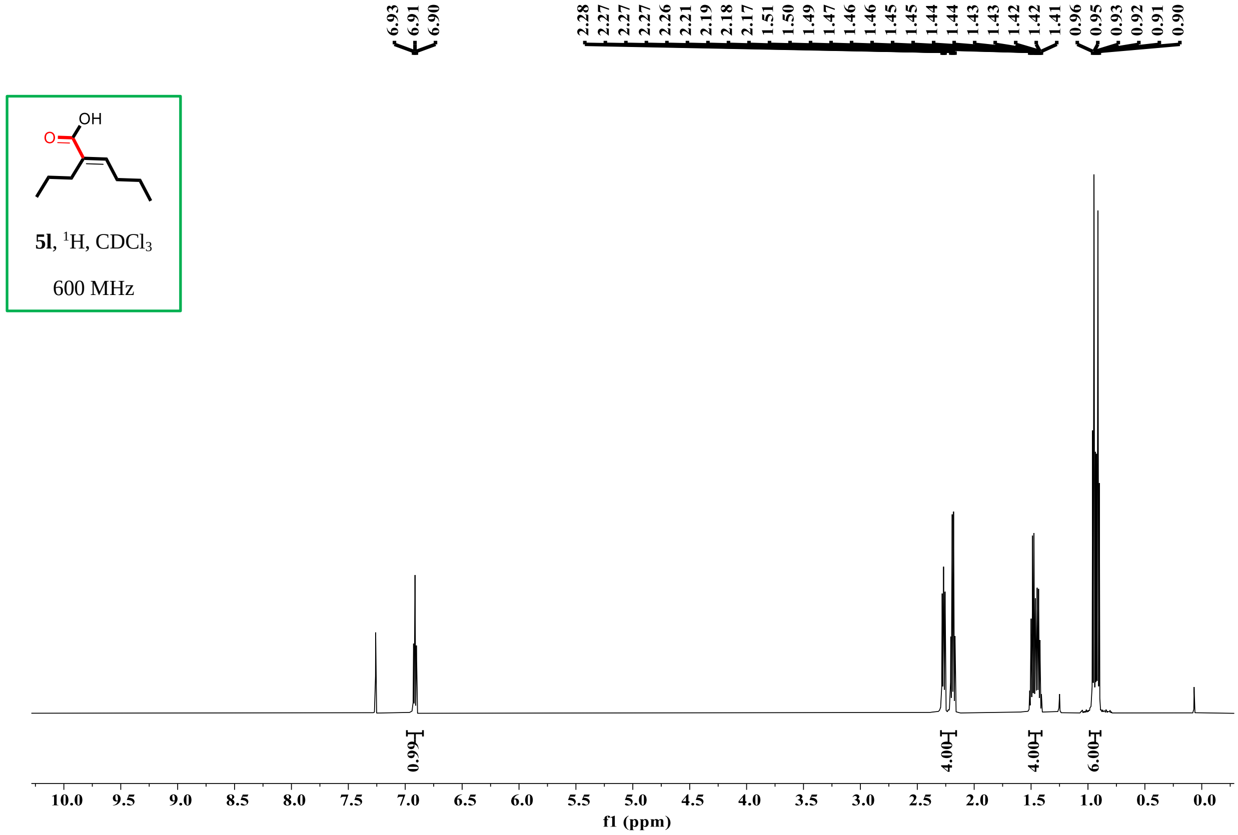

6.93
6.91
6.90
2.28
2.27
2.27
2.27
2.26
2.21
2.19
2.18
2.17
1.51
1.50
1.49
1.47
1.46
1.46
1.45
1.45
1.44
1.44
1.43
1.43
1.42
1.42
1.41
0.96
0.95
0.93
0.92
0.91
0.90
OH
O
5l, 1H, CDCl3
600 MHz
0.99
4.00
4.00
6.00
10.0 9.5 9.0 8.5 8.0 7.5 7.0 6.5 6.0 5.5 5.0 4.5 4.0 3.5 3.0 2.5 2.0 1.5 1.0 0.5 0.0
f1 (ppm)

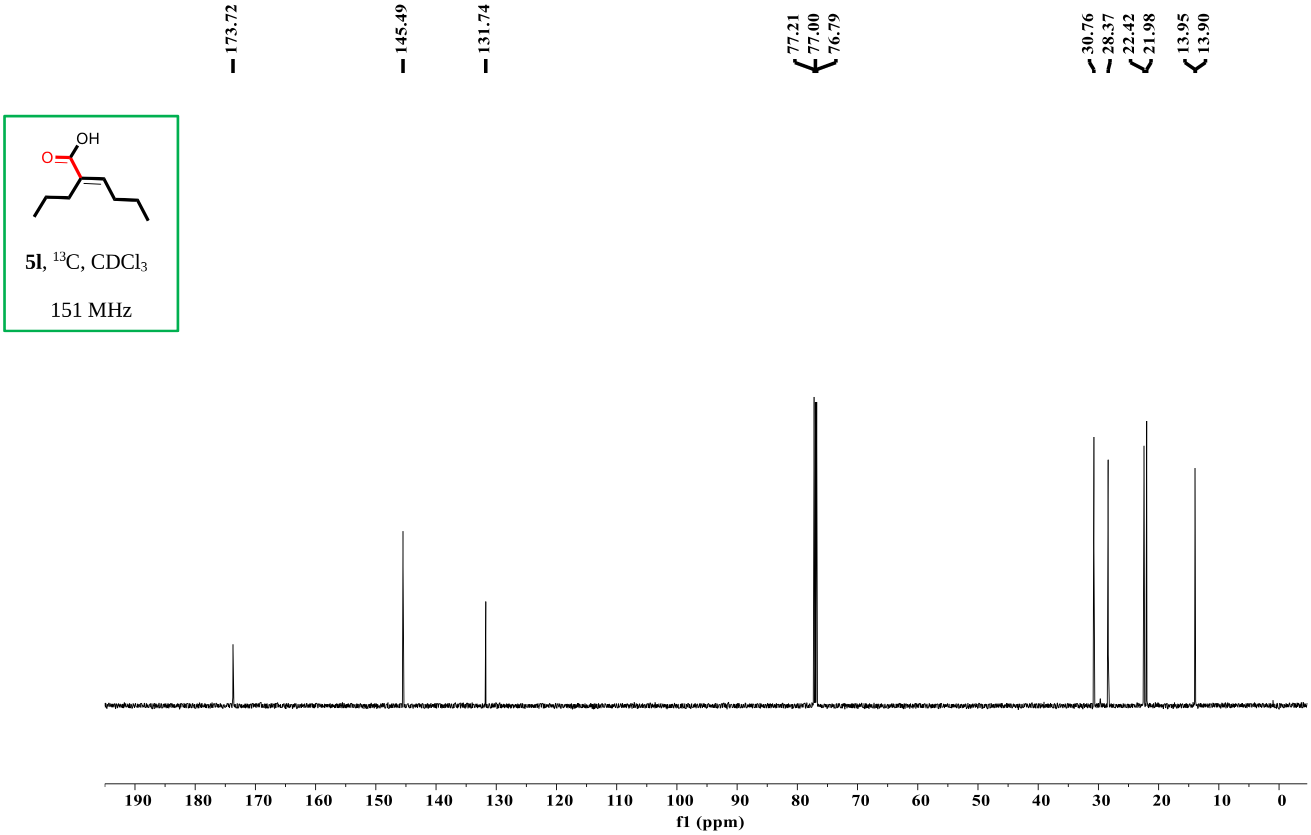

173.72
145.49
131.74
77.21
77.00
76.79
30.76
28.37
22.42
21.98
13.95
13.90
OH
O
5l, 13C, CDCl3
151 MHz
190 180 170 160 150 140 130 120 110 100 90 80 70 60 50 40 30 20 10 0
f1 (ppm)

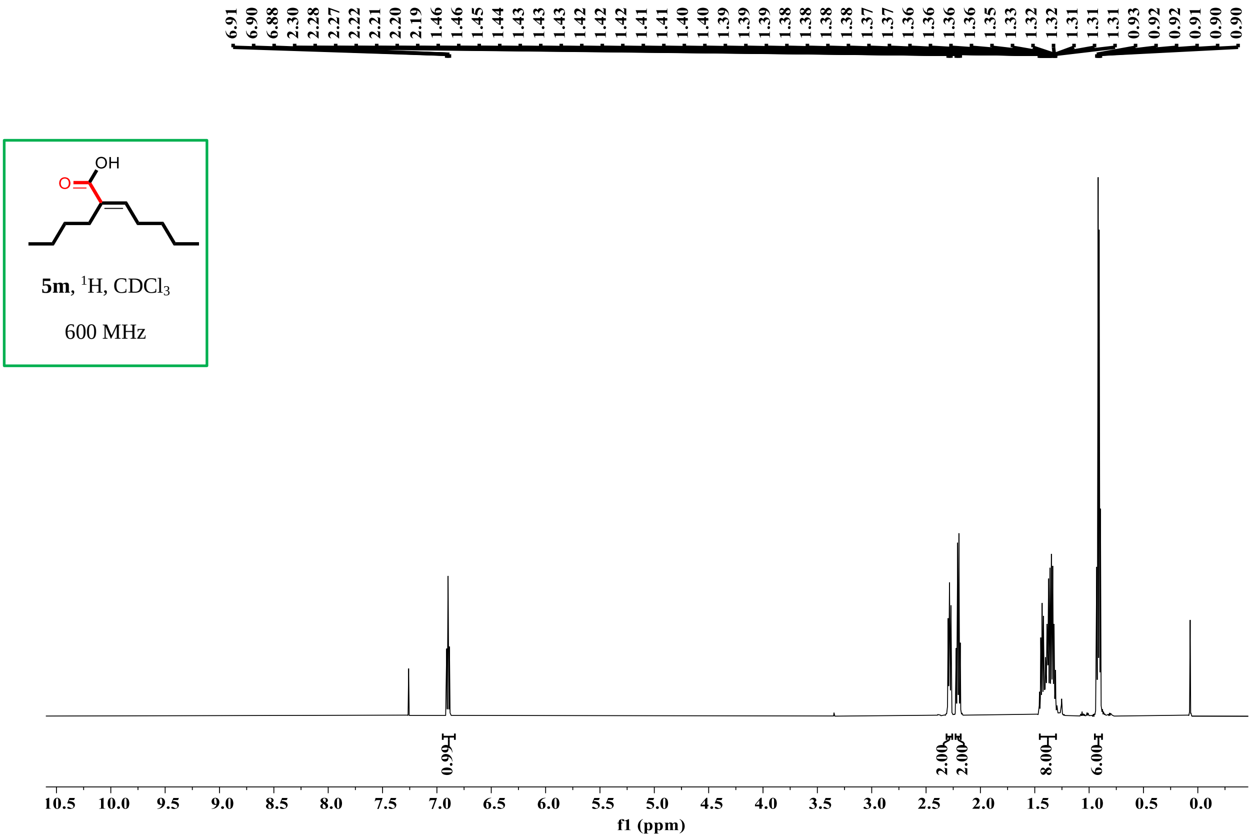
6.91 6.90 6.88 2.30 2.28 2.27 2.22 2.21 2.20 2.19 1.46 1.46 1.45 1.44 1.43 1.43 1.43 1.42 1.42 1.42 1.41 1.41 1.40 1.40 1.39 1.39 1.39 1.38 1.38 1.38 1.38 1.38 1.37 1.37 1.36 1.36 1.36 1.36 1.35 1.33 1.32 1.32 1.31 1.31 1.31 0.93 0.92 0.92 0.91 0.90 0.90
OH
O
5m, 1H, CDCl3
600 MHz
0.99
2.00
2.00
8.00
6.00
10.5 10.0 9.5 9.0 8.5 8.0 7.5 7.0 6.5 6.0 5.5 5.0 4.5 4.0 3.5 3.0 2.5 2.0 1.5 1.0 0.5 0.0
f1 (ppm)

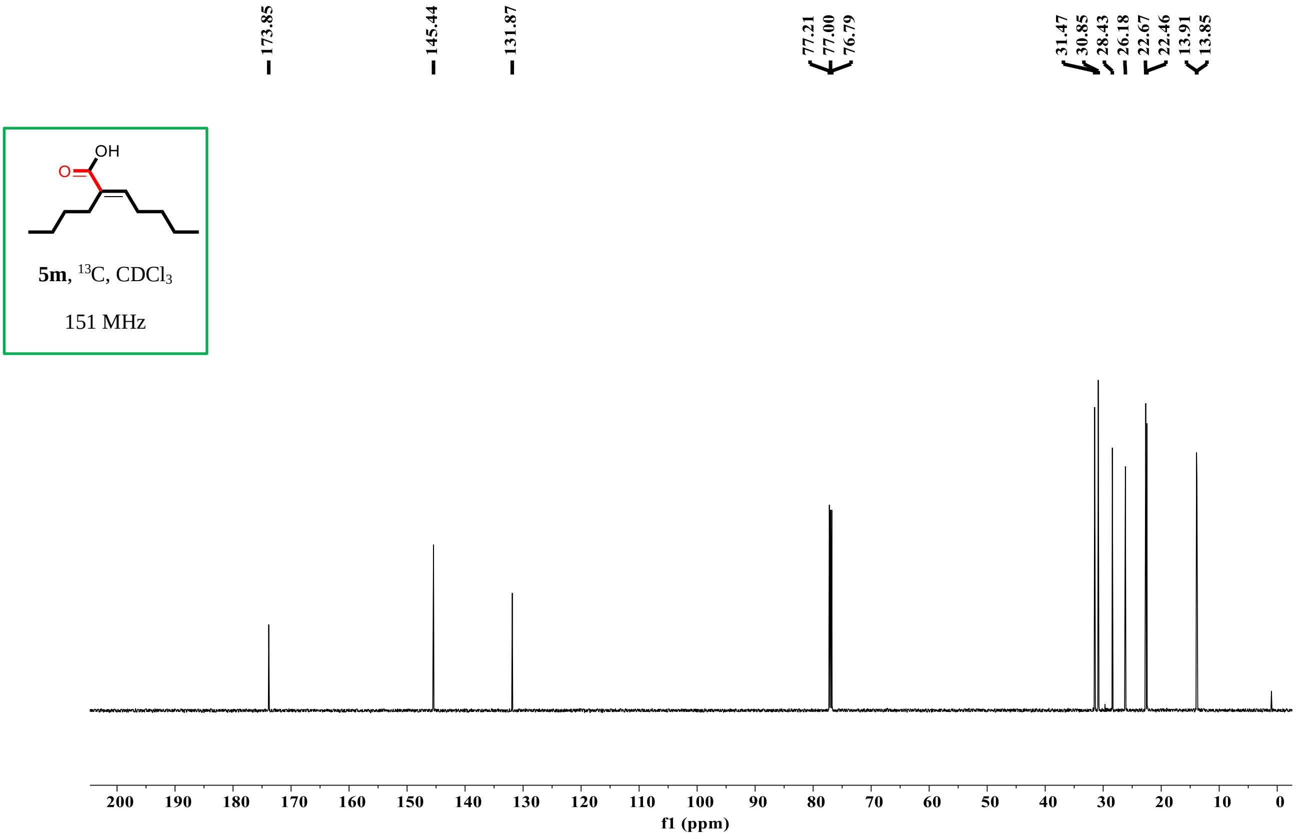
173.85
145.44
131.87
77.21 77.00 76.79
31.47 30.85 28.43 26.18 22.67 22.46 13.91 13.85
OH
O
5m, 13C, CDCl3
151 MHz
200 190 180 170 160 150 140 130 120 110 100 90 80 70 60 50 40 30 20 10 0
f1 (ppm)

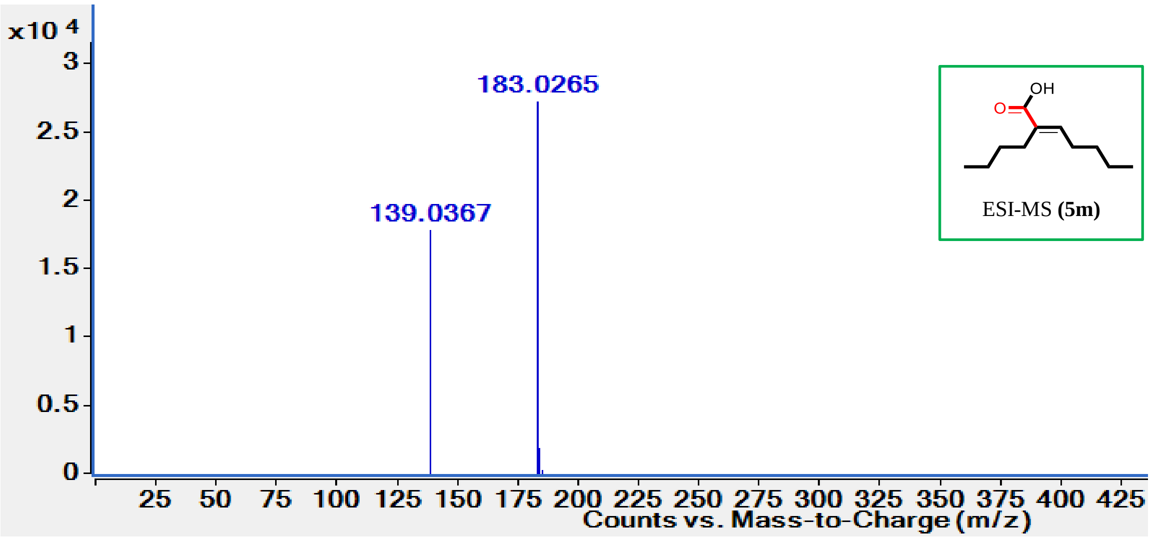
x10 4
3
2.5
2
1.5
1
0.5
0
183.0265
139.0367
25 50 75 100 125 150 175 200 225 250 275 300 325 350 375 400 425
Counts vs. Mass-to-Charge (m/z)
OH
O
ESI-MS (5m)

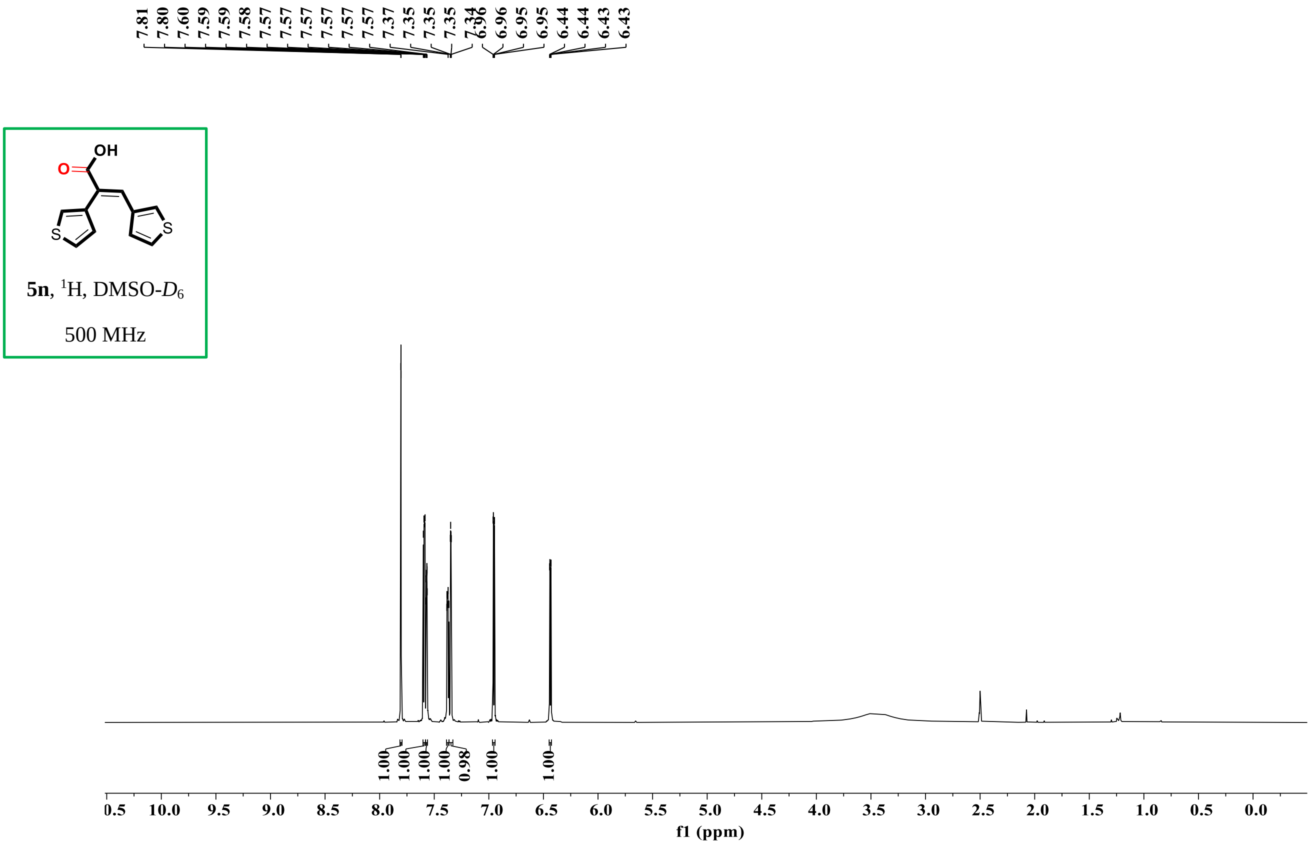
7.81 7.80 7.60 7.59 7.59 7.58 7.57 7.57 7.57 7.57 7.57 7.57 7.37 7.35 7.35 7.35 7.34 6.96 6.96 6.95 6.95 6.44 6.44 6.43 6.43
OH
O
S
S
5n, 1H, DMSO-D6
500 MHz
1.00 1.00 1.00 1.00 0.98 1.00 1.00
0.5 10.0 9.5 9.0 8.5 8.0 7.5 7.0 6.5 6.0 5.5 5.0 4.5 4.0 3.5 3.0 2.5 2.0 1.5 1.0 0.5 0.0
f1 (ppm)

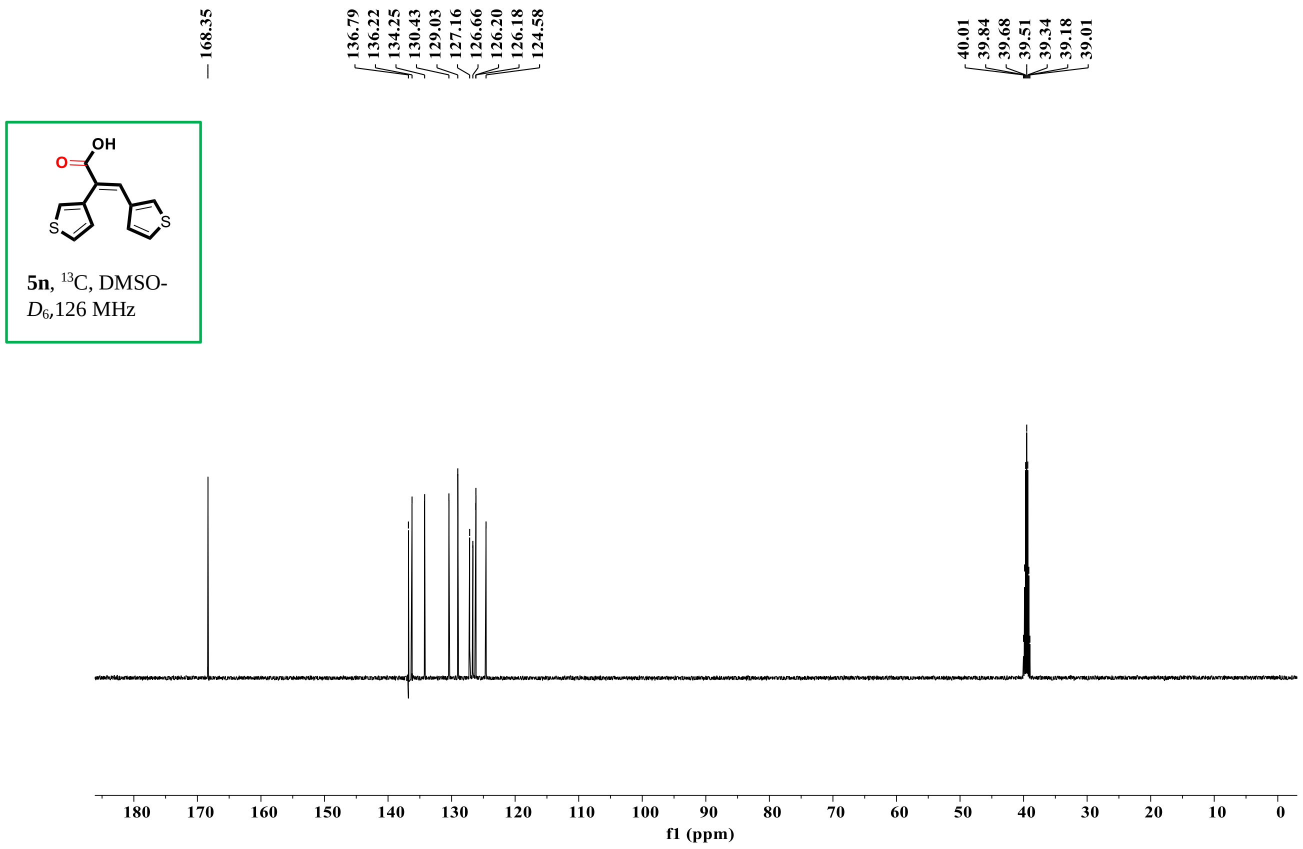
168.35
136.79
136.22
134.25
130.43
129.03
127.16
126.66
126.20
126.18
124.58
40.01
39.84
39.68
39.51
39.34
39.18
39.01
OH
O
S
S
5n, ^{13}C, DMSO-D_6,126 MHz
180 170 160 150 140 130 120 110 100 90 80 70 60 50 40 30 20 10 0
f1 (ppm)

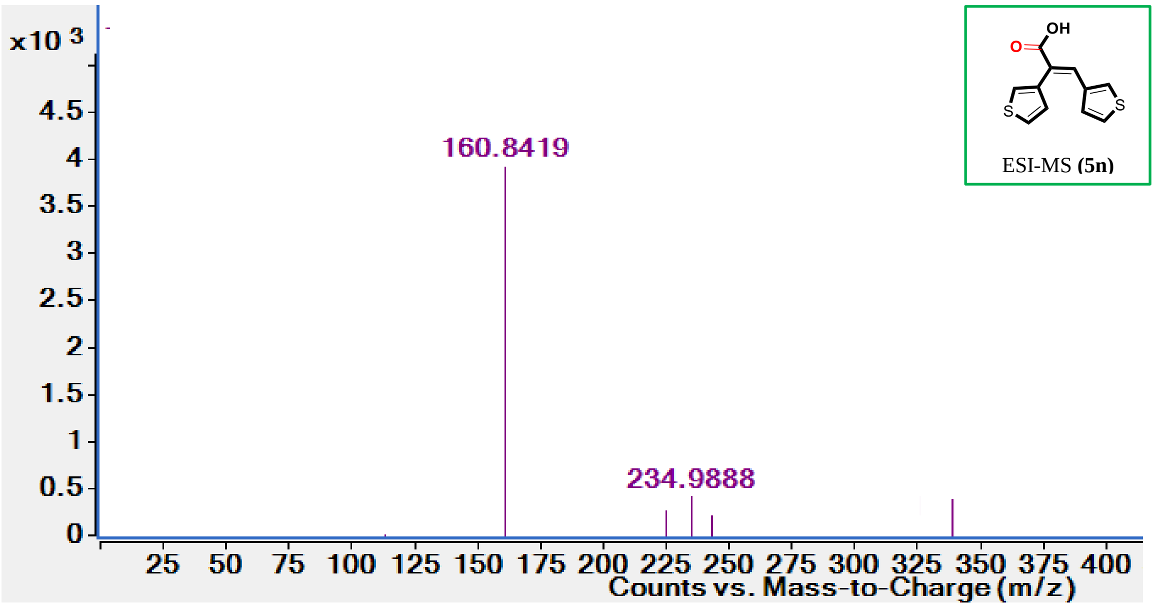
x10 3
4.5
4
3.5
3
2.5
2
1.5
1
0.5
0
160.8419
234.9888
25 50 75 100 125 150 175 200 225 250 275 300 325 350 375 400
Counts vs. Mass-to-Charge (m/z)
OH
O
S
S
ESI-MS (5n)

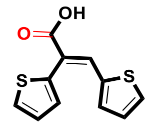

5o, ^{1}H, DMSO-D_6

126 MHz

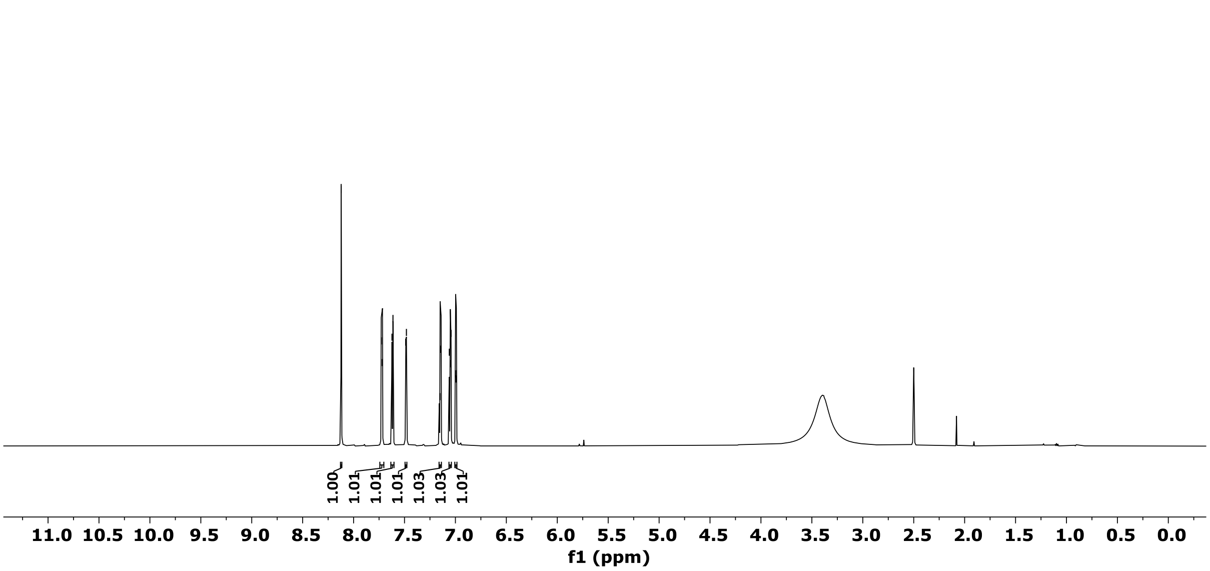

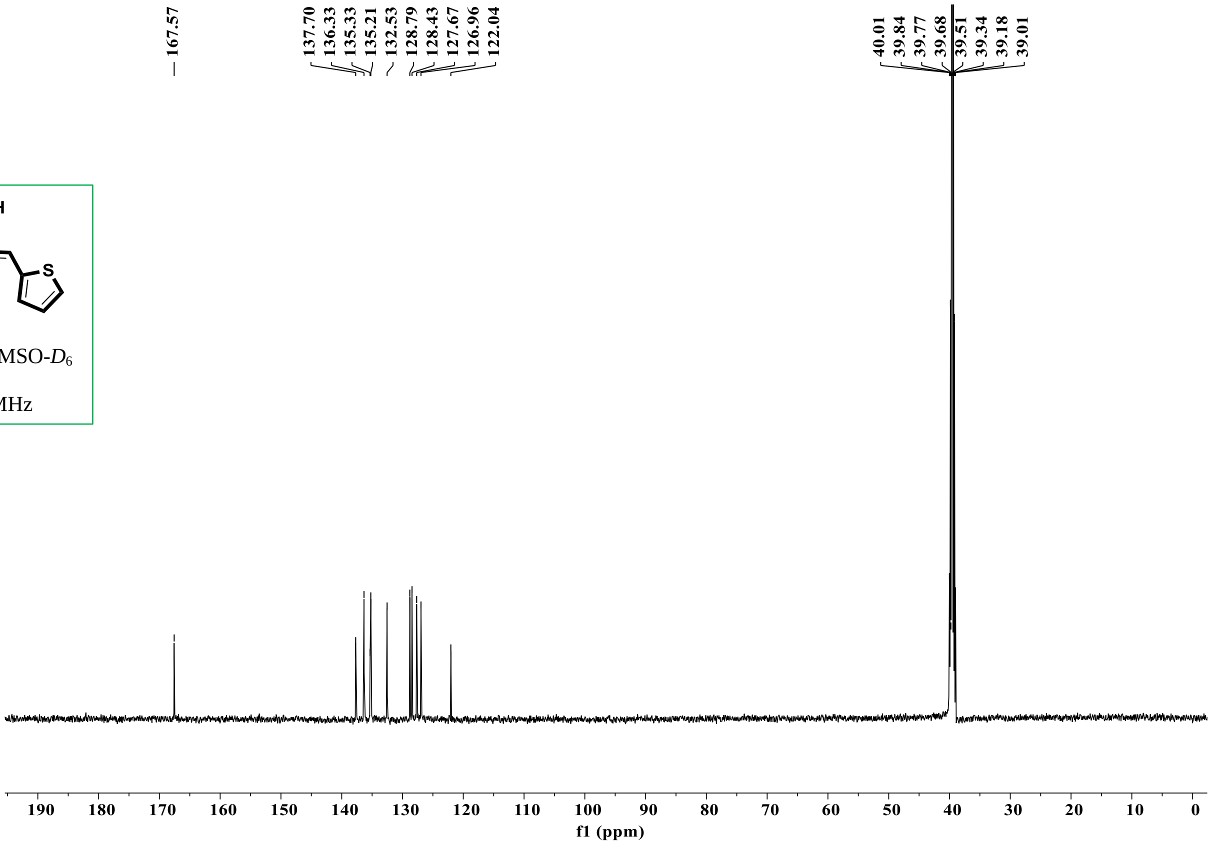

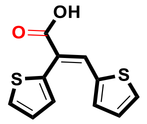

5o, ^{13}C, DMSO-D_6

126 MHz

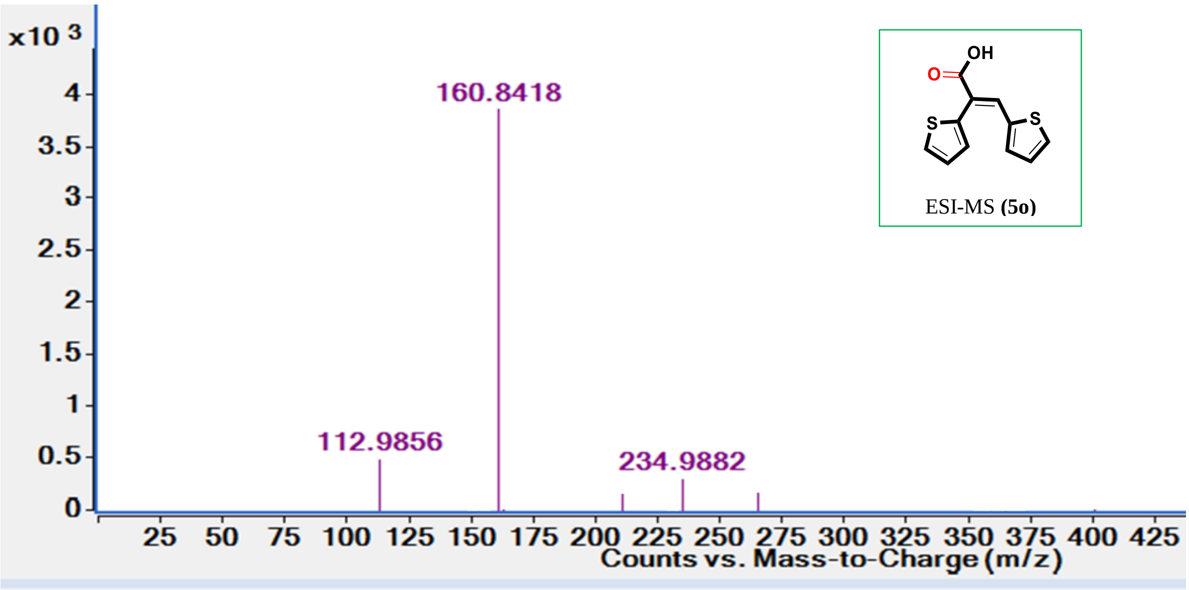
x10 3
4
3.5
3
2.5
2
1.5
1
0.5
0
160.8418
112.9856
234.9882
25 50 75 100 125 150 175 200 225 250 275 300 325 350 375 400 425
Counts vs. Mass-to-Charge (m/z)
OH
O
S
S
ESI-MS **(5o)**

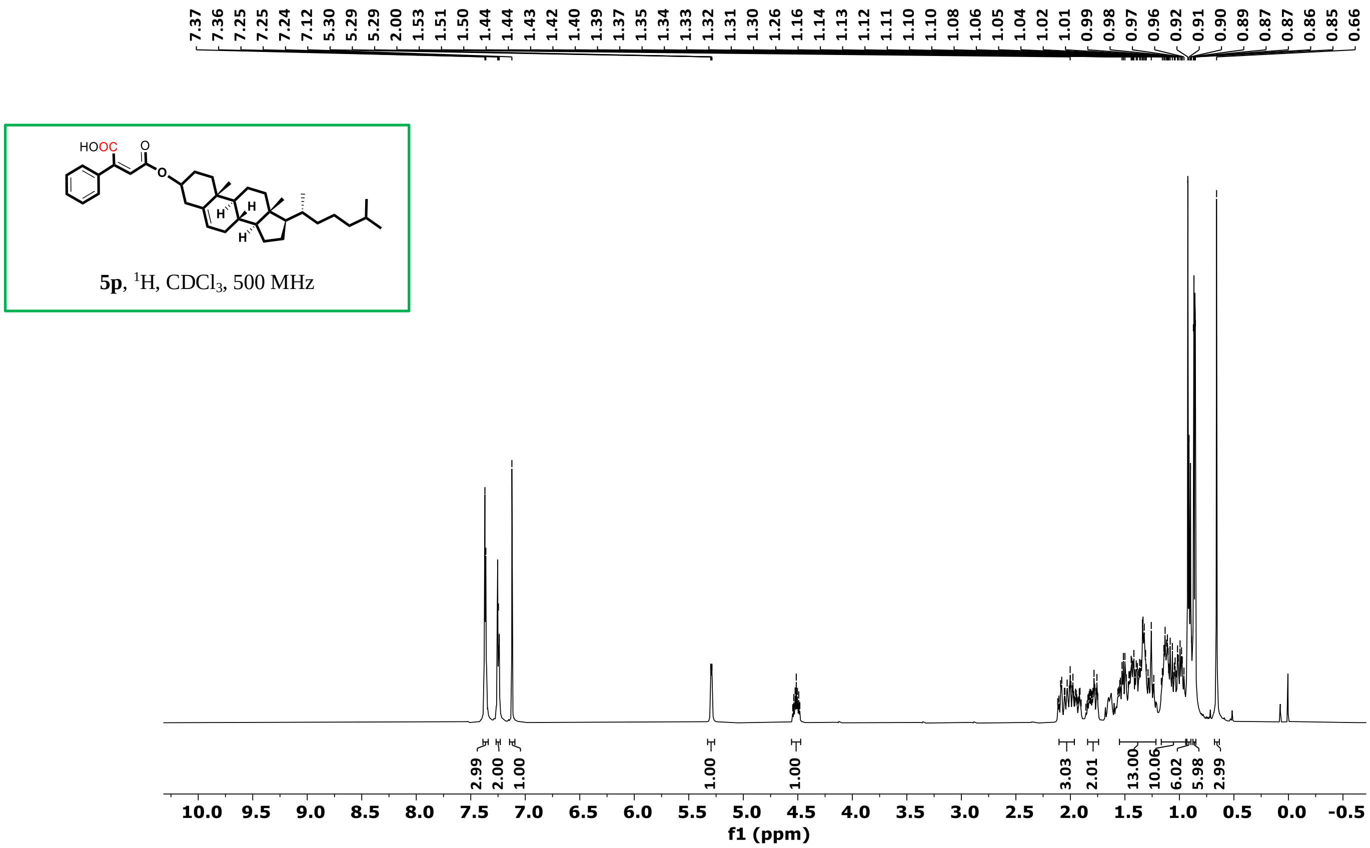
7.37 7.36 7.25 7.25 7.24 7.12 5.30 5.29 5.29 2.00 1.53 1.51 1.50 1.44 1.44 1.43 1.42 1.40 1.39 1.37 1.35 1.34 1.33 1.32 1.31 1.30 1.26 1.16 1.14 1.13 1.12 1.11 1.10 1.10 1.08 1.06 1.05 1.04 1.02 1.01 0.99 0.98 0.97 0.96 0.92 0.91 0.90 0.89 0.87 0.87 0.86 0.85 0.66
HOOC
O
H
H
H
5p, 1H, CDCl3, 500 MHz
2.99 2.00 1.00 1.00 1.00 3.03 2.01 13.00 10.06 6.02 5.98 2.99
10.0 9.5 9.0 8.5 8.0 7.5 7.0 6.5 6.0 5.5 5.0 4.5 4.0 3.5 3.0 2.5 2.0 1.5 1.0 0.5 0.0 -0.5
f1 (ppm)

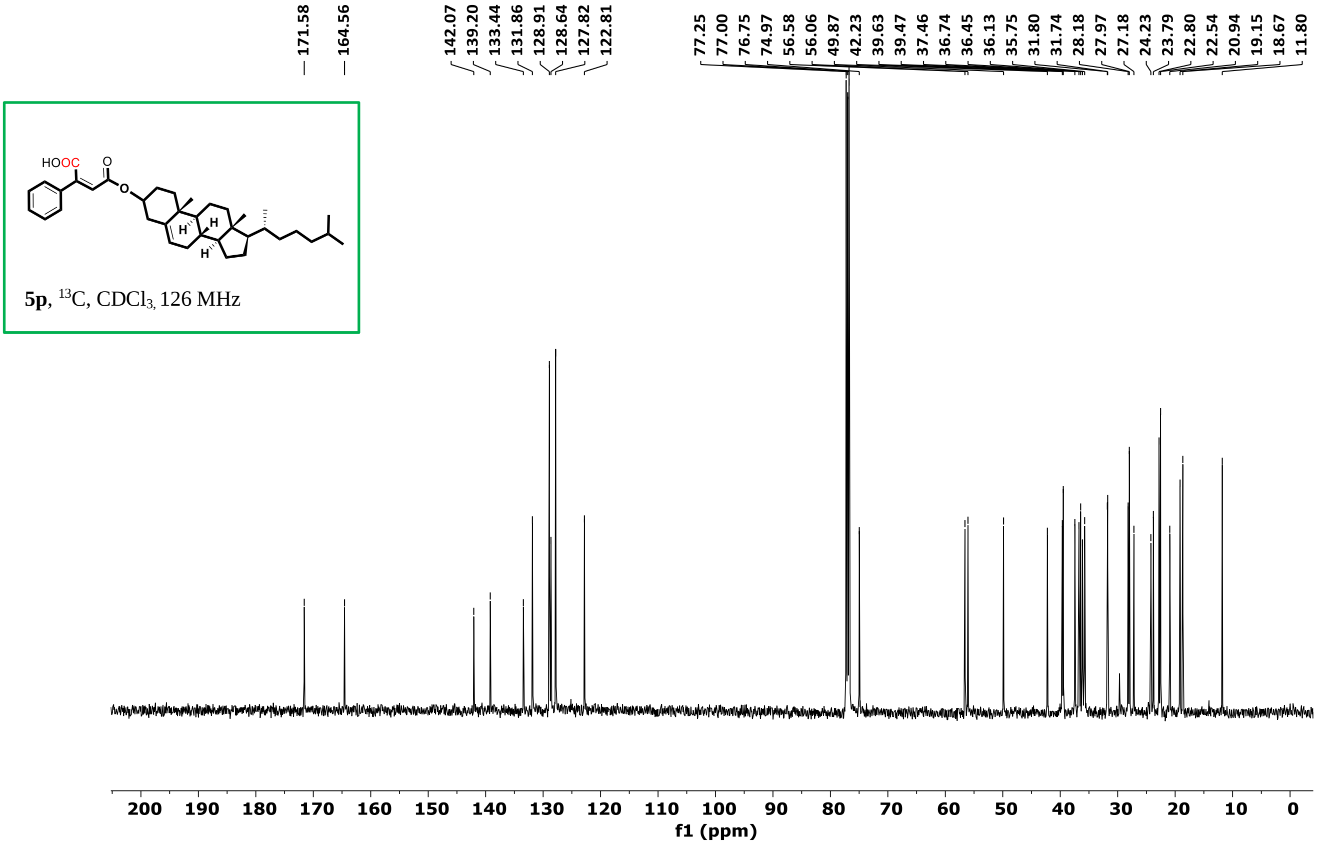
171.58 164.56 142.07 139.20 133.44 131.86 128.91 128.64 127.82 122.81 77.25 77.00 76.75 74.97 56.58 56.06 49.87 42.23 39.63 39.47 37.46 36.74 36.45 36.13 35.75 31.80 31.74 28.18 27.97 27.18 24.23 23.79 22.80 22.54 20.94 19.15 18.67 11.80
HOOC
O
H
H
H
5p, 13C, CDCl3, 126 MHz
200 190 180 170 160 150 140 130 120 110 100 90 80 70 60 50 40 30 20 10 0
f1 (ppm)

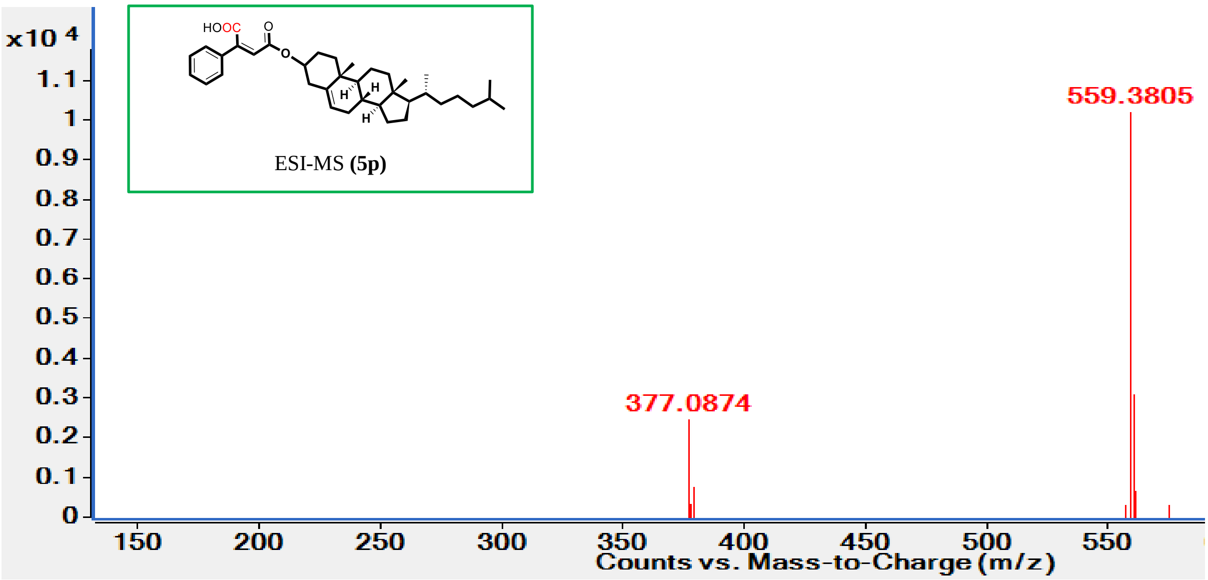

x10 4
559.3805
377.0874
Counts vs. Mass-to-Charge (m/z)
ESI-MS (5p)

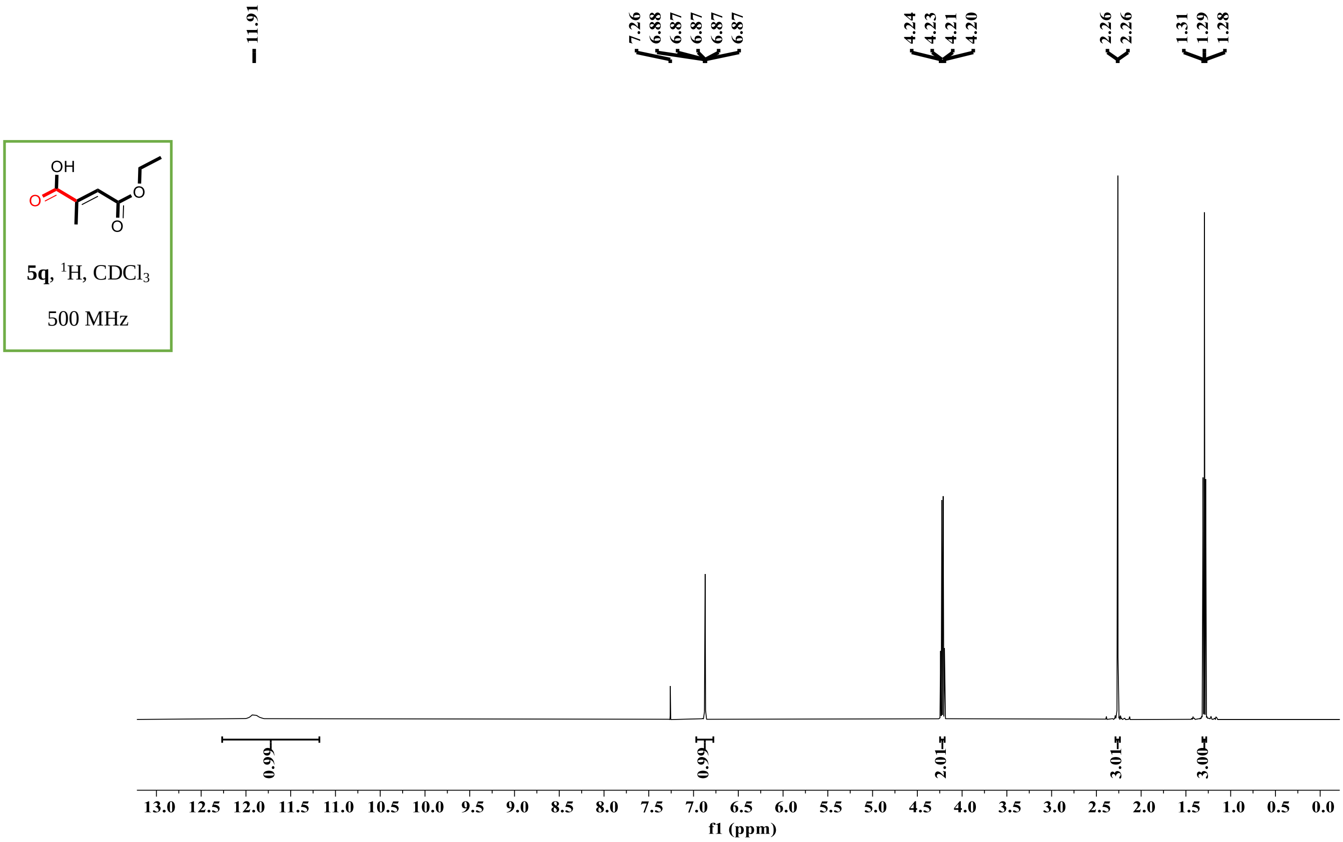

11.91
7.26
6.88
6.87
6.87
6.87
6.87
4.24
4.23
4.21
4.20
2.26
2.26
1.31
1.29
1.28
5q, ¹H, CDCl3
500 MHz
0.99
0.99
2.01
3.01
3.00
f1 (ppm)

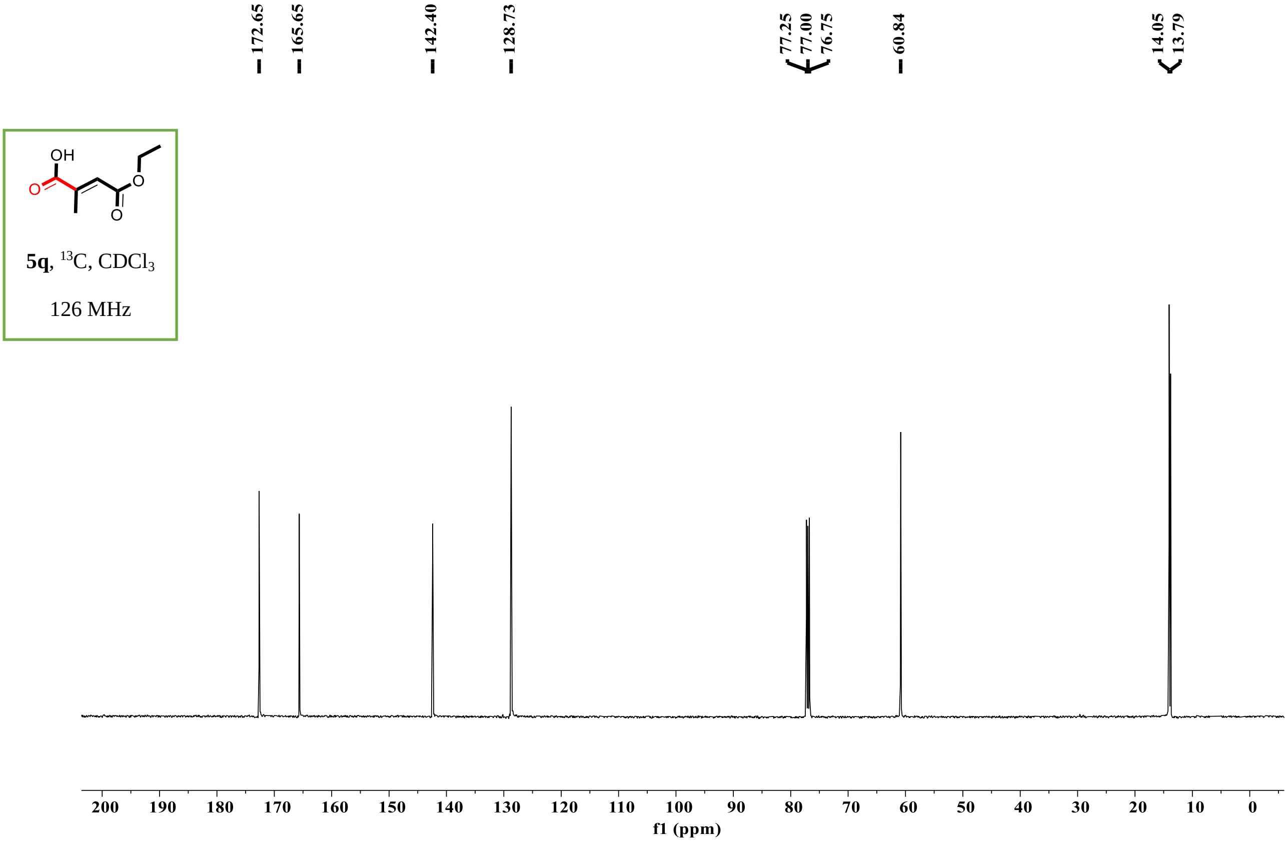
172.65
165.65
142.40
128.73
77.25
77.00
76.75
60.84
14.05
13.79
OH
O
O
O
5q, ¹³C, CDCl₃
126 MHz
200 190 180 170 160 150 140 130 120 110 100 90 80 70 60 50 40 30 20 10 0
f1 (ppm)

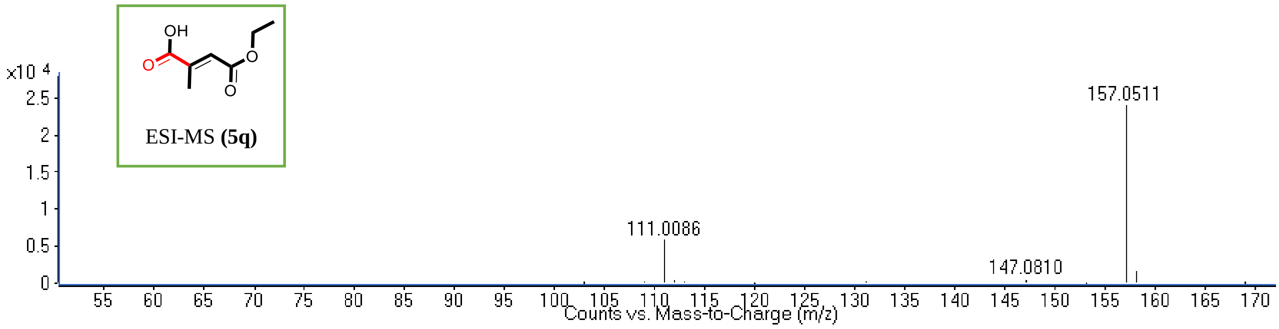
OH
O
O
O
ESI-MS (5q)
x10 4
2.5
2
1.5
1
0.5
0
157.0511
111.0086
147.0810
55 60 65 70 75 80 85 90 95 100 105 110 115 120 125 130 135 140 145 150 155 160 165 170
Counts vs. Mass-to-Charge (m/z)

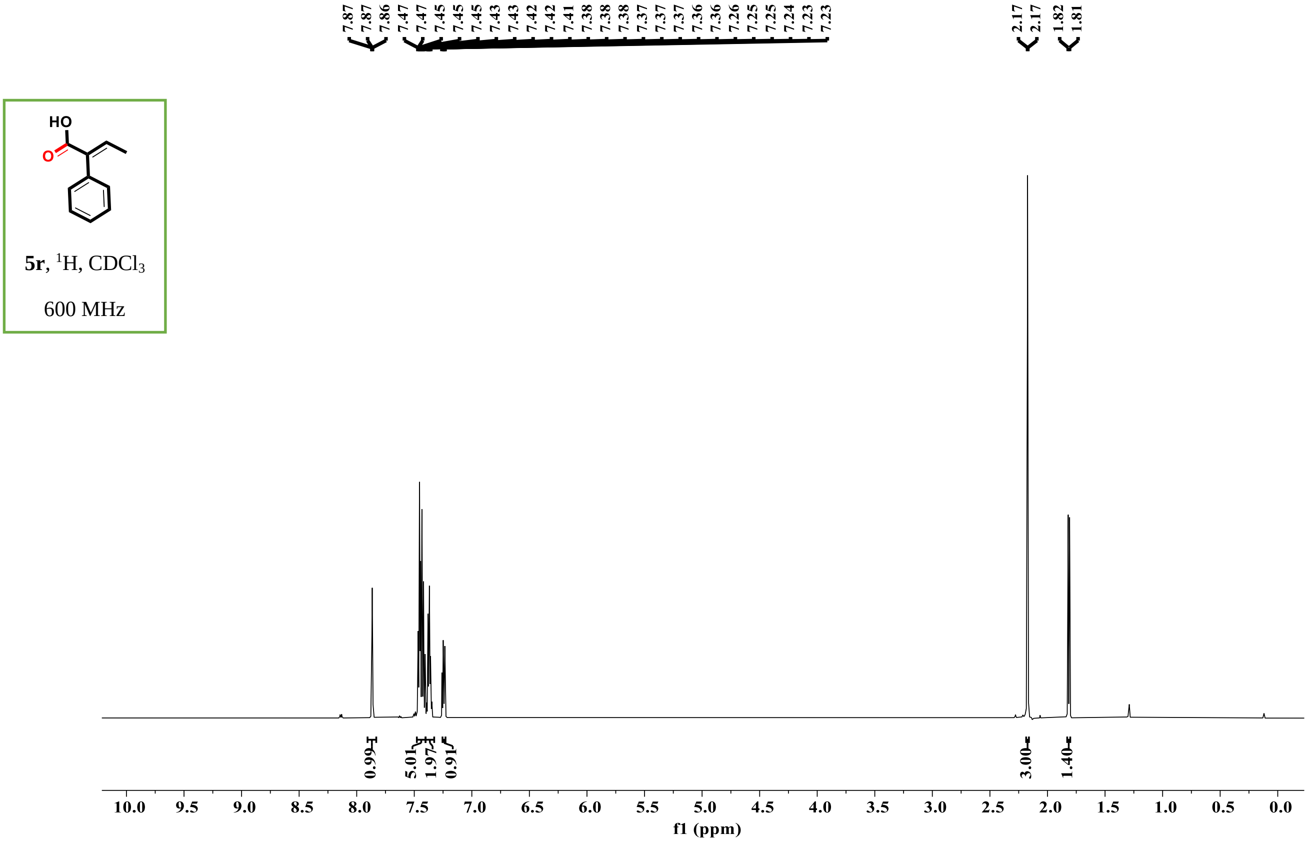
7.87
7.87
7.86
7.47
7.47
7.45
7.45
7.43
7.43
7.42
7.42
7.41
7.38
7.38
7.38
7.37
7.37
7.36
7.36
7.26
7.25
7.25
7.24
7.23
7.23
2.17
2.17
1.82
1.81
HO
O
5r, ^{1}H, CDCl$_3$
600 MHz
0.99
5.01
1.97
0.91
3.00
1.40
10.0 9.5 9.0 8.5 8.0 7.5 7.0 6.5 6.0 5.5 5.0 4.5 4.0 3.5 3.0 2.5 2.0 1.5 1.0 0.5 0.0
f1 (ppm)

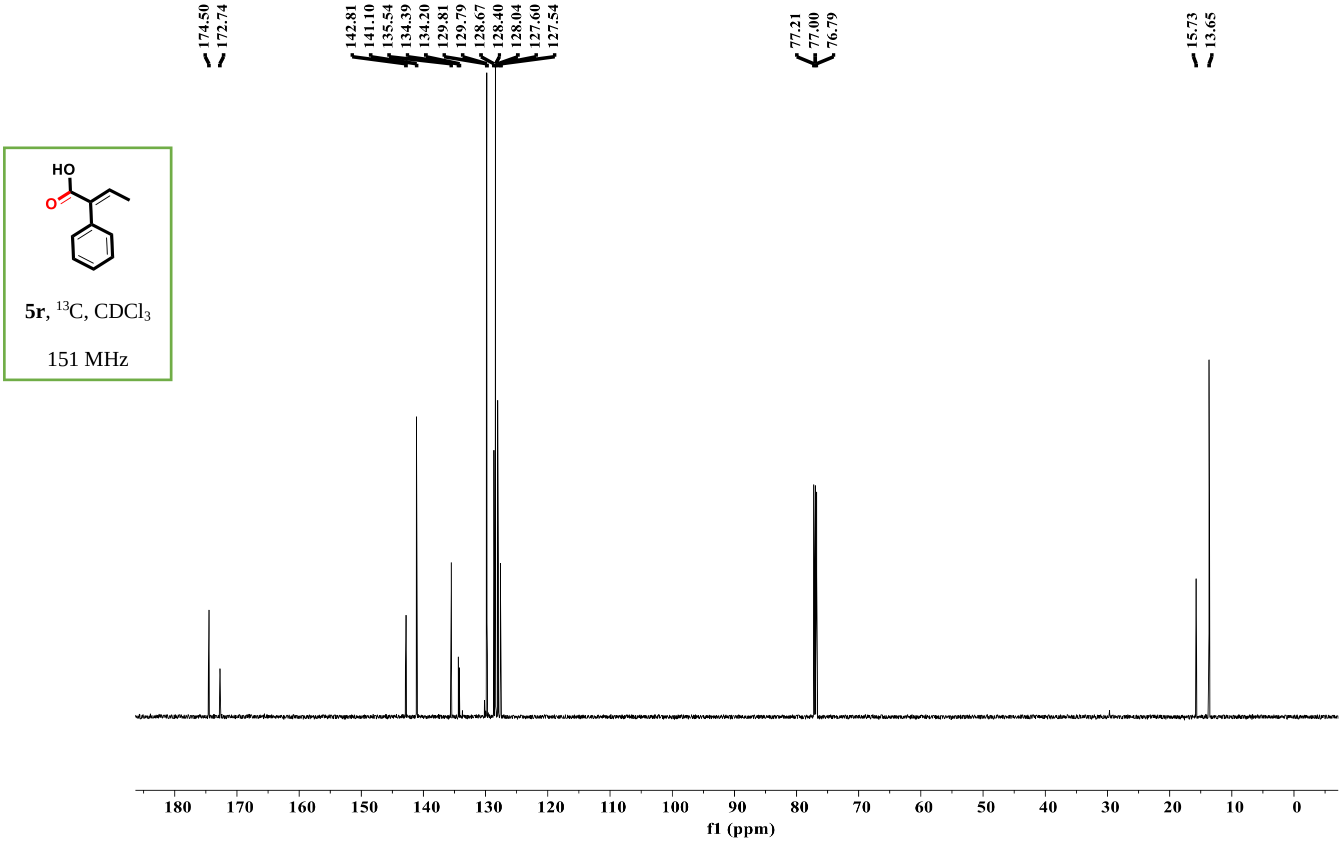
174.50
172.74
142.81
141.10
135.54
134.39
134.20
129.81
129.79
128.67
128.40
128.04
127.60
127.54
77.21
77.00
76.79
15.73
13.65
HO
O
5r, ^{13}C, CDCl$_3$
151 MHz
180 170 160 150 140 130 120 110 100 90 80 70 60 50 40 30 20 10 0
f1 (ppm)

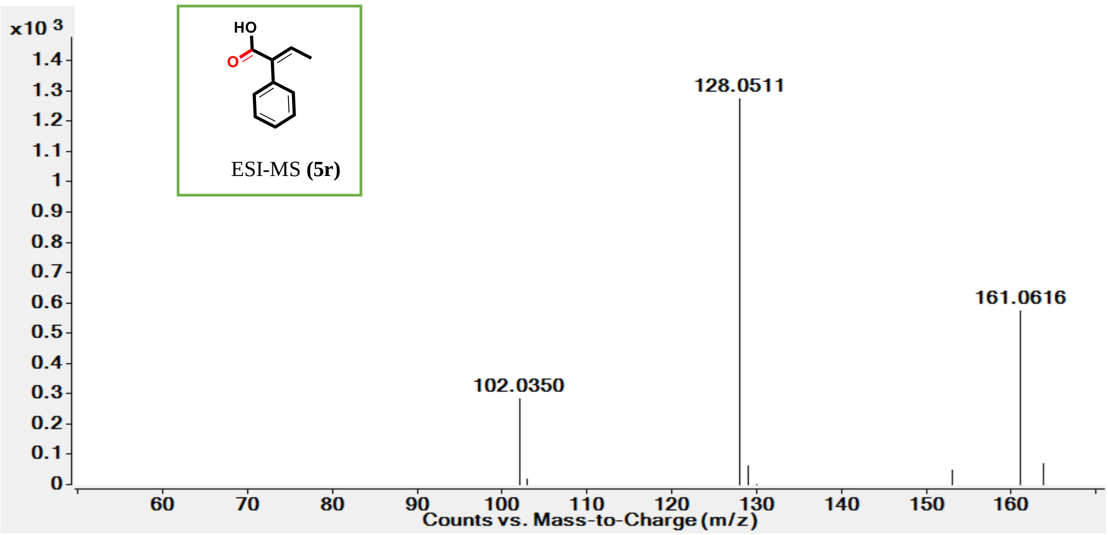

x10 3
1.4
1.3
1.2
1.1
1
0.9
0.8
0.7
0.6
0.5
0.4
0.3
0.2
0.1
0
HO
O
ESI-MS (5r)
128.0511
161.0616
102.0350
60
70
80
90
100
110
120
130
140
150
160
Counts vs. Mass-to-Charge (m/z)

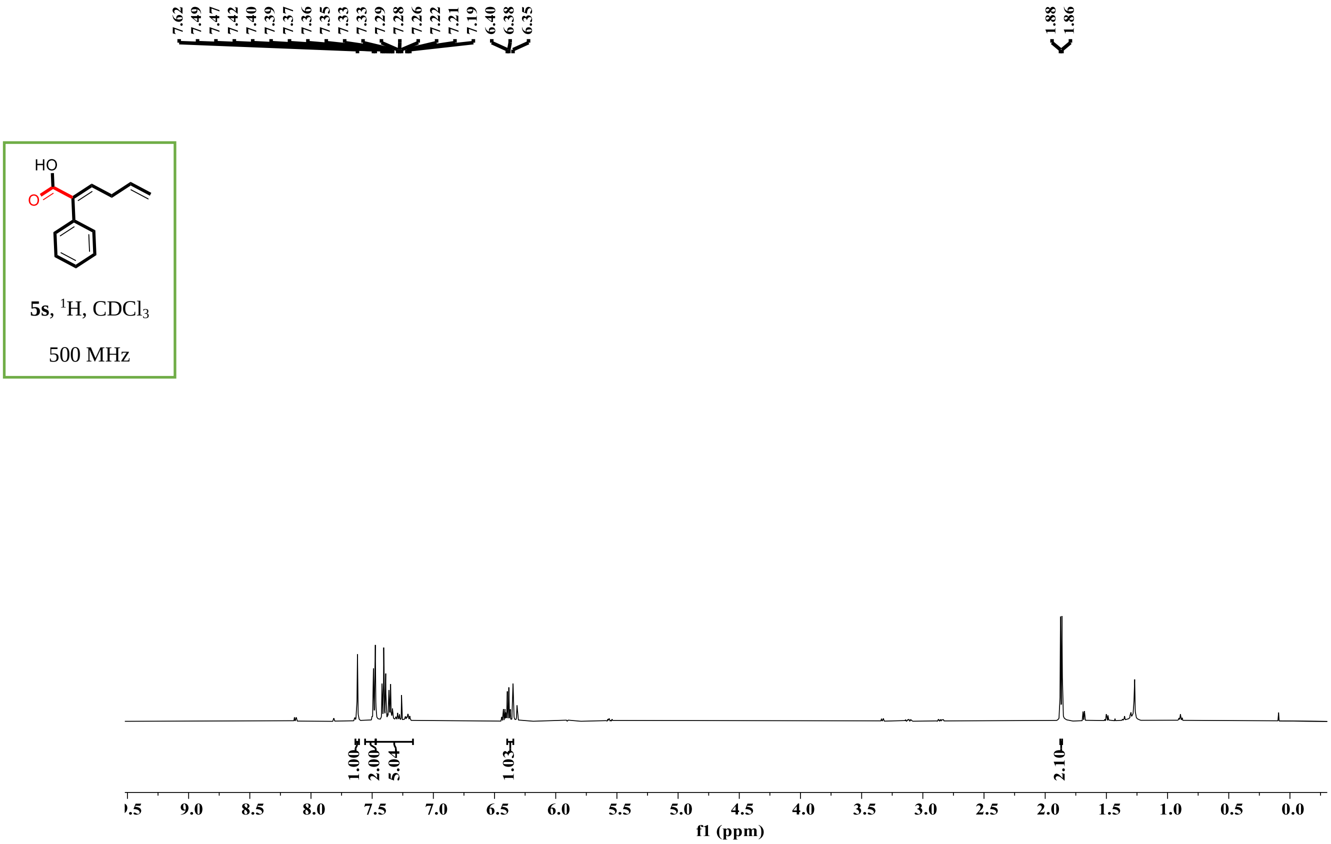

7.62
7.49
7.47
7.42
7.40
7.39
7.37
7.36
7.35
7.33
7.33
7.29
7.28
7.26
7.22
7.21
7.19
6.40
6.38
6.35
1.88
1.86
HO
O
5s, ¹H, CDCl3
500 MHz
1.00
2.00
5.04
1.03
2.10
9.5
9.0
8.5
8.0
7.5
7.0
6.5
6.0
5.5
5.0
4.5
4.0
3.5
3.0
2.5
2.0
1.5
1.0
0.5
0.0
f1 (ppm)

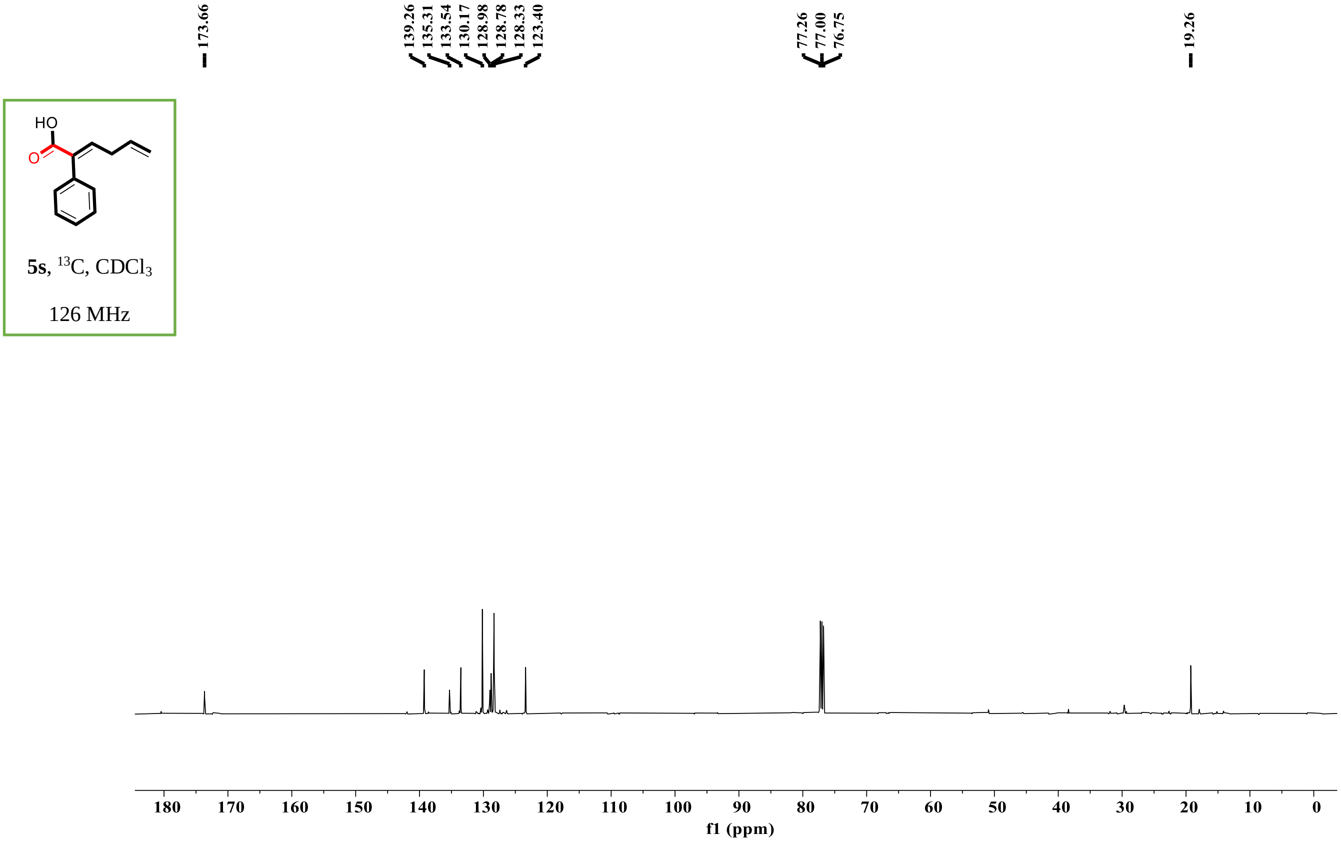
173.66
139.26
135.31
133.54
130.17
128.98
128.78
128.33
123.40
77.26
77.00
76.75
19.26
HO
O
5s, 13C, CDCl3
126 MHz
180 170 160 150 140 130 120 110 100 90 80 70 60 50 40 30 20 10 0
f1 (ppm)

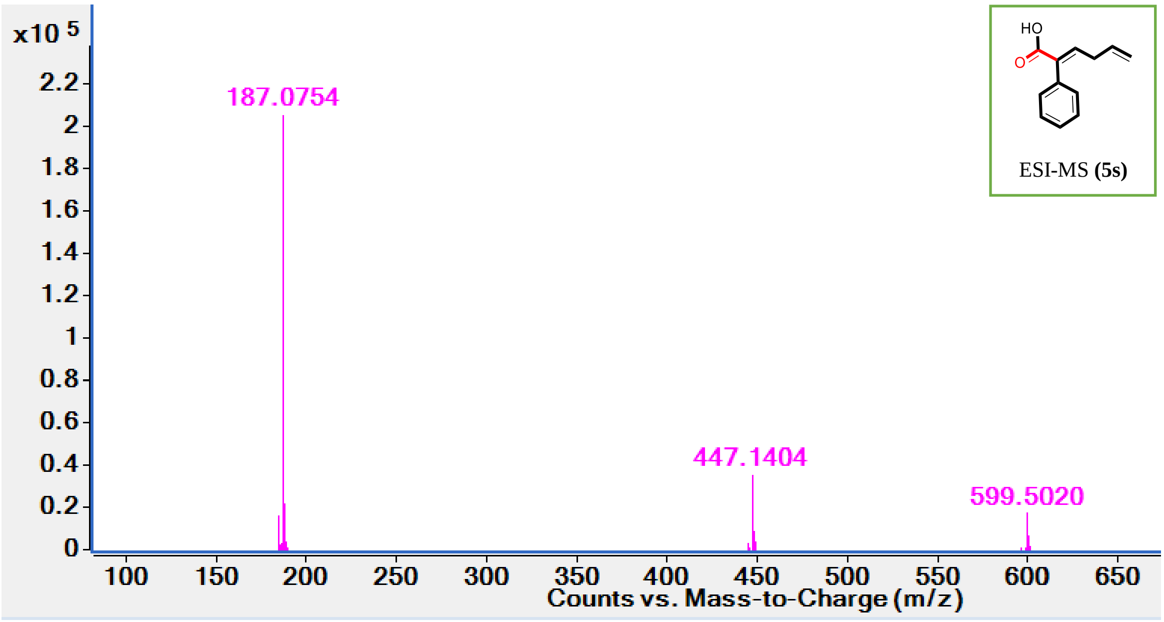
x10 5
2.2
2
1.8
1.6
1.4
1.2
1
0.8
0.6
0.4
0.2
0
187.0754
447.1404
599.5020
HO
O
ESI-MS (5s)
100 150 200 250 300 350 400 450 500 550 600 650
Counts vs. Mass-to-Charge (m/z)

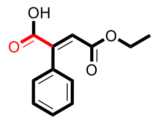

5t, ^{1}H, $CDCl_3$

600 MHz

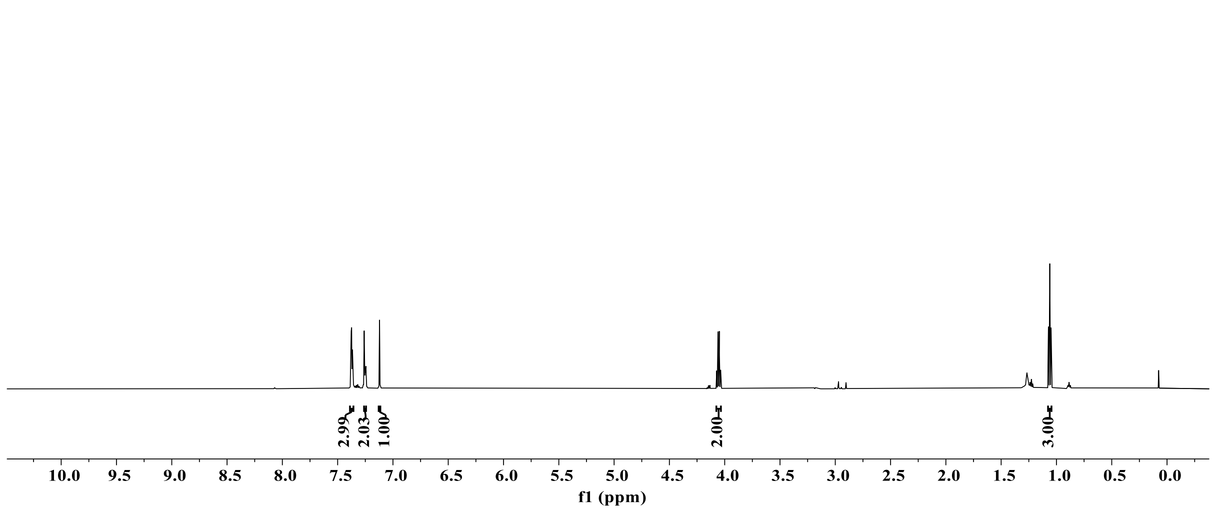

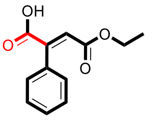

5t, ^{13}C, $CDCl_3$

126 MHz

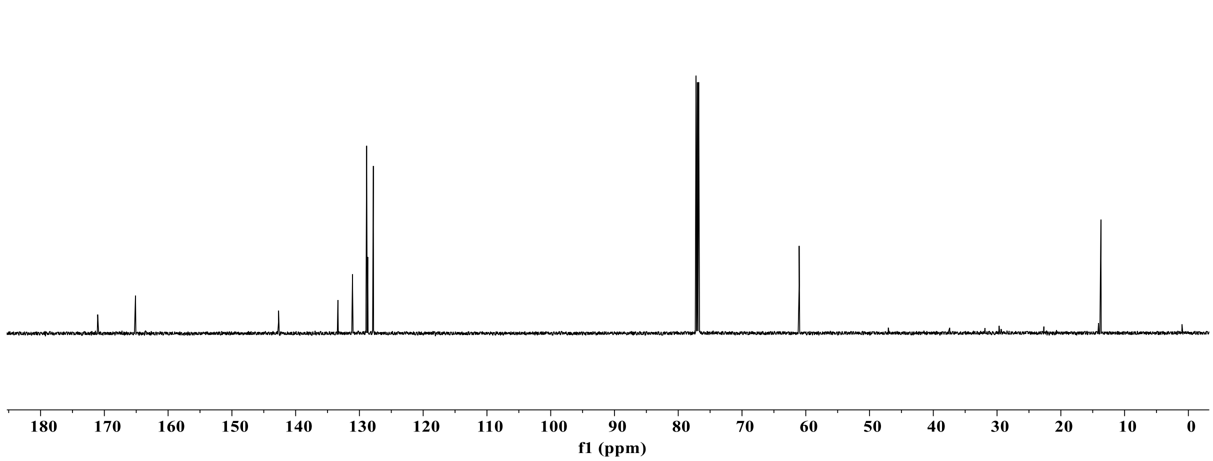

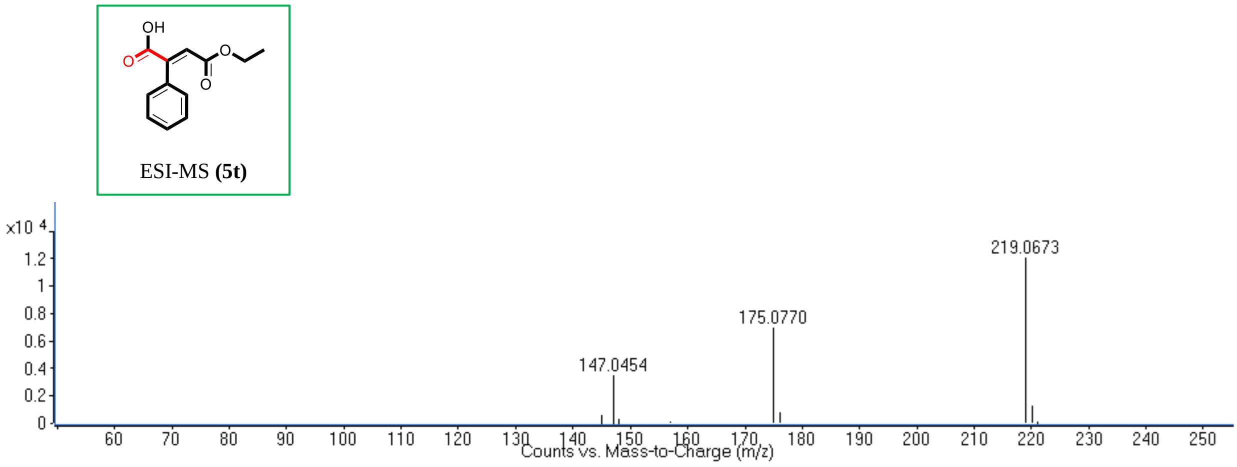
OH
O
O
O
ESI-MS (5t)
x10 4
1.2
1
0.8
0.6
0.4
0.2
0
219.0673
175.0770
147.0454
60
70
80
90
100
110
120
130
140
150
160
170
180
190
200
210
220
230
240
250
Counts vs. Mass-to-Charge (m/z)

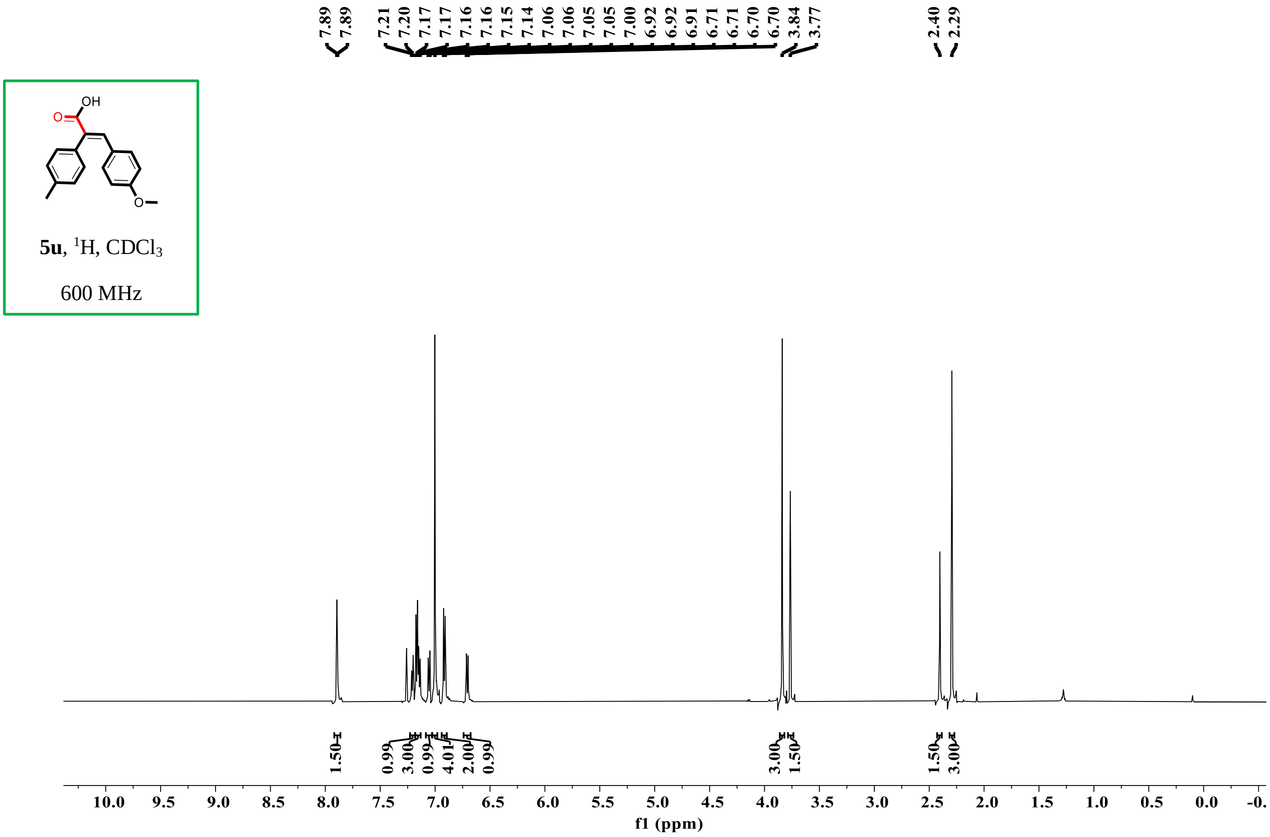
7.89
7.89
7.21
7.20
7.17
7.17
7.16
7.16
7.15
7.14
7.06
7.06
7.05
7.05
7.00
6.92
6.92
6.91
6.71
6.71
6.70
6.70
3.84
3.77
2.40
2.29
OH
O
O
5u, ^{1}H, $CDCl_3$
600 MHz
1.50
0.99
3.00
0.99
4.01
2.00
0.99
3.00
1.50
1.50
3.00
10.0
9.5
9.0
8.5
8.0
7.5
7.0
6.5
6.0
5.5
5.0
4.5
4.0
3.5
3.0
2.5
2.0
1.5
1.0
0.5
0.0
-0.
f1 (ppm)

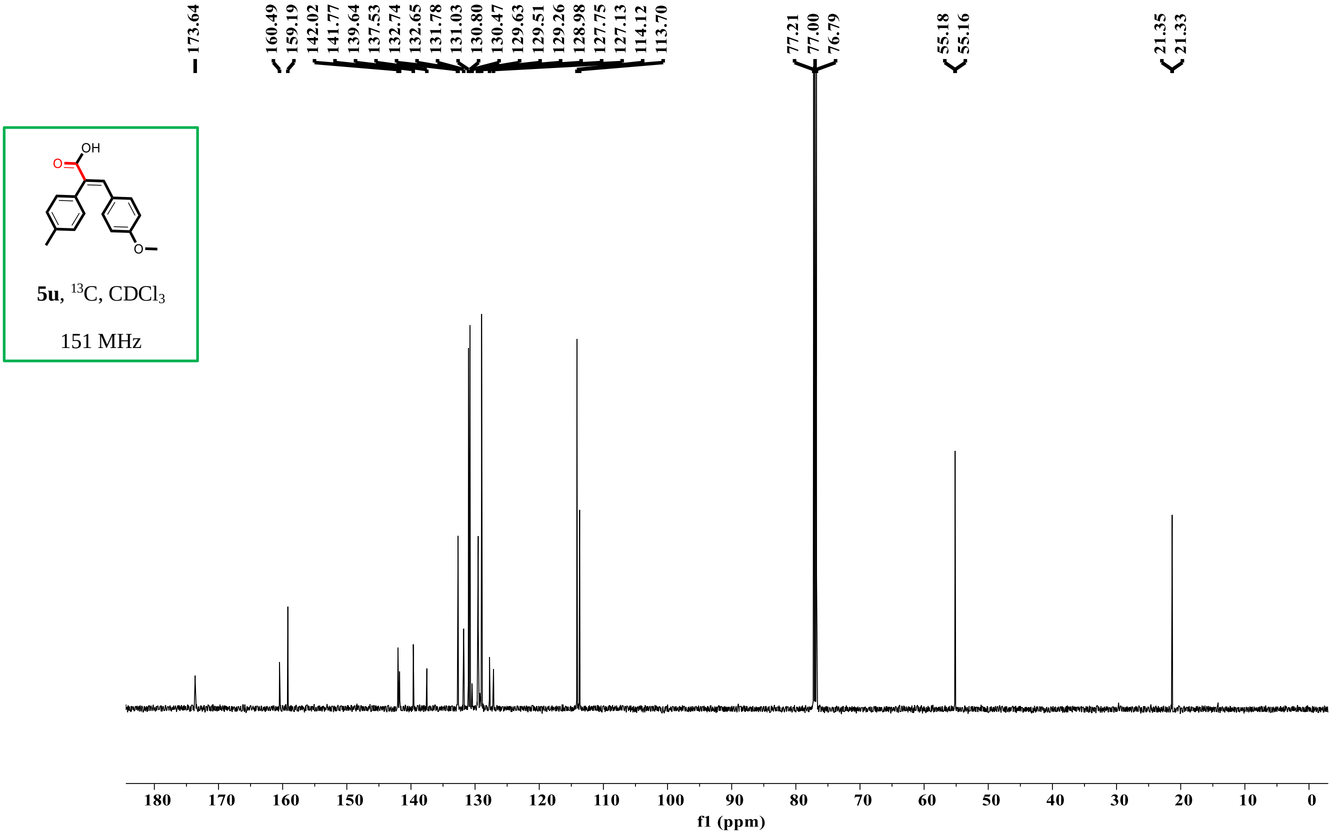

173.64
160.49
159.19
142.02
141.77
139.64
137.53
132.74
132.65
131.78
131.03
130.80
130.47
129.63
129.51
129.26
128.98
127.75
127.13
114.12
113.70
77.21
77.00
76.79
55.18
55.16
21.35
21.33
OH
O
O
5u, 13C, CDCl3
151 MHz
f1 (ppm)

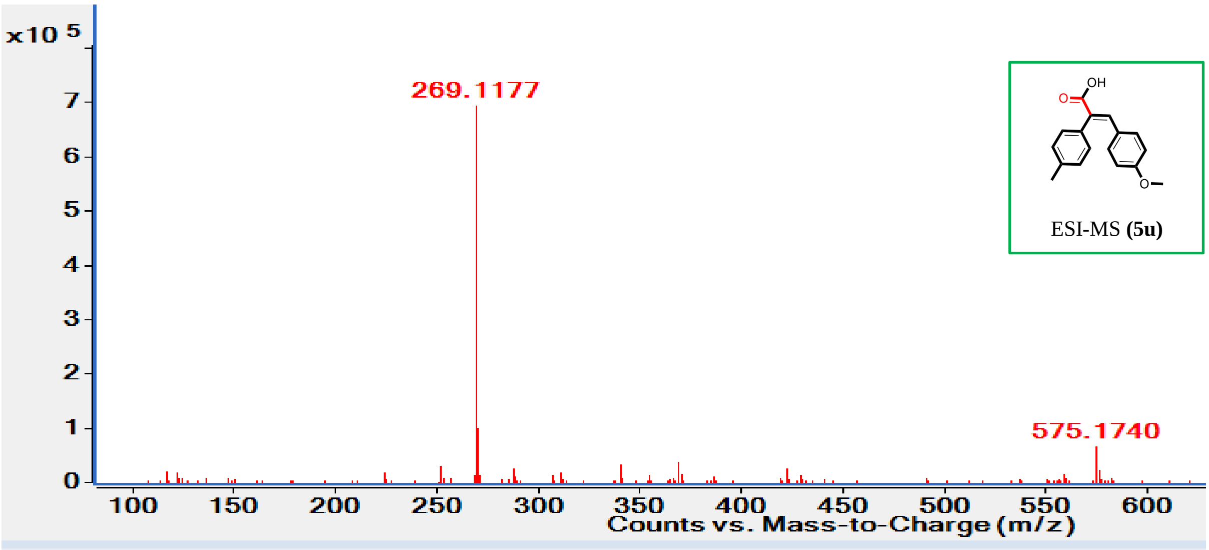

x10 5
269.1177
575.1740
OH
O
O
ESI-MS (5u)
Counts vs. Mass-to-Charge (m/z)

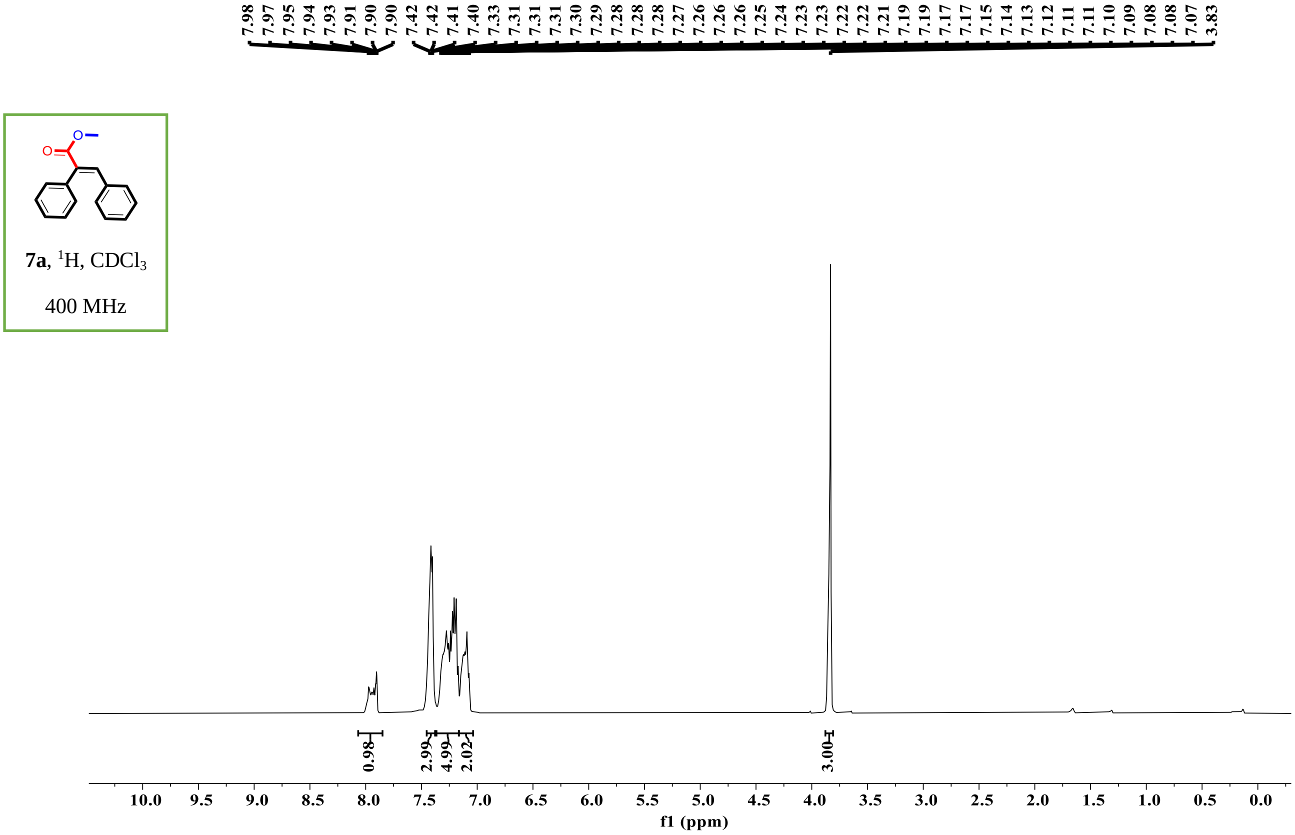
7.98
7.97
7.95
7.94
7.93
7.91
7.90
7.90
7.42
7.42
7.41
7.40
7.33
7.31
7.31
7.31
7.30
7.29
7.28
7.28
7.28
7.27
7.26
7.26
7.26
7.25
7.24
7.23
7.23
7.22
7.22
7.21
7.19
7.19
7.17
7.17
7.15
7.14
7.13
7.12
7.11
7.11
7.10
7.09
7.08
7.08
7.07
3.83
7a, ¹H, CDCl₃
400 MHz
0.98
2.99
4.99
2.02
3.00
10.0
9.5
9.0
8.5
8.0
7.5
7.0
6.5
6.0
5.5
5.0
4.5
4.0
3.5
3.0
2.5
2.0
1.5
1.0
0.5
0.0
f1 (ppm)

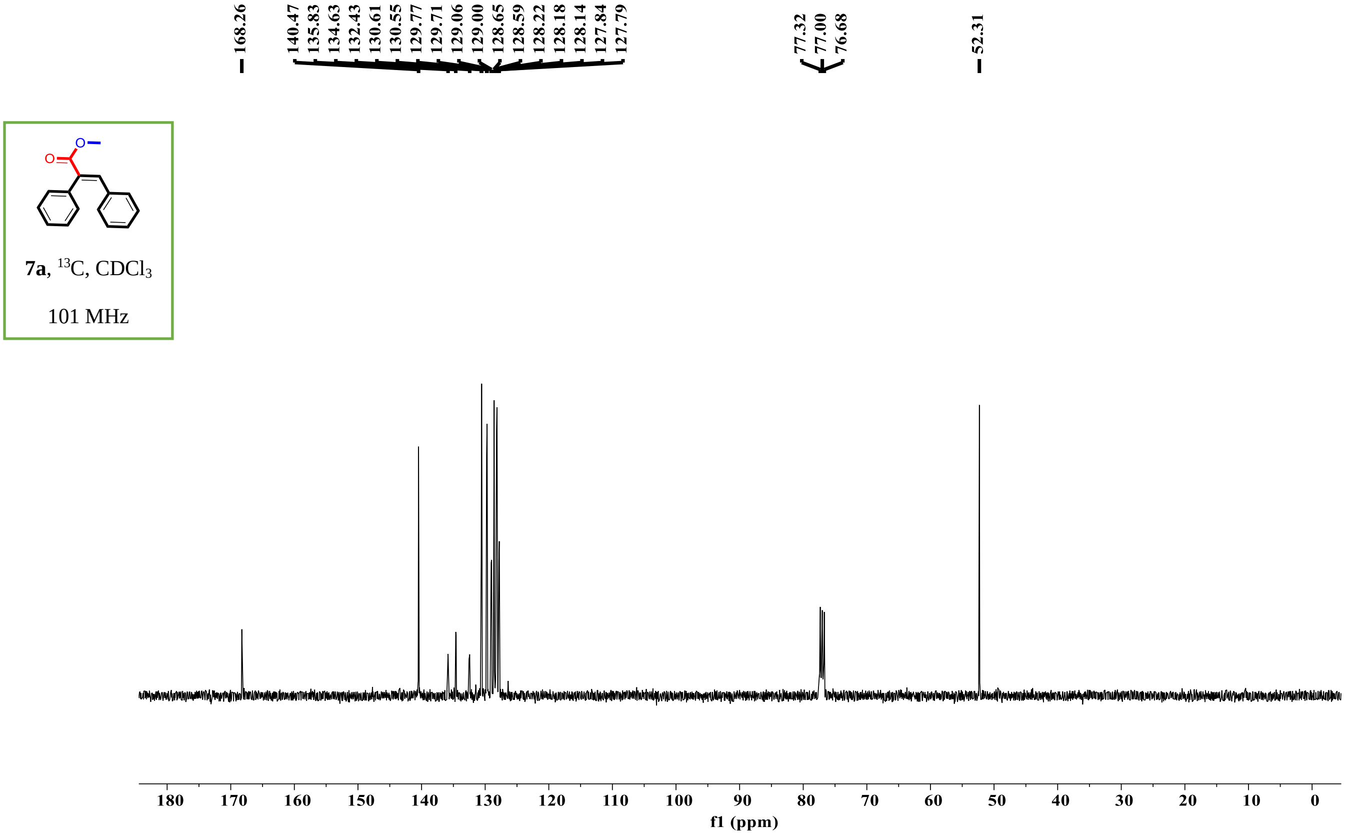
168.26
140.47
135.83
134.63
132.43
130.61
130.55
129.77
129.71
129.06
129.00
128.65
128.59
128.22
128.18
128.14
127.84
127.79
77.32
77.00
76.68
52.31
7a, ¹³C, CDCl₃
101 MHz
180
170
160
150
140
130
120
110
100
90
80
70
60
50
40
30
20
10
0
f1 (ppm)

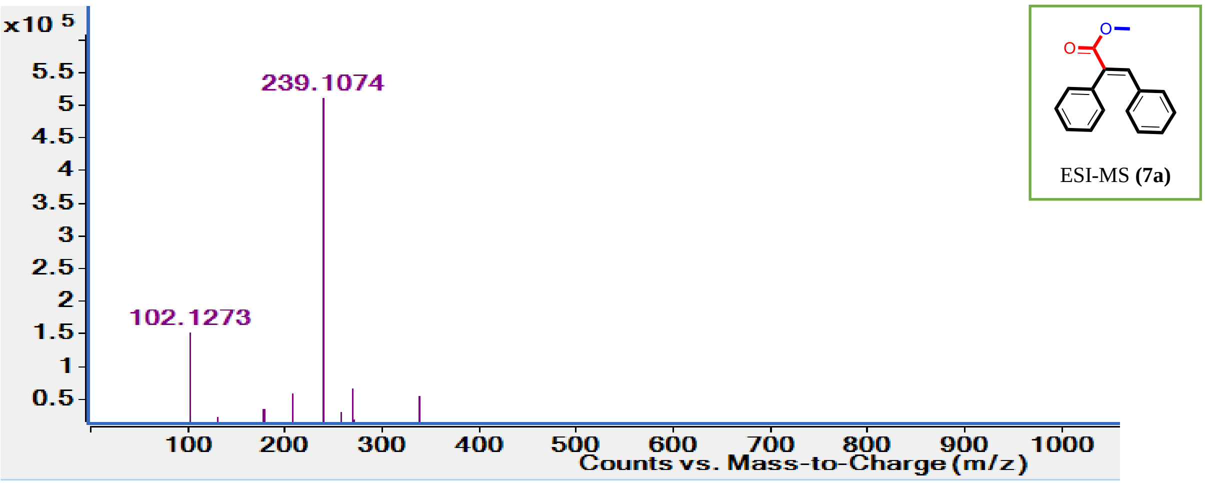
x10 5
239.1074
102.1273
Counts vs. Mass-to-Charge (m/z)
ESI-MS (7a)

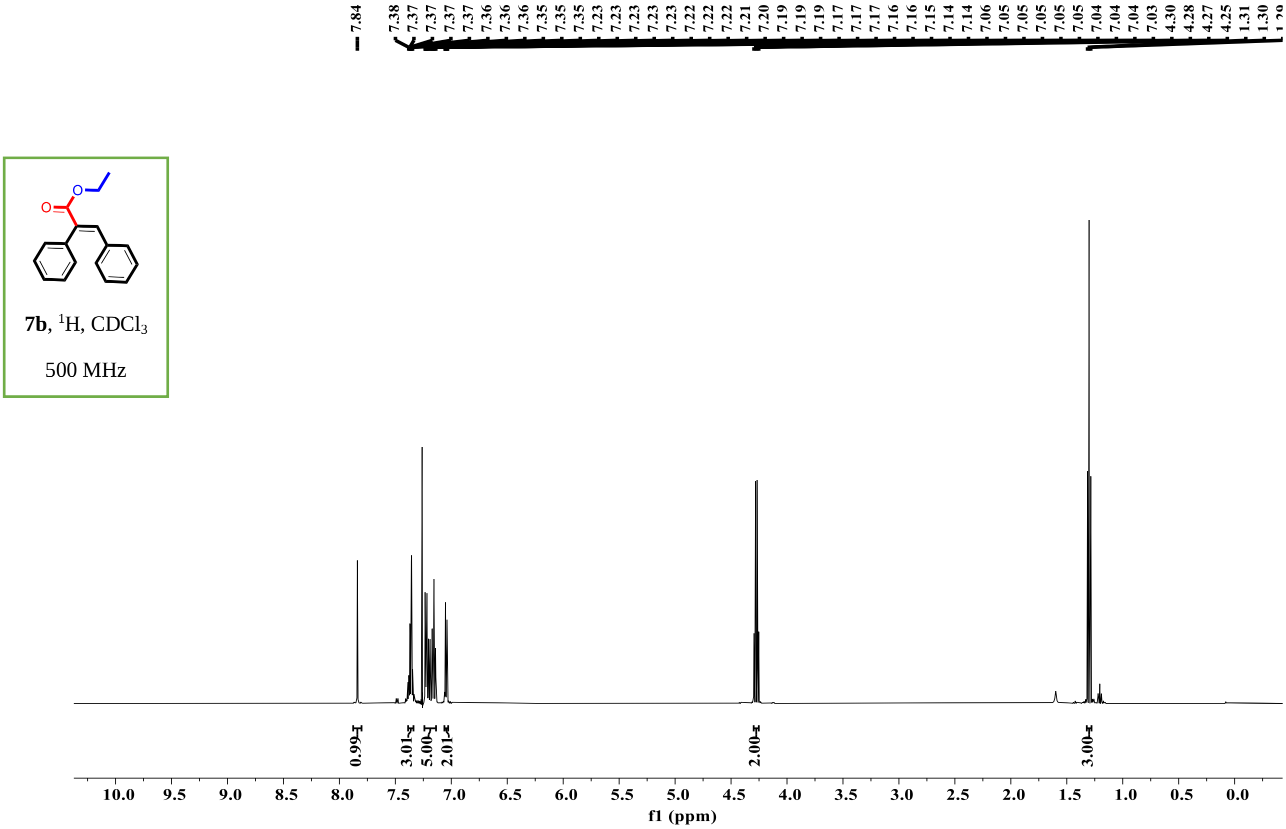
7b, 1H, CDCl3
500 MHz
f1 (ppm)

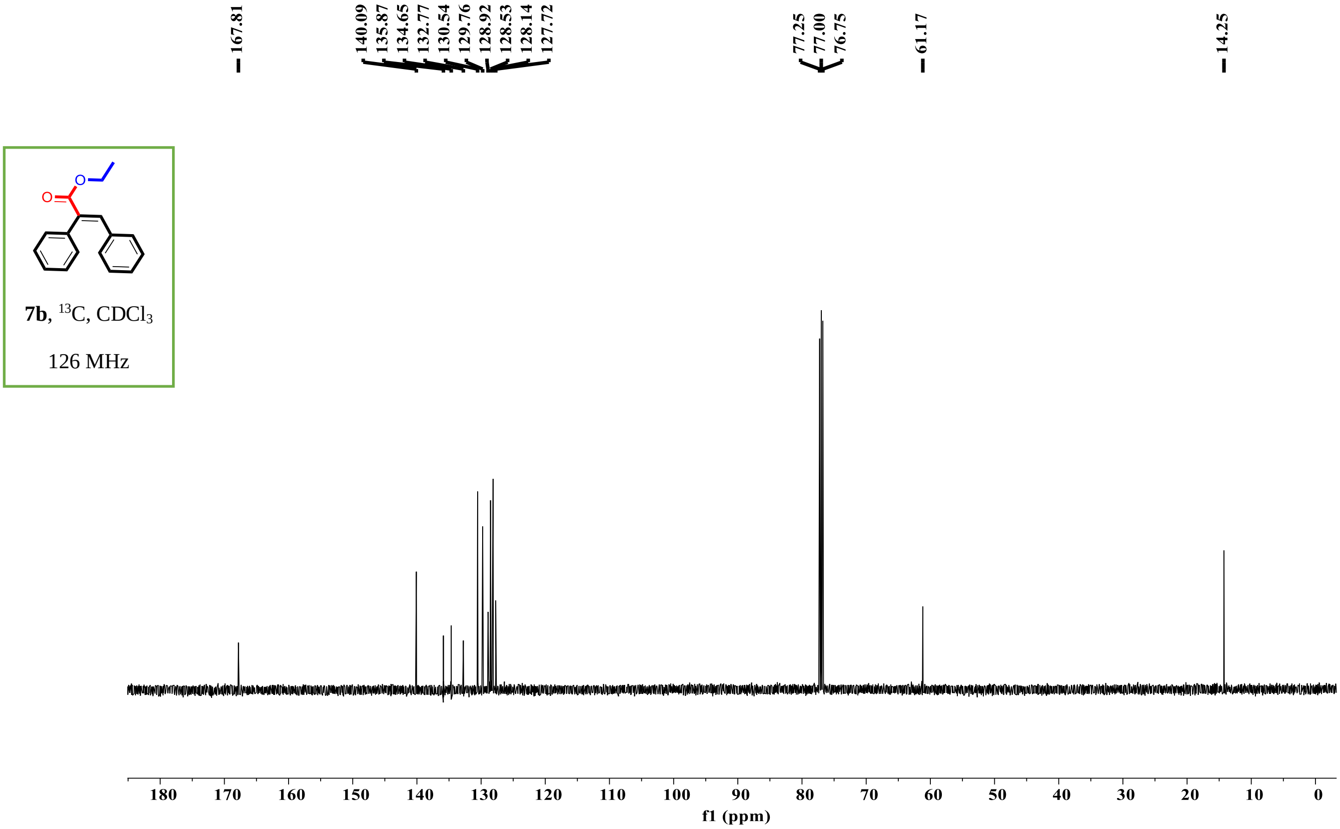
167.81
140.09
135.87
134.65
132.77
130.54
129.76
128.92
128.53
128.14
127.72
77.25
77.00
76.75
61.17
14.25
O
O
7b, 13C, CDCl3
126 MHz
180
170
160
150
140
130
120
110
100
90
80
70
60
50
40
30
20
10
0
f1 (ppm)

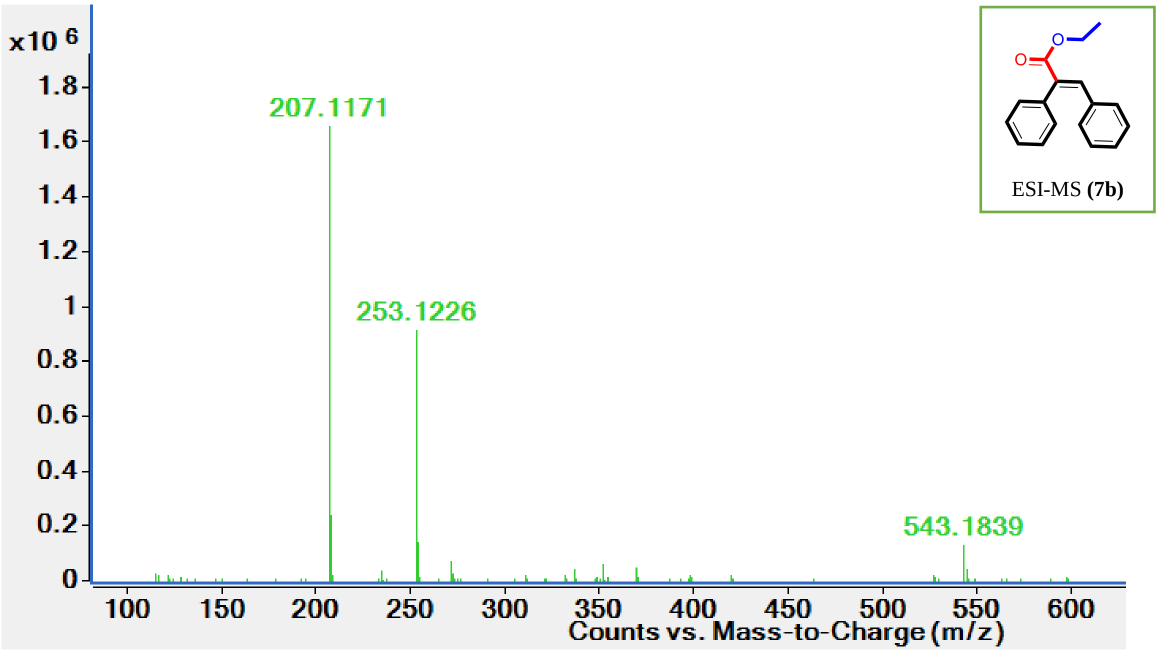
x10 6
1.8
1.6
1.4
1.2
1
0.8
0.6
0.4
0.2
0
207.1171
253.1226
543.1839
100
150
200
250
300
350
400
450
500
550
600
Counts vs. Mass-to-Charge (m/z)
O
O
ESI-MS (7b)

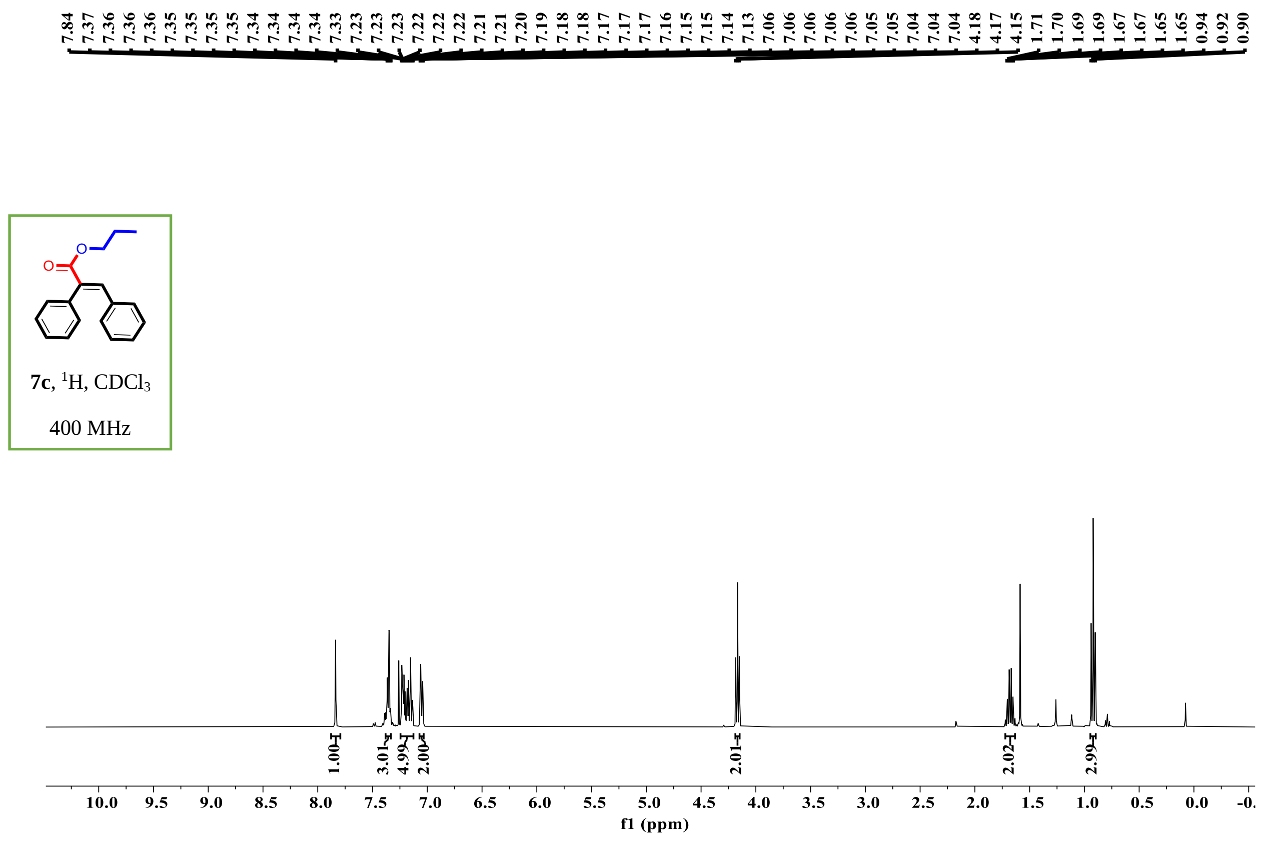
7.84 7.37 7.36 7.36 7.35 7.35 7.35 7.34 7.34 7.34 7.34 7.33 7.23 7.23 7.23 7.22 7.22 7.22 7.21 7.21 7.20 7.19 7.18 7.18 7.17 7.17 7.17 7.16 7.15 7.15 7.14 7.13 7.06 7.06 7.06 7.06 7.05 7.05 7.04 7.04 7.04 4.18 4.17 4.15 1.71 1.70 1.69 1.69 1.67 1.67 1.65 1.65 0.94 0.92 0.90
O
O
7c, 1H, CDCl3
400 MHz
1.00 3.01 4.99 2.00 2.01 2.02 2.99
f1 (ppm)

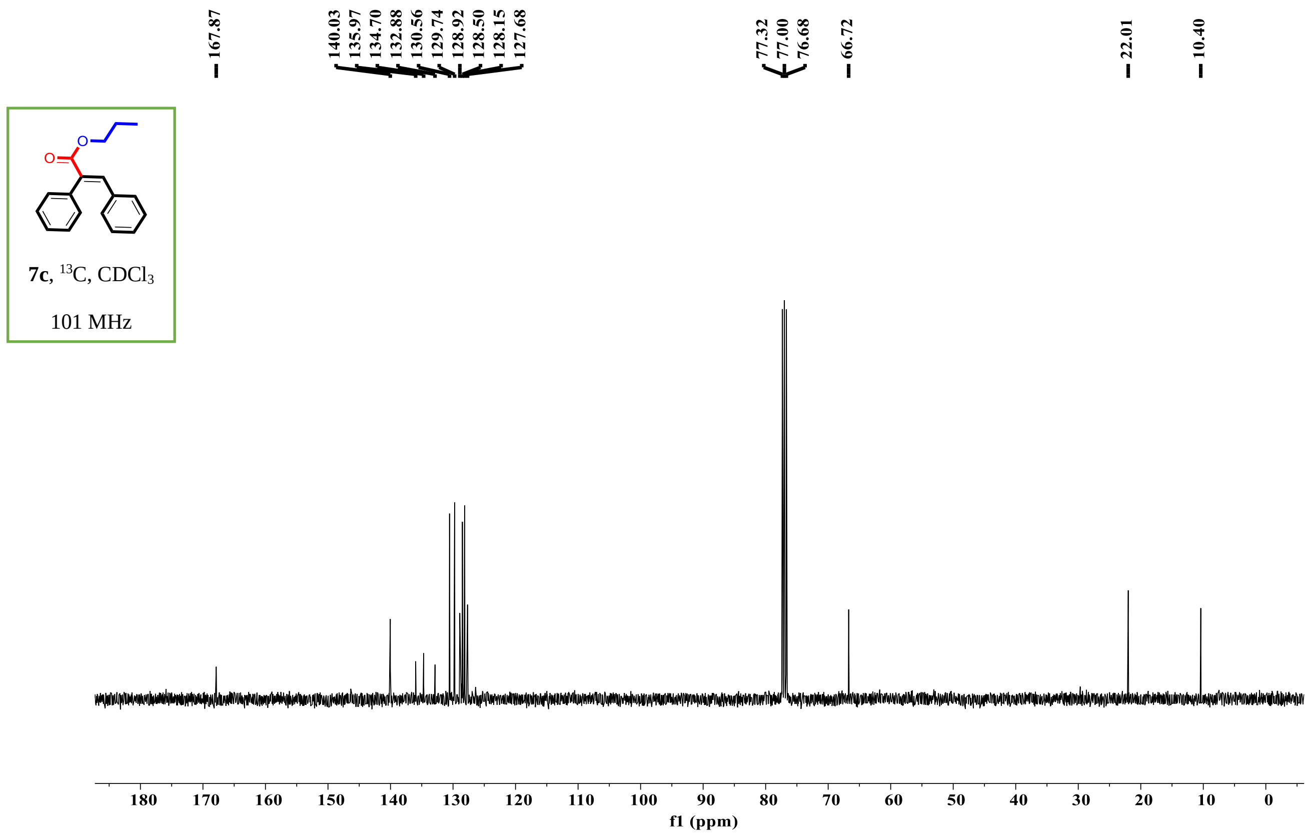
167.87 140.03 135.97 134.70 132.88 130.56 129.74 128.92 128.50 128.15 127.68 77.32 77.00 76.68 66.72 22.01 10.40
O
O
7c, 13C, CDCl3
101 MHz
f1 (ppm)

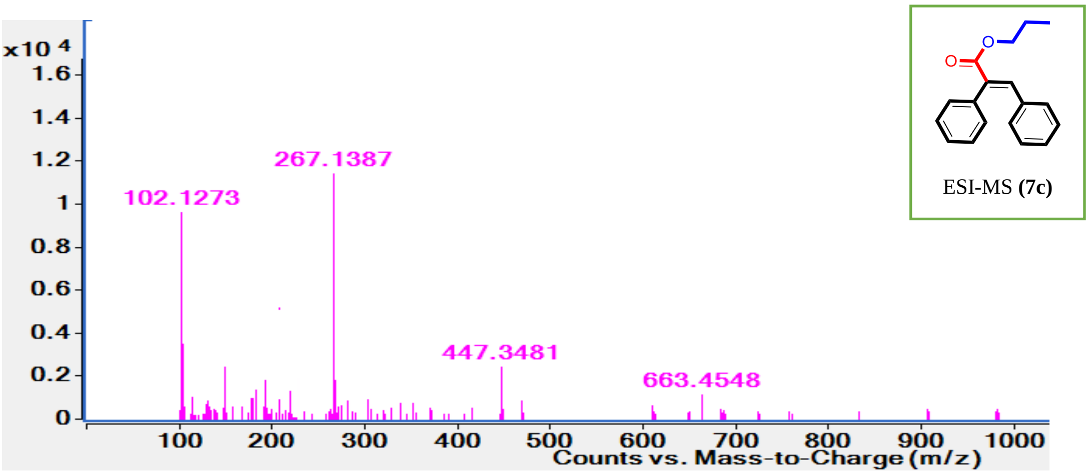

x10 4
1.6
1.4
1.2
1
0.8
0.6
0.4
0.2
0
102.1273
267.1387
447.3481
663.4548
100
200
300
400
500
600
700
800
900
1000
Counts vs. Mass-to-Charge (m/z)
ESI-MS (7c)

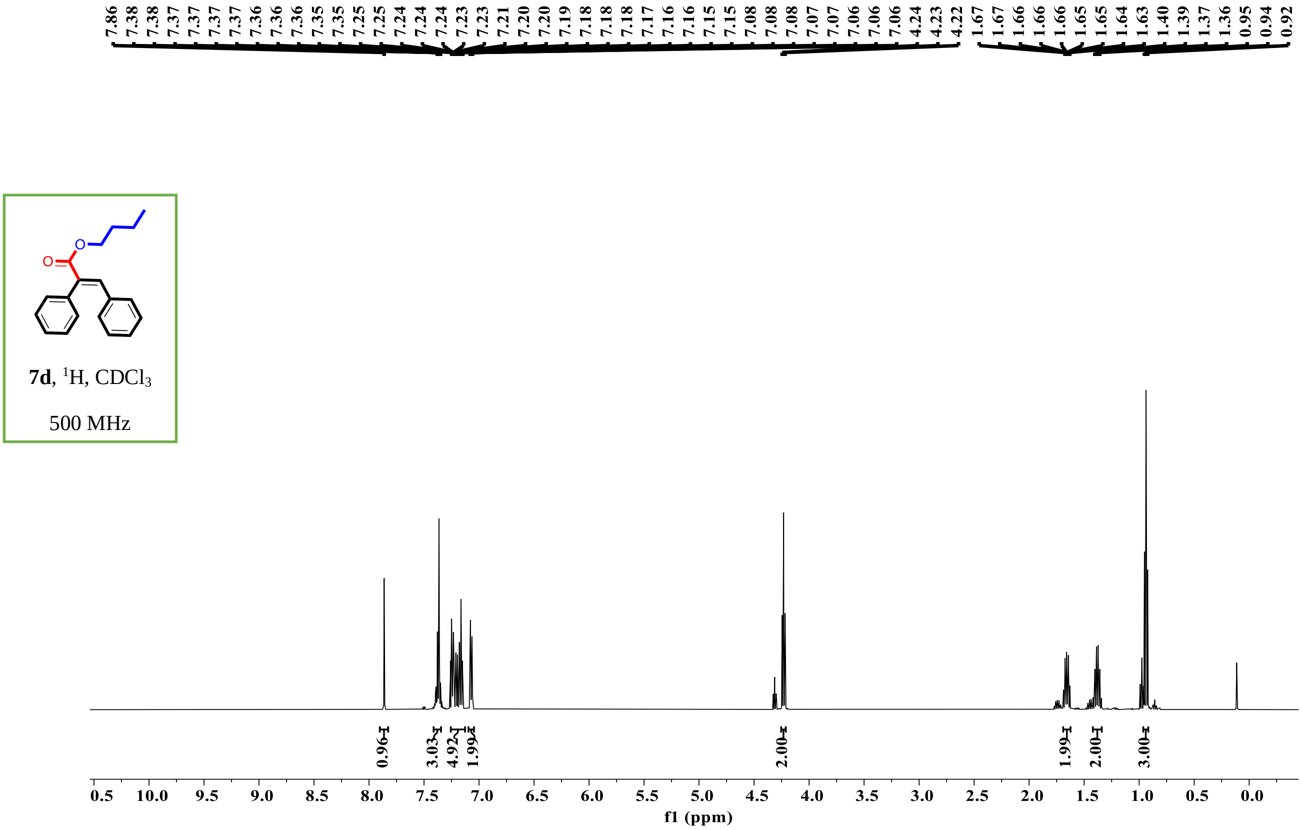

7.86 7.38 7.38 7.37 7.37 7.37 7.36 7.36 7.36 7.35 7.35 7.25 7.24 7.24 7.24 7.23 7.23 7.21 7.20 7.20 7.19 7.18 7.18 7.18 7.17 7.16 7.16 7.15 7.15 7.08 7.08 7.07 7.07 7.06 7.06 7.06 4.24 4.23 4.22 1.67 1.67 1.66 1.66 1.66 1.65 1.65 1.64 1.63 1.40 1.39 1.37 1.36 0.95 0.94 0.92
7d, ¹H, CDCl₃
500 MHz
0.96
3.03
4.92
1.99
2.00
1.99
2.00
3.00
f1 (ppm)

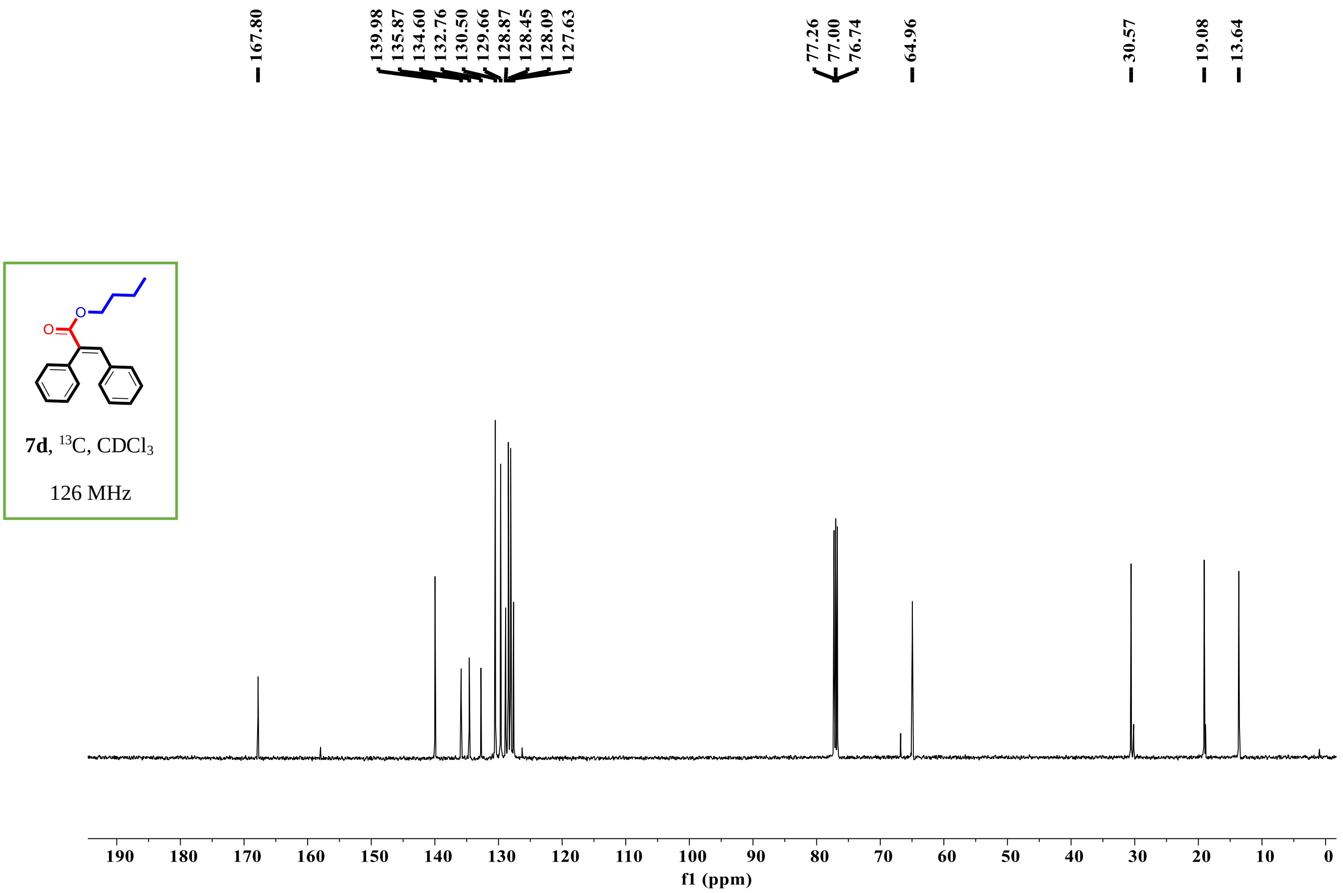
167.80
139.98
135.87
134.60
132.76
130.50
129.66
128.87
128.45
128.09
127.63
77.26
77.00
76.74
64.96
30.57
19.08
13.64
7d, ^{13}C, $CDCl_3$
126 MHz
190 180 170 160 150 140 130 120 110 100 90 80 70 60 50 40 30 20 10 0
f1 (ppm)

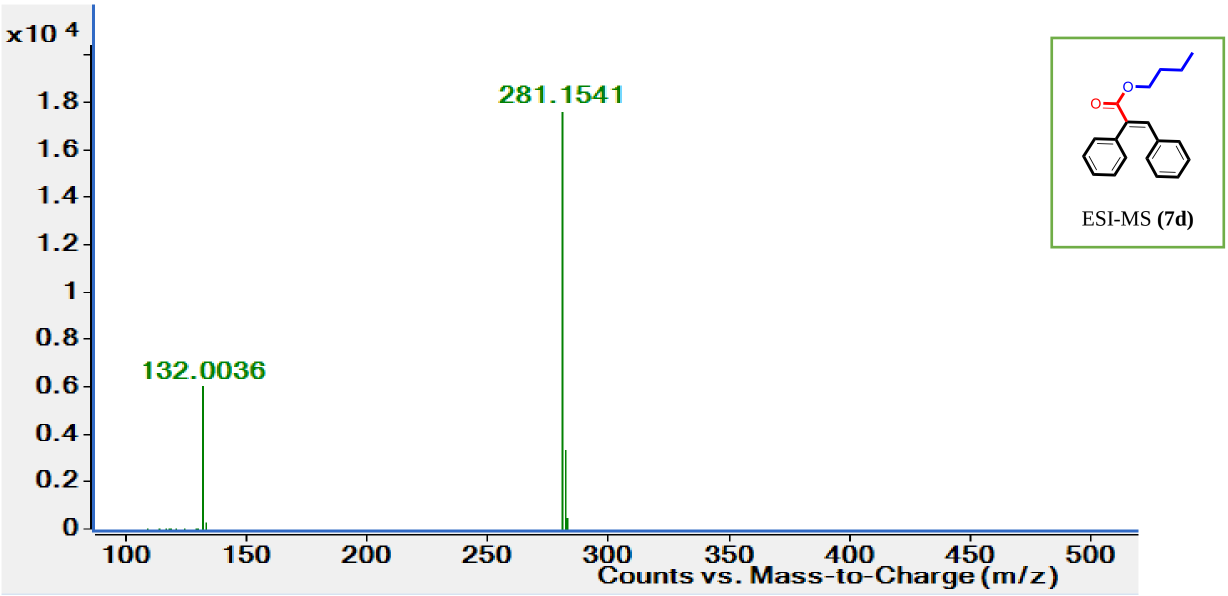
x10 4
1.8
1.6
1.4
1.2
1
0.8
0.6
0.4
0.2
0
281.1541
132.0036
100 150 200 250 300 350 400 450 500
Counts vs. Mass-to-Charge (m/z)
ESI-MS (7d)

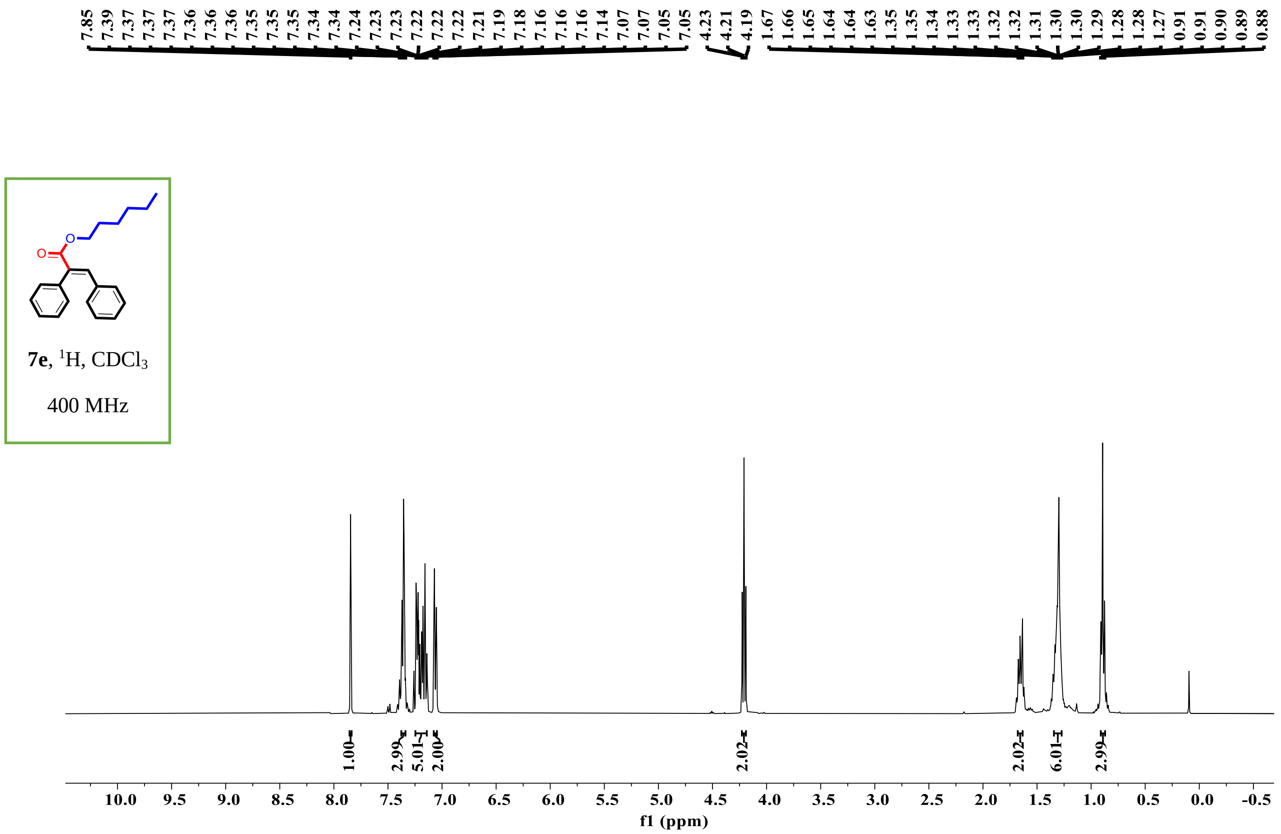
7.85
7.39
7.37
7.37
7.37
7.36
7.36
7.36
7.35
7.35
7.35
7.34
7.34
7.24
7.23
7.23
7.22
7.22
7.22
7.21
7.19
7.18
7.16
7.16
7.16
7.14
7.07
7.07
7.05
7.05
4.23
4.21
4.19
1.67
1.66
1.65
1.64
1.64
1.63
1.35
1.35
1.34
1.33
1.33
1.32
1.32
1.31
1.30
1.30
1.29
1.28
1.28
1.27
0.91
0.91
0.90
0.89
0.88
7e, ¹H, CDCl₃
400 MHz
1.00
2.99
5.01
2.00
2.02
2.02
6.01
2.99
10.0 9.5 9.0 8.5 8.0 7.5 7.0 6.5 6.0 5.5 5.0 4.5 4.0 3.5 3.0 2.5 2.0 1.5 1.0 0.5 0.0 -0.5
f1 (ppm)

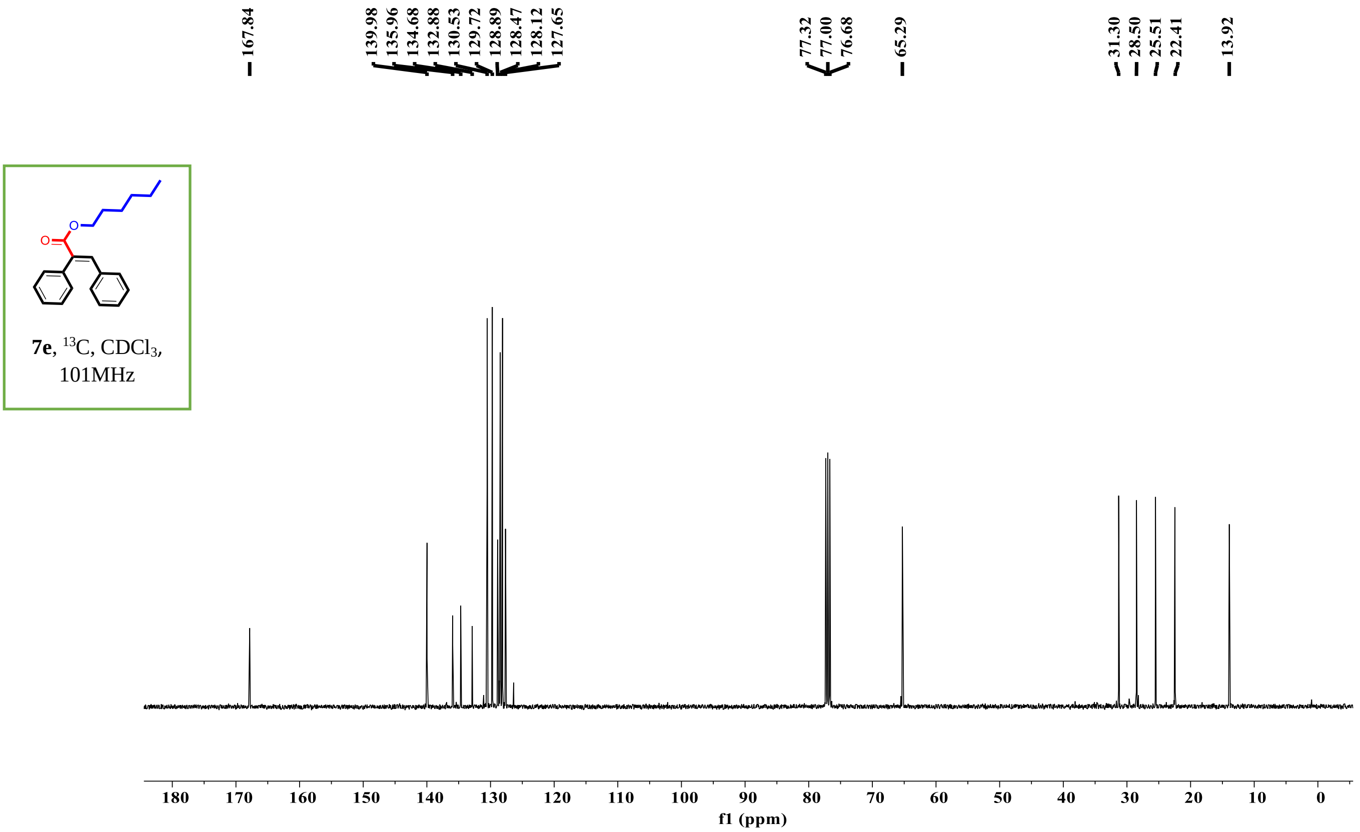
167.84
139.98
135.96
134.68
132.88
130.53
129.72
128.89
128.47
128.12
127.65
77.32
77.00
76.68
65.29
31.30
28.50
25.51
22.41
13.92
7e, ¹³C, CDCl₃,
101MHz
180 170 160 150 140 130 120 110 100 90 80 70 60 50 40 30 20 10 0
f1 (ppm)

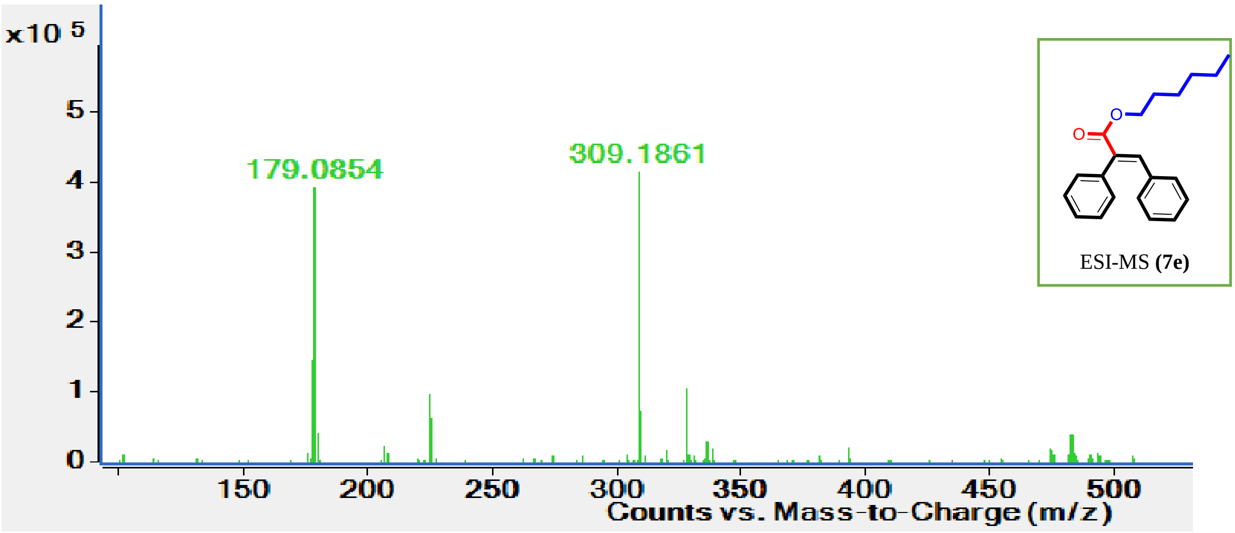
x10 5
179.0854
309.1861
150
200
250
300
350
400
450
500
Counts vs. Mass-to-Charge (m/z)
ESI-MS (7e)

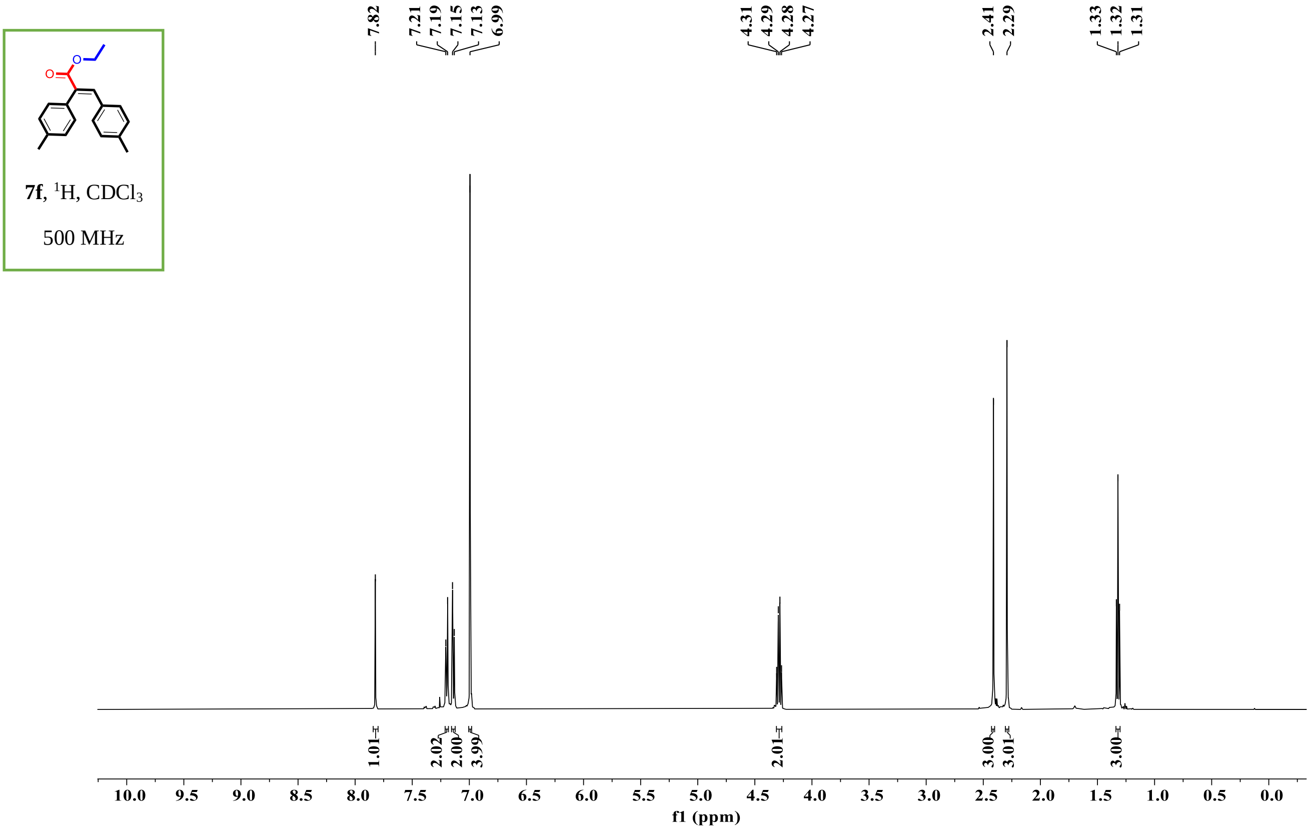
7f, ¹H, CDCl₃
500 MHz
7.82
7.21
7.19
7.15
7.13
6.99
4.31
4.29
4.28
4.27
2.41
2.29
1.33
1.32
1.31
1.01
2.02
2.00
3.99
2.01
3.00
3.01
3.00
f1 (ppm)

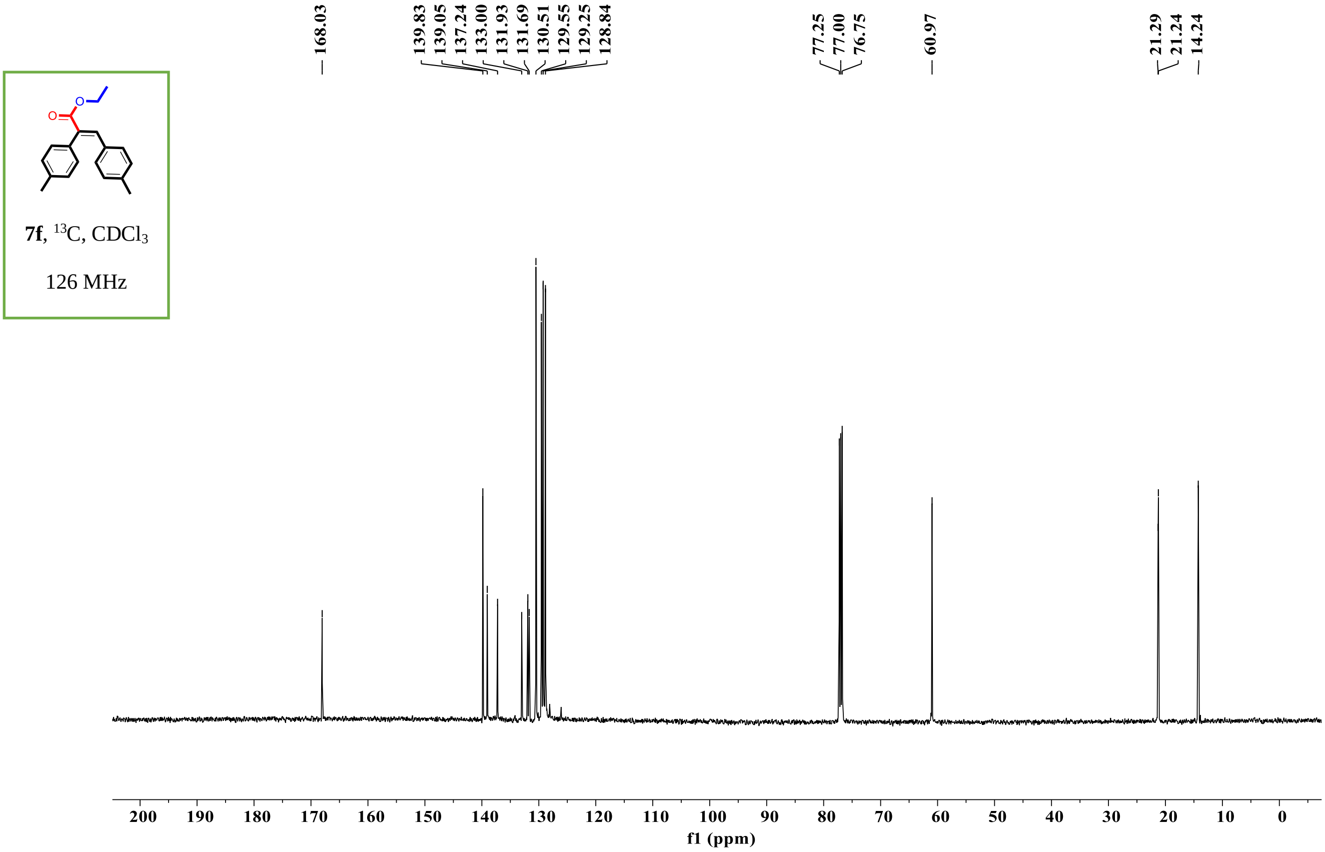
168.03
139.83
139.05
137.24
133.00
131.93
131.69
130.51
129.55
129.25
128.84
77.25
77.00
76.75
60.97
21.29
21.24
14.24
O
O
7f, ^{13}C, CDCl$_3$
126 MHz
200 190 180 170 160 150 140 130 120 110 100 90 80 70 60 50 40 30 20 10 0
f1 (ppm)

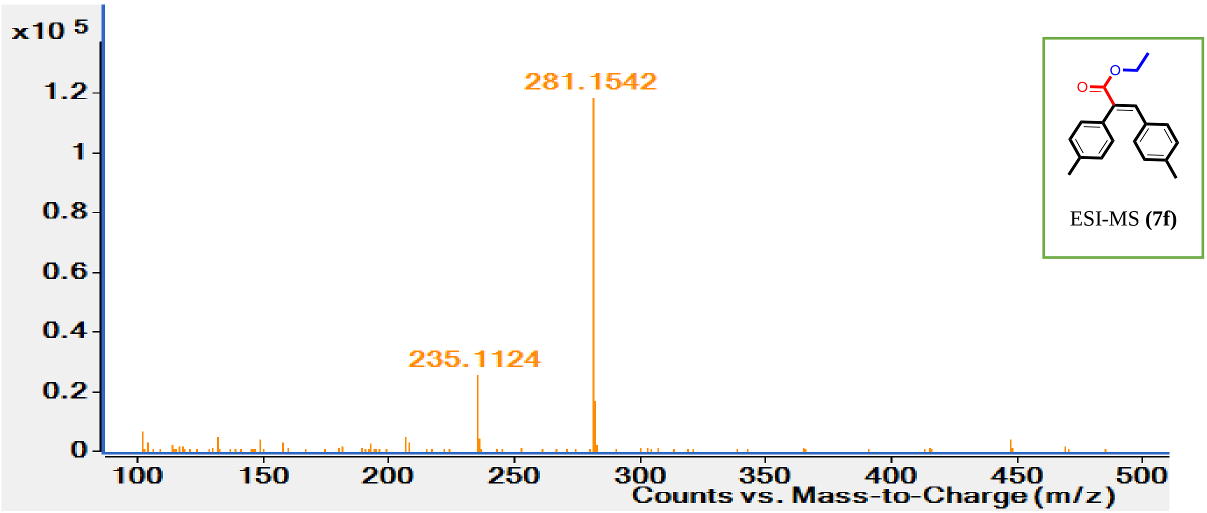
x10 5
1.2
1
0.8
0.6
0.4
0.2
0
281.1542
235.1124
100 150 200 250 300 350 400 450 500
Counts vs. Mass-to-Charge (m/z)
O
O
ESI-MS (7f)

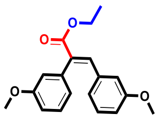

7g, ¹H, CDCl$_3$

400 MHz

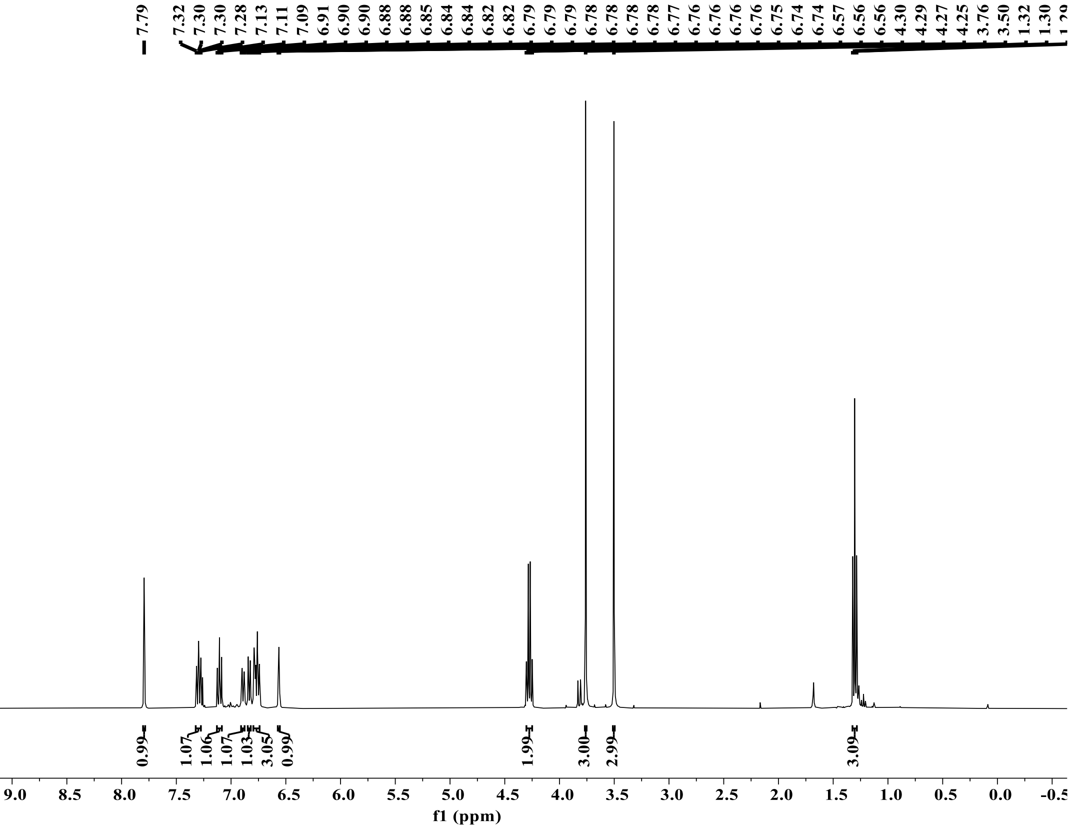

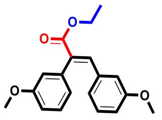

7g, ¹³C, CDCl$_3$

101 MHz

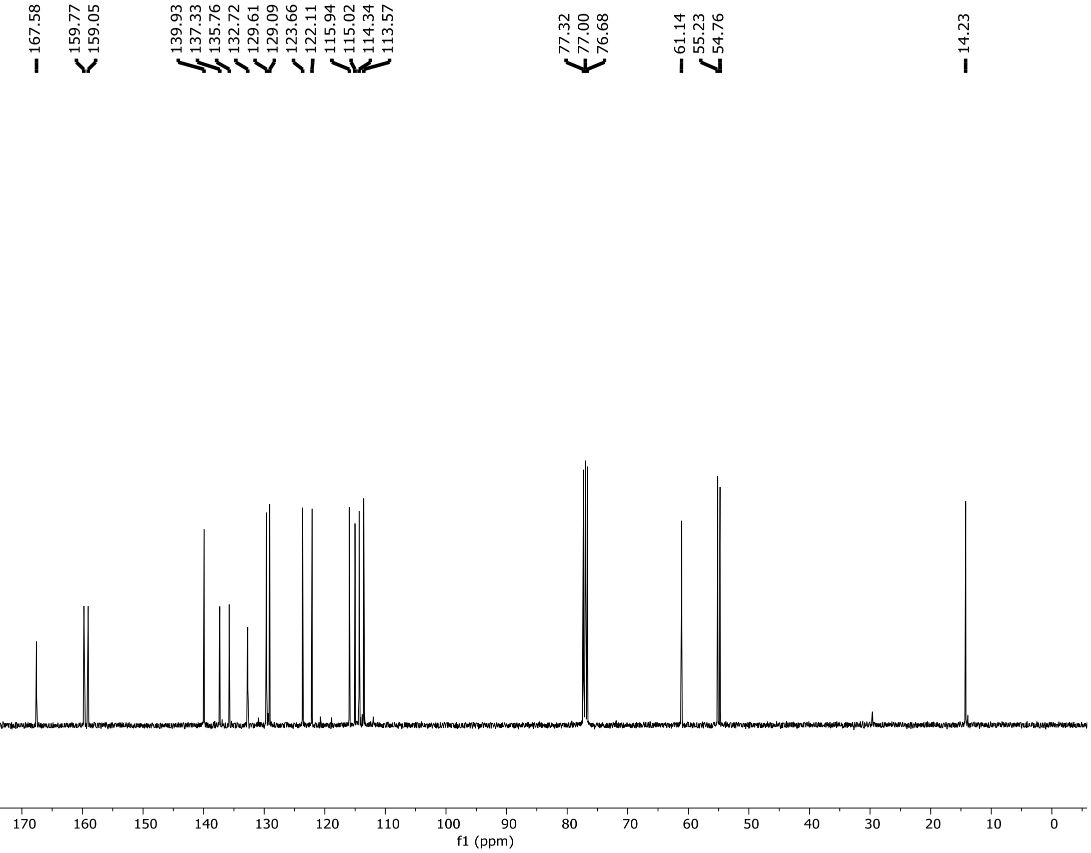

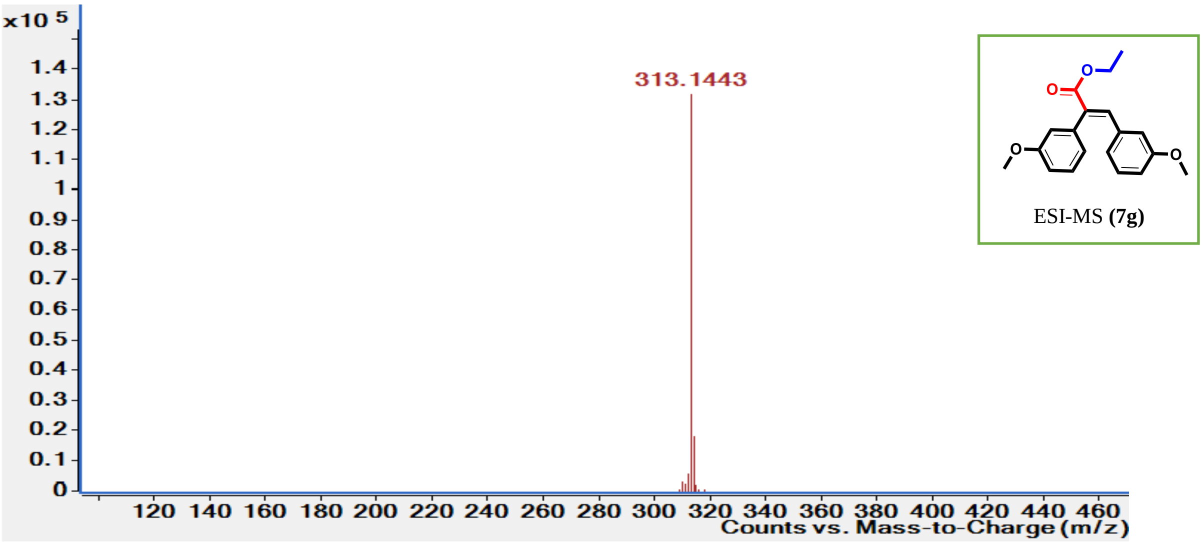
x10 5
313.1443
Counts vs. Mass-to-Charge (m/z)
ESI-MS (7g)

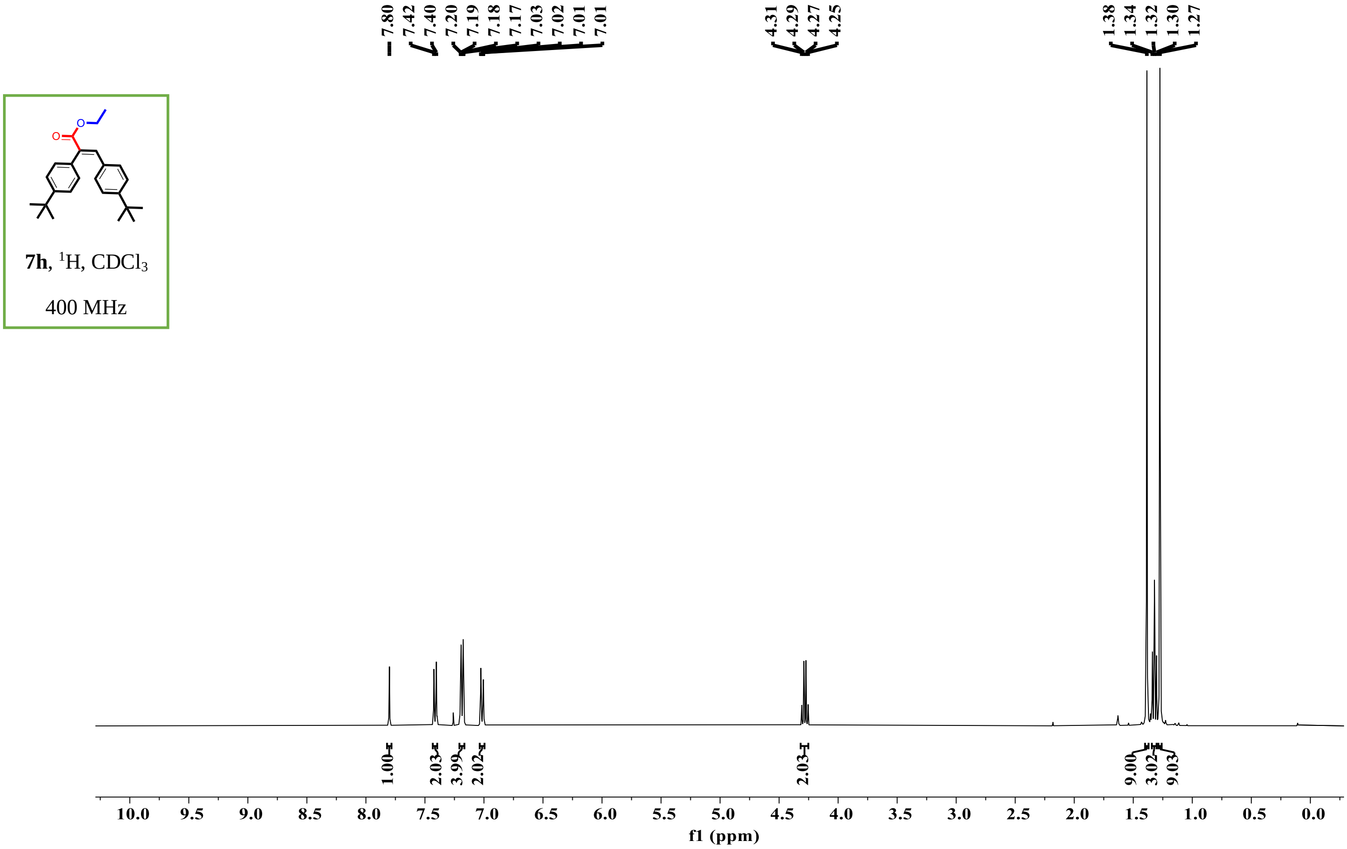
7h, 1H, CDCl3
400 MHz
f1 (ppm)

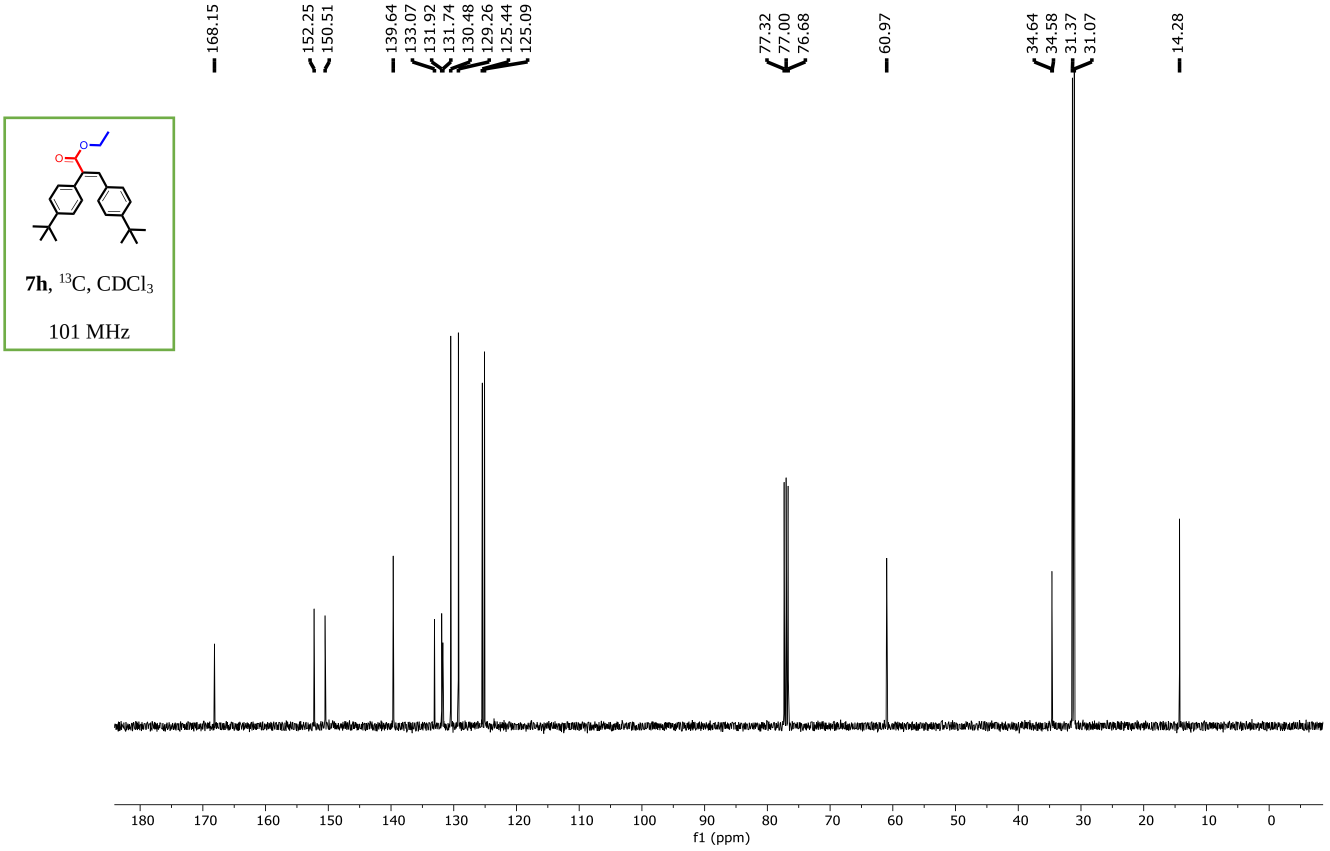

168.15
152.25
150.51
139.64
133.07
131.92
131.74
130.48
129.26
125.44
125.09
77.32
77.00
76.68
60.97
34.64
34.58
31.37
31.07
14.28
O
O
7h, ^{13}C, CDCl$_3$
101 MHz
180
170
160
150
140
130
120
110
100
90
80
70
60
50
40
30
20
10
0
f1 (ppm)

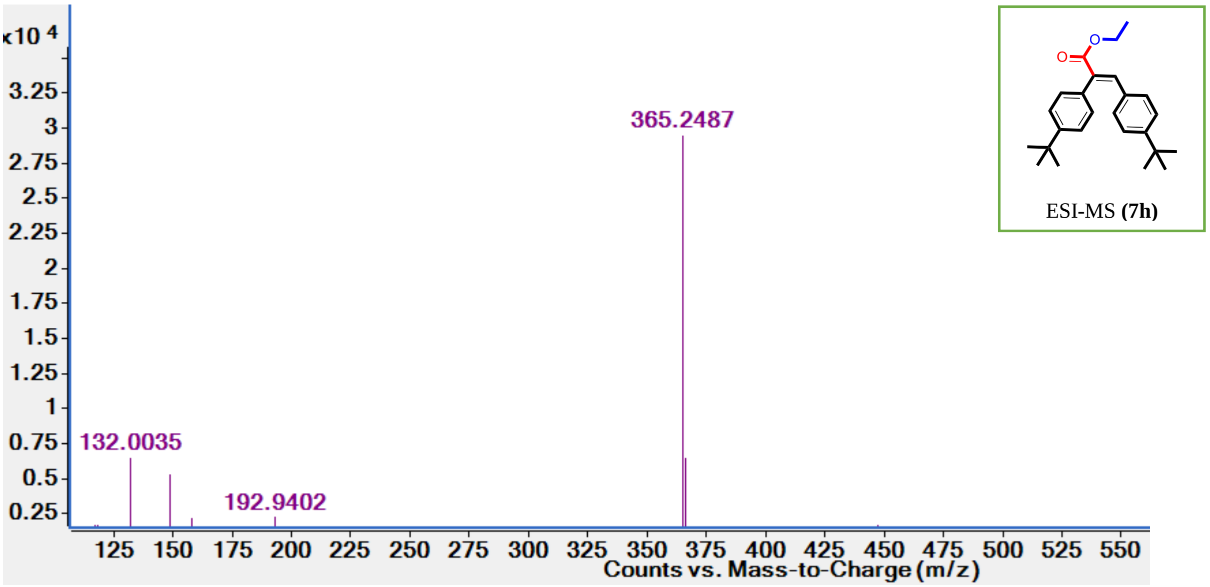

x10 4
3.25
3
2.75
2.5
2.25
2
1.75
1.5
1.25
1
0.75
0.5
0.25
365.2487
132.0035
192.9402
125 150 175 200 225 250 275 300 325 350 375 400 425 450 475 500 525 550
Counts vs. Mass-to-Charge (m/z)
O
O
ESI-MS (7h)

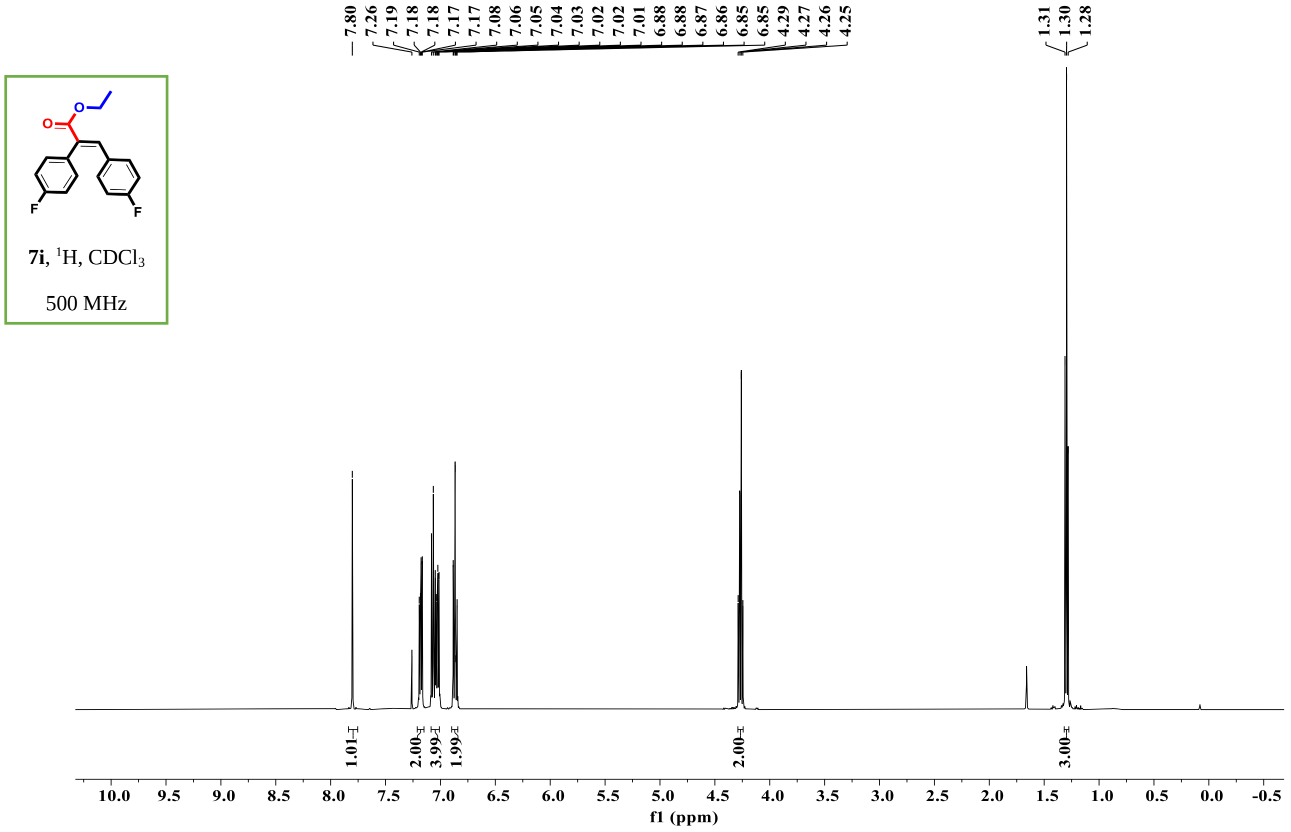
7.80
7.26
7.19
7.18
7.18
7.17
7.17
7.08
7.06
7.05
7.04
7.03
7.02
7.02
7.01
6.88
6.88
6.87
6.86
6.85
6.85
4.29
4.27
4.26
4.25
1.31
1.30
1.28
O
O
F
F
7i, 1H, CDCl3
500 MHz
1.01
2.00
3.99
1.99
2.00
3.00
10.0 9.5 9.0 8.5 8.0 7.5 7.0 6.5 6.0 5.5 5.0 4.5 4.0 3.5 3.0 2.5 2.0 1.5 1.0 0.5 0.0 -0.5
f1 (ppm)

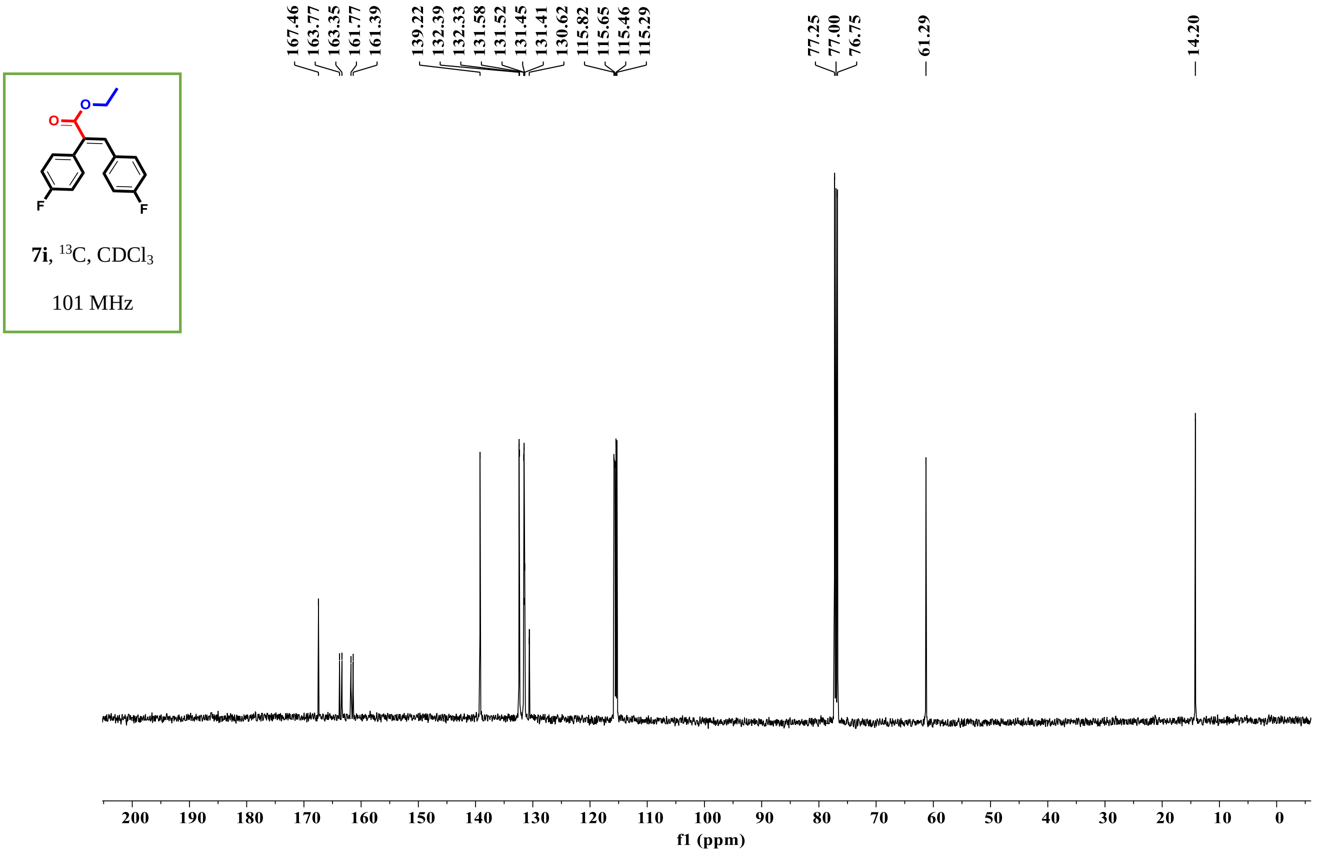
167.46
163.77
163.35
161.77
161.39
139.22
132.39
132.33
131.58
131.52
131.45
131.41
130.62
115.82
115.65
115.46
115.29
77.25
77.00
76.75
61.29
14.20
O
O
F
F
7i, 13C, CDCl3
101 MHz
200 190 180 170 160 150 140 130 120 110 100 90 80 70 60 50 40 30 20 10 0
f1 (ppm)

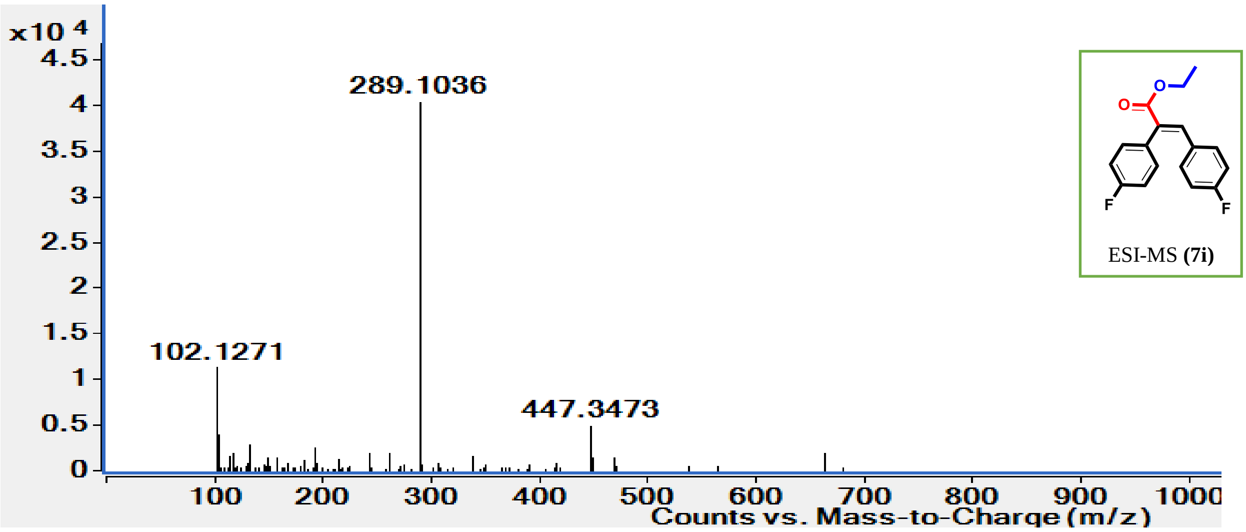

x10 4
4.5
4
3.5
3
2.5
2
1.5
1
0.5
0
289.1036
102.1271
447.3473
100
200
300
400
500
600
700
800
900
1000
Counts vs. Mass-to-Charge (m/z)
O
O
F
F
ESI-MS (7i)

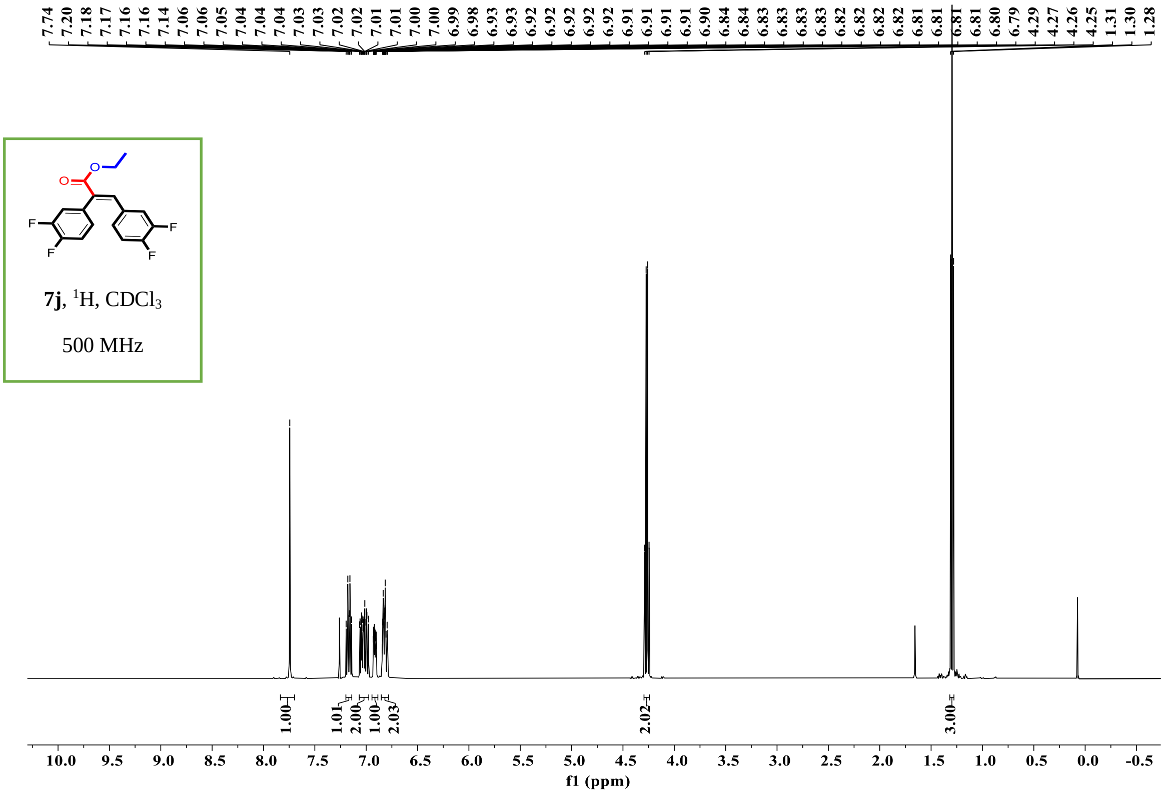

7.74 7.20 7.18 7.17 7.16 7.16 7.14 7.06 7.06 7.05 7.04 7.04 7.04 7.03 7.03 7.02 7.02 7.01 7.01 7.00 6.99 6.98 6.93 6.93 6.92 6.92 6.92 6.92 6.92 6.91 6.91 6.91 6.91 6.90 6.84 6.84 6.83 6.83 6.83 6.83 6.82 6.82 6.82 6.82 6.81 6.81 6.81 6.81 6.80 6.79 4.29 4.27 4.26 4.25 1.31 1.30 1.28
O
O
F
F
F
F
7j, 1H, CDCl3
500 MHz
1.00
1.01
2.00
1.00
2.03
2.02
3.00
10.0 9.5 9.0 8.5 8.0 7.5 7.0 6.5 6.0 5.5 5.0 4.5 4.0 3.5 3.0 2.5 2.0 1.5 1.0 0.5 0.0 -0.5
f1 (ppm)

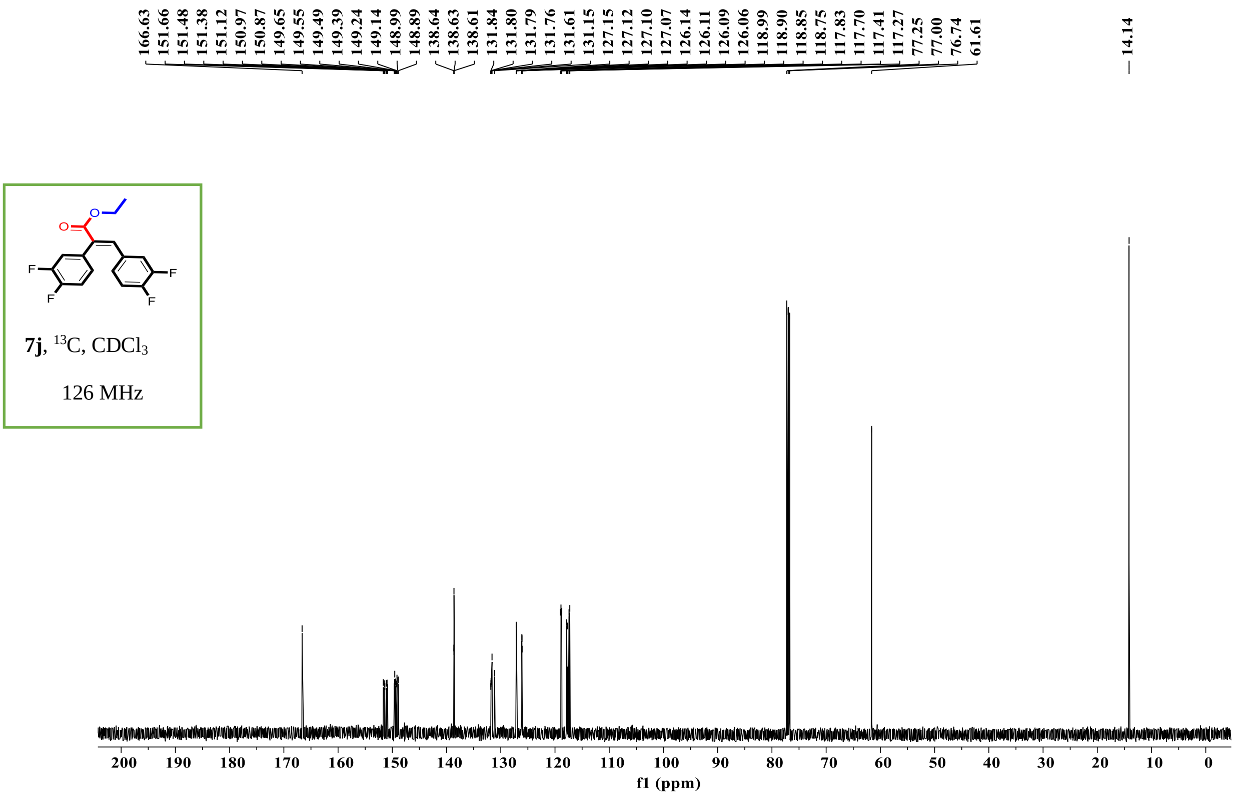
166.63
151.66
151.48
151.38
151.12
150.97
150.87
149.65
149.55
149.49
149.39
149.24
149.14
148.99
148.89
138.64
138.63
138.61
131.84
131.80
131.79
131.76
131.61
131.15
127.15
127.12
127.10
127.07
126.14
126.11
126.09
126.06
118.99
118.90
118.85
118.75
117.83
117.70
117.41
117.27
77.25
77.00
76.74
61.61
14.14
O
O
F
F
F
F
7j, ¹³C, CDCl₃
126 MHz
200 190 180 170 160 150 140 130 120 110 100 90 80 70 60 50 40 30 20 10 0
f1 (ppm)

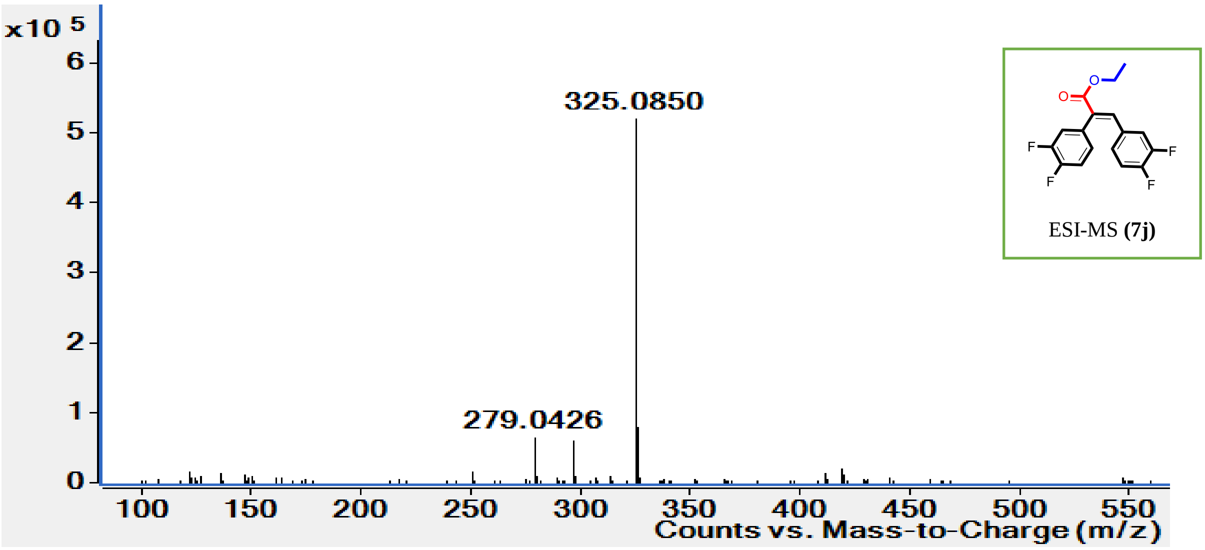
x10 5
6
5
4
3
2
1
0
325.0850
279.0426
100 150 200 250 300 350 400 450 500 550
Counts vs. Mass-to-Charge (m/z)
O
O
F
F
F
F
ESI-MS (7j)

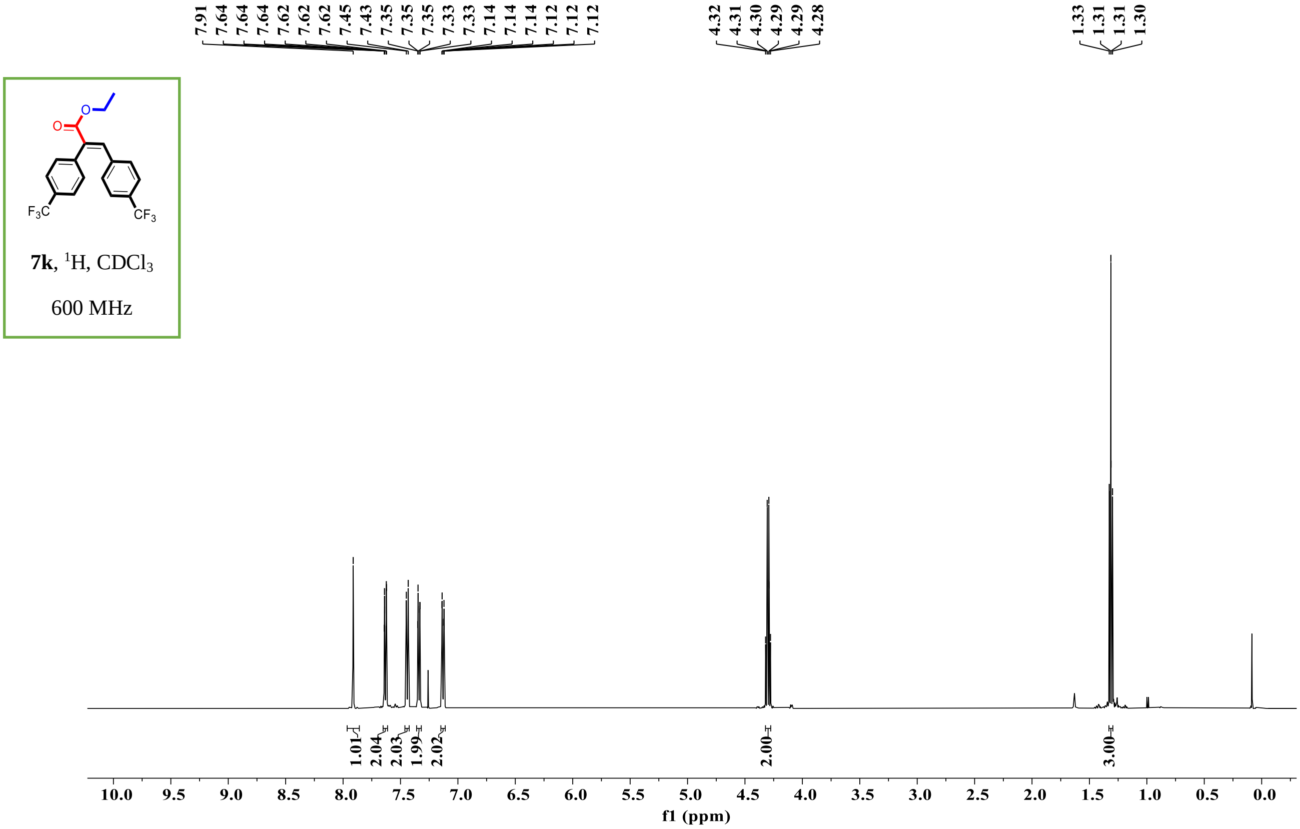
7.91
7.64
7.64
7.64
7.62
7.62
7.62
7.45
7.43
7.35
7.35
7.35
7.33
7.33
7.14
7.14
7.14
7.12
7.12
7.12
4.32
4.31
4.30
4.29
4.29
4.28
1.33
1.31
1.31
1.30
O
O
F_3C
CF_3
7k, ^{1}H, $CDCl_3$
600 MHz
1.01
2.04
2.03
1.99
2.02
2.00
3.00
10.0 9.5 9.0 8.5 8.0 7.5 7.0 6.5 6.0 5.5 5.0 4.5 4.0 3.5 3.0 2.5 2.0 1.5 1.0 0.5 0.0
f1 (ppm)

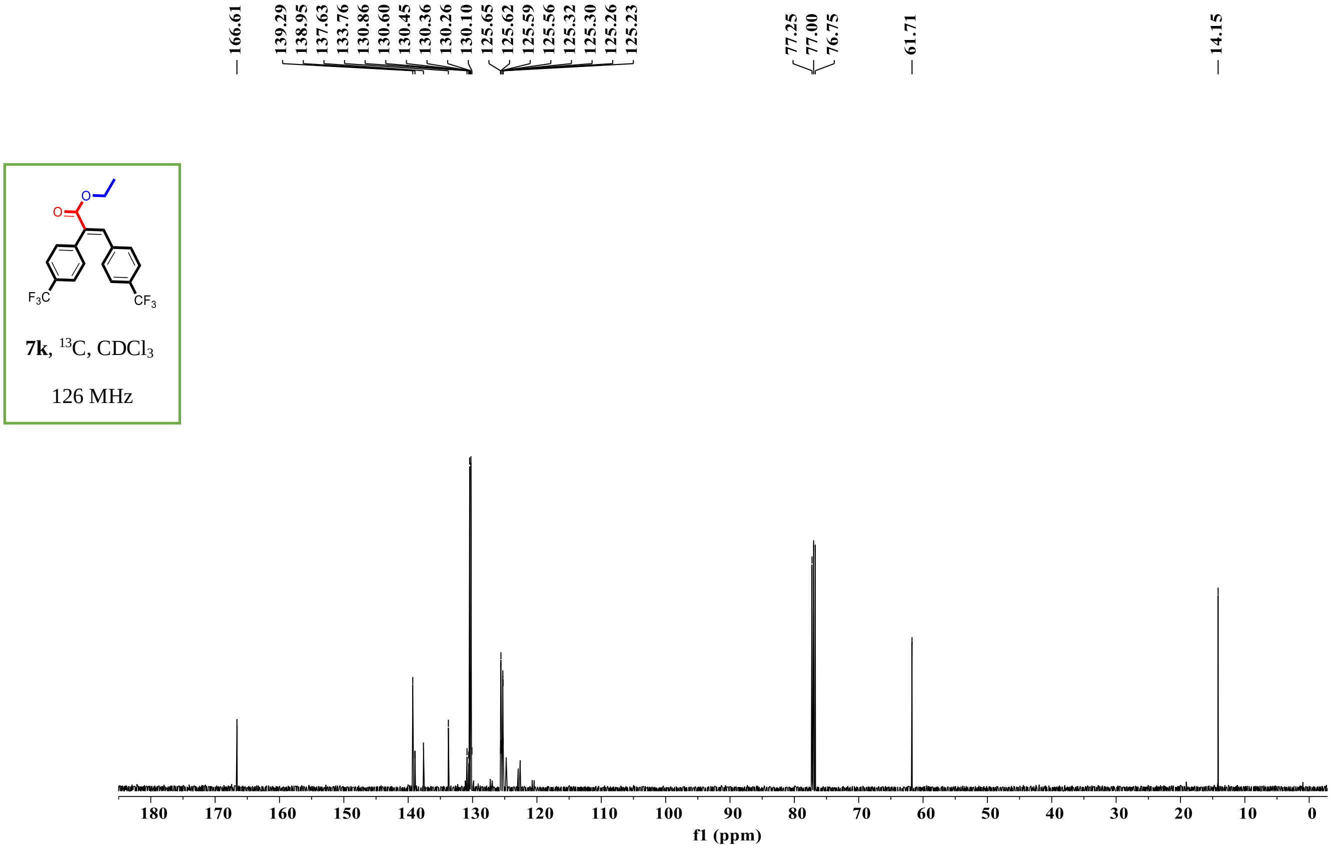
166.61
139.29
138.95
137.63
133.76
130.86
130.60
130.45
130.36
130.26
130.10
125.65
125.62
125.59
125.56
125.32
125.30
125.26
125.23
77.25
77.00
76.75
61.71
14.15
O
O
F_3C
CF_3
7k, ^{13}C, $CDCl_3$
126 MHz
180 170 160 150 140 130 120 110 100 90 80 70 60 50 40 30 20 10 0
f1 (ppm)

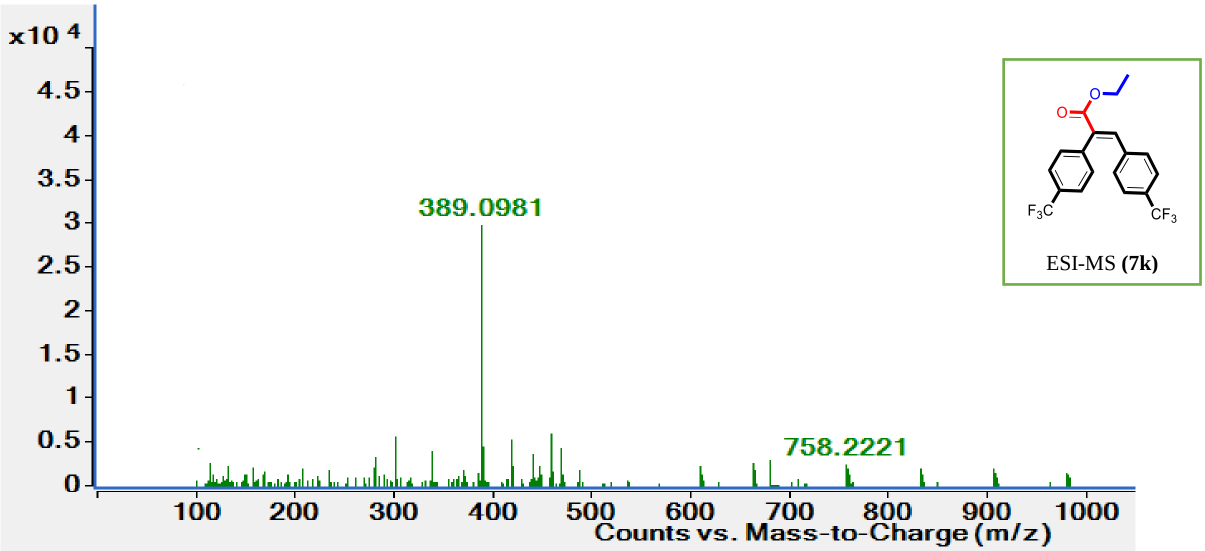

x10 4
4.5
4
3.5
3
2.5
2
1.5
1
0.5
0
389.0981
758.2221
100
200
300
400
500
600
700
800
900
1000
Counts vs. Mass-to-Charge (m/z)
O
O
F_3C
CF_3
ESI-MS (**7k**)

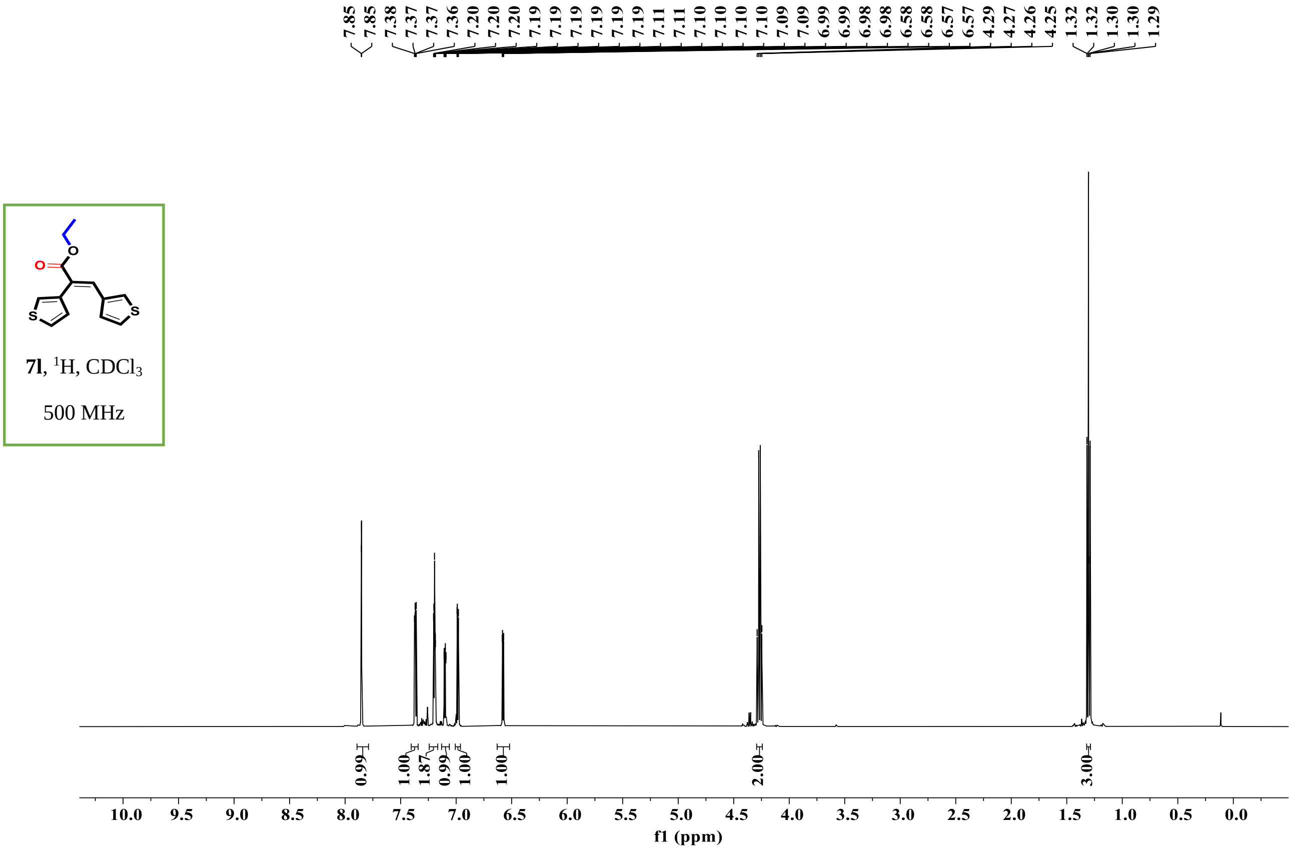

7.85 7.85 7.38 7.37 7.37 7.36 7.20 7.20 7.20 7.19 7.19 7.19 7.19 7.19 7.19 7.11 7.11 7.10 7.10 7.10 7.10 7.09 7.09 6.99 6.99 6.98 6.98 6.58 6.58 6.57 6.57 4.29 4.27 4.26 4.25 1.32 1.32 1.30 1.30 1.29
O
O
S
S
7l, ^1H, $CDCl_3$
500 MHz
0.99
1.00
1.87
0.99
1.00
1.00
2.00
3.00
10.0
9.5
9.0
8.5
8.0
7.5
7.0
6.5
6.0
5.5
5.0
4.5
4.0
3.5
3.0
2.5
2.0
1.5
1.0
0.5
0.0
f1 (ppm)

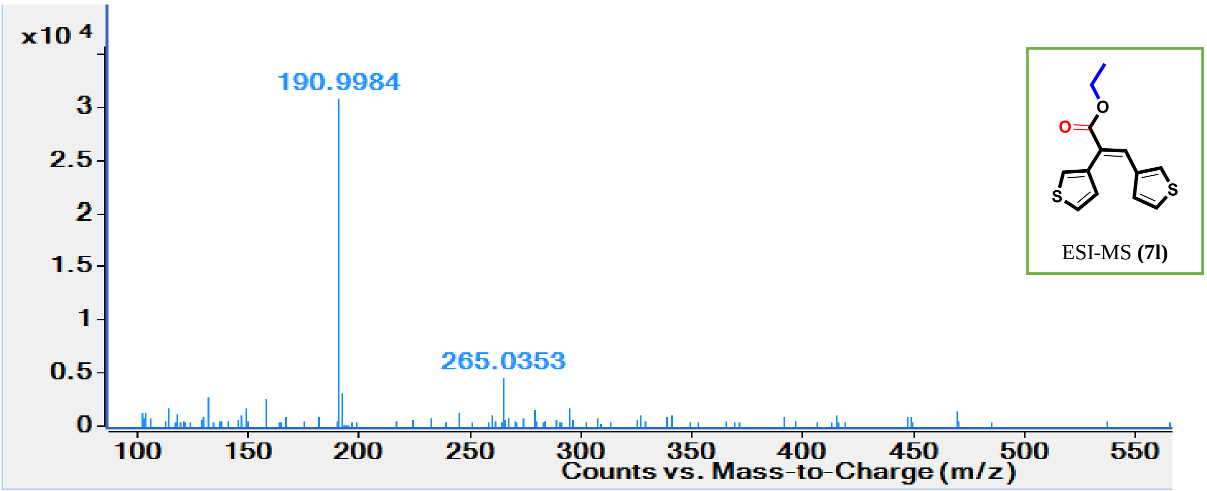
x10 4
190.9984
265.0353
Counts vs. Mass-to-Charge (m/z)
ESI-MS (7l)

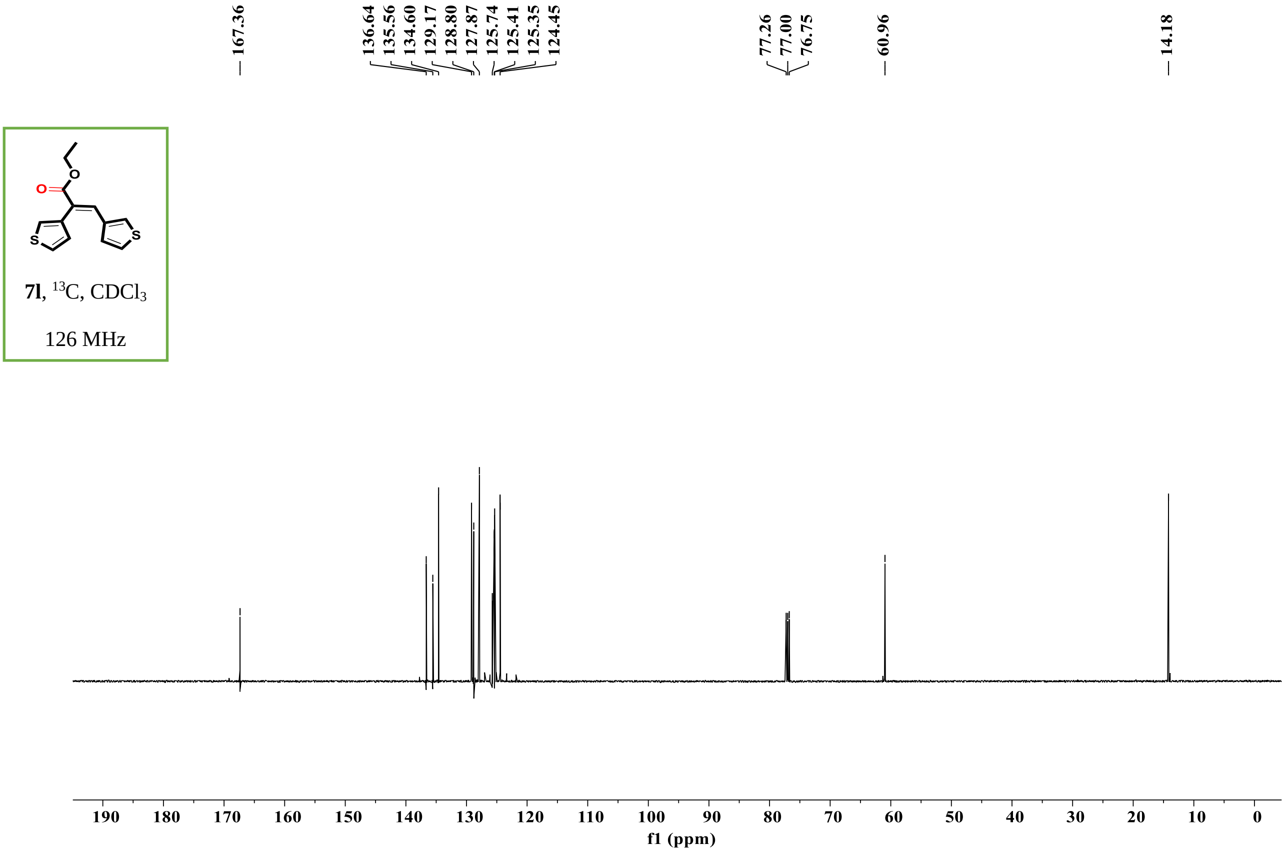
167.36
136.64
135.56
134.60
129.17
128.80
127.87
125.74
125.41
125.35
124.45
77.26
77.00
76.75
60.96
14.18
7l, 13C, CDCl3
126 MHz
f1 (ppm)

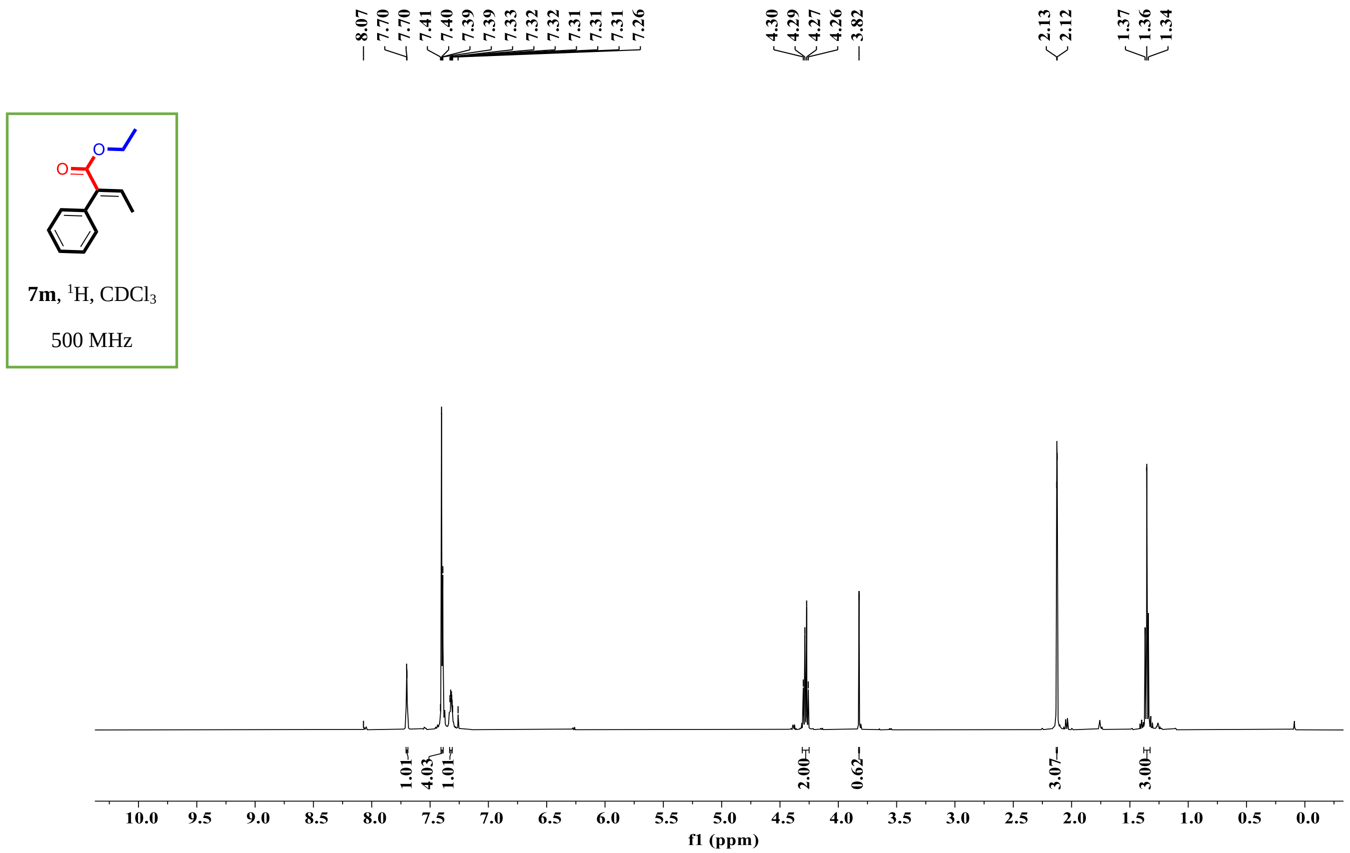

8.07
7.70
7.70
7.41
7.40
7.39
7.39
7.33
7.32
7.32
7.31
7.31
7.31
7.26
4.30
4.29
4.27
4.26
3.82
2.13
2.12
1.37
1.36
1.34
7m, ¹H, CDCl₃
500 MHz
1.01
4.03
1.01
2.00
0.62
3.07
3.00
10.0 9.5 9.0 8.5 8.0 7.5 7.0 6.5 6.0 5.5 5.0 4.5 4.0 3.5 3.0 2.5 2.0 1.5 1.0 0.5 0.0
f1 (ppm)

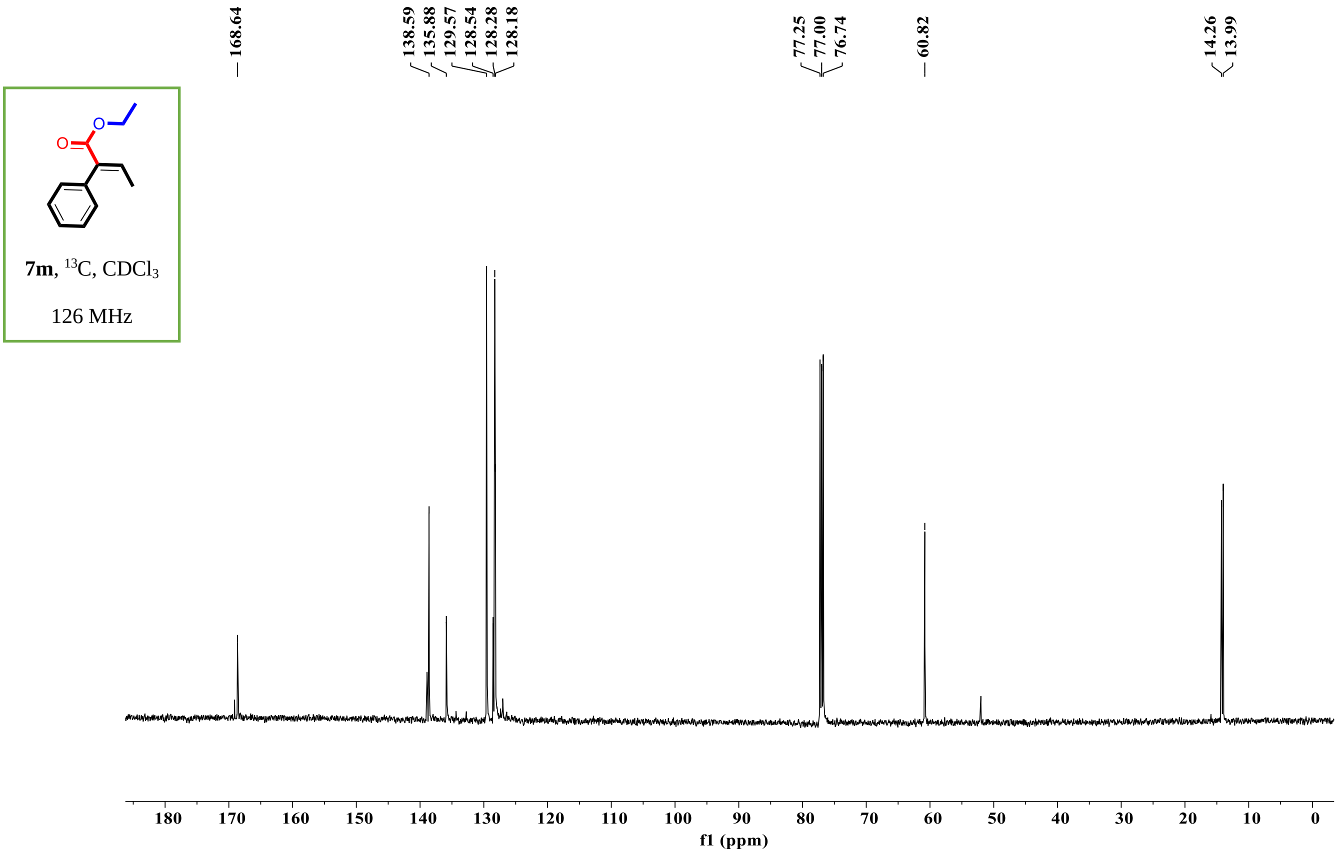

168.64
138.59
135.88
129.57
128.54
128.28
128.18
77.25
77.00
76.74
60.82
14.26
13.99
7m, ¹³C, CDCl₃
126 MHz
180 170 160 150 140 130 120 110 100 90 80 70 60 50 40 30 20 10 0
f1 (ppm)

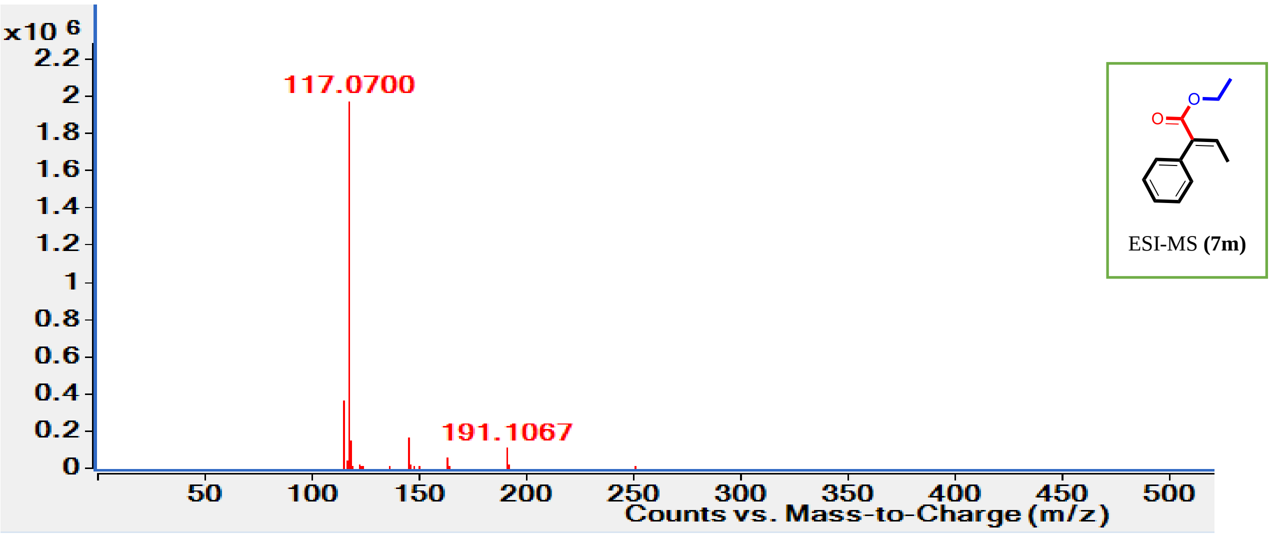
x10 6
2.2
2
1.8
1.6
1.4
1.2
1
0.8
0.6
0.4
0.2
0
117.0700
191.1067
50
100
150
200
250
300
350
400
450
500
Counts vs. Mass-to-Charge (m/z)
O
O

ESI-MS **(7m)**
